# Association between gut microbiota and preeclampsia-eclampsia: a two-sample Mendelian randomization study

**DOI:** 10.1186/s12916-022-02657-x

**Published:** 2022-11-15

**Authors:** Pengsheng Li, Haiyan Wang, Lan Guo, Xiaoyan Gou, Gengdong Chen, Dongxin Lin, Dazhi Fan, Xiaoling Guo, Zhengping Liu

**Affiliations:** 1 Foshan Fetal Medicine Research Institute, Affiliated Foshan Maternity & Child Healthcare Hospital, Southern Medical University, Foshan, China; 2 Department of Obstetrics, Affiliated Foshan Maternity & Child Healthcare Hospital, Southern Medical University, 11 Renminxi, Foshan, 528000 Guangdong China; 3 Biobank, Foshan Fetal Medicine Research Institute, Affiliated Foshan Maternity & Child Healthcare Hospital, Southern Medical University, Foshan, China; 4 Department of Epidemiology, School of Public Health, Sun Yat-sen University, Guangzhou, China

**Keywords:** Preeclampsia, Eclampsia, Gut microbiota, Causal inference, Mendelian randomization study

## Abstract

**Background:**

Several recent observational studies have reported that gut microbiota composition is associated with preeclampsia. However, the causal effect of gut microbiota on preeclampsia-eclampsia is unknown.

**Methods:**

A two-sample Mendelian randomization study was performed using the summary statistics of gut microbiota from the largest available genome-wide association study meta-analysis (*n*=13,266) conducted by the MiBioGen consortium. The summary statistics of preeclampsia-eclampsia were obtained from the FinnGen consortium R7 release data (5731 cases and 160,670 controls). Inverse variance weighted, maximum likelihood, MR-Egger, weighted median, weighted model, MR-PRESSO, and cML-MA were used to examine the causal association between gut microbiota and preeclampsia-eclampsia. Reverse Mendelian randomization analysis was performed on the bacteria that were found to be causally associated with preeclampsia-eclampsia in forward Mendelian randomization analysis. Cochran’s *Q* statistics were used to quantify the heterogeneity of instrumental variables.

**Results:**

Inverse variance weighted estimates suggested that *Bifidobacterium* had a protective effect on preeclampsia-eclampsia (odds ratio = 0.76, 95% confidence interval: 0.64–0.89, *P* = 8.03 × 10^−4^). In addition, *Collinsella* (odds ratio = 0.77, 95% confidence interval: 0.60–0.98, *P* = 0.03), *Enterorhabdus* (odds ratio = 0.76, 95% confidence interval: 0.62–0.93, *P* = 8.76 × 10^−3^), *Eubacterium (ventriosum group)* (odds ratio = 0.76, 95% confidence interval: 0.63–0.91, *P* = 2.43 × 10^−3^), *Lachnospiraceae (NK4A136 group)* (odds ratio = 0.77, 95% confidence interval: 0.65–0.92, *P* = 3.77 × 10^−3^), and *Tyzzerella 3* (odds ratio = 0.85, 95% confidence interval: 0.74–0.97, *P* = 0.01) presented a suggestive association with preeclampsia-eclampsia. According to the results of reverse MR analysis, no significant causal effect of preeclampsia-eclampsia was found on gut microbiota. No significant heterogeneity of instrumental variables or horizontal pleiotropy was found.

**Conclusions:**

This two-sample Mendelian randomization study found that *Bifidobacterium* was causally associated with preeclampsia-eclampsia. Further randomized controlled trials are needed to clarify the protective effect of probiotics on preeclampsia-eclampsia and their specific protective mechanisms.

**Supplementary Information:**

The online version contains supplementary material available at 10.1186/s12916-022-02657-x.

## Background

Preeclampsia and eclampsia (PE) are serious complications of pregnancy that affect 3–8% of pregnancies worldwide [1, 2] and are the leading causes of maternal and neonatal death [3, 4]. PE increases the risk of adverse pregnancy outcomes, including preterm birth and low birth weight [5]. It is also associated with serious maternal and child health problems, such as chronic hypertension, myocardial ischemia, and end-stage kidney disease in mothers [6, 7], as well as bronchopulmonary dysplasia and cognitive impairment in offspring [7, 8]. The pathogenesis of PE is still not fully understood. A variety of mechanisms including failure of spiral artery remodeling [9], imbalance of vascular endothelial growth factor (VEGF) and soluble fms-like tyrosine kinase 1 (sFlt1) [10], placental oxidative stress [11], and immune dysregulation [12] are believed to be involved. Moreover, PE is considered a progressive disease in which symptoms and organ function deteriorate over time and are cured only by delivery [1].

The gut microbiome has been observed to change significantly during pregnancy [13] and plays an important role in both maternal and fetal health [14]. Multiple studies have found that *Bifidobacterium* has a protective effect on PE [15–17]. Further research on probiotics and prebiotics may contribute to the prevention and treatment of PE. However, the results of published studies are not consistent. For example, unlike other studies, Altemani et al. found that *Bifidobacterium* increased in PE patients [16]. Miao and Lv et al. found that *Blautia* is a risk factor for PE [18, 19], while Chang and Yu reported the opposite result [20, 21]. Most previous studies were designed as case-control studies, and the timing of exposure and outcome is difficult to confirm. In addition, in observational studies, the association between gut microbiota and PE is susceptible to confounding factors such as age, environment, dietary patterns, and lifestyle [22], and it is difficult to effectively control these factors in an observational study. These conditions limit the causal inference between the gut microbiota and PE.

In this context, Mendelian randomization (MR) is a novel approach to explore the causal association between gut microbiota and PE. MR uses genetic variants to construct instrumental variables of exposure to estimate the causal association between exposure and disease outcome [23]. Because the allocation of genotypes from parent to offspring is random, the association between genetic variants and outcome is not affected by common confounding factors, and a causal sequence is reasonable [24]. MR has been widely applied to explore the causal association between gut microbiota and diseases, including metabolic diseases [25], autoimmune diseases [26], and rheumatoid arthritis [27]. In this study, using the genome-wide association study (GWAS) summary statistics from the MiBioGen and FinnGen consortiums, a two-sample MR analysis was conducted to evaluate the causal association between gut microbiota and PE.

## Methods

### Data sources

Genetic variants for gut microbiota were obtained from the largest genome-wide meta-analysis published to date for gut microbiota composition conducted by the MiBioGen consortium [28, 29]. The study included 18,340 individuals from 24 cohorts, most of whom had European ancestry (*n* = 13,266), targeting variable regions V4, V3–V4, and V1–V2 of the 16S rRNA gene to profile the microbial composition and to conduct taxonomic classification using direct taxonomic binning. Microbiota quantitative trait loci (mbQTL) mapping analysis was conducted to identify host genetic variants that were mapped to genetic loci associated with the abundance levels of bacterial taxa in the gut microbiota. In the study, genus was the lowest taxonomic level, and 131 genera with a mean abundance greater than 1% were identified, which included 12 unknown genera [28]. Therefore, 119 genus-level taxa were included in the current study for analysis. GWAS summary statistics for PE were obtained from FinnGen consortium R7 release data [30, 31]. The phenotype “pre-eclampsia or eclampsia” was adopted in the current study. This GWAS included 166,401 Finnish adult female subjects and consisted of 5731 cases and 160,670 controls. Sex, age, first 10 principal components, and genotyping batch were corrected during the analysis [30].

### Instrumental variable (IV)

The following selection criteria were used to choose the IVs: (1) single nucleotide polymorphisms (SNPs) associated with each genus at the locus-wide significance threshold (*P* < 1.0×10^–5^) were selected as potential IVs [25]; (2) 1000 Genomes project European samples data were used as the reference panel to calculate the linkage disequilibrium (LD) between the SNPs, and among those SNPs that had *R*^2^ < 0.001 (clumping window size=10,000 kb), only the SNPs with the lowest *P*-values were retained; (3) SNPs with minor allele frequency (MAF) ≤ 0.01 were removed; and (4) when palindromic SNPs existed, the forward strand alleles were inferred using allele frequency information.

### Statistical analysis

In this study, multiple methods including inverse variance weighted (IVW), maximum likelihood (ML), MR-Egger regression, weighted median, weighted model, MR-PRESSO, and cML-MA were used to examine whether there was a causal association between gut microbiota and PE. The IVW method used a meta-analysis approach combined with the Wald estimates for each SNP to obtain an overall estimate of the effect for gut microbiota on PE. If horizontal pleiotropy was not present, the IVW results would be unbiased [32]. The ML method is similar to IVW, assuming that heterogeneity and horizontal pleiotropy do not exist. If the hypotheses are satisfied, the results will be unbiased, and the standard errors will be smaller than IVW [33]. MR-Egger regression is based on the assumption of instrument strength independent of direct effect (InSIDE), which makes it possible to evaluate the existence of pleiotropy with the intercept term. If the intercept term is equal to zero, this indicates that horizontal pleiotropy does not exist and the result of the MR-Egger regression is consistent with IVW [34]. The weighted median method allows for the correct estimation of causal association when up to 50% of instrumental variables are invalid [35]. If the InSIDE hypothesis is violated, the weighted model estimate has been found to have greater power to detect a causal effect, less bias, and lower type I error rates than MR-Egger regression [35]. The MR-PRESSO analysis detects and attempts to reduce horizontal pleiotropy by removing significant outliers. But the MR-PRESSO outlier test requires that at least 50% of the genetic variants be valid instruments and relies on InSIDE assumptions [36]. A constrained maximum likelihood and model averaging-based MR method, cML-MA, which without relying on the InSIDE assumption, was used in this study to control correlated and uncorrelated pleiotropic effects [37].

Cochran’s IVW *Q* statistics were used to quantify the heterogeneity of IVs. In addition, to identify potential heterogeneous SNPs, the “leave-one-out” analysis was performed by omitting each instrumental SNP in turn. To assess the causal association between gut microbiota and PE, we also performed reverse MR analysis on the bacteria that were found to be causally associated with PE in forward MR analysis. The methods and settings adopted were consistent with those of forward MR.

The strength of IVs was assessed by calculating the *F*-statistic using the formula $F=\frac{R^2\times \left(N-1-K\right)}{\left(1-{R}^2\right)\times K}$, where *R*^2^ represents the proportion of variance in the exposure explained by the genetic variants, *N* represents sample size, and *K* represents the number of instruments [38]. If the corresponding *F*-statistic was >10, it was considered that there was no significant weak instrumental bias [38]. The power of the MR estimates was calculated using the online calculator tool [39] provided by Stephen Burgess [40].

False discovery rate (FDR) correction was conducted by applied *q*-value procedure, with a false discovery rate of *q*-value < 0.1 [41]. Genera of gut microbiota and PE were considered to have a suggestive association when *P* < 0.05 but *q* ≥ 0.1.

All statistical analyses were performed using R version 4.2.1 (R Foundation for Statistical Computing, Vienna, Austria). MR analyses were performed using the TwosampleMR (version 0.5.6) [42], MR-PRESSO (version 1.0) [36], MRcML [37], and qvalue [41] R packages.

## Results

According to the selection criteria of IVs, a total of 1232 SNPs were used as IVs for 119 bacterial genera. Details about the selected instrumental variables are shown in Additional file 1: Table S1.

**Fig. 1:**
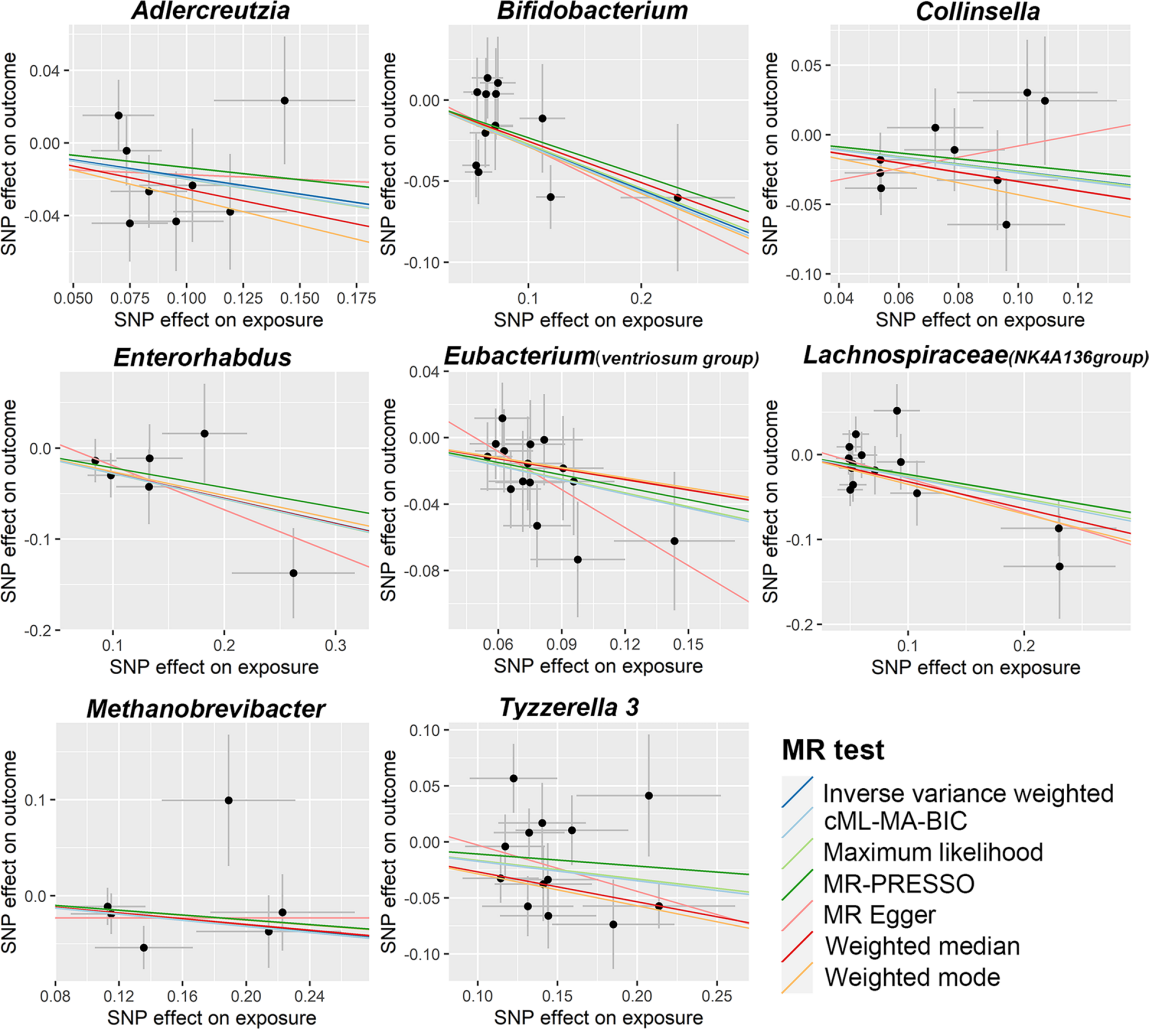
Scatter plots for the causal association between gut microbiota and PE

As shown in Table 1, Additional file 1: Table S2, and Fig. 1, eight bacterial genera, specifically, *Adlercreutzia*, *Bifidobacterium*, *Collinsella*, *Enterorhabdus*, *Eubacterium (ventriosum group)*, *Lachnospiraceae (NK4A136 group)*, *Methanobrevibacter*, and *Tyzzerella 3*, were found to be associated with PE in at least one MR method. IVW estimate suggests that *Bifidobacterium* had a protective effect on PE (OR = 0.76, 95% CI: 0.64–0.89, *P* = 8.03 × 10^−4^, *q* = 0.08), and the protective effect was still significant after considering the associated pleiotropy (cML-MA-BIC OR = 0.75, 95% CI: 0.64–0.89, *P* = 9.24 × 10^−4^, *q* = 0.04). The IVW estimate of *Lachnospiraceae (NK4A136 group)* also showed its suggestive protective effect against PE (OR = 0.77, 95% CI: 0.65–0.92, *P* = 3.77 × 10^−3^, *q* = 0.13), while ML (OR = 0.77, 95% CI: 0.66–0.91, *P* = 2.05 × 10^−3^, *q* = 0.07) and cML-MA estimate (OR = 0.77, 95% CI: 0.65–0.90, *P* = 1.37 × 10^−3^, *q* = 0.04) were still significant after FDR correction. Although IVW estimates did not support the causal associations of *Eubacterium (ventriosum group)* and *Tyzzerella 3* on PE after FDR correction (*q* > 0.1), both ML and cML-MA estimates suggested that *Eubacterium (ventriosum group)* (ML OR = 0.76, 95% CI: 0.63–0.91, *P* = 3.05 × 10^−3^, *q* = 0.07; cML-MA OR = 0.75, 95% CI: 0.63–0.90, *P* = 2.48 ×10^−3^, *q* = 0.05) and *Tyzzerella 3* (ML OR = 0.85, 95% CI: 0.76–0.94, *P* = 1.68×10^−3^, *q* = 0.07; cML-MA OR = 0.84, 95% CI: 0.76–0.93, *P* = 1.08×10^−3^, *q* =0.04) were causally associated with PE. The IVW estimates of *Collinsella* and *Enterorhabdus* showed a suggestive association with PE; however, these associations were no longer significant after FDR correction (*q* > 0.1). Similarly, the ML estimates of *Adlercreutzia* and *Methanobrevibacter* presented a suggestive association with PE.

**Table 1:** MR estimates for the association between gut microbiota and PE

Bacterial taxa (exposure)	MR method	No. of SNP	***F***-statistic	OR	95% CI	***P***-value	***q***-value
*Adlercreutzia*	IVW	8	103.69	0.83	0.68–1.01	0.06	0.61
	MR-Egger	8		0.95	0.37–2.45	0.92	1.00
	Weighted median	8		0.77	0.59–1.01	0.06	0.97
	Weighted mode	8		0.74	0.48–1.14	0.21	0.98
	ML	8		0.82	0.67–1.00	0.04	0.50
	cML-MA-BIC	8		0.82	0.68–1.00	0.05	0.39
*Bifidobacterium*	IVW	13	115.25	0.76	0.64–0.89	8.03E−04	0.08
	MR-Egger	13		0.71	0.47–1.08	0.14	1.00
	Weighted median	13		0.78	0.61–0.98	0.04	0.97
	Weighted mode	13		0.75	0.54–1.03	0.10	0.98
	ML	13		0.76	0.65–0.90	1.29E−03	0.07
	cML-MA-BIC	13		0.75	0.64–0.89	9.24E−04	0.04
*Collinsella*	IVW	9	104.60	0.77	0.60–0.98	0.03	0.61
	MR-Egger	9		1.50	0.60–3.75	0.42	1.00
	Weighted median	9		0.71	0.51–1.01	0.05	0.97
	Weighted mode	9		0.65	0.38–1.12	0.16	0.98
	ML	9		0.77	0.60–0.99	0.04	0.50
	cML-MA-BIC	9		0.76	0.59–0.98	0.03	0.39
*Enterorhabdus*	IVW	6	194.91	0.76	0.62–0.93	8.76E−03	0.23
	MR-Egger	6		0.62	0.36–1.07	0.16	1.00
	Weighted median	6		0.76	0.57–1.01	0.06	0.97
	Weighted mode	6		0.77	0.51–1.16	0.27	0.98
	ML	6		0.75	0.61–0.93	8.78E−03	0.17
	cML-MA-BIC	6		0.76	0.61–0.93	9.40E−03	0.15
*Eubacterium (ventriosum group)*	IVW	15	90.27	0.76	0.63–0.91	2.43E−03	0.13
	MR-Egger	15		0.47	0.21–1.03	0.08	1.00
	Weighted median	15		0.81	0.63–1.04	0.10	1.00
	Weighted mode	15		0.82	0.53–1.26	0.38	0.98
	ML	15		0.76	0.63–0.91	3.05E−03	0.07
	cML-MA-BIC	15		0.75	0.63–0.90	2.48E−03	0.05
*Lachnospiraceae (NK4A136 group)*	IVW	15	86.22	0.77	0.65–0.92	3.77E−03	0.13
	MR-Egger	15		0.67	0.47–0.94	0.04	1.00
	Weighted median	15		0.73	0.57–0.92	9.20E−03	0.55
	Weighted mode	15		0.71	0.52–0.95	0.04	0.98
	ML	15		0.77	0.66–0.91	2.05E−03	0.07
	cML-MA-BIC	15		0.77	0.65–0.90	1.37E−03	0.04
*Methanobrevibacter*	IVW	6	137.60	0.86	0.73–1.01	0.06	0.61
	MR-Egger	6		1.00	0.51–1.96	1.00	1.00
	Weighted median	6		0.86	0.71–1.05	0.13	1.00
	Weighted mode	6		0.88	0.69–1.12	0.35	0.98
	ML	6		0.85	0.73–0.99	0.04	0.50
	cML-MA-BIC	6		0.85	0.73–0.99	0.04	0.39
*Tyzzerella 3*	IVW	13	85.50	0.85	0.74–0.97	0.01	0.27
	MR-Egger	13		0.66	0.36–1.21	0.21	1.00
	Weighted median	13		0.77	0.66–0.89	6.00E−04	0.07
	Weighted mode	13		0.75	0.62–0.92	0.02	0.98
	ML	13		0.85	0.76–0.94	1.68E−03	0.07
	cML-MA-BIC	13		0.84	0.76–0.93	1.08E−03	0.04

*MR* Mendelian randomization, *PE* preeclampsia or eclampsia, *SNP* single nucleotide polymorphism, *OR* odds ratio, *CI* confidence interval, *IVW* inverse variance weighted, *ML* maximum likelihood

Among these eight causal associations, the *F*-statistics of the IVs ranged from 85.50 to 194.91, eliminating the bias of weak IVs. The results of Cochran’s IVW *Q* test showed no significant heterogeneity of these IVs (Additional file 1: Tables S3). In addition, there was no significant directional horizontal pleiotropy according to the results of the MR-Egger regression intercept analysis (Additional file 1: Table S4).

**Fig. 2:**
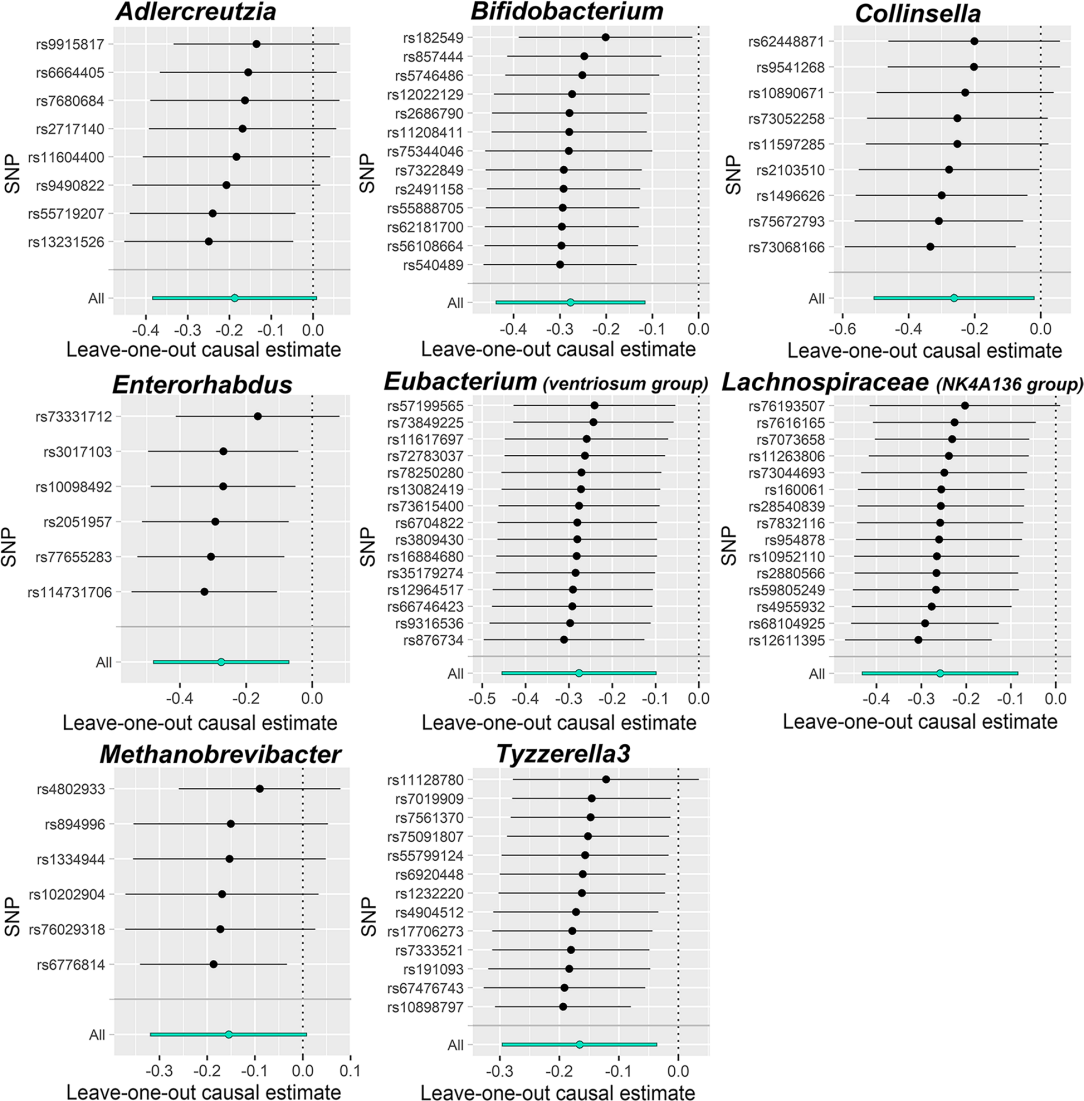
Leave-one-out plots for the causal association between gut microbiota and PE

There were potential outliers of the IVs of *Adlercreutzia*, *Methanobrevibacter*, and *Collinsella* that were present on visual inspection in scatter plots (Fig. 1) and leave-one-out plots (Fig. 2). However, further MR-PRESSO analysis did not find any significant outliers (global test *P*>0.05, Additional file 1: Tables S5). Therefore, there was insufficient evidence for horizontal pleiotropy in the association between these bacteria and PE.

According to the results of reverse MR analysis, there was a suggestive association between PE and *Collinsella* (IVW OR = 0.94, 95% CI: 0.88–1.00, *P* = 0.04); however, such association became insignificant after correction for FDR (*q* = 0.33). No significant causal association was found between PE and the other gut microbiota (Additional file 1: Tables S6 and S7). Cochran’s IVW *Q* test showed that there was no significant heterogeneity in PE IVs (Additional file 1: Table S8). The results of MR-Egger regression intercepted item analysis (Additional file 1: Table S9) and MR-PRESSO analysis (Additional file 1: Table S10) also did not find significant horizontal pleiotropy.

## Discussion

In this study, using the summary statistics of gut microbiota from the largest GWAS meta-analysis conducted by the MiBioGen consortium and the summary statistics of PE from the FinnGen consortium R7 release data, we performed a two-sample MR analysis to evaluate the causal association between gut microbiota and PE. We found that *Bifidobacterium* had protective effects on PE, and several genera of gut microbiota had suggestive protective effects against PE, including *Collinsella*, *Enterorhabdus*, *Eubacterium (ventriosum group)*, *Lachnospiraceae (NK4A136 group)*, and *Tyzzerella 3*.

A number of observational studies have reported the association between gut microbiota and PE [16–19, 43–46]. *Bifidobacterium* was found to be associated with a lower risk of PE, which is consistent with the results of our study [18, 20]. *Bifidobacterium*, as a probiotic, has been widely reported to have a protective effect on cardiovascular [47] and metabolic diseases [48]. Consistent with previous studies [20, 21, 45], we also found that *Lachnospiraceae (NK4A136 group)*, butyrate-producing bacteria [49], reduced the risk of PE. Elevated levels of trimethylamine n-oxide (TMAO) and its precursor trimethylamine (TMA) were found in PE patients [44, 50], which could induce spiral arterial remodeling defects by increasing sFlt-1 and reactive oxygen species (ROS) levels in the placenta [51]. As methanogenic archaea, *Methanobrevibacter* can convert TMA to methane [52] and thereby reduce the risk of PE [19]. In addition, we also found that *Eubacterium (ventriosum group)*, *Enterorhabdus*, and *Tyzzerella 3* were associated with PE. *Eubacterium (ventriosum group)* can increase the level of SCFA and thus decrease visceral fat accumulation [53]; furthermore, some other species of *Eubacterium*, such as *E. rectale* and *E. hallii*, were found to have a protective effect on PE [20]. There have been relatively few previous studies on *Tyzzerella 3*, but a reduced abundance of *Tyzzerella 3* has been reported to be associated with acute myocardial infarction [54], which may be related to its ability to produce formic and butyric acid [55].

SCFAs—mostly acetic acid, propionic acid, and butyric acid—are the main end products of gut microbiota metabolism in the human body. In this study, part of the gut microbiota identified to be associated with PE were SCFA-producing bacteria, including *Bifidobacterium* [56], *Collinsella* [20], *Eubacterium (ventriosum group)* [57], *Lachnospiraceae (NK4A136 group)* [49], and *Tyzzerella 3* [55]. Several clinical and animal studies have reported that SCFA metabolized by gut microbiota can effectively reduce blood pressure [58–60]. SCFA can be involved in blood pressure regulation through a variety of mechanisms, but mainly through the activation of transmembrane G protein-coupled receptors (GPCR), including CPR41, CPR43, and olfactory receptor 78 (Olfr78) [60]. Acetic acid and butyric acid can improve endothelial function by restoring Th17/Treg imbalance and alleviating arterial inflammation [61]. Furthermore, butyric acid can directly activate colonic vagus signal transduction via the GPR41/43 receptor [62]. Altemani et al. found reduced levels of serum butyric acid in patients with late-onset preeclampsia and also found the gene abundance of butyryl-CoA: acetate CoA transferase (*but*) and butyrate kinase (*buk*) to be decreased in the gut microbiome, suggesting that a reduction in the level of butyric acid produced by gut microbiota is related to preeclampsia [16]. Yong et al. report that sodium butyrate improves hypertension and proteinuria in PE rats and found that sodium butyrate alleviates PE symptoms by decreasing placental antiangiogenic factors (sFlt1 and soluble endoglin [sEng]) and increasing angiogenic factors (placental growth factor [PLGF]), while reducing placental and intestinal inflammation [63]. In addition, Gomez-Arango et al. found that plasminogen activator inhibitor 1 (PAI-1) levels are positively correlated with blood pressure but negatively correlated with *buk* expression in obese pregnant women, suggesting that SCFAs produced by gut microbiota may also regulate blood pressure through PAI-1 [64].

The maintenance of intestinal barrier function depends on the balance of pathogenic bacteria and probiotics [65]. Chen et al. found that the opportunistic pathogens *Fusobacterium* and *Veillonella* are increased in preeclampsia patients. They further gavaged mice with fecal supernatants from preeclampsia patients, which gave the mice clinical and placental pathological features similar to PE [17]. Impaired intestinal barrier function can increase the entry of LPS produced by gut microbiota into the blood [65], triggering placental inflammation, leading to deficient trophoblast invasion and spiral artery remodeling [66]. Although the present study did not find a causal effect of bacteria, which were previously reported to impair the intestinal barrier in PE, some probiotics such as *Bifidobacterium* have been reported to stimulate the expression of Mucins 3 in intestinal epithelial cells [67] and restore mucus growth [68], thereby maintaining intestinal barrier function. In addition, some SCFAs produced by probiotics, for example butyric acid, are chief energy sources of intestinal epithelial cells, and they participate in cell proliferation and differentiation, thereby maintaining cell homeostasis through anti-inflammatory and antioxidant effects [69, 70]. Therefore, probiotics and SCFAs may help pregnant women maintain intestinal barrier function and prevent placental inflammation caused by the migration of pathogenic bacteria to reduce the risk of PE. Nevertheless, further randomized controlled trials are needed to confirm these findings.

This study has several strengths. MR analysis was performed to determine the causal association between gut microbiota and PE, thus excluding the interference of confounding factors and reversing causation on causal inference. Genetic variants of gut microbiota were obtained from the largest available GWAS meta-analysis, ensuring the strength of instruments in the MR analysis. Horizontal pleiotropy was detected and excluded by using the MR-PRESSO and MR-Egger regression intercept term tests. Furthermore, cML-MA was used to rule out the bias caused by correlated and uncorrelated pleiotropy. A two-sample MR design was adopted and non-overlapping exposure and outcome summary-level data were used to avoid bias [71].

However, there are also several limitations in this study, which should be noted while interpreting the results. Because summary statistics rather than raw data were used in the analysis, it was not possible to perform subgroup analyses, such as distinguishing early-onset preeclampsia and late-onset preeclampsia, or exploring non-linear relationships. Since the lowest taxonomic level in the exposure dataset was genus, this restriction prevented us from further exploring the causal association between gut microbiota and PE at the species level. To conduct sensitivity analysis and horizontal pleiotropy detection, more genetic variations need to be included as instrumental variables; therefore, SNP used in the analysis did not reach the traditional GWAS significance threshold (*P* < 5×10^–8^). For this, we used FDR correction to restrict the possibility of false positives. The sample size of gut microbiota was relatively small, so the results of reverse MR analysis may have been affected by weak instrumental bias, and a reverse causal association could not be completely excluded. The GWAS meta-analysis for gut microbiota was not restricted to female participants [28]. Although the genetic variants located on the sex chromosomes were excluded, as well as sex was adjusted in the analysis [28], the potential bias due to sex could not be excluded. Although most participants in the GWAS meta-analysis for gut microbiota data were of European descent, there may still be interference from population stratification, and the results of this study may not be entirely applicable to subjects of non-European descent [72]. Future MR studies on the causal association between gut microbiota and PE could be considered in diverse European and non-European populations for better generalizability.

## Conclusions

In summary, this two-sample MR study found that *Bifidobacterium* was causally associated with PE. Further RCT studies are needed to clarify the protective effect of probiotics on PE and its specific protective mechanism. In addition, although reverse MR estimates did not support the causal association between PE and gut microbiota, it cannot be ruled out that PE may affect the intestinal microecology; this again needs to be confirmed by further studies.

## Supplementary Information

**Additional file 1: Table S1.** Instrumental variables used in MR analysis of the association between gut microbiota and PE. **Table S2.** Full result of MR estimates for the association between gut microbiota and PE. **Table S3.** The heterogeneity of gut microbiota instrumental variables. **Table S4.** Directional horizontal pleiotropy assessed by intercept term in MR Egger regression of the association between gut microbiota and PE. **Table S5.** MR-PRESSO analysis for the association between gut microbiota and PE. **Table S6.** Instrumental variables used in the MR analysis of the association between PE and gut microbiota. **Table S7.** Full result of MR estimates for the association between PE and gut microbiota. **Table S8.** The heterogeneity of gut microbiota instrumental variables. **Table S9.** Directional horizontal pleiotropy assessed by intercept term in MR Egger regression of the association between PE and gut microbiota. **Table S10.** MR-PRESSO analysis for the association between PE and gut microbiota.

## Data Availability

The datasets analyzed during the current study are available in the MiBioGen repository, https://mibiogen.gcc.rug.nl/ [28, 29], and the FinnGen repository, https://r7.finngen.fi/ [30, 31].

## Abbreviations

buk: Butyrate kinase
but: Butyryl-CoA: acetate CoA transferase
CI: Confidence interval
FDR: False discovery rate
GPCR: G protein-coupled receptors
GWAS: Genome-wide association study
InSIDE: Instrument strength independent of direct effect
IV: Instrumental variable
IVW: Inverse variance weighted
LD: Linkage disequilibrium
MAF: Minor allele frequency
mbQTL: Microbiota quantitative trait loci
ML: Maximum likelihood
MR: Mendelian randomization
Olfr: Olfactory receptor
OR: Odds ratio
PAI: Plasminogen activator inhibitor
PE: Preeclampsia and eclampsia
PLGF: Placental growth factor
ROS: Reactive oxygen species
SCFA: Short-chain fatty acid
sEng: Soluble endoglin
sFlt1: Soluble fms-like tyrosine kinase 1
SNP: Single nucleotide polymorphism
TMA: Trimethylamine
TMAO: Trimethylamine n-oxide
VEGF: Vascular endothelial growth factor
