# Supplementary material for: Association between gut microbiota and preeclampsia-eclampsia: a two-sample Mendelian randomization study
Source: BMC Med. 2022 Nov 15;20:443. doi: 10.1186/s12916-022-02657-x (PMC9667679; doi:10.1186/s12916-022-02657-x)
Supplement: Supplementary file 1 — Additional file 1: Table S1. Instrumental variables used in MR analysis of the association between gut microbiota and PE. Table S2. Full result of MR estimates for the association between gut microbiota and PE. Table S3. The heterogeneity of gut microbiota instrumental variables. Table S4. Directional horizontal pleiotropy assessed by intercept term in MR Egger regression of the association between gut microbiota and PE. Table S5. MR-PRESSO analysis for the association between gut microbiota and PE. Table S6. Instrumental variables used in the MR analysis of the association between PE and gut microbiota. Table S7. Full result of MR estimates for the association between PE and gut microbiota. Table S8. The heterogeneity of gut microbiota instrumental variables. Table S9. Directional horizontal pleiotropy assessed by intercept term in MR Egger regression of the association between PE and gut microbiota. Table S10. MR-PRESSO analysis for the association between PE and gut microbiota. [file 12916_2022_2657_MOESM1_ESM.docx]

| **Table S1 Instrumental variables used in MR analysis of the association between gut microbiota and PE.** | | | | | | | | | | | |
| --- | --- | --- | --- | --- | --- | --- | --- | --- | --- | --- | --- |
| **Bacterial taxa (exposure)** | **SNP** | **Effect allele** | **Other allele** | **MAF** | **Exposure (Bacteria)** | | | **Outcome (PE)** | | |  |
|  |  |  |  |  | **Beta** | **SE** | **P-value** | **Beta** | **SE** | **P-value** | |
| *Actinomyces* | rs34583783 | G | T | 0.061 | 0.127 | 0.027 | 4.49E-06 | 0.072 | 0.040 | 0.07 | |
| *Actinomyces* | rs4146653 | G | A | 0.142 | 0.099 | 0.021 | 4.50E-06 | 0.029 | 0.028 | 0.29 | |
| *Actinomyces* | rs7915461 | T | C | 0.069 | 0.188 | 0.040 | 5.92E-06 | 0.097 | 0.037 | 0.01 | |
| *Actinomyces* | rs2715439 | C | T | 0.471 | 0.075 | 0.016 | 6.27E-06 | -0.035 | 0.019 | 0.07 | |
| *Actinomyces* | rs35011108 | A | G | 0.068 | 0.233 | 0.051 | 6.34E-06 | -0.047 | 0.038 | 0.22 | |
| *Actinomyces* | rs4073240 | G | A | 0.379 | 0.075 | 0.017 | 7.94E-06 | 0.013 | 0.020 | 0.52 | |
| *Actinomyces* | rs71315246 | A | G | 0.136 | -0.097 | 0.022 | 9.83E-06 | 0.018 | 0.028 | 0.52 | |
| *Adlercreutzia* | rs7680684 | C | T | 0.343 | -0.083 | 0.017 | 9.77E-07 | 0.027 | 0.020 | 0.18 | |
| *Adlercreutzia* | rs2717140 | C | T | 0.099 | -0.119 | 0.025 | 2.05E-06 | 0.038 | 0.032 | 0.23 | |
| *Adlercreutzia* | rs9490822 | C | T | 0.451 | -0.073 | 0.016 | 2.54E-06 | 0.004 | 0.019 | 0.82 | |
| *Adlercreutzia* | rs13231526 | C | A | 0.080 | 0.143 | 0.031 | 4.81E-06 | 0.023 | 0.035 | 0.51 | |
| *Adlercreutzia* | rs6664405 | T | C | 0.146 | -0.095 | 0.021 | 5.23E-06 | 0.043 | 0.027 | 0.12 | |
| *Adlercreutzia* | rs9915817 | T | C | 0.292 | 0.075 | 0.017 | 8.22E-06 | -0.044 | 0.021 | 0.04 | |
| *Adlercreutzia* | rs55719207 | G | A | 0.401 | -0.070 | 0.016 | 9.61E-06 | -0.015 | 0.020 | 0.44 | |
| *Adlercreutzia* | rs11604400 | C | T | 0.106 | -0.103 | 0.023 | 9.74E-06 | 0.023 | 0.031 | 0.45 | |
| *Akkermansia* | rs11729256 | T | C | 0.170 | 0.075 | 0.015 | 6.58E-07 | 0.005 | 0.025 | 0.83 | |
| *Akkermansia* | rs4936098 | A | G | 0.349 | 0.065 | 0.014 | 1.10E-06 | 0.005 | 0.020 | 0.80 | |
| *Akkermansia* | rs74542928 | T | C | 0.047 | 0.113 | 0.024 | 1.48E-06 | 0.051 | 0.045 | 0.26 | |
| *Akkermansia* | rs12908520 | G | A | 0.425 | 0.062 | 0.013 | 2.26E-06 | -0.021 | 0.019 | 0.29 | |
| *Akkermansia* | rs9349825 | A | G | 0.189 | -0.070 | 0.015 | 2.60E-06 | 0.009 | 0.025 | 0.72 | |
| *Akkermansia* | rs2602429 | C | T | 0.261 | 0.075 | 0.016 | 2.72E-06 | 0.011 | 0.022 | 0.61 | |
| *Akkermansia* | rs4242783 | G | A | 0.284 | 0.069 | 0.015 | 3.00E-06 | 0.032 | 0.021 | 0.13 | |
| *Akkermansia* | rs117107102 | A | G | 0.047 | 0.204 | 0.043 | 3.01E-06 | 0.094 | 0.045 | 0.04 | |
| *Akkermansia* | rs111862613 | T | C | 0.177 | 0.091 | 0.020 | 3.39E-06 | 0.009 | 0.025 | 0.71 | |
| *Akkermansia* | rs61779207 | G | A | 0.223 | -0.076 | 0.017 | 6.32E-06 | 0.001 | 0.023 | 0.95 | |
| *Akkermansia* | rs941682 | G | A | 0.273 | -0.063 | 0.014 | 9.17E-06 | -0.005 | 0.021 | 0.81 | |
| *Alistipes* | rs11769002 | G | A | 0.415 | -0.053 | 0.011 | 1.45E-06 | -0.027 | 0.019 | 0.16 | |
| *Alistipes* | rs7129639 | C | A | 0.335 | -0.052 | 0.011 | 1.78E-06 | -0.026 | 0.020 | 0.20 | |
| *Alistipes* | rs1107244 | G | A | 0.078 | 0.076 | 0.017 | 3.59E-06 | -0.031 | 0.036 | 0.39 | |
| *Alistipes* | rs8130320 | A | G | 0.558 | -0.049 | 0.011 | 4.84E-06 | 0.037 | 0.019 | 0.06 | |
| *Alistipes* | rs1689282 | A | C | 0.323 | -0.052 | 0.011 | 5.28E-06 | 0.022 | 0.020 | 0.28 | |
| *Alistipes* | rs34417064 | A | G | 0.452 | -0.048 | 0.011 | 7.01E-06 | 0.023 | 0.019 | 0.23 | |
| *Alistipes* | rs2450745 | A | C | 0.069 | -0.081 | 0.018 | 7.12E-06 | -0.003 | 0.038 | 0.93 | |
| *Alistipes* | rs4810359 | A | G | 0.123 | -0.065 | 0.015 | 7.50E-06 | 0.020 | 0.029 | 0.49 | |
| *Alistipes* | rs12990744 | C | T | 0.103 | -0.078 | 0.017 | 8.21E-06 | 0.041 | 0.032 | 0.20 | |
| *Alistipes* | rs2875322 | T | C | 0.171 | -0.058 | 0.013 | 8.78E-06 | -0.030 | 0.026 | 0.24 | |
| *Alistipes* | rs2290844 | C | T | 0.113 | 0.081 | 0.019 | 9.10E-06 | 0.044 | 0.030 | 0.14 | |
| *Alistipes* | rs11958296 | A | G | 0.045 | -0.098 | 0.022 | 9.30E-06 | 0.065 | 0.047 | 0.17 | |
| *Allisonella* | rs602075 | A | G | 0.264 | 0.169 | 0.030 | 3.57E-08 | 0.007 | 0.022 | 0.75 | |
| *Allisonella* | rs35778461 | C | T | 0.228 | 0.147 | 0.030 | 1.21E-06 | 0.022 | 0.023 | 0.35 | |
| *Allisonella* | rs6742198 | G | A | 0.243 | 0.149 | 0.032 | 3.35E-06 | -0.003 | 0.022 | 0.90 | |
| *Allisonella* | rs1901739 | T | G | 0.536 | 0.116 | 0.025 | 3.59E-06 | 0.006 | 0.019 | 0.76 | |
| *Allisonella* | rs35110698 | T | C | 0.147 | -0.146 | 0.032 | 5.72E-06 | -0.019 | 0.027 | 0.49 | |
| *Allisonella* | rs76904847 | G | A | 0.175 | 0.149 | 0.033 | 6.09E-06 | -0.005 | 0.025 | 0.85 | |
| *Allisonella* | rs7898615 | T | G | 0.137 | 0.168 | 0.037 | 8.87E-06 | -0.035 | 0.028 | 0.21 | |
| *Allisonella* | rs594561 | C | T | 0.512 | 0.112 | 0.025 | 9.41E-06 | -0.015 | 0.019 | 0.43 | |
| *Alloprevotella* | rs4680035 | A | G | 0.614 | -0.120 | 0.026 | 4.99E-06 | -0.037 | 0.020 | 0.06 | |
| *Alloprevotella* | rs58212166 | A | G | 0.184 | -0.162 | 0.036 | 7.94E-06 | -0.012 | 0.025 | 0.63 | |
| *Alloprevotella* | rs2154444 | T | G | 0.263 | 0.138 | 0.031 | 8.37E-06 | 0.022 | 0.022 | 0.30 | |
| *Alloprevotella* | rs4364940 | A | G | 0.305 | 0.126 | 0.028 | 8.58E-06 | -0.037 | 0.021 | 0.07 | |
| *Alloprevotella* | rs34619204 | G | A | 0.173 | -0.156 | 0.034 | 8.84E-06 | 0.053 | 0.025 | 0.04 | |
| *Anaerofilum* | rs816292 | T | C | 0.295 | -0.113 | 0.022 | 2.64E-07 | 0.050 | 0.021 | 0.02 | |
| *Anaerofilum* | rs79598899 | C | T | 0.047 | 0.183 | 0.036 | 3.75E-07 | -0.033 | 0.046 | 0.47 | |
| *Anaerofilum* | rs712981 | A | C | 0.383 | 0.101 | 0.020 | 6.83E-07 | -0.002 | 0.020 | 0.92 | |
| *Anaerofilum* | rs4506496 | G | A | 0.293 | 0.103 | 0.021 | 1.49E-06 | -0.024 | 0.021 | 0.27 | |
| *Anaerofilum* | rs10794359 | T | C | 0.467 | -0.095 | 0.020 | 2.23E-06 | -0.021 | 0.019 | 0.29 | |
| *Anaerofilum* | rs17096874 | C | T | 0.211 | -0.126 | 0.027 | 2.86E-06 | 0.000 | 0.023 | 0.99 | |
| *Anaerofilum* | rs1563175 | A | C | 0.457 | 0.092 | 0.020 | 5.54E-06 | 0.033 | 0.019 | 0.09 | |
| *Anaerofilum* | rs356049 | G | A | 0.069 | 0.133 | 0.029 | 6.56E-06 | 0.037 | 0.039 | 0.33 | |
| *Anaerofilum* | rs17012738 | T | G | 0.490 | 0.090 | 0.020 | 7.24E-06 | 0.016 | 0.019 | 0.39 | |
| *Anaerofilum* | rs9299345 | T | C | 0.096 | -0.136 | 0.030 | 8.04E-06 | -0.013 | 0.032 | 0.68 | |
| *Anaerofilum* | rs4244069 | G | A | 0.126 | -0.147 | 0.033 | 9.81E-06 | -0.031 | 0.029 | 0.28 | |
| *Anaerostipes* | rs7193624 | C | T | 0.083 | 0.075 | 0.015 | 5.35E-07 | 0.047 | 0.035 | 0.17 | |
| *Anaerostipes* | rs62157625 | T | C | 0.124 | 0.089 | 0.019 | 1.45E-06 | -0.062 | 0.029 | 0.04 | |
| *Anaerostipes* | rs62215703 | G | A | 0.215 | 0.064 | 0.014 | 1.98E-06 | -0.011 | 0.023 | 0.64 | |
| *Anaerostipes* | rs2804244 | A | G | 0.383 | -0.053 | 0.011 | 2.04E-06 | -0.034 | 0.020 | 0.08 | |
| *Anaerostipes* | rs3900776 | G | A | 0.028 | -0.110 | 0.024 | 2.75E-06 | -0.094 | 0.058 | 0.11 | |
| *Anaerostipes* | rs2396460 | T | C | 0.486 | -0.051 | 0.011 | 2.91E-06 | 0.001 | 0.019 | 0.94 | |
| *Anaerostipes* | rs6854026 | T | C | 0.562 | -0.051 | 0.011 | 3.20E-06 | -0.007 | 0.019 | 0.73 | |
| *Anaerostipes* | rs6726833 | C | A | 0.079 | -0.088 | 0.019 | 3.32E-06 | 0.013 | 0.035 | 0.71 | |
| *Anaerostipes* | rs60983350 | G | A | 0.362 | -0.054 | 0.012 | 4.42E-06 | 0.025 | 0.021 | 0.22 | |
| *Anaerostipes* | rs2014785 | T | C | 0.442 | 0.052 | 0.011 | 4.68E-06 | 0.026 | 0.019 | 0.19 | |
| *Anaerostipes* | rs78735375 | A | C | 0.040 | -0.137 | 0.031 | 5.33E-06 | 0.065 | 0.049 | 0.18 | |
| *Anaerostipes* | rs6474958 | A | G | 0.308 | -0.050 | 0.011 | 6.74E-06 | 0.007 | 0.021 | 0.74 | |
| *Anaerostipes* | rs10502061 | A | G | 0.114 | 0.084 | 0.019 | 7.94E-06 | -0.010 | 0.030 | 0.75 | |
| *Anaerotruncus* | rs6563550 | T | C | 0.078 | 0.088 | 0.018 | 2.35E-07 | -0.030 | 0.036 | 0.39 | |
| *Anaerotruncus* | rs8005030 | C | T | 0.332 | 0.055 | 0.012 | 2.28E-06 | -0.035 | 0.020 | 0.08 | |
| *Anaerotruncus* | rs4669806 | G | T | 0.214 | 0.058 | 0.012 | 2.42E-06 | -0.005 | 0.023 | 0.84 | |
| *Anaerotruncus* | rs9347879 | T | C | 0.489 | 0.051 | 0.011 | 4.22E-06 | -0.008 | 0.019 | 0.67 | |
| *Anaerotruncus* | rs1272208 | G | T | 0.238 | -0.061 | 0.013 | 4.28E-06 | 0.013 | 0.023 | 0.56 | |
| *Anaerotruncus* | rs11018566 | A | G | 0.053 | -0.156 | 0.037 | 6.14E-06 | -0.014 | 0.043 | 0.74 | |
| *Anaerotruncus* | rs6494922 | A | G | 0.053 | 0.090 | 0.020 | 6.62E-06 | -0.037 | 0.043 | 0.39 | |
| *Anaerotruncus* | rs10150232 | A | G | 0.200 | 0.057 | 0.012 | 6.68E-06 | -0.006 | 0.024 | 0.80 | |
| *Anaerotruncus* | rs1431492 | C | T | 0.157 | -0.065 | 0.015 | 7.36E-06 | -0.006 | 0.026 | 0.83 | |
| *Anaerotruncus* | rs17734739 | T | C | 0.140 | 0.066 | 0.015 | 7.43E-06 | -0.016 | 0.028 | 0.56 | |
| *Anaerotruncus* | rs7155595 | C | A | 0.303 | 0.054 | 0.012 | 7.55E-06 | -0.001 | 0.021 | 0.95 | |
| *Anaerotruncus* | rs34449434 | A | C | 0.392 | -0.050 | 0.011 | 9.85E-06 | 0.003 | 0.020 | 0.88 | |
| *Bacteroides* | rs6795673 | C | T | 0.478 | 0.054 | 0.011 | 3.38E-07 | 0.024 | 0.019 | 0.20 | |
| *Bacteroides* | rs11585893 | A | G | 0.246 | -0.074 | 0.015 | 1.80E-06 | 0.025 | 0.022 | 0.27 | |
| *Bacteroides* | rs17619981 | T | G | 0.135 | 0.088 | 0.019 | 2.69E-06 | 0.016 | 0.028 | 0.56 | |
| *Bacteroides* | rs2023437 | T | C | 0.124 | -0.078 | 0.017 | 5.02E-06 | 0.021 | 0.029 | 0.47 | |
| *Bacteroides* | rs66710942 | C | T | 0.524 | 0.049 | 0.011 | 5.86E-06 | -0.022 | 0.019 | 0.26 | |
| *Bacteroides* | rs1340391 | T | C | 0.135 | -0.059 | 0.013 | 6.73E-06 | -0.008 | 0.028 | 0.79 | |
| *Bacteroides* | rs13207588 | A | G | 0.196 | -0.059 | 0.013 | 7.49E-06 | -0.021 | 0.024 | 0.38 | |
| *Barnesiella* | rs2276875 | A | G | 0.262 | -0.070 | 0.014 | 4.65E-07 | -0.008 | 0.022 | 0.73 | |
| *Barnesiella* | rs2428166 | G | A | 0.014 | -0.166 | 0.034 | 8.51E-07 | -0.015 | 0.082 | 0.86 | |
| *Barnesiella* | rs13242616 | T | C | 0.323 | -0.058 | 0.012 | 2.29E-06 | -0.014 | 0.021 | 0.50 | |
| *Barnesiella* | rs35177866 | A | G | 0.077 | 0.092 | 0.019 | 2.95E-06 | -0.006 | 0.037 | 0.88 | |
| *Barnesiella* | rs199035 | G | A | 0.555 | 0.056 | 0.012 | 3.00E-06 | -0.024 | 0.019 | 0.22 | |
| *Barnesiella* | rs77455852 | T | G | 0.151 | -0.089 | 0.020 | 3.16E-06 | -0.015 | 0.027 | 0.57 | |
| *Barnesiella* | rs79795328 | A | G | 0.139 | -0.082 | 0.018 | 4.23E-06 | 0.038 | 0.028 | 0.17 | |
| *Barnesiella* | rs62251337 | A | G | 0.141 | -0.069 | 0.015 | 4.24E-06 | -0.001 | 0.027 | 0.98 | |
| *Barnesiella* | rs12909713 | C | T | 0.489 | -0.055 | 0.012 | 4.95E-06 | 0.050 | 0.019 | 0.01 | |
| *Barnesiella* | rs72684847 | T | C | 0.076 | -0.114 | 0.025 | 6.76E-06 | -0.035 | 0.036 | 0.34 | |
| *Barnesiella* | rs76181748 | C | T | 0.230 | -0.078 | 0.017 | 6.78E-06 | 0.025 | 0.023 | 0.28 | |
| *Barnesiella* | rs11155559 | T | C | 0.092 | 0.096 | 0.021 | 8.92E-06 | -0.010 | 0.033 | 0.77 | |
| *Bifidobacterium* | rs182549 | C | T | 0.401 | 0.120 | 0.013 | 1.28E-20 | -0.060 | 0.020 | 0.00 | |
| *Bifidobacterium* | rs7322849 | T | C | 0.091 | 0.112 | 0.020 | 1.08E-08 | -0.011 | 0.033 | 0.73 | |
| *Bifidobacterium* | rs62181700 | G | A | 0.247 | -0.062 | 0.013 | 2.17E-06 | -0.004 | 0.022 | 0.87 | |
| *Bifidobacterium* | rs56108664 | T | C | 0.130 | 0.073 | 0.016 | 2.44E-06 | 0.011 | 0.028 | 0.71 | |
| *Bifidobacterium* | rs857444 | C | T | 0.369 | 0.056 | 0.012 | 3.57E-06 | -0.044 | 0.020 | 0.03 | |
| *Bifidobacterium* | rs75344046 | C | T | 0.047 | 0.232 | 0.051 | 4.86E-06 | -0.060 | 0.045 | 0.18 | |
| *Bifidobacterium* | rs540489 | T | G | 0.176 | -0.064 | 0.014 | 5.19E-06 | -0.014 | 0.025 | 0.59 | |
| *Bifidobacterium* | rs55888705 | A | G | 0.280 | 0.055 | 0.012 | 6.67E-06 | 0.005 | 0.021 | 0.82 | |
| *Bifidobacterium* | rs2686790 | T | C | 0.148 | 0.071 | 0.016 | 7.50E-06 | -0.016 | 0.027 | 0.55 | |
| *Bifidobacterium* | rs11208411 | T | C | 0.840 | 0.071 | 0.016 | 7.57E-06 | -0.016 | 0.026 | 0.55 | |
| *Bifidobacterium* | rs12022129 | G | A | 0.275 | 0.062 | 0.014 | 8.00E-06 | -0.020 | 0.021 | 0.35 | |
| *Bifidobacterium* | rs2491158 | G | A | 0.133 | 0.071 | 0.016 | 8.05E-06 | 0.004 | 0.028 | 0.89 | |
| *Bifidobacterium* | rs5746486 | T | C | 0.388 | -0.054 | 0.012 | 9.00E-06 | 0.040 | 0.020 | 0.04 | |
| *Bilophila* | rs1571225 | C | T | 0.165 | 0.083 | 0.017 | 1.12E-06 | 0.047 | 0.026 | 0.07 | |
| *Bilophila* | rs7802841 | C | A | 0.303 | 0.067 | 0.014 | 1.77E-06 | -0.017 | 0.021 | 0.40 | |
| *Bilophila* | rs3827020 | C | T | 0.196 | 0.077 | 0.016 | 1.79E-06 | -0.014 | 0.024 | 0.57 | |
| *Bilophila* | rs6793291 | C | A | 0.053 | 0.113 | 0.024 | 3.11E-06 | -0.055 | 0.043 | 0.20 | |
| *Bilophila* | rs1241171 | G | A | 0.144 | -0.069 | 0.015 | 4.24E-06 | 0.009 | 0.027 | 0.73 | |
| *Bilophila* | rs542415 | T | C | 0.389 | -0.061 | 0.013 | 4.71E-06 | -0.005 | 0.020 | 0.80 | |
| *Bilophila* | rs72676854 | T | C | 0.054 | 0.123 | 0.027 | 5.62E-06 | -0.009 | 0.043 | 0.84 | |
| *Bilophila* | rs2728491 | G | T | 0.239 | -0.063 | 0.014 | 6.33E-06 | -0.001 | 0.022 | 0.98 | |
| *Bilophila* | rs4798126 | G | A | 0.197 | 0.073 | 0.017 | 7.15E-06 | 0.016 | 0.024 | 0.49 | |
| *Bilophila* | rs11069458 | T | C | 0.186 | -0.068 | 0.016 | 7.72E-06 | 0.018 | 0.025 | 0.46 | |
| *Bilophila* | rs60178956 | G | A | 0.227 | -0.062 | 0.014 | 8.06E-06 | 0.008 | 0.023 | 0.73 | |
| *Bilophila* | rs9899990 | A | G | 0.084 | -0.103 | 0.023 | 9.07E-06 | -0.009 | 0.035 | 0.80 | |
| *Bilophila* | rs1969927 | G | A | 0.336 | 0.056 | 0.013 | 9.07E-06 | 0.038 | 0.020 | 0.06 | |
| *Blautia* | rs67794373 | C | T | 0.223 | 0.060 | 0.012 | 1.00E-06 | 0.027 | 0.023 | 0.23 | |
| *Blautia* | rs11149971 | C | T | 0.055 | 0.118 | 0.023 | 1.04E-06 | 0.027 | 0.042 | 0.52 | |
| *Blautia* | rs12453000 | C | T | 0.137 | 0.063 | 0.013 | 1.26E-06 | -0.014 | 0.028 | 0.62 | |
| *Blautia* | rs72973581 | A | G | 0.059 | 0.125 | 0.027 | 1.74E-06 | -0.020 | 0.041 | 0.63 | |
| *Blautia* | rs7860714 | A | G | 0.368 | -0.050 | 0.011 | 4.09E-06 | 0.029 | 0.020 | 0.15 | |
| *Blautia* | rs682885 | A | G | 0.326 | -0.049 | 0.011 | 4.49E-06 | -0.012 | 0.020 | 0.54 | |
| *Blautia* | rs4926264 | T | C | 0.106 | 0.083 | 0.018 | 5.10E-06 | 0.004 | 0.031 | 0.90 | |
| *Blautia* | rs115043014 | G | A | 0.017 | -0.207 | 0.044 | 5.19E-06 | 0.000 | 0.076 | 1.00 | |
| *Blautia* | rs3005511 | A | G | 0.300 | 0.050 | 0.011 | 6.19E-06 | 0.001 | 0.021 | 0.96 | |
| *Blautia* | rs2788271 | T | G | 0.172 | -0.058 | 0.013 | 7.16E-06 | 0.005 | 0.025 | 0.84 | |
| *Blautia* | rs16892041 | T | C | 0.212 | -0.062 | 0.014 | 8.82E-06 | 0.000 | 0.023 | 0.99 | |
| *Blautia* | rs117001700 | T | C | 0.015 | 0.196 | 0.044 | 8.84E-06 | -0.038 | 0.082 | 0.64 | |
| *Butyricicoccus* | rs56221232 | T | C | 0.101 | 0.083 | 0.017 | 7.62E-07 | -0.011 | 0.032 | 0.72 | |
| *Butyricicoccus* | rs2017189 | G | T | 0.471 | -0.051 | 0.011 | 3.87E-06 | -0.010 | 0.019 | 0.59 | |
| *Butyricicoccus* | rs7322368 | T | C | 0.091 | 0.082 | 0.018 | 5.52E-06 | 0.010 | 0.033 | 0.76 | |
| *Butyricicoccus* | rs12585793 | T | C | 0.029 | -0.262 | 0.056 | 5.79E-06 | 0.029 | 0.058 | 0.62 | |
| *Butyricicoccus* | rs62478070 | T | G | 0.026 | 0.224 | 0.049 | 5.94E-06 | 0.044 | 0.061 | 0.47 | |
| *Butyricicoccus* | rs4962426 | G | T | 0.195 | 0.061 | 0.014 | 7.38E-06 | 0.024 | 0.024 | 0.31 | |
| *Butyricicoccus* | rs10084203 | A | G | 0.130 | 0.055 | 0.012 | 8.59E-06 | -0.046 | 0.029 | 0.11 | |
| *Butyricicoccus* | rs12034718 | A | G | 0.223 | 0.070 | 0.016 | 9.58E-06 | 0.010 | 0.023 | 0.68 | |
| *Butyricimonas* | rs113054641 | G | A | 0.045 | -0.145 | 0.027 | 1.74E-07 | 0.024 | 0.046 | 0.60 | |
| *Butyricimonas* | rs62390301 | T | C | 0.200 | -0.087 | 0.017 | 7.42E-07 | 0.038 | 0.024 | 0.11 | |
| *Butyricimonas* | rs7083431 | A | C | 0.274 | 0.070 | 0.014 | 8.85E-07 | 0.015 | 0.022 | 0.47 | |
| *Butyricimonas* | rs62130338 | G | A | 0.344 | -0.073 | 0.016 | 3.90E-06 | -0.025 | 0.020 | 0.21 | |
| *Butyricimonas* | rs78453362 | A | G | 0.026 | -0.149 | 0.033 | 4.06E-06 | 0.027 | 0.061 | 0.66 | |
| *Butyricimonas* | rs9657374 | C | T | 0.294 | 0.068 | 0.015 | 4.50E-06 | 0.019 | 0.021 | 0.36 | |
| *Butyricimonas* | rs1862649 | G | A | 0.069 | 0.113 | 0.025 | 4.76E-06 | 0.029 | 0.038 | 0.44 | |
| *Butyricimonas* | rs71428626 | G | T | 0.032 | -0.133 | 0.029 | 4.80E-06 | -0.072 | 0.055 | 0.19 | |
| *Butyricimonas* | rs12458763 | A | C | 0.045 | 0.122 | 0.027 | 6.37E-06 | 0.034 | 0.046 | 0.46 | |
| *Butyricimonas* | rs11228830 | A | G | 0.087 | 0.135 | 0.030 | 6.55E-06 | 0.036 | 0.034 | 0.29 | |
| *Butyricimonas* | rs12304031 | G | A | 0.124 | -0.086 | 0.020 | 6.70E-06 | -0.005 | 0.029 | 0.86 | |
| *Butyricimonas* | rs2114713 | G | T | 0.448 | 0.063 | 0.014 | 6.88E-06 | -0.013 | 0.019 | 0.48 | |
| *Butyricimonas* | rs72814525 | A | G | 0.244 | 0.066 | 0.015 | 8.25E-06 | -0.017 | 0.022 | 0.44 | |
| *Butyrivibrio* | rs7752361 | A | G | 0.483 | -0.119 | 0.024 | 7.69E-07 | 0.027 | 0.019 | 0.16 | |
| *Butyrivibrio* | rs72723662 | C | T | 0.142 | 0.224 | 0.045 | 7.86E-07 | -0.043 | 0.028 | 0.13 | |
| *Butyrivibrio* | rs16941336 | C | T | 0.231 | 0.127 | 0.027 | 1.53E-06 | -0.008 | 0.023 | 0.71 | |
| *Butyrivibrio* | rs4537857 | T | C | 0.334 | -0.125 | 0.026 | 1.80E-06 | -0.033 | 0.020 | 0.10 | |
| *Butyrivibrio* | rs11761679 | T | C | 0.140 | 0.155 | 0.032 | 2.20E-06 | 0.007 | 0.028 | 0.81 | |
| *Butyrivibrio* | rs74622183 | A | G | 0.090 | -0.201 | 0.043 | 2.46E-06 | -0.042 | 0.033 | 0.21 | |
| *Butyrivibrio* | rs7763512 | G | A | 0.424 | 0.120 | 0.025 | 3.11E-06 | -0.027 | 0.019 | 0.16 | |
| *Butyrivibrio* | rs17163238 | G | A | 0.192 | 0.141 | 0.031 | 5.51E-06 | -0.027 | 0.024 | 0.27 | |
| *Butyrivibrio* | rs9349693 | A | G | 0.291 | 0.118 | 0.026 | 5.55E-06 | 0.002 | 0.021 | 0.92 | |
| *Butyrivibrio* | rs486484 | A | G | 0.425 | -0.108 | 0.024 | 6.61E-06 | 0.038 | 0.019 | 0.05 | |
| *Butyrivibrio* | rs77356209 | T | C | 0.046 | 0.217 | 0.048 | 6.66E-06 | 0.099 | 0.046 | 0.03 | |
| *Butyrivibrio* | rs1007475 | G | T | 0.277 | 0.118 | 0.026 | 7.92E-06 | -0.006 | 0.021 | 0.79 | |
| *Butyrivibrio* | rs4928024 | A | G | 0.186 | -0.175 | 0.039 | 8.19E-06 | -0.013 | 0.025 | 0.60 | |
| *Butyrivibrio* | rs16934069 | T | C | 0.178 | -0.134 | 0.030 | 8.86E-06 | 0.065 | 0.025 | 0.01 | |
| *Candidatus Soleaferrea* | rs4294381 | T | C | 0.157 | 0.112 | 0.023 | 1.37E-06 | 0.000 | 0.026 | 0.99 | |
| *Candidatus Soleaferrea* | rs10090365 | A | G | 0.479 | -0.083 | 0.018 | 4.17E-06 | 0.017 | 0.019 | 0.37 | |
| *Candidatus Soleaferrea* | rs36155147 | C | T | 0.341 | 0.105 | 0.024 | 5.41E-06 | -0.028 | 0.021 | 0.18 | |
| *Candidatus Soleaferrea* | rs10809135 | T | C | 0.444 | 0.083 | 0.018 | 5.47E-06 | -0.011 | 0.019 | 0.58 | |
| *Candidatus Soleaferrea* | rs4678258 | T | C | 0.243 | 0.099 | 0.022 | 5.53E-06 | -0.018 | 0.022 | 0.43 | |
| *Candidatus Soleaferrea* | rs6494306 | A | G | 0.325 | -0.097 | 0.021 | 5.80E-06 | 0.009 | 0.020 | 0.66 | |
| *Candidatus Soleaferrea* | rs9973954 | A | G | 0.358 | 0.089 | 0.020 | 5.95E-06 | -0.003 | 0.020 | 0.90 | |
| *Candidatus Soleaferrea* | rs6489992 | A | G | 0.371 | -0.084 | 0.019 | 7.89E-06 | -0.027 | 0.020 | 0.18 | |
| *Candidatus Soleaferrea* | rs7400877 | T | C | 0.213 | -0.095 | 0.021 | 9.29E-06 | 0.006 | 0.023 | 0.79 | |
| *Catenibacterium* | rs12404911 | C | T | 0.185 | 0.141 | 0.030 | 2.80E-06 | 0.040 | 0.025 | 0.11 | |
| *Catenibacterium* | rs212393 | G | A | 0.208 | -0.135 | 0.029 | 3.62E-06 | 0.012 | 0.024 | 0.63 | |
| *Catenibacterium* | rs73128290 | A | G | 0.300 | 0.130 | 0.028 | 4.29E-06 | -0.014 | 0.021 | 0.50 | |
| *Catenibacterium* | rs7742829 | C | T | 0.436 | 0.114 | 0.025 | 5.61E-06 | 0.018 | 0.019 | 0.34 | |
| *Christensenellaceae (R7 group)* | rs62467127 | C | T | 0.025 | 0.114 | 0.025 | 3.25E-06 | 0.022 | 0.062 | 0.73 | |
| *Christensenellaceae (R7 group)* | rs17081797 | A | G | 0.065 | -0.090 | 0.020 | 3.34E-06 | -0.014 | 0.039 | 0.71 | |
| *Christensenellaceae (R7 group)* | rs892686 | A | G | 0.472 | 0.051 | 0.011 | 3.97E-06 | 0.029 | 0.019 | 0.13 | |
| *Christensenellaceae (R7 group)* | rs78521377 | C | T | 0.030 | 0.125 | 0.027 | 5.61E-06 | -0.064 | 0.056 | 0.26 | |
| *Christensenellaceae (R7 group)* | rs62132810 | A | G | 0.145 | -0.083 | 0.018 | 5.67E-06 | -0.003 | 0.028 | 0.90 | |
| *Christensenellaceae (R7 group)* | rs10461257 | A | G | 0.352 | -0.055 | 0.012 | 6.51E-06 | 0.025 | 0.020 | 0.22 | |
| *Christensenellaceae (R7 group)* | rs73952017 | C | T | 0.103 | -0.086 | 0.019 | 8.46E-06 | -0.023 | 0.032 | 0.46 | |
| *Christensenellaceae (R7 group)* | rs62190261 | A | C | 0.088 | 0.096 | 0.021 | 8.74E-06 | 0.005 | 0.034 | 0.89 | |
| *Clostridium (innocuum group)* | rs6890185 | T | C | 0.323 | 0.113 | 0.023 | 1.12E-06 | -0.003 | 0.020 | 0.88 | |
| *Clostridium (innocuum group)* | rs1942371 | G | A | 0.123 | -0.158 | 0.034 | 4.06E-06 | -0.033 | 0.029 | 0.26 | |
| *Clostridium (innocuum group)* | rs61267978 | T | C | 0.122 | 0.147 | 0.032 | 5.59E-06 | 0.000 | 0.029 | 1.00 | |
| *Clostridium (innocuum group)* | rs4869133 | G | A | 0.177 | -0.181 | 0.041 | 7.24E-06 | -0.021 | 0.025 | 0.40 | |
| *Clostridium (innocuum group)* | rs6577484 | G | A | 0.104 | 0.160 | 0.036 | 8.41E-06 | 0.005 | 0.032 | 0.88 | |
| *Clostridium (innocuum group)* | rs40656 | C | T | 0.206 | 0.143 | 0.031 | 8.62E-06 | -0.004 | 0.024 | 0.85 | |
| *Clostridium (innocuum group)* | rs10506058 | A | G | 0.423 | 0.100 | 0.022 | 8.92E-06 | -0.032 | 0.019 | 0.10 | |
| *Clostridium sensustricto 1* | rs550843 | T | C | 0.282 | -0.078 | 0.017 | 2.05E-06 | 0.007 | 0.021 | 0.75 | |
| *Clostridium sensustricto 1* | rs2795528 | G | A | 0.056 | -0.184 | 0.039 | 2.72E-06 | -0.016 | 0.041 | 0.70 | |
| *Clostridium sensustricto 1* | rs2817172 | C | T | 0.396 | 0.058 | 0.012 | 2.77E-06 | -0.024 | 0.020 | 0.22 | |
| *Clostridium sensustricto 1* | rs115807074 | A | G | 0.011 | -0.227 | 0.049 | 4.32E-06 | -0.119 | 0.095 | 0.21 | |
| *Clostridium sensustricto 1* | rs116847295 | C | T | 0.127 | 0.110 | 0.025 | 4.58E-06 | 0.001 | 0.029 | 0.97 | |
| *Clostridium sensustricto 1* | rs12341505 | G | A | 0.093 | 0.081 | 0.018 | 4.82E-06 | -0.018 | 0.033 | 0.58 | |
| *Clostridium sensustricto 1* | rs11264403 | G | A | 0.076 | -0.139 | 0.033 | 7.76E-06 | -0.036 | 0.036 | 0.33 | |
| *Collinsella* | rs9541268 | C | A | 0.090 | 0.096 | 0.020 | 8.79E-07 | -0.065 | 0.033 | 0.05 | |
| *Collinsella* | rs73052258 | G | A | 0.076 | 0.093 | 0.020 | 1.72E-06 | -0.033 | 0.036 | 0.36 | |
| *Collinsella* | rs2103510 | G | A | 0.116 | 0.079 | 0.017 | 2.42E-06 | -0.011 | 0.030 | 0.72 | |
| *Collinsella* | rs75672793 | A | G | 0.046 | -0.109 | 0.024 | 6.14E-06 | -0.024 | 0.046 | 0.60 | |
| *Collinsella* | rs10890671 | T | C | 0.563 | -0.054 | 0.012 | 6.52E-06 | 0.027 | 0.019 | 0.16 | |
| *Collinsella* | rs1496626 | T | C | 0.134 | -0.072 | 0.016 | 6.78E-06 | -0.005 | 0.028 | 0.86 | |
| *Collinsella* | rs62448871 | C | A | 0.537 | -0.054 | 0.012 | 6.78E-06 | 0.038 | 0.019 | 0.05 | |
| *Collinsella* | rs73068166 | G | T | 0.068 | -0.103 | 0.024 | 8.58E-06 | -0.030 | 0.038 | 0.42 | |
| *Collinsella* | rs11597285 | G | T | 0.398 | -0.054 | 0.012 | 9.38E-06 | 0.018 | 0.020 | 0.36 | |
| *Coprobacter* | rs305411 | A | G | 0.103 | 0.129 | 0.026 | 1.01E-06 | -0.064 | 0.032 | 0.05 | |
| *Coprobacter* | rs213863 | C | T | 0.360 | -0.089 | 0.019 | 2.35E-06 | -0.014 | 0.020 | 0.47 | |
| *Coprobacter* | rs3828477 | G | T | 0.345 | -0.091 | 0.020 | 2.89E-06 | 0.052 | 0.020 | 0.01 | |
| *Coprobacter* | rs143662916 | C | T | 0.030 | 0.253 | 0.054 | 3.07E-06 | -0.017 | 0.056 | 0.76 | |
| *Coprobacter* | rs72821405 | T | C | 0.090 | -0.147 | 0.032 | 4.76E-06 | -0.023 | 0.034 | 0.49 | |
| *Coprobacter* | rs11532348 | C | T | 0.149 | -0.104 | 0.023 | 5.71E-06 | 0.008 | 0.027 | 0.78 | |
| *Coprobacter* | rs74919520 | G | A | 0.107 | 0.126 | 0.028 | 5.76E-06 | -0.003 | 0.031 | 0.91 | |
| *Coprobacter* | rs12684609 | T | C | 0.197 | 0.101 | 0.022 | 6.10E-06 | 0.002 | 0.024 | 0.92 | |
| *Coprobacter* | rs189356 | G | A | 0.571 | 0.078 | 0.017 | 6.26E-06 | 0.055 | 0.019 | 0.00 | |
| *Coprobacter* | rs12996055 | A | C | 0.269 | 0.092 | 0.021 | 8.08E-06 | 0.025 | 0.022 | 0.26 | |
| *Coprococcus 1* | rs4277593 | G | A | 0.411 | -0.059 | 0.011 | 1.14E-07 | 0.020 | 0.019 | 0.31 | |
| *Coprococcus 1* | rs74101919 | T | C | 0.108 | -0.072 | 0.014 | 1.03E-06 | -0.038 | 0.031 | 0.22 | |
| *Coprococcus 1* | rs56405618 | A | G | 0.116 | -0.090 | 0.019 | 1.57E-06 | 0.022 | 0.030 | 0.46 | |
| *Coprococcus 1* | rs1010560 | C | A | 0.267 | 0.058 | 0.012 | 1.96E-06 | 0.006 | 0.022 | 0.79 | |
| *Coprococcus 1* | rs73031725 | T | C | 0.032 | 0.168 | 0.036 | 1.98E-06 | 0.022 | 0.055 | 0.69 | |
| *Coprococcus 1* | rs1576241 | A | G | 0.382 | -0.051 | 0.011 | 3.33E-06 | 0.023 | 0.020 | 0.24 | |
| *Coprococcus 1* | rs12794898 | G | T | 0.129 | 0.090 | 0.020 | 4.92E-06 | 0.020 | 0.029 | 0.47 | |
| *Coprococcus 1* | rs2907920 | A | G | 0.284 | 0.056 | 0.013 | 7.65E-06 | -0.010 | 0.021 | 0.62 | |
| *Coprococcus 1* | rs73167075 | T | C | 0.211 | 0.057 | 0.013 | 8.57E-06 | -0.008 | 0.023 | 0.73 | |
| *Coprococcus 1* | rs946513 | C | T | 0.047 | 0.206 | 0.046 | 8.62E-06 | 0.041 | 0.046 | 0.37 | |
| *Coprococcus 1* | rs1519491 | T | C | 0.396 | 0.050 | 0.011 | 8.95E-06 | -0.011 | 0.020 | 0.59 | |
| *Coprococcus 2* | rs6677933 | C | T | 0.148 | -0.080 | 0.016 | 1.19E-06 | -0.005 | 0.027 | 0.84 | |
| *Coprococcus 2* | rs72680320 | T | C | 0.335 | -0.065 | 0.014 | 2.27E-06 | 0.023 | 0.020 | 0.25 | |
| *Coprococcus 2* | rs2482516 | C | T | 0.226 | 0.075 | 0.016 | 4.72E-06 | -0.037 | 0.023 | 0.11 | |
| *Coprococcus 2* | rs9426473 | A | G | 0.259 | 0.073 | 0.016 | 6.31E-06 | -0.004 | 0.022 | 0.86 | |
| *Coprococcus 2* | rs61823518 | A | C | 0.106 | -0.096 | 0.022 | 6.68E-06 | 0.033 | 0.031 | 0.29 | |
| *Coprococcus 2* | rs10070053 | A | G | 0.414 | 0.059 | 0.014 | 7.65E-06 | -0.003 | 0.019 | 0.89 | |
| *Coprococcus 2* | rs35890118 | A | G | 0.254 | -0.067 | 0.015 | 8.26E-06 | 0.019 | 0.022 | 0.39 | |
| *Coprococcus 2* | rs12634070 | T | C | 0.257 | 0.074 | 0.016 | 9.95E-06 | 0.010 | 0.022 | 0.64 | |
| *Coprococcus3* | rs8100692 | T | C | 0.559 | 0.058 | 0.011 | 4.16E-07 | -0.020 | 0.019 | 0.30 | |
| *Coprococcus3* | rs178271 | T | C | 0.017 | 0.145 | 0.029 | 7.81E-07 | 0.058 | 0.077 | 0.45 | |
| *Coprococcus3* | rs13394391 | C | T | 0.155 | -0.071 | 0.015 | 2.20E-06 | 0.010 | 0.027 | 0.70 | |
| *Coprococcus3* | rs7521171 | G | A | 0.310 | -0.060 | 0.013 | 4.32E-06 | -0.048 | 0.021 | 0.02 | |
| *Coprococcus3* | rs11080344 | C | T | 0.445 | 0.052 | 0.011 | 4.79E-06 | -0.005 | 0.019 | 0.80 | |
| *Coprococcus3* | rs4575475 | G | A | 0.230 | 0.062 | 0.014 | 7.04E-06 | -0.034 | 0.023 | 0.14 | |
| *Coprococcus3* | rs13247359 | G | A | 0.446 | 0.051 | 0.011 | 7.33E-06 | 0.014 | 0.019 | 0.48 | |
| *Coprococcus3* | rs10810043 | A | G | 0.336 | 0.052 | 0.012 | 9.27E-06 | -0.001 | 0.020 | 0.96 | |
| *Coprococcus3* | rs11077359 | T | C | 0.171 | -0.065 | 0.015 | 9.64E-06 | 0.034 | 0.026 | 0.19 | |
| *Defluviitaleaceae (UCG011)* | rs72731813 | C | T | 0.048 | -0.147 | 0.029 | 4.33E-07 | 0.026 | 0.045 | 0.56 | |
| *Defluviitaleaceae (UCG011)* | rs4677103 | A | G | 0.180 | 0.098 | 0.020 | 9.60E-07 | -0.003 | 0.025 | 0.91 | |
| *Defluviitaleaceae (UCG011)* | rs112893842 | T | C | 0.090 | 0.114 | 0.023 | 1.45E-06 | -0.029 | 0.034 | 0.40 | |
| *Defluviitaleaceae (UCG011)* | rs1582238 | T | C | 0.358 | 0.081 | 0.017 | 1.57E-06 | 0.025 | 0.020 | 0.21 | |
| *Defluviitaleaceae (UCG011)* | rs55658617 | T | C | 0.036 | 0.174 | 0.036 | 2.15E-06 | -0.007 | 0.052 | 0.90 | |
| *Defluviitaleaceae (UCG011)* | rs9608282 | T | G | 0.033 | 0.143 | 0.030 | 2.52E-06 | -0.006 | 0.053 | 0.91 | |
| *Defluviitaleaceae (UCG011)* | rs9725395 | A | G | 0.114 | -0.138 | 0.030 | 3.52E-06 | 0.028 | 0.030 | 0.35 | |
| *Defluviitaleaceae (UCG011)* | rs4344384 | G | T | 0.496 | 0.072 | 0.016 | 4.83E-06 | -0.002 | 0.019 | 0.93 | |
| *Defluviitaleaceae (UCG011)* | rs2892880 | G | A | 0.250 | 0.082 | 0.018 | 6.83E-06 | -0.016 | 0.022 | 0.48 | |
| *Desulfovibrio* | rs16863365 | A | G | 0.047 | 0.109 | 0.023 | 1.79E-06 | 0.019 | 0.046 | 0.68 | |
| *Desulfovibrio* | rs2853179 | C | T | 0.230 | 0.081 | 0.017 | 2.42E-06 | -0.006 | 0.023 | 0.79 | |
| *Desulfovibrio* | rs13066142 | G | A | 0.091 | 0.119 | 0.025 | 3.79E-06 | 0.003 | 0.033 | 0.94 | |
| *Desulfovibrio* | rs6580353 | T | C | 0.204 | 0.077 | 0.017 | 4.94E-06 | 0.023 | 0.024 | 0.34 | |
| *Desulfovibrio* | rs4797774 | G | A | 0.043 | 0.213 | 0.047 | 5.64E-06 | -0.028 | 0.048 | 0.56 | |
| *Desulfovibrio* | rs12031543 | T | C | 0.138 | -0.127 | 0.028 | 6.55E-06 | 0.001 | 0.028 | 0.97 | |
| *Desulfovibrio* | rs2590913 | G | A | 0.052 | 0.154 | 0.034 | 6.65E-06 | 0.096 | 0.044 | 0.03 | |
| *Desulfovibrio* | rs72647089 | T | G | 0.081 | -0.107 | 0.024 | 8.30E-06 | -0.012 | 0.035 | 0.73 | |
| *Desulfovibrio* | rs2032031 | A | G | 0.509 | -0.065 | 0.015 | 9.14E-06 | -0.023 | 0.019 | 0.24 | |
| *Desulfovibrio* | rs7729080 | C | A | 0.293 | -0.070 | 0.016 | 9.96E-06 | -0.021 | 0.021 | 0.32 | |
| *Dialister* | rs11166701 | G | A | 0.509 | -0.066 | 0.013 | 5.51E-07 | -0.015 | 0.019 | 0.43 | |
| *Dialister* | rs4753063 | G | A | 0.446 | -0.060 | 0.013 | 4.86E-06 | 0.000 | 0.019 | 0.99 | |
| *Dialister* | rs4747450 | C | A | 0.224 | 0.067 | 0.015 | 5.84E-06 | 0.023 | 0.023 | 0.32 | |
| *Dialister* | rs11071887 | T | C | 0.324 | 0.066 | 0.015 | 5.91E-06 | 0.004 | 0.020 | 0.85 | |
| *Dialister* | rs2435610 | A | C | 0.253 | 0.065 | 0.014 | 5.93E-06 | -0.007 | 0.022 | 0.76 | |
| *Dialister* | rs10938938 | G | A | 0.157 | -0.077 | 0.017 | 7.37E-06 | -0.013 | 0.026 | 0.61 | |
| *Dialister* | rs10138457 | T | C | 0.093 | -0.113 | 0.026 | 7.88E-06 | -0.057 | 0.033 | 0.08 | |
| *Dialister* | rs2314294 | T | C | 0.136 | 0.087 | 0.019 | 8.08E-06 | -0.022 | 0.028 | 0.44 | |
| *Dialister* | rs76680460 | G | A | 0.043 | -0.161 | 0.036 | 8.19E-06 | -0.036 | 0.048 | 0.45 | |
| *Dialister* | rs75416973 | A | G | 0.222 | 0.073 | 0.016 | 9.46E-06 | -0.053 | 0.023 | 0.02 | |
| *Dialister* | rs764177 | C | A | 0.367 | -0.060 | 0.014 | 9.61E-06 | 0.035 | 0.020 | 0.08 | |
| *Dorea* | rs62503162 | A | G | 0.044 | -0.097 | 0.019 | 7.47E-07 | -0.023 | 0.047 | 0.63 | |
| *Dorea* | rs13279148 | G | A | 0.110 | 0.072 | 0.015 | 2.25E-06 | 0.006 | 0.031 | 0.84 | |
| *Dorea* | rs73729431 | C | T | 0.021 | -0.137 | 0.030 | 3.17E-06 | 0.042 | 0.067 | 0.54 | |
| *Dorea* | rs4793307 | C | T | 0.240 | 0.057 | 0.012 | 4.01E-06 | 0.021 | 0.023 | 0.35 | |
| *Dorea* | rs1899291 | C | T | 0.156 | 0.070 | 0.015 | 4.57E-06 | 0.044 | 0.026 | 0.09 | |
| *Dorea* | rs3005511 | A | G | 0.300 | 0.052 | 0.011 | 5.29E-06 | 0.001 | 0.021 | 0.96 | |
| *Dorea* | rs11150408 | T | G | 0.480 | 0.049 | 0.011 | 7.06E-06 | 0.017 | 0.019 | 0.39 | |
| *Dorea* | rs3752849 | G | A | 0.050 | 0.164 | 0.037 | 7.68E-06 | -0.040 | 0.044 | 0.37 | |
| *Dorea* | rs345219 | T | G | 0.471 | -0.050 | 0.011 | 8.80E-06 | 0.033 | 0.019 | 0.09 | |
| *Dorea* | rs12537781 | T | C | 0.238 | -0.056 | 0.013 | 9.15E-06 | -0.030 | 0.022 | 0.19 | |
| *Eggerthella* | rs2240838 | A | G | 0.540 | 0.098 | 0.020 | 7.36E-07 | 0.014 | 0.019 | 0.46 | |
| *Eggerthella* | rs112205261 | T | C | 0.081 | -0.189 | 0.040 | 3.35E-06 | 0.035 | 0.035 | 0.32 | |
| *Eggerthella* | rs2223081 | G | A | 0.284 | 0.103 | 0.022 | 3.89E-06 | 0.034 | 0.021 | 0.11 | |
| *Eggerthella* | rs3851328 | T | G | 0.222 | -0.108 | 0.024 | 4.18E-06 | -0.022 | 0.023 | 0.34 | |
| *Eggerthella* | rs76663501 | C | T | 0.052 | 0.175 | 0.038 | 4.83E-06 | 0.026 | 0.043 | 0.54 | |
| *Eggerthella* | rs1784446 | G | A | 0.488 | 0.091 | 0.020 | 5.23E-06 | -0.012 | 0.019 | 0.54 | |
| *Eggerthella* | rs4985746 | G | A | 0.104 | 0.111 | 0.025 | 5.71E-06 | -0.019 | 0.032 | 0.55 | |
| *Eggerthella* | rs13070736 | A | C | 0.165 | -0.121 | 0.027 | 7.62E-06 | -0.001 | 0.026 | 0.96 | |
| *Eggerthella* | rs6430926 | C | T | 0.469 | 0.088 | 0.020 | 8.37E-06 | -0.020 | 0.019 | 0.31 | |
| *Eggerthella* | rs67490567 | T | C | 0.257 | 0.108 | 0.025 | 8.94E-06 | -0.041 | 0.022 | 0.06 | |
| *Eisenbergiella* | rs2683098 | C | T | 0.216 | 0.107 | 0.023 | 2.24E-06 | -0.019 | 0.023 | 0.42 | |
| *Eisenbergiella* | rs3812426 | G | A | 0.155 | 0.106 | 0.022 | 2.72E-06 | 0.012 | 0.026 | 0.64 | |
| *Eisenbergiella* | rs1508033 | A | C | 0.315 | 0.092 | 0.020 | 3.23E-06 | 0.003 | 0.021 | 0.90 | |
| *Eisenbergiella* | rs4462860 | G | A | 0.396 | 0.094 | 0.020 | 4.16E-06 | -0.004 | 0.020 | 0.85 | |
| *Eisenbergiella* | rs11027642 | C | T | 0.142 | 0.129 | 0.028 | 4.92E-06 | -0.005 | 0.027 | 0.85 | |
| *Eisenbergiella* | rs1553971 | T | G | 0.226 | 0.121 | 0.026 | 5.27E-06 | -0.027 | 0.023 | 0.25 | |
| *Eisenbergiella* | rs11079158 | T | C | 0.217 | 0.101 | 0.023 | 7.35E-06 | 0.010 | 0.023 | 0.66 | |
| *Eisenbergiella* | rs13258851 | A | G | 0.141 | 0.137 | 0.030 | 7.75E-06 | 0.018 | 0.027 | 0.51 | |
| *Eisenbergiella* | rs11938607 | T | C | 0.252 | 0.098 | 0.022 | 8.22E-06 | 0.002 | 0.022 | 0.91 | |
| *Eisenbergiella* | rs12257723 | A | C | 0.327 | -0.095 | 0.021 | 8.85E-06 | 0.012 | 0.020 | 0.56 | |
| *Eisenbergiella* | rs12710729 | C | A | 0.320 | 0.089 | 0.020 | 9.84E-06 | 0.008 | 0.020 | 0.70 | |
| *Enterorhabdus* | rs114731706 | T | G | 0.032 | 0.182 | 0.038 | 2.17E-06 | 0.016 | 0.055 | 0.77 | |
| *Enterorhabdus* | rs3017103 | A | G | 0.191 | 0.098 | 0.021 | 2.94E-06 | -0.030 | 0.024 | 0.22 | |
| *Enterorhabdus* | rs73331712 | T | C | 0.042 | 0.262 | 0.055 | 4.85E-06 | -0.137 | 0.050 | 0.01 | |
| *Enterorhabdus* | rs77655283 | G | A | 0.070 | 0.133 | 0.030 | 5.88E-06 | -0.011 | 0.037 | 0.76 | |
| *Enterorhabdus* | rs10098492 | T | C | 0.058 | 0.132 | 0.029 | 6.41E-06 | -0.043 | 0.041 | 0.30 | |
| *Enterorhabdus* | rs2051957 | C | T | 0.200 | 0.084 | 0.019 | 8.90E-06 | -0.014 | 0.024 | 0.56 | |
| *Erysipelatoclostridium* | rs7221249 | A | G | 0.530 | 0.084 | 0.014 | 4.31E-09 | -0.025 | 0.019 | 0.19 | |
| *Erysipelatoclostridium* | rs710230 | T | C | 0.072 | 0.143 | 0.028 | 6.33E-07 | -0.008 | 0.037 | 0.83 | |
| *Erysipelatoclostridium* | rs4697572 | A | G | 0.199 | -0.081 | 0.016 | 7.59E-07 | 0.031 | 0.024 | 0.19 | |
| *Erysipelatoclostridium* | rs58236560 | G | T | 0.121 | -0.111 | 0.023 | 2.16E-06 | -0.006 | 0.029 | 0.84 | |
| *Erysipelatoclostridium* | rs6474512 | A | C | 0.386 | 0.067 | 0.014 | 3.02E-06 | -0.025 | 0.020 | 0.20 | |
| *Erysipelatoclostridium* | rs622418 | A | G | 0.489 | -0.067 | 0.014 | 3.68E-06 | 0.009 | 0.019 | 0.65 | |
| *Erysipelatoclostridium* | rs340991 | A | G | 0.266 | -0.074 | 0.016 | 3.75E-06 | 0.000 | 0.022 | 1.00 | |
| *Erysipelatoclostridium* | rs17804233 | T | C | 0.474 | -0.066 | 0.014 | 4.59E-06 | 0.011 | 0.019 | 0.56 | |
| *Erysipelatoclostridium* | rs16936671 | C | T | 0.139 | -0.097 | 0.022 | 6.04E-06 | -0.007 | 0.028 | 0.80 | |
| *Erysipelatoclostridium* | rs9590927 | G | A | 0.455 | -0.065 | 0.014 | 6.39E-06 | 0.017 | 0.019 | 0.38 | |
| *Erysipelatoclostridium* | rs1434153 | G | A | 0.446 | -0.068 | 0.015 | 6.85E-06 | 0.010 | 0.019 | 0.61 | |
| *Erysipelatoclostridium* | rs45480394 | T | G | 0.362 | -0.069 | 0.015 | 7.66E-06 | -0.034 | 0.020 | 0.09 | |
| *Erysipelatoclostridium* | rs2901723 | C | A | 0.531 | 0.064 | 0.014 | 8.79E-06 | 0.006 | 0.019 | 0.75 | |
| *Erysipelatoclostridium* | rs61806970 | C | T | 0.067 | 0.143 | 0.032 | 9.09E-06 | -0.020 | 0.038 | 0.59 | |
| *Erysipelatoclostridium* | rs3804326 | A | G | 0.050 | 0.141 | 0.034 | 9.85E-06 | 0.019 | 0.044 | 0.68 | |
| *Erysipelotrichaceae (UCG003)* | rs76502207 | T | C | 0.040 | 0.145 | 0.029 | 6.41E-07 | 0.056 | 0.049 | 0.25 | |
| *Erysipelotrichaceae (UCG003)* | rs28568391 | A | G | 0.515 | -0.058 | 0.012 | 6.42E-07 | 0.006 | 0.019 | 0.77 | |
| *Erysipelotrichaceae (UCG003)* | rs10164067 | T | G | 0.051 | -0.103 | 0.021 | 1.13E-06 | -0.009 | 0.044 | 0.84 | |
| *Erysipelotrichaceae (UCG003)* | rs11994308 | C | T | 0.089 | 0.115 | 0.024 | 1.33E-06 | -0.063 | 0.033 | 0.06 | |
| *Erysipelotrichaceae (UCG003)* | rs59068084 | T | G | 0.392 | 0.056 | 0.012 | 3.12E-06 | 0.004 | 0.020 | 0.84 | |
| *Erysipelotrichaceae (UCG003)* | rs17798136 | G | A | 0.068 | 0.159 | 0.035 | 3.24E-06 | 0.026 | 0.039 | 0.50 | |
| *Erysipelotrichaceae (UCG003)* | rs62403464 | T | C | 0.182 | -0.073 | 0.016 | 3.44E-06 | 0.006 | 0.025 | 0.81 | |
| *Erysipelotrichaceae (UCG003)* | rs75949021 | T | C | 0.040 | -0.170 | 0.037 | 3.58E-06 | -0.051 | 0.049 | 0.29 | |
| *Erysipelotrichaceae (UCG003)* | rs59104037 | A | G | 0.129 | -0.095 | 0.020 | 4.48E-06 | -0.024 | 0.029 | 0.41 | |
| *Erysipelotrichaceae (UCG003)* | rs8053479 | A | G | 0.117 | -0.084 | 0.019 | 5.83E-06 | -0.026 | 0.030 | 0.39 | |
| *Erysipelotrichaceae (UCG003)* | rs4758231 | G | T | 0.281 | -0.055 | 0.012 | 6.55E-06 | -0.020 | 0.021 | 0.34 | |
| *Erysipelotrichaceae (UCG003)* | rs6875357 | C | T | 0.038 | 0.166 | 0.035 | 6.70E-06 | 0.001 | 0.050 | 0.98 | |
| *Erysipelotrichaceae (UCG003)* | rs11666127 | A | G | 0.177 | -0.072 | 0.016 | 7.90E-06 | 0.020 | 0.025 | 0.42 | |
| *Erysipelotrichaceae (UCG003)* | rs74988980 | G | A | 0.043 | -0.133 | 0.035 | 8.64E-06 | 0.011 | 0.047 | 0.82 | |
| *Erysipelotrichaceae (UCG003)* | rs12251396 | A | G | 0.154 | -0.071 | 0.016 | 9.52E-06 | -0.015 | 0.026 | 0.58 | |
| *Erysipelotrichaceae (UCG003)* | rs73074432 | C | T | 0.109 | 0.072 | 0.016 | 9.99E-06 | 0.053 | 0.031 | 0.09 | |
| *Escherichia Shigella* | rs73208162 | A | G | 0.032 | -0.119 | 0.025 | 2.19E-06 | 0.008 | 0.055 | 0.89 | |
| *Escherichia Shigella* | rs1154904 | A | G | 0.492 | -0.061 | 0.013 | 3.04E-06 | 0.024 | 0.019 | 0.21 | |
| *Escherichia Shigella* | rs113127095 | A | G | 0.043 | 0.151 | 0.032 | 3.33E-06 | -0.082 | 0.048 | 0.09 | |
| *Escherichia Shigella* | rs592299 | T | C | 0.544 | -0.059 | 0.013 | 4.77E-06 | -0.021 | 0.019 | 0.28 | |
| *Escherichia Shigella* | rs113513883 | A | G | 0.032 | 0.172 | 0.038 | 5.28E-06 | -0.040 | 0.055 | 0.47 | |
| *Escherichia Shigella* | rs4731451 | G | A | 0.316 | -0.061 | 0.014 | 7.47E-06 | 0.003 | 0.020 | 0.89 | |
| *Escherichia Shigella* | rs118526 | C | A | 0.300 | -0.059 | 0.014 | 8.00E-06 | -0.005 | 0.021 | 0.82 | |
| *Escherichia Shigella* | rs112767262 | T | C | 0.223 | 0.073 | 0.016 | 8.21E-06 | -0.022 | 0.023 | 0.35 | |
| *Escherichia Shigella* | rs2798105 | A | G | 0.100 | -0.101 | 0.022 | 8.25E-06 | 0.011 | 0.032 | 0.73 | |
| *Escherichia Shigella* | rs57024273 | T | C | 0.257 | 0.063 | 0.014 | 9.70E-06 | 0.007 | 0.022 | 0.77 | |
| *Eubacterium (brachy group)* | rs112617308 | T | C | 0.092 | -0.171 | 0.036 | 2.38E-06 | 0.008 | 0.033 | 0.81 | |
| *Eubacterium (brachy group)* | rs4862235 | G | A | 0.456 | 0.105 | 0.023 | 3.73E-06 | 0.000 | 0.019 | 0.99 | |
| *Eubacterium (brachy group)* | rs62348779 | T | C | 0.077 | -0.201 | 0.043 | 3.78E-06 | -0.006 | 0.036 | 0.88 | |
| *Eubacterium (brachy group)* | rs2913110 | C | T | 0.350 | 0.105 | 0.023 | 4.56E-06 | -0.001 | 0.020 | 0.98 | |
| *Eubacterium (brachy group)* | rs1384962 | A | G | 0.320 | 0.121 | 0.027 | 6.99E-06 | 0.008 | 0.021 | 0.71 | |
| *Eubacterium (brachy group)* | rs720439 | A | G | 0.247 | -0.112 | 0.025 | 7.03E-06 | -0.017 | 0.022 | 0.45 | |
| *Eubacterium (brachy group)* | rs6591893 | G | A | 0.346 | 0.108 | 0.024 | 7.34E-06 | -0.001 | 0.020 | 0.94 | |
| *Eubacterium (brachy group)* | rs13139592 | T | C | 0.133 | -0.146 | 0.033 | 7.97E-06 | 0.018 | 0.028 | 0.51 | |
| *Eubacterium (brachy group)* | rs73199919 | T | C | 0.049 | -0.237 | 0.053 | 8.16E-06 | -0.016 | 0.044 | 0.72 | |
| *Eubacterium (brachy group)* | rs12151423 | A | G | 0.520 | 0.101 | 0.023 | 9.27E-06 | -0.036 | 0.019 | 0.06 | |
| *Eubacterium (coprostanoligenes group)* | rs17159861 | C | T | 0.108 | 0.096 | 0.017 | 1.04E-08 | 0.009 | 0.031 | 0.76 | |
| *Eubacterium (coprostanoligenes group)* | rs9648214 | T | C | 0.086 | -0.083 | 0.016 | 2.52E-07 | 0.021 | 0.034 | 0.54 | |
| *Eubacterium (coprostanoligenes group)* | rs4076415 | T | G | 0.368 | 0.052 | 0.011 | 1.99E-06 | -0.017 | 0.020 | 0.40 | |
| *Eubacterium (coprostanoligenes group)* | rs11808093 | T | C | 0.154 | 0.079 | 0.017 | 2.97E-06 | 0.018 | 0.027 | 0.51 | |
| *Eubacterium (coprostanoligenes group)* | rs6762473 | C | A | 0.340 | 0.052 | 0.011 | 4.26E-06 | 0.003 | 0.020 | 0.89 | |
| *Eubacterium (coprostanoligenes group)* | rs12906958 | C | T | 0.292 | -0.053 | 0.012 | 4.35E-06 | -0.015 | 0.021 | 0.46 | |
| *Eubacterium (coprostanoligenes group)* | rs76898927 | G | A | 0.054 | 0.123 | 0.027 | 4.79E-06 | 0.049 | 0.042 | 0.25 | |
| *Eubacterium (coprostanoligenes group)* | rs10444197 | A | G | 0.349 | -0.051 | 0.011 | 5.98E-06 | -0.030 | 0.020 | 0.14 | |
| *Eubacterium (coprostanoligenes group)* | rs62024432 | C | T | 0.096 | -0.077 | 0.017 | 7.50E-06 | 0.030 | 0.032 | 0.35 | |
| *Eubacterium (coprostanoligenes group)* | rs79895140 | T | C | 0.115 | -0.064 | 0.014 | 8.62E-06 | -0.056 | 0.031 | 0.07 | |
| *Eubacterium (coprostanoligenes group)* | rs1020520 | T | G | 0.151 | -0.059 | 0.013 | 8.89E-06 | 0.020 | 0.027 | 0.46 | |
| *Eubacterium (coprostanoligenes group)* | rs11720857 | C | T | 0.176 | 0.063 | 0.014 | 9.26E-06 | -0.021 | 0.025 | 0.41 | |
| *Eubacterium (coprostanoligenes group)* | rs11052069 | T | C | 0.441 | 0.048 | 0.011 | 9.38E-06 | -0.008 | 0.019 | 0.68 | |
| *Eubacterium (coprostanoligenes group)* | rs2644213 | G | A | 0.306 | 0.054 | 0.012 | 9.86E-06 | 0.009 | 0.021 | 0.67 | |
| *Eubacterium (eligens group)* | rs4583233 | A | C | 0.289 | 0.067 | 0.013 | 2.84E-07 | -0.023 | 0.021 | 0.28 | |
| *Eubacterium (eligens group)* | rs265534 | T | G | 0.468 | -0.056 | 0.012 | 2.27E-06 | -0.005 | 0.019 | 0.79 | |
| *Eubacterium (eligens group)* | rs6923695 | T | G | 0.067 | 0.103 | 0.023 | 4.87E-06 | -0.009 | 0.038 | 0.82 | |
| *Eubacterium (eligens group)* | rs2200429 | A | G | 0.098 | -0.089 | 0.020 | 5.30E-06 | 0.031 | 0.032 | 0.33 | |
| *Eubacterium (eligens group)* | rs182318 | G | A | 0.081 | -0.082 | 0.020 | 8.40E-06 | -0.013 | 0.035 | 0.71 | |
| *Eubacterium (eligens group)* | rs56080211 | C | T | 0.078 | 0.123 | 0.028 | 9.14E-06 | -0.002 | 0.036 | 0.96 | |
| *Eubacterium (fissicatena group)* | rs3771393 | C | T | 0.193 | 0.131 | 0.027 | 7.38E-07 | 0.001 | 0.024 | 0.97 | |
| *Eubacterium (fissicatena group)* | rs2733072 | G | A | 0.504 | 0.110 | 0.023 | 1.49E-06 | -0.007 | 0.019 | 0.72 | |
| *Eubacterium (fissicatena group)* | rs11876297 | T | C | 0.283 | 0.131 | 0.028 | 2.67E-06 | 0.015 | 0.022 | 0.49 | |
| *Eubacterium (fissicatena group)* | rs7104872 | G | A | 0.110 | 0.139 | 0.029 | 2.73E-06 | 0.004 | 0.031 | 0.90 | |
| *Eubacterium (fissicatena group)* | rs151257695 | A | G | 0.071 | 0.210 | 0.045 | 3.10E-06 | 0.012 | 0.037 | 0.75 | |
| *Eubacterium (fissicatena group)* | rs11818408 | G | A | 0.396 | 0.106 | 0.024 | 8.20E-06 | -0.017 | 0.020 | 0.38 | |
| *Eubacterium (fissicatena group)* | rs10147907 | T | G | 0.080 | 0.172 | 0.040 | 8.27E-06 | 0.025 | 0.036 | 0.48 | |
| *Eubacterium (fissicatena group)* | rs1768152 | T | C | 0.105 | 0.139 | 0.032 | 8.70E-06 | 0.022 | 0.031 | 0.48 | |
| *Eubacterium (fissicatena group)* | rs6934739 | A | G | 0.333 | 0.111 | 0.025 | 9.75E-06 | 0.026 | 0.020 | 0.20 | |
| *Eubacterium (hallii group)* | rs13116360 | T | C | 0.066 | 0.154 | 0.030 | 2.94E-07 | -0.047 | 0.039 | 0.23 | |
| *Eubacterium (hallii group)* | rs10798999 | C | T | 0.268 | 0.060 | 0.013 | 2.61E-06 | 0.022 | 0.022 | 0.32 | |
| *Eubacterium (hallii group)* | rs60254196 | A | G | 0.465 | -0.052 | 0.011 | 2.70E-06 | -0.011 | 0.019 | 0.56 | |
| *Eubacterium (hallii group)* | rs949971 | T | G | 0.325 | -0.054 | 0.012 | 3.29E-06 | -0.028 | 0.020 | 0.17 | |
| *Eubacterium (hallii group)* | rs74018587 | C | T | 0.040 | 0.209 | 0.044 | 3.70E-06 | 0.003 | 0.049 | 0.95 | |
| *Eubacterium (hallii group)* | rs10808115 | A | C | 0.490 | -0.050 | 0.011 | 4.42E-06 | 0.011 | 0.019 | 0.56 | |
| *Eubacterium (hallii group)* | rs28584818 | A | G | 0.080 | 0.126 | 0.027 | 4.43E-06 | 0.015 | 0.036 | 0.68 | |
| *Eubacterium (hallii group)* | rs6550770 | T | C | 0.043 | -0.198 | 0.044 | 4.82E-06 | -0.022 | 0.048 | 0.65 | |
| *Eubacterium (hallii group)* | rs10501370 | C | T | 0.063 | -0.116 | 0.025 | 5.42E-06 | 0.005 | 0.039 | 0.90 | |
| *Eubacterium (hallii group)* | rs11774814 | T | C | 0.304 | -0.127 | 0.029 | 7.86E-06 | 0.029 | 0.021 | 0.16 | |
| *Eubacterium (hallii group)* | rs78056098 | G | T | 0.355 | -0.051 | 0.011 | 8.29E-06 | -0.011 | 0.020 | 0.58 | |
| *Eubacterium (hallii group)* | rs630939 | C | T | 0.453 | -0.051 | 0.011 | 9.16E-06 | -0.010 | 0.019 | 0.59 | |
| *Eubacterium (hallii group)* | rs281379 | A | G | 0.408 | -0.050 | 0.011 | 9.33E-06 | 0.004 | 0.019 | 0.82 | |
| *Eubacterium (hallii group)* | rs17074066 | T | C | 0.020 | -0.081 | 0.019 | 9.35E-06 | 0.036 | 0.070 | 0.61 | |
| *Eubacterium (hallii group)* | rs17474256 | G | A | 0.098 | 0.081 | 0.018 | 9.45E-06 | -0.007 | 0.032 | 0.83 | |
| *Eubacterium (nodatum group)* | rs34297067 | A | G | 0.147 | -0.187 | 0.034 | 6.60E-08 | -0.006 | 0.027 | 0.84 | |
| *Eubacterium (nodatum group)* | rs77910827 | C | T | 0.108 | 0.202 | 0.041 | 9.05E-07 | -0.004 | 0.031 | 0.90 | |
| *Eubacterium (nodatum group)* | rs61841040 | G | T | 0.197 | 0.161 | 0.034 | 3.56E-06 | 0.045 | 0.024 | 0.07 | |
| *Eubacterium (nodatum group)* | rs113893692 | C | T | 0.123 | -0.185 | 0.040 | 5.76E-06 | -0.040 | 0.029 | 0.18 | |
| *Eubacterium (nodatum group)* | rs7880204 | T | C | 0.245 | -0.125 | 0.028 | 6.84E-06 | 0.067 | 0.022 | 0.00 | |
| *Eubacterium (nodatum group)* | rs7827125 | C | T | 0.279 | 0.122 | 0.027 | 7.17E-06 | -0.011 | 0.021 | 0.61 | |
| *Eubacterium (nodatum group)* | rs9425984 | T | C | 0.218 | -0.130 | 0.029 | 7.21E-06 | -0.011 | 0.023 | 0.64 | |
| *Eubacterium (nodatum group)* | rs6818880 | A | G | 0.467 | -0.110 | 0.025 | 7.83E-06 | 0.035 | 0.019 | 0.07 | |
| *Eubacterium (nodatum group)* | rs11006576 | A | G | 0.540 | -0.110 | 0.025 | 7.99E-06 | 0.000 | 0.019 | 0.99 | |
| *Eubacterium (nodatum group)* | rs10458299 | T | C | 0.075 | -0.188 | 0.042 | 8.37E-06 | 0.023 | 0.036 | 0.53 | |
| *Eubacterium (nodatum group)* | rs10263623 | C | T | 0.038 | 0.193 | 0.044 | 8.91E-06 | 0.105 | 0.051 | 0.04 | |
| *Eubacterium (oxidoreducens group)* | rs440215 | C | T | 0.440 | 0.093 | 0.020 | 1.65E-06 | -0.011 | 0.019 | 0.56 | |
| *Eubacterium (oxidoreducens group)* | rs2973294 | G | T | 0.414 | 0.092 | 0.020 | 2.39E-06 | -0.008 | 0.019 | 0.70 | |
| *Eubacterium (oxidoreducens group)* | rs34561138 | G | A | 0.039 | 0.216 | 0.046 | 2.51E-06 | -0.051 | 0.051 | 0.32 | |
| *Eubacterium (oxidoreducens group)* | rs12423772 | G | T | 0.152 | 0.141 | 0.030 | 2.63E-06 | 0.019 | 0.027 | 0.50 | |
| *Eubacterium (oxidoreducens group)* | rs12129908 | C | A | 0.398 | 0.089 | 0.020 | 5.80E-06 | -0.008 | 0.020 | 0.70 | |
| *Eubacterium (rectale group)* | rs35398954 | A | G | 0.160 | -0.090 | 0.017 | 5.40E-07 | -0.016 | 0.026 | 0.55 | |
| *Eubacterium (rectale group)* | rs314726 | T | C | 0.496 | 0.053 | 0.011 | 1.38E-06 | 0.003 | 0.019 | 0.86 | |
| *Eubacterium (rectale group)* | rs10797540 | A | G | 0.445 | 0.050 | 0.011 | 3.53E-06 | -0.026 | 0.019 | 0.17 | |
| *Eubacterium (rectale group)* | rs10248854 | C | A | 0.396 | -0.053 | 0.011 | 4.21E-06 | 0.033 | 0.020 | 0.09 | |
| *Eubacterium (rectale group)* | rs59427698 | A | G | 0.189 | -0.058 | 0.013 | 5.37E-06 | -0.022 | 0.025 | 0.37 | |
| *Eubacterium (rectale group)* | rs2884897 | A | G | 0.035 | -0.129 | 0.029 | 6.44E-06 | -0.022 | 0.052 | 0.68 | |
| *Eubacterium (rectale group)* | rs143694765 | T | C | 0.102 | 0.087 | 0.020 | 9.75E-06 | -0.006 | 0.032 | 0.85 | |
| *Eubacterium (rectale group)* | rs62547233 | A | G | 0.283 | 0.054 | 0.012 | 9.90E-06 | -0.030 | 0.021 | 0.15 | |
| *Eubacterium (ruminantium group)* | rs2116427 | A | G | 0.255 | 0.091 | 0.018 | 4.67E-07 | -0.018 | 0.022 | 0.41 | |
| *Eubacterium (ruminantium group)* | rs139749 | C | T | 0.341 | -0.085 | 0.017 | 8.59E-07 | 0.005 | 0.020 | 0.80 | |
| *Eubacterium (ruminantium group)* | rs2229917 | A | G | 0.042 | 0.154 | 0.032 | 2.16E-06 | 0.032 | 0.048 | 0.51 | |
| *Eubacterium (ruminantium group)* | rs16891896 | G | A | 0.086 | -0.175 | 0.039 | 2.38E-06 | -0.011 | 0.034 | 0.74 | |
| *Eubacterium (ruminantium group)* | rs10131724 | A | C | 0.100 | -0.200 | 0.041 | 2.39E-06 | -0.030 | 0.032 | 0.35 | |
| *Eubacterium (ruminantium group)* | rs72836424 | C | T | 0.112 | -0.140 | 0.030 | 2.62E-06 | -0.014 | 0.030 | 0.65 | |
| *Eubacterium (ruminantium group)* | rs7000472 | A | G | 0.416 | -0.076 | 0.017 | 4.07E-06 | 0.019 | 0.019 | 0.32 | |
| *Eubacterium (ruminantium group)* | rs17519472 | C | T | 0.139 | 0.108 | 0.023 | 4.70E-06 | -0.030 | 0.028 | 0.28 | |
| *Eubacterium (ruminantium group)* | rs606117 | A | G | 0.271 | 0.083 | 0.018 | 4.82E-06 | -0.018 | 0.021 | 0.39 | |
| *Eubacterium (ruminantium group)* | rs57340348 | T | C | 0.202 | -0.098 | 0.021 | 4.93E-06 | 0.014 | 0.024 | 0.56 | |
| *Eubacterium (ruminantium group)* | rs73139629 | A | C | 0.092 | -0.115 | 0.025 | 5.36E-06 | -0.046 | 0.033 | 0.17 | |
| *Eubacterium (ruminantium group)* | rs11637981 | G | T | 0.557 | -0.073 | 0.016 | 5.44E-06 | 0.060 | 0.019 | 0.00 | |
| *Eubacterium (ruminantium group)* | rs2418654 | C | T | 0.432 | -0.075 | 0.017 | 6.17E-06 | -0.006 | 0.020 | 0.75 | |
| *Eubacterium (ruminantium group)* | rs6676699 | G | T | 0.300 | -0.089 | 0.020 | 6.38E-06 | 0.042 | 0.021 | 0.04 | |
| *Eubacterium (ruminantium group)* | rs10923018 | G | A | 0.579 | 0.073 | 0.016 | 6.80E-06 | -0.003 | 0.019 | 0.89 | |
| *Eubacterium (ruminantium group)* | rs13025464 | T | C | 0.403 | -0.074 | 0.016 | 6.97E-06 | 0.009 | 0.019 | 0.64 | |
| *Eubacterium (ruminantium group)* | rs2817174 | C | T | 0.395 | -0.073 | 0.016 | 7.87E-06 | -0.013 | 0.020 | 0.52 | |
| *Eubacterium (ruminantium group)* | rs209813 | G | A | 0.145 | -0.103 | 0.024 | 9.23E-06 | -0.031 | 0.027 | 0.25 | |
| *Eubacterium (ventriosum group)* | rs11617697 | A | G | 0.056 | -0.143 | 0.029 | 7.22E-07 | 0.062 | 0.042 | 0.14 | |
| *Eubacterium (ventriosum group)* | rs57199565 | T | C | 0.199 | 0.078 | 0.016 | 7.97E-07 | -0.053 | 0.025 | 0.03 | |
| *Eubacterium (ventriosum group)* | rs73615400 | T | C | 0.098 | -0.096 | 0.019 | 9.54E-07 | 0.026 | 0.032 | 0.41 | |
| *Eubacterium (ventriosum group)* | rs16884680 | G | T | 0.104 | -0.091 | 0.019 | 1.74E-06 | 0.018 | 0.031 | 0.56 | |
| *Eubacterium (ventriosum group)* | rs12964517 | G | A | 0.283 | 0.059 | 0.012 | 2.07E-06 | -0.004 | 0.021 | 0.86 | |
| *Eubacterium (ventriosum group)* | rs876734 | C | T | 0.283 | -0.062 | 0.013 | 2.89E-06 | -0.012 | 0.021 | 0.58 | |
| *Eubacterium (ventriosum group)* | rs78250280 | G | A | 0.140 | 0.075 | 0.016 | 3.36E-06 | -0.027 | 0.028 | 0.33 | |
| *Eubacterium (ventriosum group)* | rs3809430 | T | C | 0.308 | -0.055 | 0.012 | 3.55E-06 | 0.011 | 0.021 | 0.58 | |
| *Eubacterium (ventriosum group)* | rs73849225 | T | C | 0.083 | 0.098 | 0.022 | 5.21E-06 | -0.073 | 0.035 | 0.04 | |
| *Eubacterium (ventriosum group)* | rs35179274 | C | T | 0.178 | -0.063 | 0.014 | 5.76E-06 | 0.008 | 0.025 | 0.75 | |
| *Eubacterium (ventriosum group)* | rs66746423 | C | T | 0.153 | 0.075 | 0.016 | 6.11E-06 | -0.004 | 0.027 | 0.88 | |
| *Eubacterium (ventriosum group)* | rs72783037 | C | A | 0.206 | 0.066 | 0.014 | 6.55E-06 | -0.031 | 0.024 | 0.19 | |
| *Eubacterium (ventriosum group)* | rs6704822 | A | G | 0.130 | 0.074 | 0.017 | 6.62E-06 | -0.015 | 0.028 | 0.58 | |
| *Eubacterium (ventriosum group)* | rs9316536 | T | G | 0.145 | -0.082 | 0.018 | 7.84E-06 | 0.001 | 0.028 | 0.96 | |
| *Eubacterium (ventriosum group)* | rs13082419 | C | T | 0.111 | -0.072 | 0.016 | 9.56E-06 | 0.026 | 0.030 | 0.39 | |
| *Eubacterium (xylanophilum group)* | rs17830032 | G | A | 0.081 | -0.161 | 0.031 | 2.39E-07 | 0.029 | 0.035 | 0.41 | |
| *Eubacterium (xylanophilum group)* | rs13239072 | G | A | 0.276 | 0.069 | 0.014 | 1.82E-06 | 0.008 | 0.021 | 0.71 | |
| *Eubacterium (xylanophilum group)* | rs10917203 | A | C | 0.394 | 0.061 | 0.013 | 3.15E-06 | -0.007 | 0.020 | 0.72 | |
| *Eubacterium (xylanophilum group)* | rs112176119 | C | T | 0.093 | -0.113 | 0.025 | 3.33E-06 | -0.032 | 0.033 | 0.33 | |
| *Eubacterium (xylanophilum group)* | rs1999224 | G | T | 0.098 | -0.095 | 0.020 | 3.75E-06 | 0.060 | 0.032 | 0.06 | |
| *Eubacterium (xylanophilum group)* | rs2213117 | T | G | 0.164 | 0.088 | 0.019 | 4.21E-06 | -0.010 | 0.026 | 0.71 | |
| *Eubacterium (xylanophilum group)* | rs10140184 | A | C | 0.475 | 0.058 | 0.013 | 4.96E-06 | 0.015 | 0.020 | 0.44 | |
| *Eubacterium (xylanophilum group)* | rs2012708 | A | G | 0.343 | 0.057 | 0.013 | 6.53E-06 | -0.013 | 0.020 | 0.52 | |
| *Eubacterium (xylanophilum group)* | rs75586835 | A | G | 0.071 | -0.114 | 0.026 | 9.39E-06 | -0.020 | 0.037 | 0.59 | |
| *Faecalibacterium* | rs1271565 | C | T | 0.261 | -0.058 | 0.012 | 1.30E-06 | -0.002 | 0.022 | 0.94 | |
| *Faecalibacterium* | rs6910935 | A | G | 0.061 | 0.135 | 0.028 | 1.38E-06 | 0.051 | 0.040 | 0.21 | |
| *Faecalibacterium* | rs75499067 | C | T | 0.075 | 0.228 | 0.047 | 1.76E-06 | 0.008 | 0.037 | 0.82 | |
| *Faecalibacterium* | rs9536330 | T | C | 0.472 | -0.048 | 0.011 | 5.33E-06 | -0.053 | 0.019 | 0.01 | |
| *Faecalibacterium* | rs114946999 | C | T | 0.126 | -0.086 | 0.019 | 5.70E-06 | 0.006 | 0.029 | 0.84 | |
| *Faecalibacterium* | rs11776390 | T | C | 0.066 | -0.078 | 0.017 | 6.40E-06 | -0.022 | 0.038 | 0.57 | |
| *Faecalibacterium* | rs10927394 | G | T | 0.019 | -0.232 | 0.051 | 7.02E-06 | -0.027 | 0.071 | 0.71 | |
| *Faecalibacterium* | rs2835874 | T | C | 0.035 | -0.087 | 0.020 | 7.54E-06 | 0.045 | 0.052 | 0.39 | |
| *Faecalibacterium* | rs79656633 | T | C | 0.101 | 0.146 | 0.032 | 8.14E-06 | -0.021 | 0.032 | 0.51 | |
| *Faecalibacterium* | rs12753492 | A | C | 0.113 | 0.064 | 0.015 | 8.80E-06 | 0.019 | 0.030 | 0.52 | |
| *Family XIIIAD (3011 group)* | rs62200412 | C | T | 0.262 | -0.080 | 0.016 | 5.80E-07 | 0.015 | 0.022 | 0.50 | |
| *Family XIIIAD (3011 group)* | rs16840310 | A | G | 0.429 | -0.061 | 0.012 | 6.75E-07 | 0.033 | 0.019 | 0.09 | |
| *Family XIIIAD (3011 group)* | rs72730932 | C | A | 0.094 | -0.090 | 0.018 | 6.89E-07 | -0.006 | 0.033 | 0.85 | |
| *Family XIIIAD (3011 group)* | rs12812672 | T | C | 0.072 | -0.096 | 0.021 | 2.56E-06 | 0.047 | 0.037 | 0.20 | |
| *Family XIIIAD (3011 group)* | rs62029761 | A | G | 0.058 | 0.129 | 0.028 | 3.89E-06 | 0.036 | 0.042 | 0.40 | |
| *Family XIIIAD (3011 group)* | rs16940167 | C | T | 0.191 | 0.073 | 0.016 | 3.91E-06 | -0.027 | 0.024 | 0.27 | |
| *Family XIIIAD (3011 group)* | rs17156849 | G | A | 0.059 | -0.113 | 0.025 | 4.19E-06 | 0.017 | 0.041 | 0.67 | |
| *Family XIIIAD (3011 group)* | rs11126423 | C | T | 0.094 | 0.090 | 0.020 | 5.91E-06 | -0.065 | 0.034 | 0.06 | |
| *Family XIIIAD (3011 group)* | rs149302 | T | C | 0.232 | -0.065 | 0.014 | 7.48E-06 | -0.028 | 0.023 | 0.22 | |
| *Family XIIIAD (3011 group)* | rs739451 | C | T | 0.206 | 0.065 | 0.015 | 7.88E-06 | -0.035 | 0.024 | 0.14 | |
| *Family XIIIAD (3011 group)* | rs9837139 | A | G | 0.085 | 0.108 | 0.024 | 8.71E-06 | 0.000 | 0.034 | 1.00 | |
| *Family XIIIAD (3011 group)* | rs9276029 | A | G | 0.194 | -0.081 | 0.019 | 8.93E-06 | 0.011 | 0.024 | 0.66 | |
| *Family XIIIAD (3011 group)* | rs11736617 | G | A | 0.055 | -0.076 | 0.017 | 9.02E-06 | -0.014 | 0.043 | 0.73 | |
| *Family XIII (UCG001)* | rs12049454 | T | C | 0.396 | -0.065 | 0.013 | 1.17E-06 | -0.001 | 0.020 | 0.96 | |
| *Family XIII (UCG001)* | rs1426266 | T | C | 0.272 | -0.067 | 0.014 | 1.25E-06 | -0.011 | 0.022 | 0.62 | |
| *Family XIII (UCG001)* | rs7119679 | G | A | 0.241 | -0.081 | 0.017 | 3.52E-06 | -0.004 | 0.022 | 0.86 | |
| *Family XIII (UCG001)* | rs76463770 | A | G | 0.030 | 0.193 | 0.042 | 3.77E-06 | 0.040 | 0.056 | 0.48 | |
| *Family XIII (UCG001)* | rs62414802 | C | T | 0.250 | -0.061 | 0.013 | 4.29E-06 | 0.042 | 0.022 | 0.06 | |
| *Family XIII (UCG001)* | rs3842897 | G | A | 0.089 | -0.113 | 0.024 | 5.20E-06 | 0.033 | 0.034 | 0.33 | |
| *Family XIII (UCG001)* | rs112362903 | A | G | 0.034 | -0.149 | 0.033 | 7.88E-06 | 0.083 | 0.056 | 0.14 | |
| *Family XIII (UCG001)* | rs8076666 | A | G | 0.120 | 0.089 | 0.020 | 8.02E-06 | 0.029 | 0.029 | 0.33 | |
| *Flavonifractor* | rs12030302 | A | G | 0.471 | -0.069 | 0.014 | 5.61E-07 | -0.002 | 0.019 | 0.94 | |
| *Flavonifractor* | rs806808 | T | C | 0.441 | 0.067 | 0.014 | 1.18E-06 | 0.010 | 0.019 | 0.62 | |
| *Flavonifractor* | rs34066017 | A | G | 0.204 | 0.076 | 0.016 | 1.52E-06 | 0.019 | 0.024 | 0.42 | |
| *Flavonifractor* | rs11811696 | T | C | 0.082 | -0.116 | 0.024 | 2.07E-06 | 0.007 | 0.035 | 0.84 | |
| *Fusicatenibacter* | rs4378146 | A | C | 0.255 | -0.062 | 0.013 | 7.20E-07 | 0.024 | 0.022 | 0.27 | |
| *Fusicatenibacter* | rs704418 | T | C | 0.131 | 0.074 | 0.015 | 7.77E-07 | -0.033 | 0.029 | 0.25 | |
| *Fusicatenibacter* | rs2132128 | G | A | 0.102 | -0.077 | 0.016 | 1.08E-06 | -0.024 | 0.032 | 0.46 | |
| *Fusicatenibacter* | rs62353480 | A | G | 0.174 | -0.070 | 0.015 | 1.57E-06 | 0.028 | 0.026 | 0.27 | |
| *Fusicatenibacter* | rs2025938 | G | A | 0.066 | -0.097 | 0.021 | 2.99E-06 | -0.011 | 0.039 | 0.78 | |
| *Fusicatenibacter* | rs3303 | T | C | 0.065 | -0.095 | 0.020 | 3.94E-06 | 0.033 | 0.039 | 0.40 | |
| *Fusicatenibacter* | rs62187631 | T | C | 0.192 | -0.071 | 0.016 | 4.55E-06 | 0.025 | 0.024 | 0.31 | |
| *Fusicatenibacter* | rs8063430 | T | C | 0.051 | -0.104 | 0.022 | 4.93E-06 | 0.007 | 0.044 | 0.87 | |
| *Fusicatenibacter* | rs1864685 | A | C | 0.408 | -0.049 | 0.011 | 4.96E-06 | 0.010 | 0.019 | 0.62 | |
| *Fusicatenibacter* | rs60254196 | A | G | 0.465 | -0.049 | 0.011 | 5.47E-06 | -0.011 | 0.019 | 0.56 | |
| *Fusicatenibacter* | rs167879 | C | T | 0.148 | -0.066 | 0.015 | 5.87E-06 | 0.022 | 0.027 | 0.42 | |
| *Fusicatenibacter* | rs6515626 | G | A | 0.067 | 0.142 | 0.031 | 7.29E-06 | -0.017 | 0.038 | 0.67 | |
| *Fusicatenibacter* | rs9905659 | G | A | 0.184 | -0.062 | 0.014 | 7.31E-06 | -0.054 | 0.025 | 0.03 | |
| *Fusicatenibacter* | rs10439674 | A | G | 0.202 | -0.057 | 0.013 | 7.68E-06 | -0.007 | 0.024 | 0.78 | |
| *Fusicatenibacter* | rs8028026 | A | G | 0.093 | -0.079 | 0.018 | 8.06E-06 | 0.028 | 0.033 | 0.40 | |
| *Fusicatenibacter* | rs73103914 | A | G | 0.150 | -0.060 | 0.013 | 8.30E-06 | -0.015 | 0.027 | 0.57 | |
| *Fusicatenibacter* | rs792108 | T | C | 0.410 | -0.051 | 0.011 | 8.50E-06 | -0.026 | 0.019 | 0.17 | |
| *Fusicatenibacter* | rs206581 | A | G | 0.218 | -0.057 | 0.013 | 8.96E-06 | 0.007 | 0.023 | 0.77 | |
| *Gordonibacter* | rs7294633 | C | T | 0.273 | 0.129 | 0.025 | 3.44E-07 | 0.021 | 0.022 | 0.34 | |
| *Gordonibacter* | rs72714787 | C | A | 0.132 | 0.181 | 0.038 | 1.43E-06 | 0.037 | 0.028 | 0.19 | |
| *Gordonibacter* | rs322296 | G | A | 0.074 | 0.179 | 0.038 | 4.02E-06 | -0.002 | 0.037 | 0.95 | |
| *Gordonibacter* | rs16955299 | G | A | 0.099 | -0.196 | 0.043 | 6.37E-06 | -0.006 | 0.032 | 0.86 | |
| *Gordonibacter* | rs3765837 | T | G | 0.072 | -0.191 | 0.043 | 7.17E-06 | -0.009 | 0.037 | 0.81 | |
| *Gordonibacter* | rs768830 | G | A | 0.157 | 0.150 | 0.033 | 7.76E-06 | -0.030 | 0.026 | 0.26 | |
| *Gordonibacter* | rs72939513 | A | G | 0.051 | -0.214 | 0.049 | 7.98E-06 | -0.036 | 0.044 | 0.42 | |
| *Gordonibacter* | rs35042269 | C | A | 0.113 | -0.180 | 0.040 | 8.11E-06 | -0.021 | 0.030 | 0.49 | |
| *Gordonibacter* | rs13412653 | A | C | 0.371 | 0.108 | 0.024 | 8.61E-06 | 0.001 | 0.020 | 0.96 | |
| *Gordonibacter* | rs4596722 | A | G | 0.501 | 0.103 | 0.023 | 9.06E-06 | -0.030 | 0.019 | 0.12 | |
| *Haemophilus* | rs9382510 | C | T | 0.263 | -0.094 | 0.017 | 7.12E-08 | 0.035 | 0.022 | 0.11 | |
| *Haemophilus* | rs111582866 | G | A | 0.087 | -0.124 | 0.026 | 1.27E-06 | -0.010 | 0.034 | 0.76 | |
| *Haemophilus* | rs9328464 | T | C | 0.447 | 0.072 | 0.015 | 1.42E-06 | -0.032 | 0.019 | 0.09 | |
| *Haemophilus* | rs78909003 | T | C | 0.055 | -0.246 | 0.050 | 1.67E-06 | -0.039 | 0.043 | 0.36 | |
| *Haemophilus* | rs76022354 | C | T | 0.049 | 0.245 | 0.051 | 1.83E-06 | 0.062 | 0.044 | 0.16 | |
| *Haemophilus* | rs35509 | G | A | 0.042 | 0.128 | 0.027 | 2.01E-06 | 0.008 | 0.048 | 0.87 | |
| *Haemophilus* | rs9895850 | T | C | 0.044 | -0.193 | 0.042 | 2.14E-06 | 0.118 | 0.047 | 0.01 | |
| *Haemophilus* | rs4822728 | T | C | 0.483 | 0.071 | 0.015 | 3.48E-06 | 0.023 | 0.019 | 0.22 | |
| *Haemophilus* | rs10781340 | G | A | 0.125 | 0.095 | 0.020 | 4.32E-06 | 0.012 | 0.029 | 0.67 | |
| *Holdemanella* | rs607782 | T | C | 0.391 | -0.085 | 0.017 | 7.19E-07 | -0.006 | 0.020 | 0.76 | |
| *Holdemanella* | rs73011279 | T | C | 0.235 | -0.096 | 0.020 | 1.36E-06 | -0.024 | 0.023 | 0.30 | |
| *Holdemanella* | rs75764681 | T | C | 0.045 | -0.283 | 0.060 | 1.94E-06 | 0.049 | 0.047 | 0.30 | |
| *Holdemanella* | rs4541991 | T | C | 0.323 | -0.093 | 0.019 | 2.10E-06 | 0.017 | 0.020 | 0.41 | |
| *Holdemanella* | rs8113760 | G | A | 0.311 | 0.079 | 0.017 | 4.62E-06 | -0.016 | 0.021 | 0.45 | |
| *Holdemanella* | rs12513188 | G | A | 0.270 | 0.090 | 0.020 | 4.65E-06 | 0.037 | 0.022 | 0.08 | |
| *Holdemanella* | rs34187114 | C | A | 0.111 | -0.105 | 0.023 | 5.13E-06 | -0.021 | 0.031 | 0.50 | |
| *Holdemanella* | rs62113381 | T | C | 0.131 | -0.105 | 0.023 | 5.54E-06 | 0.023 | 0.028 | 0.41 | |
| *Holdemanella* | rs35228298 | G | A | 0.160 | 0.093 | 0.020 | 7.30E-06 | 0.029 | 0.026 | 0.26 | |
| *Holdemanella* | rs1926302 | G | A | 0.226 | -0.108 | 0.023 | 7.50E-06 | -0.005 | 0.023 | 0.84 | |
| *Holdemanella* | rs17586763 | T | C | 0.051 | -0.227 | 0.051 | 7.72E-06 | -0.112 | 0.044 | 0.01 | |
| *Holdemania* | rs1867876 | T | C | 0.298 | 0.084 | 0.016 | 2.74E-07 | -0.014 | 0.021 | 0.50 | |
| *Holdemania* | rs9500080 | C | T | 0.172 | 0.093 | 0.018 | 4.09E-07 | -0.009 | 0.026 | 0.71 | |
| *Holdemania* | rs77293403 | A | G | 0.035 | 0.165 | 0.034 | 1.77E-06 | -0.041 | 0.052 | 0.43 | |
| *Holdemania* | rs116500994 | G | T | 0.047 | -0.138 | 0.029 | 2.34E-06 | 0.009 | 0.045 | 0.85 | |
| *Holdemania* | rs111745969 | A | G | 0.136 | 0.121 | 0.027 | 3.71E-06 | -0.015 | 0.028 | 0.60 | |
| *Holdemania* | rs9529719 | T | C | 0.323 | 0.074 | 0.016 | 5.97E-06 | -0.008 | 0.020 | 0.69 | |
| *Holdemania* | rs80149660 | C | T | 0.043 | -0.233 | 0.052 | 6.04E-06 | 0.125 | 0.045 | 0.01 | |
| *Holdemania* | rs11080063 | G | A | 0.406 | -0.067 | 0.015 | 6.67E-06 | 0.004 | 0.019 | 0.85 | |
| *Holdemania* | rs4146507 | C | T | 0.243 | 0.079 | 0.018 | 7.23E-06 | 0.001 | 0.022 | 0.97 | |
| *Holdemania* | rs73139538 | G | A | 0.030 | -0.149 | 0.033 | 7.77E-06 | 0.012 | 0.057 | 0.83 | |
| *Holdemania* | rs967319 | T | C | 0.240 | 0.079 | 0.018 | 8.38E-06 | -0.021 | 0.022 | 0.35 | |
| *Holdemania* | rs10885477 | T | C | 0.050 | -0.135 | 0.030 | 8.60E-06 | 0.076 | 0.044 | 0.09 | |
| *Holdemania* | rs113593397 | A | G | 0.097 | -0.129 | 0.028 | 9.36E-06 | -0.040 | 0.032 | 0.22 | |
| *Holdemania* | rs12701617 | A | G | 0.464 | -0.066 | 0.015 | 9.52E-06 | -0.017 | 0.019 | 0.39 | |
| *Howardella* | rs17167098 | G | A | 0.137 | -0.169 | 0.035 | 1.12E-06 | -0.014 | 0.028 | 0.63 | |
| *Howardella* | rs1484873 | A | G | 0.154 | -0.228 | 0.046 | 2.56E-06 | -0.019 | 0.026 | 0.47 | |
| *Howardella* | rs609430 | T | G | 0.350 | -0.112 | 0.024 | 3.34E-06 | 0.039 | 0.020 | 0.05 | |
| *Howardella* | rs672217 | G | A | 0.199 | 0.164 | 0.035 | 3.52E-06 | 0.000 | 0.024 | 1.00 | |
| *Howardella* | rs12452946 | A | G | 0.498 | -0.106 | 0.023 | 3.80E-06 | -0.014 | 0.019 | 0.45 | |
| *Howardella* | rs36081916 | T | C | 0.089 | -0.181 | 0.040 | 4.70E-06 | 0.056 | 0.034 | 0.10 | |
| *Howardella* | rs10048062 | C | T | 0.089 | -0.147 | 0.034 | 8.59E-06 | 0.028 | 0.034 | 0.42 | |
| *Howardella* | rs3791893 | A | G | 0.142 | 0.147 | 0.034 | 9.50E-06 | -0.023 | 0.027 | 0.40 | |
| *Howardella* | rs2154047 | C | A | 0.088 | -0.193 | 0.042 | 9.97E-06 | 0.009 | 0.034 | 0.78 | |
| *Hungatella* | rs13128780 | T | C | 0.187 | -0.150 | 0.031 | 1.75E-06 | 0.026 | 0.024 | 0.29 | |
| *Hungatella* | rs72759041 | G | T | 0.208 | -0.126 | 0.028 | 3.86E-06 | -0.031 | 0.024 | 0.19 | |
| *Hungatella* | rs17092615 | G | A | 0.137 | 0.152 | 0.034 | 7.38E-06 | -0.021 | 0.028 | 0.44 | |
| *Hungatella* | rs10044993 | C | A | 0.085 | 0.140 | 0.032 | 8.07E-06 | 0.032 | 0.034 | 0.36 | |
| *Hungatella* | rs13249325 | T | G | 0.470 | -0.100 | 0.023 | 9.69E-06 | -0.010 | 0.019 | 0.60 | |
| *Intestinibacter* | rs10805326 | G | A | 0.294 | 0.078 | 0.014 | 3.55E-08 | 0.002 | 0.021 | 0.92 | |
| *Intestinibacter* | rs4327025 | G | A | 0.185 | -0.081 | 0.015 | 1.64E-07 | 0.040 | 0.025 | 0.11 | |
| *Intestinibacter* | rs16938435 | T | C | 0.097 | -0.112 | 0.024 | 1.80E-06 | -0.045 | 0.032 | 0.16 | |
| *Intestinibacter* | rs478972 | T | C | 0.092 | -0.143 | 0.030 | 1.82E-06 | 0.043 | 0.033 | 0.19 | |
| *Intestinibacter* | rs6875660 | C | T | 0.061 | 0.089 | 0.019 | 3.06E-06 | 0.057 | 0.040 | 0.16 | |
| *Intestinibacter* | rs2702387 | A | G | 0.405 | 0.061 | 0.013 | 4.26E-06 | -0.023 | 0.019 | 0.23 | |
| *Intestinibacter* | rs12449107 | T | C | 0.045 | -0.147 | 0.032 | 5.26E-06 | 0.011 | 0.046 | 0.81 | |
| *Intestinibacter* | rs11109097 | C | T | 0.492 | 0.062 | 0.014 | 5.49E-06 | -0.013 | 0.019 | 0.49 | |
| *Intestinibacter* | rs447950 | A | G | 0.376 | 0.063 | 0.014 | 5.64E-06 | -0.023 | 0.020 | 0.25 | |
| *Intestinibacter* | rs9348442 | C | T | 0.126 | 0.099 | 0.022 | 6.26E-06 | -0.008 | 0.029 | 0.78 | |
| *Intestinibacter* | rs6062862 | A | G | 0.083 | 0.092 | 0.020 | 6.68E-06 | 0.030 | 0.035 | 0.38 | |
| *Intestinibacter* | rs2098844 | C | T | 0.378 | -0.058 | 0.013 | 6.79E-06 | 0.003 | 0.020 | 0.89 | |
| *Intestinibacter* | rs62430350 | T | C | 0.037 | 0.151 | 0.035 | 6.84E-06 | -0.149 | 0.053 | 0.01 | |
| *Intestinibacter* | rs893394 | G | A | 0.453 | 0.058 | 0.013 | 7.85E-06 | -0.016 | 0.019 | 0.42 | |
| *Intestinibacter* | rs68093214 | C | T | 0.241 | 0.066 | 0.015 | 9.26E-06 | -0.017 | 0.022 | 0.44 | |
| *Intestinimonas* | rs12226153 | A | G | 0.014 | -0.151 | 0.031 | 5.12E-07 | 0.033 | 0.084 | 0.69 | |
| *Intestinimonas* | rs11258178 | A | G | 0.491 | 0.066 | 0.013 | 6.98E-07 | 0.004 | 0.019 | 0.82 | |
| *Intestinimonas* | rs716604 | A | G | 0.230 | 0.082 | 0.017 | 8.57E-07 | 0.012 | 0.023 | 0.60 | |
| *Intestinimonas* | rs4784055 | T | C | 0.050 | -0.175 | 0.039 | 8.72E-07 | 0.033 | 0.044 | 0.45 | |
| *Intestinimonas* | rs2930225 | G | T | 0.233 | 0.073 | 0.015 | 1.35E-06 | 0.003 | 0.023 | 0.88 | |
| *Intestinimonas* | rs2731794 | C | T | 0.035 | 0.121 | 0.026 | 1.92E-06 | -0.052 | 0.053 | 0.32 | |
| *Intestinimonas* | rs10262702 | T | C | 0.125 | 0.092 | 0.019 | 2.06E-06 | -0.013 | 0.029 | 0.64 | |
| *Intestinimonas* | rs62240188 | G | A | 0.093 | 0.130 | 0.027 | 2.20E-06 | -0.009 | 0.033 | 0.79 | |
| *Intestinimonas* | rs7170984 | T | C | 0.282 | -0.066 | 0.014 | 2.98E-06 | 0.028 | 0.021 | 0.18 | |
| *Intestinimonas* | rs1859797 | G | A | 0.548 | 0.060 | 0.013 | 4.12E-06 | -0.033 | 0.019 | 0.09 | |
| *Intestinimonas* | rs72982915 | C | T | 0.057 | 0.183 | 0.040 | 4.91E-06 | 0.034 | 0.041 | 0.42 | |
| *Intestinimonas* | rs17067892 | C | T | 0.088 | 0.107 | 0.025 | 6.38E-06 | 0.013 | 0.034 | 0.70 | |
| *Intestinimonas* | rs4113676 | A | C | 0.015 | -0.219 | 0.049 | 7.42E-06 | 0.156 | 0.082 | 0.06 | |
| *Intestinimonas* | rs2276760 | A | G | 0.240 | -0.069 | 0.015 | 7.84E-06 | 0.007 | 0.022 | 0.76 | |
| *Intestinimonas* | rs6934519 | C | T | 0.256 | 0.069 | 0.015 | 8.57E-06 | -0.009 | 0.022 | 0.67 | |
| *Intestinimonas* | rs9823439 | T | C | 0.462 | -0.058 | 0.013 | 9.86E-06 | 0.015 | 0.019 | 0.44 | |
| *Lachnoclostridium* | rs6112314 | A | C | 0.332 | -0.056 | 0.011 | 2.43E-07 | 0.026 | 0.020 | 0.20 | |
| *Lachnoclostridium* | rs62285313 | A | G | 0.098 | 0.086 | 0.018 | 1.58E-06 | -0.023 | 0.033 | 0.48 | |
| *Lachnoclostridium* | rs615997 | T | C | 0.511 | 0.051 | 0.011 | 2.03E-06 | -0.033 | 0.019 | 0.09 | |
| *Lachnoclostridium* | rs78068103 | A | G | 0.117 | 0.089 | 0.019 | 3.67E-06 | -0.049 | 0.030 | 0.10 | |
| *Lachnoclostridium* | rs789029 | C | T | 0.141 | -0.064 | 0.014 | 3.75E-06 | -0.027 | 0.028 | 0.33 | |
| *Lachnoclostridium* | rs4738679 | G | A | 0.385 | -0.052 | 0.011 | 4.42E-06 | 0.024 | 0.020 | 0.22 | |
| *Lachnoclostridium* | rs72829893 | G | T | 0.103 | 0.117 | 0.027 | 5.58E-06 | 0.047 | 0.031 | 0.14 | |
| *Lachnoclostridium* | rs1997204 | T | C | 0.045 | -0.108 | 0.024 | 5.97E-06 | 0.027 | 0.046 | 0.56 | |
| *Lachnoclostridium* | rs1031599 | G | T | 0.064 | -0.079 | 0.018 | 6.31E-06 | 0.044 | 0.039 | 0.27 | |
| *Lachnoclostridium* | rs3821998 | C | A | 0.104 | -0.086 | 0.019 | 6.72E-06 | -0.034 | 0.031 | 0.27 | |
| *Lachnoclostridium* | rs2385421 | A | G | 0.120 | 0.075 | 0.018 | 7.14E-06 | 0.020 | 0.030 | 0.50 | |
| *Lachnoclostridium* | rs12566975 | T | C | 0.453 | -0.047 | 0.011 | 9.57E-06 | -0.002 | 0.019 | 0.90 | |
| *Lachnoclostridium* | rs1528479 | G | A | 0.375 | -0.050 | 0.011 | 9.64E-06 | -0.011 | 0.020 | 0.59 | |
| *Lachnospira* | rs13157098 | A | G | 0.163 | -0.077 | 0.016 | 5.99E-07 | 0.005 | 0.026 | 0.83 | |
| *Lachnospira* | rs4923324 | G | A | 0.161 | -0.062 | 0.013 | 2.44E-06 | 0.016 | 0.026 | 0.53 | |
| *Lachnospira* | rs4686798 | T | C | 0.362 | 0.053 | 0.011 | 2.74E-06 | -0.001 | 0.020 | 0.96 | |
| *Lachnospira* | rs56791201 | T | C | 0.362 | 0.052 | 0.011 | 2.93E-06 | 0.006 | 0.020 | 0.77 | |
| *Lachnospira* | rs159484 | G | A | 0.073 | 0.079 | 0.018 | 6.68E-06 | 0.091 | 0.037 | 0.01 | |
| *Lachnospira* | rs2520509 | A | G | 0.291 | 0.052 | 0.012 | 7.42E-06 | -0.011 | 0.021 | 0.62 | |
| *Lachnospiraceae (FCS020 group)* | rs7249113 | G | A | 0.300 | 0.068 | 0.013 | 3.72E-07 | -0.010 | 0.021 | 0.62 | |
| *Lachnospiraceae (FCS020 group)* | rs9788306 | C | T | 0.288 | -0.063 | 0.013 | 1.39E-06 | 0.016 | 0.021 | 0.46 | |
| *Lachnospiraceae (FCS020 group)* | rs1363769 | T | C | 0.032 | -0.201 | 0.045 | 1.58E-06 | 0.018 | 0.055 | 0.74 | |
| *Lachnospiraceae (FCS020 group)* | rs72793667 | A | G | 0.040 | -0.117 | 0.025 | 1.63E-06 | 0.088 | 0.049 | 0.07 | |
| *Lachnospiraceae (FCS020 group)* | rs35035870 | T | C | 0.040 | -0.191 | 0.041 | 2.62E-06 | -0.055 | 0.049 | 0.27 | |
| *Lachnospiraceae (FCS020 group)* | rs10093861 | G | A | 0.407 | -0.057 | 0.012 | 3.06E-06 | 0.001 | 0.019 | 0.95 | |
| *Lachnospiraceae (FCS020 group)* | rs2862811 | T | C | 0.301 | 0.056 | 0.012 | 3.92E-06 | 0.002 | 0.021 | 0.94 | |
| *Lachnospiraceae (FCS020 group)* | rs2322265 | C | T | 0.267 | -0.067 | 0.014 | 5.21E-06 | -0.002 | 0.022 | 0.93 | |
| *Lachnospiraceae (FCS020 group)* | rs1254846 | G | A | 0.145 | 0.106 | 0.023 | 5.60E-06 | 0.010 | 0.027 | 0.71 | |
| *Lachnospiraceae (FCS020 group)* | rs3999074 | G | T | 0.493 | -0.055 | 0.012 | 6.55E-06 | 0.011 | 0.019 | 0.56 | |
| *Lachnospiraceae (FCS020 group)* | rs9308097 | A | G | 0.494 | 0.055 | 0.012 | 7.47E-06 | -0.019 | 0.019 | 0.32 | |
| *Lachnospiraceae (FCS020 group)* | rs4452603 | T | G | 0.272 | 0.060 | 0.014 | 8.98E-06 | -0.033 | 0.022 | 0.13 | |
| *Lachnospiraceae (NC2004 group)* | rs6116753 | G | A | 0.178 | 0.099 | 0.021 | 2.92E-06 | 0.012 | 0.025 | 0.62 | |
| *Lachnospiraceae (NC2004 group)* | rs12127733 | G | A | 0.179 | 0.115 | 0.025 | 3.11E-06 | -0.034 | 0.025 | 0.18 | |
| *Lachnospiraceae (NC2004 group)* | rs3756315 | A | G | 0.297 | -0.088 | 0.019 | 3.33E-06 | -0.016 | 0.021 | 0.44 | |
| *Lachnospiraceae (NC2004 group)* | rs17067076 | G | A | 0.115 | -0.155 | 0.035 | 5.61E-06 | 0.056 | 0.030 | 0.06 | |
| *Lachnospiraceae (NC2004 group)* | rs12863463 | G | A | 0.077 | -0.156 | 0.035 | 6.04E-06 | -0.052 | 0.036 | 0.15 | |
| *Lachnospiraceae (NC2004 group)* | rs1928659 | T | C | 0.202 | 0.103 | 0.023 | 6.17E-06 | -0.007 | 0.024 | 0.78 | |
| *Lachnospiraceae (NC2004 group)* | rs1929743 | T | C | 0.336 | 0.084 | 0.019 | 9.06E-06 | 0.000 | 0.020 | 0.99 | |
| *Lachnospiraceae (NC2004 group)* | rs117467633 | T | C | 0.040 | -0.170 | 0.038 | 9.13E-06 | 0.005 | 0.050 | 0.92 | |
| *Lachnospiraceae (NC2004 group)* | rs12208226 | C | A | 0.107 | -0.155 | 0.034 | 9.75E-06 | -0.044 | 0.031 | 0.15 | |
| *Lachnospiraceae (ND3007 group)* | rs9932954 | A | G | 0.334 | -0.056 | 0.012 | 1.25E-06 | -0.003 | 0.020 | 0.89 | |
| *Lachnospiraceae (ND3007 group)* | rs2861203 | G | A | 0.302 | 0.057 | 0.013 | 7.37E-06 | 0.015 | 0.021 | 0.48 | |
| *Lachnospiraceae (ND3007 group)* | rs72776675 | T | C | 0.172 | -0.065 | 0.015 | 8.72E-06 | -0.041 | 0.025 | 0.10 | |
| *Lachnospiraceae (NK4A136 group)* | rs954878 | A | G | 0.373 | -0.052 | 0.011 | 1.78E-06 | 0.011 | 0.020 | 0.59 | |
| *Lachnospiraceae (NK4A136 group)* | rs160061 | A | G | 0.519 | 0.051 | 0.011 | 2.12E-06 | -0.016 | 0.019 | 0.41 | |
| *Lachnospiraceae (NK4A136 group)* | rs68104925 | T | C | 0.305 | -0.055 | 0.012 | 2.37E-06 | -0.024 | 0.021 | 0.24 | |
| *Lachnospiraceae (NK4A136 group)* | rs7616165 | G | T | 0.025 | -0.231 | 0.048 | 2.77E-06 | 0.132 | 0.062 | 0.03 | |
| *Lachnospiraceae (NK4A136 group)* | rs76193507 | A | G | 0.086 | -0.230 | 0.050 | 2.93E-06 | 0.087 | 0.033 | 0.01 | |
| *Lachnospiraceae (NK4A136 group)* | rs7832116 | A | G | 0.129 | -0.071 | 0.015 | 3.57E-06 | 0.018 | 0.029 | 0.52 | |
| *Lachnospiraceae (NK4A136 group)* | rs73044693 | A | G | 0.071 | -0.108 | 0.023 | 3.57E-06 | 0.045 | 0.038 | 0.24 | |
| *Lachnospiraceae (NK4A136 group)* | rs11263806 | A | G | 0.343 | -0.052 | 0.012 | 5.07E-06 | 0.035 | 0.020 | 0.08 | |
| *Lachnospiraceae (NK4A136 group)* | rs7073658 | T | G | 0.485 | -0.050 | 0.011 | 5.27E-06 | 0.041 | 0.019 | 0.03 | |
| *Lachnospiraceae (NK4A136 group)* | rs2880566 | T | C | 0.148 | 0.060 | 0.013 | 5.61E-06 | 0.000 | 0.027 | 0.99 | |
| *Lachnospiraceae (NK4A136 group)* | rs12611395 | A | G | 0.106 | -0.090 | 0.020 | 5.83E-06 | -0.052 | 0.031 | 0.10 | |
| *Lachnospiraceae (NK4A136 group)* | rs4955932 | T | C | 0.388 | -0.049 | 0.011 | 7.05E-06 | -0.009 | 0.020 | 0.64 | |
| *Lachnospiraceae (NK4A136 group)* | rs10952110 | G | T | 0.445 | 0.049 | 0.011 | 9.08E-06 | -0.004 | 0.019 | 0.83 | |
| *Lachnospiraceae (NK4A136 group)* | rs28540839 | A | C | 0.475 | 0.051 | 0.011 | 9.34E-06 | -0.015 | 0.019 | 0.44 | |
| *Lachnospiraceae (NK4A136 group)* | rs59805249 | T | C | 0.093 | 0.094 | 0.021 | 9.45E-06 | -0.008 | 0.033 | 0.80 | |
| *Lachnospiraceae (UCG001)* | rs437876 | T | C | 0.355 | 0.078 | 0.014 | 7.17E-08 | -0.006 | 0.020 | 0.75 | |
| *Lachnospiraceae (UCG001)* | rs985416 | C | T | 0.180 | 0.097 | 0.018 | 1.46E-07 | 0.089 | 0.024 | 0.00 | |
| *Lachnospiraceae (UCG001)* | rs2050911 | G | A | 0.337 | 0.075 | 0.015 | 1.11E-06 | -0.015 | 0.020 | 0.47 | |
| *Lachnospiraceae (UCG001)* | rs573933 | T | C | 0.107 | -0.108 | 0.023 | 3.11E-06 | 0.034 | 0.031 | 0.27 | |
| *Lachnospiraceae (UCG001)* | rs74034332 | G | A | 0.062 | 0.168 | 0.038 | 3.33E-06 | -0.004 | 0.040 | 0.93 | |
| *Lachnospiraceae (UCG001)* | rs78848836 | A | G | 0.104 | -0.119 | 0.026 | 3.38E-06 | -0.009 | 0.031 | 0.78 | |
| *Lachnospiraceae (UCG001)* | rs9403580 | C | T | 0.130 | 0.108 | 0.023 | 3.47E-06 | 0.010 | 0.028 | 0.72 | |
| *Lachnospiraceae (UCG001)* | rs62496417 | T | G | 0.215 | -0.075 | 0.017 | 5.88E-06 | -0.031 | 0.023 | 0.18 | |
| *Lachnospiraceae (UCG001)* | rs4981345 | T | C | 0.329 | -0.068 | 0.015 | 6.09E-06 | -0.030 | 0.020 | 0.15 | |
| *Lachnospiraceae (UCG001)* | rs12131224 | C | T | 0.110 | 0.117 | 0.026 | 7.40E-06 | -0.016 | 0.031 | 0.59 | |
| *Lachnospiraceae (UCG001)* | rs2371284 | T | C | 0.231 | -0.076 | 0.017 | 7.56E-06 | -0.003 | 0.023 | 0.91 | |
| *Lachnospiraceae (UCG001)* | rs7341608 | T | C | 0.135 | -0.078 | 0.018 | 9.48E-06 | -0.027 | 0.028 | 0.34 | |
| *Lachnospiraceae (UCG004)* | rs12747809 | G | A | 0.282 | -0.062 | 0.013 | 8.65E-07 | 0.008 | 0.021 | 0.70 | |
| *Lachnospiraceae (UCG004)* | rs2882478 | G | A | 0.536 | -0.058 | 0.012 | 1.21E-06 | -0.020 | 0.019 | 0.29 | |
| *Lachnospiraceae (UCG004)* | rs12673420 | G | A | 0.499 | 0.055 | 0.012 | 2.98E-06 | 0.015 | 0.019 | 0.44 | |
| *Lachnospiraceae (UCG004)* | rs12894272 | A | G | 0.337 | 0.058 | 0.013 | 4.34E-06 | -0.023 | 0.020 | 0.25 | |
| *Lachnospiraceae (UCG004)* | rs11128180 | A | G | 0.232 | 0.065 | 0.014 | 4.52E-06 | 0.001 | 0.023 | 0.97 | |
| *Lachnospiraceae (UCG004)* | rs2444793 | C | T | 0.386 | -0.054 | 0.012 | 4.77E-06 | 0.000 | 0.020 | 0.98 | |
| *Lachnospiraceae (UCG004)* | rs35182105 | A | G | 0.055 | -0.110 | 0.024 | 4.87E-06 | -0.040 | 0.042 | 0.35 | |
| *Lachnospiraceae (UCG004)* | rs6656451 | C | T | 0.470 | -0.054 | 0.012 | 5.57E-06 | 0.010 | 0.019 | 0.58 | |
| *Lachnospiraceae (UCG004)* | rs7629954 | A | G | 0.045 | 0.108 | 0.024 | 5.77E-06 | -0.021 | 0.047 | 0.66 | |
| *Lachnospiraceae (UCG004)* | rs233486 | A | G | 0.139 | -0.080 | 0.018 | 6.28E-06 | -0.035 | 0.028 | 0.21 | |
| *Lachnospiraceae (UCG004)* | rs2726805 | A | G | 0.470 | 0.055 | 0.012 | 6.30E-06 | 0.000 | 0.019 | 0.99 | |
| *Lachnospiraceae (UCG004)* | rs12072562 | T | C | 0.039 | 0.133 | 0.030 | 7.07E-06 | -0.018 | 0.050 | 0.72 | |
| *Lachnospiraceae (UCG008)* | rs10793103 | C | T | 0.450 | 0.097 | 0.018 | 9.35E-08 | -0.001 | 0.019 | 0.94 | |
| *Lachnospiraceae (UCG008)* | rs67078837 | T | C | 0.417 | -0.085 | 0.017 | 7.68E-07 | 0.001 | 0.019 | 0.97 | |
| *Lachnospiraceae (UCG008)* | rs10741777 | T | C | 0.309 | -0.097 | 0.019 | 7.69E-07 | 0.016 | 0.021 | 0.45 | |
| *Lachnospiraceae (UCG008)* | rs10801803 | G | A | 0.142 | -0.117 | 0.024 | 1.40E-06 | -0.037 | 0.027 | 0.18 | |
| *Lachnospiraceae (UCG008)* | rs62277846 | C | T | 0.191 | 0.102 | 0.021 | 1.59E-06 | 0.001 | 0.024 | 0.96 | |
| *Lachnospiraceae (UCG008)* | rs955844 | A | C | 0.141 | 0.112 | 0.023 | 1.81E-06 | -0.003 | 0.028 | 0.93 | |
| *Lachnospiraceae (UCG008)* | rs13024781 | T | C | 0.520 | -0.080 | 0.017 | 2.29E-06 | 0.010 | 0.019 | 0.59 | |
| *Lachnospiraceae (UCG008)* | rs57091572 | A | G | 0.132 | -0.110 | 0.024 | 2.86E-06 | -0.001 | 0.028 | 0.96 | |
| *Lachnospiraceae (UCG008)* | rs61944774 | A | G | 0.053 | 0.180 | 0.039 | 6.34E-06 | 0.001 | 0.043 | 0.98 | |
| *Lachnospiraceae (UCG008)* | rs75356640 | G | A | 0.125 | 0.137 | 0.030 | 9.83E-06 | -0.063 | 0.029 | 0.03 | |
| *Lachnospiraceae (UCG010)* | rs11192447 | A | G | 0.050 | 0.127 | 0.024 | 4.69E-07 | -0.028 | 0.044 | 0.53 | |
| *Lachnospiraceae (UCG010)* | rs9981767 | A | C | 0.258 | 0.066 | 0.013 | 9.96E-07 | 0.017 | 0.022 | 0.45 | |
| *Lachnospiraceae (UCG010)* | rs12346653 | C | T | 0.209 | 0.066 | 0.014 | 2.70E-06 | 0.006 | 0.024 | 0.79 | |
| *Lachnospiraceae (UCG010)* | rs74315802 | G | T | 0.184 | 0.087 | 0.018 | 3.19E-06 | -0.057 | 0.025 | 0.02 | |
| *Lachnospiraceae (UCG010)* | rs10414815 | T | C | 0.047 | 0.105 | 0.023 | 4.24E-06 | -0.044 | 0.046 | 0.34 | |
| *Lachnospiraceae (UCG010)* | rs72894957 | G | A | 0.023 | 0.222 | 0.049 | 5.68E-06 | -0.004 | 0.064 | 0.94 | |
| *Lachnospiraceae (UCG010)* | rs336138 | G | T | 0.115 | 0.078 | 0.017 | 7.48E-06 | -0.021 | 0.030 | 0.49 | |
| *Lachnospiraceae (UCG010)* | rs4576377 | A | C | 0.366 | -0.057 | 0.013 | 7.63E-06 | 0.004 | 0.020 | 0.83 | |
| *Lachnospiraceae (UCG010)* | rs17730011 | G | A | 0.215 | -0.070 | 0.016 | 7.85E-06 | 0.001 | 0.023 | 0.97 | |
| *Lachnospiraceae (UCG010)* | rs2833528 | C | T | 0.373 | -0.056 | 0.013 | 9.92E-06 | -0.007 | 0.020 | 0.73 | |
| *Lactobacillus* | rs921925 | A | C | 0.213 | 0.099 | 0.020 | 9.72E-07 | -0.026 | 0.023 | 0.27 | |
| *Lactobacillus* | rs16861661 | G | A | 0.066 | -0.183 | 0.038 | 1.28E-06 | -0.024 | 0.039 | 0.53 | |
| *Lactobacillus* | rs62314653 | C | A | 0.060 | 0.188 | 0.039 | 2.24E-06 | 0.001 | 0.041 | 0.98 | |
| *Lactobacillus* | rs7399658 | G | A | 0.183 | -0.107 | 0.022 | 3.12E-06 | -0.012 | 0.025 | 0.62 | |
| *Lactobacillus* | rs768253 | T | G | 0.439 | -0.079 | 0.017 | 4.25E-06 | 0.024 | 0.019 | 0.21 | |
| *Lactobacillus* | rs1530559 | G | A | 0.463 | 0.080 | 0.018 | 4.93E-06 | -0.035 | 0.019 | 0.07 | |
| *Lactobacillus* | rs77478751 | A | G | 0.119 | -0.220 | 0.048 | 7.33E-06 | -0.002 | 0.030 | 0.95 | |
| *Lactobacillus* | rs12693845 | C | T | 0.410 | -0.081 | 0.018 | 8.96E-06 | 0.002 | 0.020 | 0.92 | |
| *Lactococcus* | rs123059 | T | C | 0.218 | -0.137 | 0.027 | 1.27E-06 | -0.010 | 0.023 | 0.68 | |
| *Lactococcus* | rs10417872 | T | G | 0.286 | 0.118 | 0.025 | 1.29E-06 | -0.009 | 0.021 | 0.66 | |
| *Lactococcus* | rs2293361 | C | T | 0.054 | -0.199 | 0.043 | 1.40E-06 | -0.015 | 0.042 | 0.72 | |
| *Lactococcus* | rs4766997 | C | T | 0.426 | 0.115 | 0.024 | 2.06E-06 | -0.017 | 0.019 | 0.37 | |
| *Lactococcus* | rs55910161 | C | T | 0.113 | 0.146 | 0.031 | 2.36E-06 | -0.014 | 0.031 | 0.64 | |
| *Lactococcus* | rs7992246 | T | C | 0.390 | 0.104 | 0.023 | 4.45E-06 | 0.064 | 0.019 | 0.00 | |
| *Lactococcus* | rs6674304 | C | T | 0.038 | 0.201 | 0.044 | 6.18E-06 | -0.020 | 0.051 | 0.70 | |
| *Lactococcus* | rs17168302 | G | A | 0.107 | 0.192 | 0.042 | 6.29E-06 | 0.022 | 0.031 | 0.47 | |
| *Lactococcus* | rs12621813 | G | A | 0.265 | 0.108 | 0.024 | 6.61E-06 | -0.035 | 0.022 | 0.10 | |
| *Marvinbryantia* | rs2724813 | A | G | 0.237 | -0.084 | 0.017 | 6.28E-07 | -0.034 | 0.023 | 0.13 | |
| *Marvinbryantia* | rs2842896 | C | T | 0.587 | -0.065 | 0.013 | 7.25E-07 | 0.001 | 0.020 | 0.95 | |
| *Marvinbryantia* | rs61884471 | G | A | 0.110 | 0.124 | 0.025 | 1.01E-06 | -0.058 | 0.031 | 0.06 | |
| *Marvinbryantia* | rs1187983 | C | T | 0.104 | -0.094 | 0.019 | 2.02E-06 | 0.014 | 0.032 | 0.65 | |
| *Marvinbryantia* | rs2863363 | A | G | 0.244 | 0.063 | 0.014 | 3.11E-06 | -0.001 | 0.022 | 0.98 | |
| *Marvinbryantia* | rs72948274 | A | C | 0.064 | -0.126 | 0.027 | 3.26E-06 | -0.051 | 0.039 | 0.19 | |
| *Marvinbryantia* | rs3125832 | A | C | 0.226 | 0.068 | 0.015 | 5.03E-06 | -0.028 | 0.023 | 0.22 | |
| *Marvinbryantia* | rs8006832 | G | T | 0.092 | -0.095 | 0.022 | 6.58E-06 | 0.009 | 0.033 | 0.79 | |
| *Marvinbryantia* | rs146541147 | G | A | 0.033 | 0.119 | 0.027 | 6.86E-06 | 0.005 | 0.054 | 0.92 | |
| *Marvinbryantia* | rs11620597 | T | C | 0.024 | 0.119 | 0.027 | 7.80E-06 | 0.049 | 0.063 | 0.44 | |
| *Methanobrevibacter* | rs76029318 | T | C | 0.063 | 0.223 | 0.045 | 1.08E-06 | -0.017 | 0.040 | 0.66 | |
| *Methanobrevibacter* | rs10202904 | T | G | 0.409 | -0.113 | 0.024 | 3.09E-06 | 0.011 | 0.020 | 0.57 | |
| *Methanobrevibacter* | rs894996 | C | A | 0.070 | 0.214 | 0.046 | 3.82E-06 | -0.037 | 0.038 | 0.32 | |
| *Methanobrevibacter* | rs1334944 | T | C | 0.278 | 0.115 | 0.026 | 7.61E-06 | -0.019 | 0.021 | 0.38 | |
| *Methanobrevibacter* | rs6776814 | T | C | 0.021 | -0.189 | 0.042 | 8.05E-06 | -0.099 | 0.068 | 0.15 | |
| *Methanobrevibacter* | rs4802933 | A | G | 0.232 | -0.136 | 0.031 | 9.74E-06 | 0.054 | 0.023 | 0.02 | |
| *Odoribacter* | rs77779484 | G | A | 0.061 | -0.133 | 0.027 | 6.56E-07 | -0.025 | 0.040 | 0.53 | |
| *Odoribacter* | rs10093869 | A | G | 0.423 | -0.058 | 0.013 | 3.67E-06 | 0.010 | 0.019 | 0.61 | |
| *Odoribacter* | rs28417404 | A | G | 0.097 | -0.073 | 0.016 | 3.68E-06 | -0.036 | 0.032 | 0.27 | |
| *Odoribacter* | rs4793970 | A | G | 0.364 | -0.058 | 0.013 | 6.03E-06 | -0.028 | 0.020 | 0.15 | |
| *Odoribacter* | rs6856150 | G | A | 0.123 | 0.088 | 0.019 | 6.06E-06 | -0.023 | 0.029 | 0.42 | |
| *Odoribacter* | rs10423795 | C | T | 0.383 | 0.055 | 0.012 | 6.58E-06 | 0.016 | 0.020 | 0.43 | |
| *Odoribacter* | rs74553962 | T | G | 0.076 | 0.121 | 0.026 | 9.49E-06 | 0.055 | 0.037 | 0.13 | |
| *Olsenella* | rs62112538 | C | T | 0.111 | -0.199 | 0.041 | 1.19E-06 | -0.098 | 0.031 | 0.00 | |
| *Olsenella* | rs17148768 | G | A | 0.167 | 0.140 | 0.030 | 2.20E-06 | 0.010 | 0.026 | 0.69 | |
| *Olsenella* | rs72691585 | C | A | 0.135 | -0.249 | 0.052 | 2.95E-06 | 0.024 | 0.028 | 0.39 | |
| *Olsenella* | rs2759329 | G | A | 0.369 | -0.111 | 0.024 | 3.43E-06 | 0.011 | 0.020 | 0.59 | |
| *Olsenella* | rs35225860 | A | G | 0.039 | -0.224 | 0.048 | 3.87E-06 | 0.009 | 0.050 | 0.86 | |
| *Olsenella* | rs1035588 | A | G | 0.373 | -0.108 | 0.024 | 4.86E-06 | -0.008 | 0.020 | 0.69 | |
| *Olsenella* | rs7540303 | C | T | 0.365 | 0.108 | 0.024 | 5.32E-06 | 0.039 | 0.020 | 0.05 | |
| *Olsenella* | rs61090148 | A | G | 0.431 | -0.105 | 0.023 | 6.44E-06 | -0.028 | 0.019 | 0.15 | |
| *Olsenella* | rs9460691 | C | A | 0.195 | 0.120 | 0.027 | 7.28E-06 | -0.003 | 0.024 | 0.89 | |
| *Olsenella* | rs8066522 | G | A | 0.331 | -0.107 | 0.024 | 9.70E-06 | -0.017 | 0.020 | 0.42 | |
| *Oscillibacter* | rs234108 | A | G | 0.396 | 0.075 | 0.015 | 9.16E-07 | -0.013 | 0.019 | 0.49 | |
| *Oscillibacter* | rs9393920 | A | G | 0.378 | -0.074 | 0.015 | 9.92E-07 | -0.024 | 0.020 | 0.22 | |
| *Oscillibacter* | rs11627628 | T | C | 0.076 | 0.144 | 0.029 | 1.01E-06 | 0.012 | 0.036 | 0.74 | |
| *Oscillibacter* | rs133832 | A | C | 0.282 | -0.080 | 0.016 | 1.15E-06 | 0.003 | 0.021 | 0.90 | |
| *Oscillibacter* | rs36095275 | C | T | 0.418 | -0.075 | 0.016 | 1.40E-06 | -0.011 | 0.019 | 0.56 | |
| *Oscillibacter* | rs761240 | T | G | 0.051 | -0.177 | 0.039 | 2.04E-06 | 0.077 | 0.045 | 0.09 | |
| *Oscillibacter* | rs16866406 | A | G | 0.153 | 0.099 | 0.021 | 3.08E-06 | -0.048 | 0.026 | 0.07 | |
| *Oscillibacter* | rs4506202 | A | G | 0.532 | -0.071 | 0.015 | 3.21E-06 | 0.006 | 0.019 | 0.77 | |
| *Oscillibacter* | rs61883564 | A | G | 0.136 | -0.101 | 0.022 | 3.39E-06 | -0.006 | 0.028 | 0.83 | |
| *Oscillibacter* | rs12649930 | T | G | 0.101 | 0.122 | 0.026 | 4.09E-06 | -0.009 | 0.032 | 0.77 | |
| *Oscillibacter* | rs16934185 | A | G | 0.103 | -0.130 | 0.028 | 4.38E-06 | -0.040 | 0.031 | 0.20 | |
| *Oscillibacter* | rs11990279 | T | C | 0.200 | -0.082 | 0.018 | 4.94E-06 | -0.007 | 0.024 | 0.78 | |
| *Oscillibacter* | rs75453768 | G | T | 0.098 | 0.122 | 0.027 | 5.35E-06 | 0.056 | 0.032 | 0.08 | |
| *Oscillospira* | rs12206468 | G | A | 0.078 | -0.133 | 0.027 | 1.04E-06 | -0.018 | 0.036 | 0.62 | |
| *Oscillospira* | rs1954532 | T | C | 0.199 | -0.083 | 0.018 | 2.27E-06 | -0.021 | 0.024 | 0.38 | |
| *Oscillospira* | rs28889936 | A | C | 0.096 | 0.114 | 0.025 | 3.37E-06 | -0.018 | 0.032 | 0.57 | |
| *Oscillospira* | rs8076323 | A | G | 0.326 | 0.072 | 0.016 | 5.61E-06 | 0.013 | 0.020 | 0.53 | |
| *Oscillospira* | rs72866977 | A | C | 0.080 | -0.131 | 0.028 | 5.63E-06 | -0.009 | 0.036 | 0.80 | |
| *Oscillospira* | rs62422654 | C | T | 0.208 | 0.090 | 0.020 | 6.47E-06 | -0.033 | 0.023 | 0.16 | |
| *Oscillospira* | rs751183 | T | C | 0.182 | -0.077 | 0.017 | 6.85E-06 | 0.000 | 0.025 | 0.99 | |
| *Oscillospira* | rs12925026 | T | C | 0.056 | 0.136 | 0.031 | 9.31E-06 | 0.041 | 0.041 | 0.32 | |
| *Oxalobacter* | rs736744 | C | T | 0.432 | 0.118 | 0.021 | 2.57E-08 | 0.070 | 0.019 | 0.00 | |
| *Oxalobacter* | rs4428215 | G | A | 0.253 | 0.130 | 0.024 | 7.51E-08 | -0.012 | 0.022 | 0.59 | |
| *Oxalobacter* | rs6000536 | C | T | 0.170 | -0.131 | 0.025 | 2.06E-07 | -0.012 | 0.026 | 0.63 | |
| *Oxalobacter* | rs36057338 | G | T | 0.033 | 0.208 | 0.042 | 8.80E-07 | 0.037 | 0.053 | 0.49 | |
| *Oxalobacter* | rs12002250 | A | C | 0.043 | 0.217 | 0.047 | 1.42E-06 | -0.056 | 0.048 | 0.24 | |
| *Oxalobacter* | rs10464997 | G | A | 0.185 | 0.138 | 0.029 | 3.30E-06 | -0.012 | 0.025 | 0.63 | |
| *Oxalobacter* | rs1569853 | T | C | 0.120 | -0.138 | 0.030 | 3.65E-06 | -0.005 | 0.029 | 0.86 | |
| *Oxalobacter* | rs11108500 | A | G | 0.088 | -0.199 | 0.043 | 3.74E-06 | 0.066 | 0.034 | 0.05 | |
| *Oxalobacter* | rs6993398 | G | A | 0.184 | 0.127 | 0.028 | 7.13E-06 | 0.026 | 0.025 | 0.29 | |
| *Oxalobacter* | rs111966731 | T | C | 0.086 | 0.213 | 0.047 | 7.30E-06 | 0.038 | 0.034 | 0.26 | |
| *Oxalobacter* | rs3862635 | C | T | 0.091 | -0.172 | 0.039 | 9.19E-06 | 0.015 | 0.033 | 0.64 | |
| *Parabacteroides* | rs60884758 | C | T | 0.178 | -0.070 | 0.014 | 5.71E-07 | 0.023 | 0.025 | 0.36 | |
| *Parabacteroides* | rs115602804 | G | A | 0.107 | 0.103 | 0.022 | 1.93E-06 | -0.015 | 0.031 | 0.63 | |
| *Parabacteroides* | rs4236095 | G | A | 0.103 | 0.076 | 0.016 | 1.93E-06 | 0.027 | 0.032 | 0.39 | |
| *Parabacteroides* | rs7298818 | C | T | 0.102 | 0.089 | 0.020 | 8.54E-06 | -0.029 | 0.031 | 0.35 | |
| *Parabacteroides* | rs6657302 | T | C | 0.063 | -0.105 | 0.023 | 9.76E-06 | -0.010 | 0.039 | 0.79 | |
| *Paraprevotella* | rs2081023 | A | G | 0.142 | -0.123 | 0.024 | 2.64E-07 | 0.006 | 0.028 | 0.82 | |
| *Paraprevotella* | rs9602779 | A | C | 0.242 | -0.107 | 0.022 | 6.93E-07 | -0.028 | 0.022 | 0.21 | |
| *Paraprevotella* | rs9900242 | A | G | 0.343 | -0.085 | 0.018 | 1.14E-06 | 0.008 | 0.020 | 0.70 | |
| *Paraprevotella* | rs17785622 | A | G | 0.039 | 0.248 | 0.052 | 1.93E-06 | 0.001 | 0.050 | 0.99 | |
| *Paraprevotella* | rs140997932 | T | C | 0.055 | -0.162 | 0.035 | 2.11E-06 | 0.052 | 0.042 | 0.22 | |
| *Paraprevotella* | rs4767113 | C | T | 0.328 | 0.088 | 0.018 | 2.14E-06 | -0.002 | 0.020 | 0.92 | |
| *Paraprevotella* | rs4756632 | G | T | 0.130 | -0.139 | 0.029 | 3.82E-06 | -0.051 | 0.028 | 0.07 | |
| *Paraprevotella* | rs145020347 | A | G | 0.148 | -0.125 | 0.026 | 4.03E-06 | 0.021 | 0.027 | 0.44 | |
| *Paraprevotella* | rs3008582 | T | C | 0.194 | 0.106 | 0.023 | 4.36E-06 | -0.056 | 0.024 | 0.02 | |
| *Paraprevotella* | rs3801748 | G | A | 0.362 | 0.078 | 0.017 | 5.20E-06 | 0.033 | 0.020 | 0.10 | |
| *Paraprevotella* | rs7240324 | T | G | 0.246 | -0.102 | 0.023 | 5.96E-06 | -0.004 | 0.022 | 0.85 | |
| *Paraprevotella* | rs10842464 | T | C | 0.302 | -0.076 | 0.017 | 6.60E-06 | -0.034 | 0.021 | 0.10 | |
| *Paraprevotella* | rs17109926 | A | G | 0.271 | -0.099 | 0.022 | 6.75E-06 | 0.000 | 0.021 | 0.98 | |
| *Parasutterella* | rs7572229 | G | A | 0.522 | 0.066 | 0.013 | 6.32E-07 | 0.017 | 0.019 | 0.38 | |
| *Parasutterella* | rs10899911 | A | G | 0.235 | -0.072 | 0.015 | 1.15E-06 | 0.006 | 0.023 | 0.79 | |
| *Parasutterella* | rs7303158 | C | T | 0.448 | 0.065 | 0.013 | 1.33E-06 | 0.018 | 0.019 | 0.36 | |
| *Parasutterella* | rs78383039 | T | C | 0.040 | -0.146 | 0.030 | 1.57E-06 | 0.042 | 0.048 | 0.39 | |
| *Parasutterella* | rs6828768 | C | T | 0.468 | 0.064 | 0.013 | 1.78E-06 | 0.030 | 0.019 | 0.12 | |
| *Parasutterella* | rs55877868 | A | C | 0.100 | -0.104 | 0.023 | 2.87E-06 | -0.013 | 0.032 | 0.67 | |
| *Parasutterella* | rs2090816 | A | C | 0.186 | 0.084 | 0.018 | 2.90E-06 | 0.004 | 0.025 | 0.88 | |
| *Parasutterella* | rs35055552 | T | C | 0.140 | 0.110 | 0.024 | 3.35E-06 | -0.036 | 0.028 | 0.19 | |
| *Parasutterella* | rs8039785 | T | G | 0.512 | 0.062 | 0.013 | 3.62E-06 | -0.019 | 0.019 | 0.31 | |
| *Parasutterella* | rs823424 | G | A | 0.254 | -0.071 | 0.016 | 4.95E-06 | -0.010 | 0.022 | 0.66 | |
| *Parasutterella* | rs62273907 | A | G | 0.067 | 0.229 | 0.050 | 5.88E-06 | 0.008 | 0.038 | 0.84 | |
| *Parasutterella* | rs7311004 | T | C | 0.444 | -0.062 | 0.014 | 5.92E-06 | 0.025 | 0.019 | 0.20 | |
| *Parasutterella* | rs11715853 | G | A | 0.295 | -0.066 | 0.015 | 6.23E-06 | -0.049 | 0.021 | 0.02 | |
| *Parasutterella* | rs6809952 | G | A | 0.278 | -0.068 | 0.015 | 8.13E-06 | 0.011 | 0.022 | 0.62 | |
| *Peptococcus* | rs10031059 | T | C | 0.236 | -0.121 | 0.023 | 1.24E-07 | -0.015 | 0.022 | 0.51 | |
| *Peptococcus* | rs77681628 | C | T | 0.081 | 0.200 | 0.039 | 2.69E-07 | 0.012 | 0.035 | 0.72 | |
| *Peptococcus* | rs6918730 | G | A | 0.060 | 0.135 | 0.029 | 1.15E-06 | 0.013 | 0.041 | 0.74 | |
| *Peptococcus* | rs11001941 | G | A | 0.087 | -0.196 | 0.039 | 1.33E-06 | -0.009 | 0.034 | 0.79 | |
| *Peptococcus* | rs2054133 | G | A | 0.364 | 0.090 | 0.019 | 2.14E-06 | 0.005 | 0.020 | 0.82 | |
| *Peptococcus* | rs7033353 | T | G | 0.524 | 0.090 | 0.019 | 2.22E-06 | 0.013 | 0.019 | 0.50 | |
| *Peptococcus* | rs5770862 | T | C | 0.092 | 0.162 | 0.036 | 3.22E-06 | 0.010 | 0.033 | 0.76 | |
| *Peptococcus* | rs413827 | G | A | 0.240 | 0.110 | 0.024 | 3.30E-06 | -0.041 | 0.022 | 0.06 | |
| *Peptococcus* | rs72850165 | T | C | 0.077 | -0.134 | 0.030 | 5.74E-06 | -0.020 | 0.036 | 0.58 | |
| *Peptococcus* | rs36121075 | A | G | 0.172 | -0.141 | 0.031 | 6.99E-06 | 0.011 | 0.025 | 0.67 | |
| *Peptococcus* | rs74592222 | G | A | 0.116 | 0.138 | 0.030 | 8.55E-06 | 0.011 | 0.030 | 0.70 | |
| *Peptococcus* | rs12069354 | C | T | 0.062 | 0.168 | 0.038 | 9.28E-06 | -0.046 | 0.040 | 0.25 | |
| *Phascolarctobacterium* | rs75882962 | T | C | 0.126 | 0.097 | 0.019 | 3.19E-07 | 0.021 | 0.029 | 0.46 | |
| *Phascolarctobacterium* | rs56157888 | A | C | 0.221 | 0.095 | 0.019 | 1.09E-06 | 0.005 | 0.023 | 0.81 | |
| *Phascolarctobacterium* | rs56069061 | G | A | 0.067 | -0.111 | 0.023 | 1.87E-06 | -0.049 | 0.039 | 0.20 | |
| *Phascolarctobacterium* | rs12618201 | A | G | 0.442 | 0.064 | 0.014 | 3.38E-06 | -0.027 | 0.019 | 0.16 | |
| *Phascolarctobacterium* | rs74540770 | G | A | 0.080 | -0.121 | 0.026 | 3.60E-06 | -0.006 | 0.035 | 0.87 | |
| *Phascolarctobacterium* | rs1264476 | T | G | 0.187 | 0.077 | 0.017 | 4.30E-06 | -0.001 | 0.025 | 0.98 | |
| *Phascolarctobacterium* | rs28525131 | G | A | 0.048 | -0.119 | 0.027 | 8.23E-06 | -0.052 | 0.045 | 0.24 | |
| *Phascolarctobacterium* | rs7982713 | G | A | 0.285 | 0.073 | 0.016 | 9.72E-06 | -0.022 | 0.021 | 0.31 | |
| *Prevotella 7* | rs57404562 | C | A | 0.128 | 0.155 | 0.032 | 6.22E-07 | 0.018 | 0.029 | 0.53 | |
| *Prevotella 7* | rs9959718 | G | A | 0.203 | 0.133 | 0.028 | 1.90E-06 | 0.010 | 0.024 | 0.68 | |
| *Prevotella 7* | rs9608249 | A | G | 0.112 | -0.158 | 0.034 | 2.07E-06 | 0.015 | 0.031 | 0.63 | |
| *Prevotella 7* | rs430270 | A | C | 0.192 | 0.139 | 0.030 | 2.87E-06 | -0.014 | 0.024 | 0.55 | |
| *Prevotella 7* | rs2240542 | C | T | 0.266 | 0.121 | 0.026 | 4.84E-06 | -0.011 | 0.022 | 0.61 | |
| *Prevotella 7* | rs2918132 | C | T | 0.374 | -0.115 | 0.025 | 6.42E-06 | 0.010 | 0.020 | 0.61 | |
| *Prevotella 7* | rs79263163 | A | C | 0.202 | -0.144 | 0.032 | 7.51E-06 | -0.003 | 0.024 | 0.90 | |
| *Prevotella 7* | rs118038478 | A | G | 0.069 | 0.206 | 0.047 | 7.85E-06 | -0.019 | 0.038 | 0.61 | |
| *Prevotella 7* | rs12195431 | T | C | 0.098 | 0.197 | 0.044 | 8.73E-06 | -0.021 | 0.032 | 0.52 | |
| *Prevotella 7* | rs12124567 | A | G | 0.204 | -0.121 | 0.028 | 9.49E-06 | 0.024 | 0.024 | 0.33 | |
| *Prevotella 7* | rs9426434 | T | C | 0.350 | -0.124 | 0.028 | 9.72E-06 | 0.011 | 0.020 | 0.60 | |
| *Prevotella 9* | rs111509883 | T | C | 0.110 | 0.171 | 0.035 | 1.24E-06 | 0.021 | 0.031 | 0.48 | |
| *Prevotella 9* | rs2683313 | A | G | 0.314 | -0.072 | 0.015 | 1.69E-06 | -0.026 | 0.021 | 0.20 | |
| *Prevotella 9* | rs11685699 | C | T | 0.077 | -0.141 | 0.030 | 2.03E-06 | -0.040 | 0.036 | 0.26 | |
| *Prevotella 9* | rs746764 | T | C | 0.215 | -0.092 | 0.019 | 2.04E-06 | -0.041 | 0.023 | 0.07 | |
| *Prevotella 9* | rs117271932 | A | G | 0.056 | 0.208 | 0.044 | 2.82E-06 | -0.028 | 0.041 | 0.51 | |
| *Prevotella 9* | rs9428102 | A | G | 0.216 | -0.078 | 0.018 | 4.62E-06 | -0.016 | 0.023 | 0.49 | |
| *Prevotella 9* | rs1304512 | G | A | 0.273 | 0.076 | 0.017 | 5.29E-06 | -0.001 | 0.022 | 0.97 | |
| *Prevotella 9* | rs9613013 | G | A | 0.122 | 0.092 | 0.020 | 6.10E-06 | 0.046 | 0.029 | 0.12 | |
| *Prevotella 9* | rs7976209 | T | C | 0.155 | -0.087 | 0.020 | 7.28E-06 | 0.016 | 0.026 | 0.54 | |
| *Prevotella 9* | rs12648235 | T | C | 0.235 | 0.079 | 0.018 | 7.39E-06 | -0.022 | 0.023 | 0.32 | |
| *Prevotella 9* | rs2104588 | T | C | 0.056 | 0.106 | 0.024 | 8.13E-06 | 0.042 | 0.042 | 0.31 | |
| *Prevotella 9* | rs4968431 | G | T | 0.367 | 0.064 | 0.014 | 8.58E-06 | 0.026 | 0.020 | 0.19 | |
| *Prevotella 9* | rs72815774 | T | C | 0.060 | -0.176 | 0.039 | 8.78E-06 | 0.007 | 0.044 | 0.88 | |
| *Prevotella 9* | rs7237249 | C | T | 0.189 | -0.082 | 0.018 | 8.93E-06 | 0.008 | 0.024 | 0.73 | |
| *Prevotella 9* | rs2495052 | A | G | 0.147 | 0.084 | 0.019 | 8.97E-06 | 0.027 | 0.027 | 0.32 | |
| *Rikenellaceae (RC9gut group)* | rs2900503 | G | T | 0.163 | -0.172 | 0.033 | 1.55E-07 | 0.022 | 0.026 | 0.40 | |
| *Rikenellaceae (RC9gut group)* | rs17582787 | A | G | 0.165 | -0.158 | 0.034 | 3.55E-06 | -0.019 | 0.026 | 0.45 | |
| *Rikenellaceae (RC9gut group)* | rs2998141 | T | C | 0.235 | -0.136 | 0.029 | 4.42E-06 | 0.015 | 0.023 | 0.49 | |
| *Rikenellaceae (RC9gut group)* | rs80309088 | G | A | 0.120 | 0.174 | 0.038 | 4.56E-06 | -0.011 | 0.030 | 0.71 | |
| *Rikenellaceae (RC9gut group)* | rs4717843 | G | T | 0.473 | -0.119 | 0.026 | 4.72E-06 | 0.028 | 0.019 | 0.15 | |
| *Rikenellaceae (RC9gut group)* | rs9887954 | G | A | 0.409 | -0.115 | 0.025 | 4.81E-06 | 0.005 | 0.019 | 0.81 | |
| *Rikenellaceae (RC9gut group)* | rs4270579 | G | A | 0.326 | -0.118 | 0.027 | 5.46E-06 | -0.002 | 0.020 | 0.92 | |
| *Rikenellaceae (RC9gut group)* | rs12501673 | A | G | 0.275 | 0.116 | 0.026 | 6.29E-06 | -0.009 | 0.022 | 0.69 | |
| *Rikenellaceae (RC9gut group)* | rs17032291 | T | C | 0.126 | -0.170 | 0.037 | 6.61E-06 | -0.005 | 0.029 | 0.86 | |
| *Rikenellaceae (RC9gut group)* | rs7712231 | A | G | 0.133 | 0.156 | 0.035 | 7.97E-06 | -0.014 | 0.028 | 0.63 | |
| *Rikenellaceae (RC9gut group)* | rs2074881 | T | C | 0.131 | -0.142 | 0.032 | 9.45E-06 | 0.007 | 0.028 | 0.81 | |
| *Romboutsia* | rs61841503 | G | A | 0.129 | 0.093 | 0.017 | 4.00E-08 | -0.030 | 0.029 | 0.29 | |
| *Romboutsia* | rs10279978 | A | G | 0.310 | -0.062 | 0.013 | 1.17E-06 | -0.008 | 0.021 | 0.70 | |
| *Romboutsia* | rs62504452 | A | G | 0.139 | -0.071 | 0.016 | 4.66E-06 | -0.024 | 0.028 | 0.38 | |
| *Romboutsia* | rs75200530 | T | G | 0.029 | -0.191 | 0.042 | 5.07E-06 | -0.084 | 0.057 | 0.14 | |
| *Romboutsia* | rs16843578 | C | T | 0.053 | -0.088 | 0.020 | 5.08E-06 | 0.004 | 0.043 | 0.92 | |
| *Romboutsia* | rs9567264 | C | T | 0.337 | 0.058 | 0.013 | 5.76E-06 | 0.014 | 0.020 | 0.50 | |
| *Romboutsia* | rs34302036 | A | G | 0.421 | 0.055 | 0.012 | 5.88E-06 | -0.027 | 0.019 | 0.17 | |
| *Romboutsia* | rs11221428 | T | C | 0.227 | -0.073 | 0.016 | 6.49E-06 | 0.041 | 0.023 | 0.07 | |
| *Romboutsia* | rs75987356 | G | A | 0.080 | -0.130 | 0.028 | 6.71E-06 | -0.047 | 0.036 | 0.19 | |
| *Romboutsia* | rs7109293 | A | G | 0.116 | 0.092 | 0.021 | 6.98E-06 | 0.007 | 0.030 | 0.82 | |
| *Romboutsia* | rs77702691 | A | G | 0.089 | -0.094 | 0.021 | 7.37E-06 | 0.036 | 0.034 | 0.29 | |
| *Romboutsia* | rs28603357 | T | C | 0.020 | -0.215 | 0.047 | 8.52E-06 | 0.074 | 0.069 | 0.28 | |
| *Romboutsia* | rs9389266 | T | G | 0.175 | 0.072 | 0.016 | 9.38E-06 | 0.019 | 0.025 | 0.46 | |
| *Roseburia* | rs2160994 | T | C | 0.335 | 0.055 | 0.011 | 9.70E-07 | -0.016 | 0.020 | 0.42 | |
| *Roseburia* | rs6930661 | C | T | 0.061 | -0.096 | 0.020 | 2.48E-06 | -0.016 | 0.040 | 0.69 | |
| *Roseburia* | rs16910295 | T | C | 0.055 | -0.098 | 0.021 | 2.91E-06 | -0.048 | 0.042 | 0.26 | |
| *Roseburia* | rs6445851 | G | A | 0.372 | -0.050 | 0.011 | 3.53E-06 | -0.022 | 0.020 | 0.26 | |
| *Roseburia* | rs2943022 | T | C | 0.583 | 0.049 | 0.011 | 4.11E-06 | -0.002 | 0.019 | 0.92 | |
| *Roseburia* | rs9300744 | C | T | 0.178 | -0.059 | 0.013 | 4.75E-06 | -0.025 | 0.025 | 0.32 | |
| *Roseburia* | rs329182 | T | C | 0.159 | 0.069 | 0.015 | 5.90E-06 | -0.011 | 0.026 | 0.66 | |
| *Roseburia* | rs12740451 | T | C | 0.135 | 0.070 | 0.015 | 7.34E-06 | -0.002 | 0.028 | 0.94 | |
| *Roseburia* | rs75326254 | C | T | 0.059 | -0.105 | 0.023 | 7.50E-06 | 0.023 | 0.041 | 0.57 | |
| *Roseburia* | rs302266 | T | C | 0.127 | -0.078 | 0.017 | 8.13E-06 | -0.018 | 0.029 | 0.53 | |
| *Roseburia* | rs57466170 | C | T | 0.075 | 0.074 | 0.017 | 8.30E-06 | -0.056 | 0.037 | 0.12 | |
| *Roseburia* | rs147990086 | A | G | 0.183 | -0.058 | 0.013 | 8.93E-06 | 0.003 | 0.025 | 0.91 | |
| *Roseburia* | rs78753150 | A | C | 0.100 | 0.097 | 0.021 | 9.98E-06 | -0.020 | 0.032 | 0.53 | |
| *Roseburia* | rs55858165 | A | C | 0.038 | 0.179 | 0.040 | 9.99E-06 | 0.068 | 0.051 | 0.18 | |
| *Ruminiclostridium 5* | rs79968837 | A | G | 0.051 | -0.095 | 0.019 | 1.15E-06 | 0.095 | 0.044 | 0.03 | |
| *Ruminiclostridium 5* | rs2482038 | C | A | 0.403 | 0.052 | 0.011 | 1.70E-06 | 0.019 | 0.019 | 0.34 | |
| *Ruminiclostridium 5* | rs10827477 | A | G | 0.353 | -0.055 | 0.012 | 2.19E-06 | -0.020 | 0.020 | 0.32 | |
| *Ruminiclostridium 5* | rs6121460 | G | A | 0.082 | 0.093 | 0.020 | 2.64E-06 | 0.012 | 0.035 | 0.73 | |
| *Ruminiclostridium 5* | rs1492620 | T | C | 0.130 | -0.083 | 0.018 | 3.53E-06 | -0.013 | 0.029 | 0.64 | |
| *Ruminiclostridium 5* | rs113753996 | T | C | 0.182 | 0.082 | 0.017 | 3.99E-06 | -0.012 | 0.025 | 0.63 | |
| *Ruminiclostridium 5* | rs2791343 | T | C | 0.398 | 0.052 | 0.011 | 5.54E-06 | -0.040 | 0.020 | 0.04 | |
| *Ruminiclostridium 5* | rs8053158 | A | G | 0.122 | -0.074 | 0.016 | 5.90E-06 | -0.023 | 0.029 | 0.44 | |
| *Ruminiclostridium 5* | rs2833828 | G | A | 0.412 | 0.049 | 0.011 | 6.82E-06 | 0.044 | 0.019 | 0.02 | |
| *Ruminiclostridium 5* | rs1223978 | T | C | 0.469 | 0.048 | 0.011 | 8.16E-06 | -0.018 | 0.019 | 0.35 | |
| *Ruminiclostridium 5* | rs4955951 | A | G | 0.117 | -0.071 | 0.017 | 9.96E-06 | -0.030 | 0.030 | 0.31 | |
| *Ruminiclostridium 6* | rs71414120 | T | G | 0.051 | 0.201 | 0.041 | 1.08E-06 | -0.025 | 0.043 | 0.56 | |
| *Ruminiclostridium 6* | rs61060922 | T | G | 0.033 | 0.159 | 0.032 | 1.09E-06 | -0.055 | 0.054 | 0.31 | |
| *Ruminiclostridium 6* | rs77193512 | A | G | 0.248 | 0.074 | 0.015 | 1.30E-06 | 0.003 | 0.022 | 0.90 | |
| *Ruminiclostridium 6* | rs79968172 | G | A | 0.059 | 0.116 | 0.024 | 1.66E-06 | 0.004 | 0.041 | 0.92 | |
| *Ruminiclostridium 6* | rs663262 | T | C | 0.034 | -0.135 | 0.031 | 3.39E-06 | 0.047 | 0.053 | 0.38 | |
| *Ruminiclostridium 6* | rs11992182 | A | C | 0.222 | 0.063 | 0.014 | 4.65E-06 | -0.039 | 0.023 | 0.09 | |
| *Ruminiclostridium 6* | rs72991535 | T | G | 0.035 | 0.136 | 0.030 | 4.95E-06 | 0.015 | 0.052 | 0.77 | |
| *Ruminiclostridium 6* | rs2548459 | C | T | 0.409 | 0.055 | 0.012 | 6.40E-06 | 0.005 | 0.019 | 0.78 | |
| *Ruminiclostridium 6* | rs9555756 | A | C | 0.085 | -0.080 | 0.018 | 7.10E-06 | -0.039 | 0.034 | 0.25 | |
| *Ruminiclostridium 6* | rs73176030 | T | C | 0.273 | 0.059 | 0.013 | 7.29E-06 | 0.023 | 0.021 | 0.28 | |
| *Ruminiclostridium 6* | rs792058 | G | A | 0.437 | 0.055 | 0.013 | 8.58E-06 | -0.008 | 0.019 | 0.67 | |
| *Ruminiclostridium 6* | rs35362464 | C | A | 0.140 | 0.072 | 0.017 | 8.99E-06 | -0.016 | 0.028 | 0.55 | |
| *Ruminiclostridium 6* | rs116969552 | A | G | 0.028 | -0.167 | 0.038 | 9.16E-06 | -0.097 | 0.058 | 0.10 | |
| *Ruminiclostridium 6* | rs67479537 | T | C | 0.048 | 0.119 | 0.026 | 9.30E-06 | -0.023 | 0.045 | 0.61 | |
| *Ruminiclostridium 9* | rs918449 | A | G | 0.072 | -0.095 | 0.020 | 2.56E-06 | -0.023 | 0.037 | 0.54 | |
| *Ruminiclostridium 9* | rs9522712 | T | C | 0.149 | 0.070 | 0.015 | 4.66E-06 | 0.005 | 0.027 | 0.86 | |
| *Ruminiclostridium 9* | rs6082461 | A | C | 0.204 | 0.059 | 0.013 | 4.87E-06 | 0.025 | 0.024 | 0.30 | |
| *Ruminiclostridium 9* | rs7137760 | C | T | 0.462 | 0.051 | 0.011 | 7.07E-06 | 0.032 | 0.019 | 0.10 | |
| *Ruminiclostridium 9* | rs78191726 | T | C | 0.073 | 0.094 | 0.021 | 7.58E-06 | -0.060 | 0.037 | 0.10 | |
| *Ruminiclostridium 9* | rs74303178 | T | C | 0.315 | 0.053 | 0.012 | 7.92E-06 | -0.037 | 0.021 | 0.07 | |
| *Ruminiclostridium 9* | rs9809789 | C | T | 0.184 | -0.072 | 0.016 | 8.72E-06 | 0.026 | 0.025 | 0.29 | |
| *Ruminococcaceae (NK4A214 group)* | rs5994253 | A | G | 0.142 | -0.081 | 0.016 | 2.35E-07 | -0.010 | 0.027 | 0.71 | |
| *Ruminococcaceae (NK4A214 group)* | rs11586410 | G | A | 0.158 | -0.086 | 0.017 | 3.66E-07 | -0.043 | 0.027 | 0.10 | |
| *Ruminococcaceae (NK4A214 group)* | rs136761 | G | A | 0.363 | -0.059 | 0.012 | 8.15E-07 | 0.012 | 0.020 | 0.56 | |
| *Ruminococcaceae (NK4A214 group)* | rs7573569 | T | C | 0.060 | 0.108 | 0.023 | 3.23E-06 | 0.055 | 0.040 | 0.17 | |
| *Ruminococcaceae (NK4A214 group)* | rs12642039 | T | C | 0.362 | -0.055 | 0.012 | 3.43E-06 | -0.007 | 0.020 | 0.73 | |
| *Ruminococcaceae (NK4A214 group)* | rs4814689 | C | T | 0.045 | -0.108 | 0.023 | 4.55E-06 | -0.063 | 0.046 | 0.17 | |
| *Ruminococcaceae (NK4A214 group)* | rs147475196 | A | G | 0.107 | -0.134 | 0.030 | 4.72E-06 | 0.083 | 0.030 | 0.01 | |
| *Ruminococcaceae (NK4A214 group)* | rs12731 | A | G | 0.379 | -0.053 | 0.012 | 4.87E-06 | 0.007 | 0.020 | 0.72 | |
| *Ruminococcaceae (NK4A214 group)* | rs35559912 | T | C | 0.121 | -0.093 | 0.020 | 4.89E-06 | -0.024 | 0.029 | 0.41 | |
| *Ruminococcaceae (NK4A214 group)* | rs62027366 | T | C | 0.203 | 0.062 | 0.014 | 6.58E-06 | -0.018 | 0.024 | 0.45 | |
| *Ruminococcaceae (NK4A214 group)* | rs11241747 | C | T | 0.310 | 0.053 | 0.012 | 6.59E-06 | -0.015 | 0.021 | 0.47 | |
| *Ruminococcaceae (NK4A214 group)* | rs13087692 | T | G | 0.297 | 0.057 | 0.013 | 8.69E-06 | -0.023 | 0.021 | 0.28 | |
| *Ruminococcaceae (NK4A214 group)* | rs6681678 | C | T | 0.034 | -0.100 | 0.024 | 9.05E-06 | 0.063 | 0.053 | 0.24 | |
| *Ruminococcaceae (UCG002)* | rs77564310 | A | C | 0.208 | -0.071 | 0.014 | 3.29E-07 | 0.029 | 0.024 | 0.22 | |
| *Ruminococcaceae (UCG002)* | rs55793120 | T | C | 0.063 | 0.137 | 0.027 | 4.81E-07 | -0.013 | 0.040 | 0.75 | |
| *Ruminococcaceae (UCG002)* | rs10927423 | C | A | 0.178 | -0.071 | 0.015 | 8.50E-07 | -0.007 | 0.025 | 0.77 | |
| *Ruminococcaceae (UCG002)* | rs7155595 | C | A | 0.303 | 0.057 | 0.012 | 1.15E-06 | -0.001 | 0.021 | 0.95 | |
| *Ruminococcaceae (UCG002)* | rs11750293 | G | T | 0.370 | -0.058 | 0.012 | 1.76E-06 | 0.023 | 0.020 | 0.25 | |
| *Ruminococcaceae (UCG002)* | rs7120052 | A | C | 0.189 | 0.062 | 0.014 | 1.97E-06 | -0.039 | 0.024 | 0.11 | |
| *Ruminococcaceae (UCG002)* | rs116974815 | C | A | 0.070 | -0.190 | 0.040 | 2.03E-06 | -0.060 | 0.038 | 0.11 | |
| *Ruminococcaceae (UCG002)* | rs10916131 | C | T | 0.162 | -0.069 | 0.015 | 2.87E-06 | -0.025 | 0.026 | 0.33 | |
| *Ruminococcaceae (UCG002)* | rs12463378 | A | G | 0.302 | -0.052 | 0.011 | 2.96E-06 | 0.024 | 0.021 | 0.25 | |
| *Ruminococcaceae (UCG002)* | rs76847269 | A | G | 0.025 | 0.164 | 0.036 | 5.17E-06 | -0.025 | 0.061 | 0.68 | |
| *Ruminococcaceae (UCG002)* | rs882348 | A | G | 0.117 | -0.080 | 0.018 | 5.45E-06 | 0.025 | 0.030 | 0.40 | |
| *Ruminococcaceae (UCG002)* | rs11607472 | A | G | 0.068 | -0.078 | 0.018 | 7.19E-06 | 0.040 | 0.038 | 0.29 | |
| *Ruminococcaceae (UCG002)* | rs57079348 | T | G | 0.057 | -0.077 | 0.017 | 7.22E-06 | -0.011 | 0.042 | 0.79 | |
| *Ruminococcaceae (UCG002)* | rs10964441 | G | A | 0.104 | -0.149 | 0.034 | 7.45E-06 | 0.060 | 0.031 | 0.05 | |
| *Ruminococcaceae (UCG002)* | rs113147300 | A | G | 0.138 | -0.076 | 0.016 | 7.69E-06 | 0.038 | 0.028 | 0.17 | |
| *Ruminococcaceae (UCG002)* | rs6542556 | A | G | 0.381 | 0.051 | 0.011 | 7.86E-06 | -0.014 | 0.020 | 0.49 | |
| *Ruminococcaceae (UCG002)* | rs7249614 | A | G | 0.389 | -0.049 | 0.011 | 9.07E-06 | -0.033 | 0.020 | 0.09 | |
| *Ruminococcaceae (UCG002)* | rs15256 | C | T | 0.117 | 0.073 | 0.017 | 9.46E-06 | 0.005 | 0.030 | 0.86 | |
| *Ruminococcaceae (UCG002)* | rs6793778 | C | T | 0.259 | -0.056 | 0.013 | 9.81E-06 | 0.034 | 0.022 | 0.12 | |
| *Ruminococcaceae (UCG003)* | rs73341549 | T | C | 0.057 | -0.170 | 0.032 | 1.51E-07 | 0.054 | 0.041 | 0.19 | |
| *Ruminococcaceae (UCG003)* | rs646327 | G | A | 0.409 | 0.059 | 0.012 | 7.83E-07 | 0.006 | 0.019 | 0.77 | |
| *Ruminococcaceae (UCG003)* | rs6759615 | A | G | 0.098 | 0.103 | 0.020 | 7.86E-07 | 0.011 | 0.032 | 0.72 | |
| *Ruminococcaceae (UCG003)* | rs11613919 | G | T | 0.221 | 0.073 | 0.016 | 1.63E-06 | -0.016 | 0.023 | 0.49 | |
| *Ruminococcaceae (UCG003)* | rs11243416 | T | C | 0.070 | -0.093 | 0.019 | 1.67E-06 | 0.081 | 0.038 | 0.03 | |
| *Ruminococcaceae (UCG003)* | rs16959793 | A | C | 0.416 | -0.063 | 0.013 | 2.22E-06 | 0.014 | 0.019 | 0.47 | |
| *Ruminococcaceae (UCG003)* | rs4452755 | A | C | 0.339 | -0.063 | 0.013 | 3.29E-06 | 0.023 | 0.020 | 0.26 | |
| *Ruminococcaceae (UCG003)* | rs10490280 | C | T | 0.189 | -0.067 | 0.014 | 4.16E-06 | 0.006 | 0.024 | 0.80 | |
| *Ruminococcaceae (UCG003)* | rs3013089 | G | A | 0.375 | -0.055 | 0.012 | 4.38E-06 | 0.004 | 0.020 | 0.83 | |
| *Ruminococcaceae (UCG003)* | rs4532474 | G | A | 0.163 | 0.077 | 0.017 | 4.82E-06 | 0.006 | 0.026 | 0.82 | |
| *Ruminococcaceae (UCG003)* | rs2523124 | T | C | 0.402 | -0.055 | 0.012 | 5.78E-06 | -0.005 | 0.020 | 0.79 | |
| *Ruminococcaceae (UCG003)* | rs78720113 | A | G | 0.079 | -0.115 | 0.025 | 7.59E-06 | 0.008 | 0.036 | 0.82 | |
| *Ruminococcaceae (UCG004)* | rs6769553 | A | G | 0.261 | 0.085 | 0.016 | 7.91E-08 | -0.032 | 0.022 | 0.14 | |
| *Ruminococcaceae (UCG004)* | rs12125734 | G | T | 0.093 | 0.134 | 0.026 | 2.09E-07 | -0.027 | 0.033 | 0.41 | |
| *Ruminococcaceae (UCG004)* | rs511258 | G | A | 0.186 | -0.076 | 0.016 | 4.52E-06 | 0.005 | 0.025 | 0.84 | |
| *Ruminococcaceae (UCG004)* | rs9818949 | G | T | 0.203 | 0.086 | 0.019 | 5.39E-06 | -0.031 | 0.024 | 0.20 | |
| *Ruminococcaceae (UCG004)* | rs872501 | G | A | 0.099 | 0.116 | 0.026 | 5.81E-06 | 0.035 | 0.037 | 0.34 | |
| *Ruminococcaceae (UCG004)* | rs3800154 | A | C | 0.265 | -0.080 | 0.018 | 6.12E-06 | -0.015 | 0.022 | 0.49 | |
| *Ruminococcaceae (UCG004)* | rs10976229 | T | G | 0.129 | 0.096 | 0.021 | 7.04E-06 | 0.038 | 0.028 | 0.18 | |
| *Ruminococcaceae (UCG004)* | rs7569771 | A | G | 0.249 | -0.076 | 0.017 | 8.12E-06 | 0.030 | 0.022 | 0.18 | |
| *Ruminococcaceae (UCG004)* | rs2248146 | T | C | 0.344 | 0.069 | 0.015 | 8.20E-06 | -0.016 | 0.020 | 0.42 | |
| *Ruminococcaceae (UCG004)* | rs11961899 | G | A | 0.278 | -0.071 | 0.016 | 9.18E-06 | -0.003 | 0.021 | 0.89 | |
| *Ruminococcaceae (UCG004)* | rs550351 | A | C | 0.425 | 0.079 | 0.018 | 9.43E-06 | -0.010 | 0.019 | 0.62 | |
| *Ruminococcaceae (UCG005)* | rs10950694 | T | C | 0.359 | 0.058 | 0.011 | 4.30E-07 | -0.036 | 0.020 | 0.07 | |
| *Ruminococcaceae (UCG005)* | rs34781347 | G | A | 0.071 | 0.189 | 0.039 | 6.05E-07 | 0.001 | 0.037 | 0.99 | |
| *Ruminococcaceae (UCG005)* | rs12458218 | T | C | 0.181 | 0.068 | 0.014 | 2.41E-06 | -0.016 | 0.025 | 0.53 | |
| *Ruminococcaceae (UCG005)* | rs7555878 | A | G | 0.247 | 0.059 | 0.013 | 2.81E-06 | 0.025 | 0.022 | 0.25 | |
| *Ruminococcaceae (UCG005)* | rs12288512 | A | G | 0.239 | 0.067 | 0.014 | 3.10E-06 | -0.012 | 0.022 | 0.60 | |
| *Ruminococcaceae (UCG005)* | rs114279581 | A | G | 0.076 | -0.147 | 0.032 | 3.22E-06 | 0.047 | 0.036 | 0.19 | |
| *Ruminococcaceae (UCG005)* | rs2893871 | G | A | 0.110 | -0.074 | 0.016 | 3.54E-06 | -0.031 | 0.031 | 0.31 | |
| *Ruminococcaceae (UCG005)* | rs10873449 | T | C | 0.192 | 0.065 | 0.014 | 4.11E-06 | 0.031 | 0.024 | 0.19 | |
| *Ruminococcaceae (UCG005)* | rs7449320 | C | A | 0.232 | 0.060 | 0.013 | 4.81E-06 | 0.014 | 0.023 | 0.54 | |
| *Ruminococcaceae (UCG005)* | rs72776570 | C | A | 0.097 | 0.087 | 0.020 | 5.36E-06 | 0.050 | 0.032 | 0.12 | |
| *Ruminococcaceae (UCG005)* | rs55793120 | T | C | 0.063 | 0.122 | 0.028 | 7.37E-06 | -0.013 | 0.040 | 0.75 | |
| *Ruminococcaceae (UCG005)* | rs898577 | T | C | 0.057 | -0.123 | 0.029 | 7.46E-06 | -0.068 | 0.041 | 0.10 | |
| *Ruminococcaceae (UCG005)* | rs10937802 | G | A | 0.120 | 0.076 | 0.017 | 8.17E-06 | 0.042 | 0.030 | 0.15 | |
| *Ruminococcaceae (UCG005)* | rs7586445 | G | A | 0.130 | 0.078 | 0.018 | 8.81E-06 | -0.033 | 0.028 | 0.25 | |
| *Ruminococcaceae (UCG009)* | rs1550196 | G | A | 0.100 | 0.131 | 0.026 | 1.13E-06 | 0.036 | 0.032 | 0.26 | |
| *Ruminococcaceae (UCG009)* | rs4708333 | T | G | 0.342 | -0.084 | 0.017 | 1.56E-06 | 0.023 | 0.020 | 0.25 | |
| *Ruminococcaceae (UCG009)* | rs2058609 | A | G | 0.276 | 0.082 | 0.017 | 3.12E-06 | -0.009 | 0.021 | 0.67 | |
| *Ruminococcaceae (UCG009)* | rs4079028 | C | T | 0.240 | 0.092 | 0.020 | 3.28E-06 | -0.014 | 0.022 | 0.53 | |
| *Ruminococcaceae (UCG009)* | rs12508214 | C | T | 0.339 | -0.077 | 0.017 | 4.75E-06 | 0.011 | 0.020 | 0.58 | |
| *Ruminococcaceae (UCG009)* | rs2192926 | A | G | 0.329 | -0.089 | 0.019 | 4.88E-06 | -0.015 | 0.020 | 0.46 | |
| *Ruminococcaceae (UCG009)* | rs9558661 | T | C | 0.204 | -0.090 | 0.020 | 7.01E-06 | -0.014 | 0.024 | 0.56 | |
| *Ruminococcaceae (UCG009)* | rs113006825 | T | C | 0.216 | -0.093 | 0.021 | 7.98E-06 | 0.030 | 0.023 | 0.20 | |
| *Ruminococcaceae (UCG009)* | rs6952765 | G | A | 0.317 | 0.073 | 0.017 | 8.13E-06 | 0.015 | 0.021 | 0.48 | |
| *Ruminococcaceae (UCG009)* | rs758191 | T | G | 0.101 | 0.177 | 0.038 | 9.01E-06 | 0.036 | 0.032 | 0.26 | |
| *Ruminococcaceae (UCG009)* | rs78410648 | A | G | 0.109 | 0.121 | 0.028 | 9.67E-06 | 0.057 | 0.031 | 0.06 | |
| *Ruminococcaceae (UCG009)* | rs138460696 | A | G | 0.077 | 0.139 | 0.032 | 9.81E-06 | 0.043 | 0.036 | 0.24 | |
| *Ruminococcaceae (UCG010)* | rs682403 | A | G | 0.528 | -0.059 | 0.012 | 2.37E-06 | 0.032 | 0.019 | 0.09 | |
| *Ruminococcaceae (UCG010)* | rs6958419 | C | T | 0.467 | -0.059 | 0.012 | 2.84E-06 | 0.010 | 0.019 | 0.60 | |
| *Ruminococcaceae (UCG010)* | rs2820282 | A | C | 0.402 | -0.059 | 0.013 | 2.85E-06 | 0.010 | 0.020 | 0.62 | |
| *Ruminococcaceae (UCG010)* | rs12597105 | G | A | 0.189 | 0.067 | 0.014 | 4.87E-06 | 0.008 | 0.024 | 0.75 | |
| *Ruminococcaceae (UCG010)* | rs73218807 | G | A | 0.081 | -0.166 | 0.037 | 6.43E-06 | -0.004 | 0.035 | 0.91 | |
| *Ruminococcaceae (UCG010)* | rs7441445 | C | T | 0.514 | -0.057 | 0.013 | 6.80E-06 | 0.015 | 0.019 | 0.45 | |
| *Ruminococcaceae (UCG011)* | rs1416041 | A | C | 0.209 | -0.182 | 0.034 | 7.04E-08 | 0.021 | 0.024 | 0.37 | |
| *Ruminococcaceae (UCG011)* | rs12724320 | C | T | 0.383 | -0.121 | 0.025 | 1.52E-06 | -0.045 | 0.020 | 0.02 | |
| *Ruminococcaceae (UCG011)* | rs79113084 | C | T | 0.109 | -0.152 | 0.032 | 2.06E-06 | 0.020 | 0.032 | 0.52 | |
| *Ruminococcaceae (UCG011)* | rs9729514 | A | G | 0.093 | 0.185 | 0.039 | 2.37E-06 | -0.038 | 0.033 | 0.24 | |
| *Ruminococcaceae (UCG011)* | rs12636310 | G | A | 0.252 | 0.133 | 0.028 | 2.81E-06 | 0.004 | 0.022 | 0.86 | |
| *Ruminococcaceae (UCG011)* | rs2729556 | C | T | 0.477 | -0.109 | 0.023 | 3.19E-06 | -0.007 | 0.019 | 0.71 | |
| *Ruminococcaceae (UCG011)* | rs10274562 | C | T | 0.381 | 0.111 | 0.024 | 6.50E-06 | 0.019 | 0.020 | 0.33 | |
| *Ruminococcaceae (UCG011)* | rs4490371 | T | C | 0.413 | -0.112 | 0.025 | 7.75E-06 | 0.006 | 0.020 | 0.75 | |
| *Ruminococcaceae (UCG013)* | rs12781711 | C | T | 0.249 | -0.066 | 0.012 | 2.55E-08 | 0.024 | 0.022 | 0.27 | |
| *Ruminococcaceae (UCG013)* | rs12189346 | G | A | 0.186 | 0.068 | 0.015 | 1.68E-06 | -0.011 | 0.024 | 0.64 | |
| *Ruminococcaceae (UCG013)* | rs75088940 | T | C | 0.071 | -0.094 | 0.020 | 2.55E-06 | -0.025 | 0.038 | 0.51 | |
| *Ruminococcaceae (UCG013)* | rs76973485 | G | T | 0.041 | 0.195 | 0.042 | 3.35E-06 | 0.043 | 0.049 | 0.38 | |
| *Ruminococcaceae (UCG013)* | rs16918863 | A | C | 0.063 | 0.111 | 0.024 | 4.16E-06 | -0.005 | 0.039 | 0.90 | |
| *Ruminococcaceae (UCG013)* | rs12485353 | G | A | 0.249 | -0.061 | 0.013 | 4.19E-06 | 0.035 | 0.022 | 0.11 | |
| *Ruminococcaceae (UCG013)* | rs11581881 | C | T | 0.236 | 0.066 | 0.014 | 4.73E-06 | 0.026 | 0.023 | 0.25 | |
| *Ruminococcaceae (UCG013)* | rs4385846 | G | T | 0.195 | 0.060 | 0.013 | 6.46E-06 | -0.008 | 0.024 | 0.75 | |
| *Ruminococcaceae (UCG013)* | rs2730183 | G | A | 0.398 | -0.049 | 0.011 | 8.44E-06 | 0.026 | 0.020 | 0.19 | |
| *Ruminococcaceae (UCG013)* | rs12336782 | T | C | 0.078 | -0.086 | 0.019 | 8.60E-06 | 0.040 | 0.036 | 0.26 | |
| *Ruminococcaceae (UCG013)* | rs9313055 | T | C | 0.081 | 0.105 | 0.023 | 9.55E-06 | 0.010 | 0.035 | 0.78 | |
| *Ruminococcaceae (UCG014)* | rs115777838 | T | C | 0.112 | -0.188 | 0.039 | 4.62E-07 | -0.038 | 0.030 | 0.21 | |
| *Ruminococcaceae (UCG014)* | rs12638134 | T | G | 0.416 | 0.058 | 0.012 | 1.21E-06 | 0.024 | 0.019 | 0.22 | |
| *Ruminococcaceae (UCG014)* | rs995642 | C | T | 0.238 | 0.060 | 0.013 | 1.90E-06 | 0.003 | 0.022 | 0.89 | |
| *Ruminococcaceae (UCG014)* | rs10941294 | C | T | 0.054 | -0.122 | 0.026 | 2.40E-06 | 0.011 | 0.042 | 0.79 | |
| *Ruminococcaceae (UCG014)* | rs72809222 | T | C | 0.212 | 0.067 | 0.014 | 2.41E-06 | -0.019 | 0.023 | 0.41 | |
| *Ruminococcaceae (UCG014)* | rs56105232 | G | A | 0.062 | 0.139 | 0.030 | 2.91E-06 | -0.006 | 0.040 | 0.89 | |
| *Ruminococcaceae (UCG014)* | rs73186226 | G | A | 0.076 | -0.099 | 0.022 | 6.72E-06 | -0.039 | 0.037 | 0.29 | |
| *Ruminococcaceae (UCG014)* | rs853612 | A | G | 0.394 | -0.053 | 0.012 | 9.75E-06 | 0.017 | 0.020 | 0.38 | |
| *Ruminococcaceae (UCG014)* | rs10791168 | A | G | 0.185 | -0.066 | 0.015 | 9.76E-06 | -0.009 | 0.025 | 0.72 | |
| *Ruminococcaceae (UCG014)* | rs34402072 | C | T | 0.146 | -0.069 | 0.016 | 9.80E-06 | 0.012 | 0.027 | 0.67 | |
| *Ruminococcaceae (UCG014)* | rs10495392 | C | T | 0.072 | -0.082 | 0.019 | 9.96E-06 | -0.022 | 0.037 | 0.55 | |
| *Ruminococcus 1* | rs7117576 | A | G | 0.092 | 0.083 | 0.017 | 6.48E-07 | -0.074 | 0.033 | 0.03 | |
| *Ruminococcus 1* | rs17781867 | C | T | 0.069 | 0.100 | 0.021 | 1.96E-06 | 0.027 | 0.038 | 0.48 | |
| *Ruminococcus 1* | rs7583465 | C | T | 0.439 | 0.053 | 0.011 | 2.56E-06 | 0.008 | 0.019 | 0.67 | |
| *Ruminococcus 1* | rs6493760 | C | T | 0.356 | 0.054 | 0.012 | 3.38E-06 | -0.019 | 0.020 | 0.35 | |
| *Ruminococcus 1* | rs11783695 | G | T | 0.159 | -0.073 | 0.016 | 4.73E-06 | -0.014 | 0.026 | 0.58 | |
| *Ruminococcus 1* | rs6105066 | T | C | 0.277 | -0.061 | 0.013 | 5.06E-06 | 0.010 | 0.021 | 0.63 | |
| *Ruminococcus 1* | rs78613526 | G | A | 0.053 | 0.167 | 0.037 | 5.11E-06 | -0.016 | 0.044 | 0.71 | |
| *Ruminococcus 1* | rs78572139 | G | A | 0.102 | 0.125 | 0.028 | 5.23E-06 | -0.028 | 0.032 | 0.39 | |
| *Ruminococcus 1* | rs10167839 | A | G | 0.363 | 0.052 | 0.012 | 8.09E-06 | -0.032 | 0.020 | 0.11 | |
| *Ruminococcus 1* | rs3819978 | C | T | 0.077 | -0.115 | 0.026 | 8.74E-06 | 0.037 | 0.036 | 0.31 | |
| *Ruminococcus 2* | rs78120384 | A | G | 0.093 | -0.193 | 0.039 | 3.31E-07 | -0.004 | 0.033 | 0.90 | |
| *Ruminococcus 2* | rs7635831 | G | A | 0.377 | 0.062 | 0.013 | 1.98E-06 | -0.002 | 0.020 | 0.93 | |
| *Ruminococcus 2* | rs12986628 | C | T | 0.194 | 0.067 | 0.014 | 2.14E-06 | 0.047 | 0.024 | 0.06 | |
| *Ruminococcus 2* | rs2368224 | T | G | 0.049 | 0.200 | 0.044 | 3.63E-06 | -0.005 | 0.044 | 0.92 | |
| *Ruminococcus 2* | rs75140805 | T | G | 0.181 | 0.084 | 0.018 | 3.95E-06 | -0.015 | 0.025 | 0.55 | |
| *Ruminococcus 2* | rs58681734 | A | G | 0.212 | 0.072 | 0.016 | 4.18E-06 | 0.038 | 0.023 | 0.10 | |
| *Ruminococcus 2* | rs2997412 | A | G | 0.280 | -0.057 | 0.012 | 4.22E-06 | 0.006 | 0.021 | 0.77 | |
| *Ruminococcus 2* | rs1819812 | G | T | 0.053 | 0.084 | 0.018 | 5.28E-06 | -0.037 | 0.043 | 0.39 | |
| *Ruminococcus 2* | rs4799823 | C | T | 0.187 | 0.084 | 0.018 | 5.40E-06 | -0.005 | 0.025 | 0.83 | |
| *Ruminococcus 2* | rs4400279 | A | G | 0.323 | 0.055 | 0.012 | 5.80E-06 | 0.002 | 0.020 | 0.92 | |
| *Ruminococcus 2* | rs61791565 | T | C | 0.506 | -0.052 | 0.012 | 6.79E-06 | 0.030 | 0.019 | 0.12 | |
| *Ruminococcus 2* | rs2846589 | G | T | 0.425 | 0.052 | 0.012 | 7.59E-06 | 0.009 | 0.020 | 0.64 | |
| *Ruminococcus 2* | rs55707116 | C | A | 0.074 | 0.087 | 0.019 | 8.01E-06 | -0.014 | 0.037 | 0.70 | |
| *Ruminococcus 2* | rs7693984 | G | A | 0.044 | -0.103 | 0.024 | 9.42E-06 | -0.027 | 0.047 | 0.57 | |
| *Ruminococcus 2* | rs12406309 | A | C | 0.221 | -0.063 | 0.014 | 9.79E-06 | -0.029 | 0.023 | 0.21 | |
| *Ruminococcus (gauvreauii group)* | rs2047242 | A | G | 0.230 | -0.068 | 0.013 | 2.46E-07 | -0.011 | 0.023 | 0.64 | |
| *Ruminococcus (gauvreauii group)* | rs71386687 | T | G | 0.115 | 0.121 | 0.024 | 2.91E-07 | -0.028 | 0.031 | 0.35 | |
| *Ruminococcus (gauvreauii group)* | rs9870933 | A | G | 0.397 | 0.062 | 0.013 | 8.49E-07 | 0.002 | 0.020 | 0.90 | |
| *Ruminococcus (gauvreauii group)* | rs1391597 | C | T | 0.394 | 0.059 | 0.012 | 1.86E-06 | -0.006 | 0.019 | 0.74 | |
| *Ruminococcus (gauvreauii group)* | rs289410 | G | A | 0.278 | -0.065 | 0.014 | 2.27E-06 | -0.001 | 0.021 | 0.95 | |
| *Ruminococcus (gauvreauii group)* | rs10931481 | G | A | 0.289 | 0.061 | 0.013 | 3.38E-06 | -0.020 | 0.021 | 0.33 | |
| *Ruminococcus (gauvreauii group)* | rs12539819 | C | T | 0.070 | 0.111 | 0.024 | 4.49E-06 | -0.033 | 0.039 | 0.40 | |
| *Ruminococcus (gauvreauii group)* | rs12079579 | A | G | 0.084 | 0.096 | 0.021 | 5.04E-06 | 0.050 | 0.035 | 0.15 | |
| *Ruminococcus (gauvreauii group)* | rs2166943 | A | C | 0.418 | 0.057 | 0.012 | 5.28E-06 | -0.013 | 0.019 | 0.50 | |
| *Ruminococcus (gauvreauii group)* | rs431418 | A | G | 0.095 | -0.095 | 0.021 | 5.54E-06 | -0.001 | 0.033 | 0.98 | |
| *Ruminococcus (gauvreauii group)* | rs73802842 | C | A | 0.239 | 0.074 | 0.017 | 7.48E-06 | -0.005 | 0.023 | 0.82 | |
| *Ruminococcus (gnavus group)* | rs13163520 | G | A | 0.185 | -0.127 | 0.023 | 5.61E-08 | 0.004 | 0.025 | 0.86 | |
| *Ruminococcus (gnavus group)* | rs2909242 | C | A | 0.332 | -0.091 | 0.018 | 7.41E-07 | -0.007 | 0.020 | 0.72 | |
| *Ruminococcus (gnavus group)* | rs3124783 | A | G | 0.129 | -0.116 | 0.025 | 2.67E-06 | -0.015 | 0.028 | 0.60 | |
| *Ruminococcus (gnavus group)* | rs934940 | A | C | 0.134 | -0.105 | 0.023 | 2.74E-06 | -0.016 | 0.028 | 0.58 | |
| *Ruminococcus (gnavus group)* | rs12136548 | C | T | 0.287 | 0.090 | 0.020 | 3.10E-06 | 0.016 | 0.021 | 0.46 | |
| *Ruminococcus (gnavus group)* | rs62167033 | T | C | 0.041 | 0.185 | 0.040 | 3.50E-06 | 0.013 | 0.049 | 0.79 | |
| *Ruminococcus (gnavus group)* | rs11864644 | T | C | 0.116 | -0.140 | 0.032 | 5.01E-06 | -0.015 | 0.030 | 0.61 | |
| *Ruminococcus (gnavus group)* | rs78399089 | T | C | 0.115 | 0.144 | 0.033 | 6.63E-06 | -0.013 | 0.030 | 0.68 | |
| *Ruminococcus (gnavus group)* | rs11597105 | A | G | 0.196 | 0.115 | 0.025 | 6.95E-06 | 0.003 | 0.024 | 0.90 | |
| *Ruminococcus (gnavus group)* | rs12989336 | G | A | 0.279 | -0.085 | 0.019 | 7.12E-06 | 0.027 | 0.021 | 0.20 | |
| *Ruminococcus (gnavus group)* | rs4388134 | C | T | 0.278 | -0.090 | 0.020 | 9.12E-06 | 0.005 | 0.021 | 0.83 | |
| *Ruminococcus (torques group)* | rs35866622 | T | C | 0.338 | -0.061 | 0.011 | 2.21E-08 | 0.016 | 0.020 | 0.43 | |
| *Ruminococcus (torques group)* | rs10904297 | A | G | 0.022 | -0.168 | 0.039 | 2.69E-06 | -0.093 | 0.066 | 0.16 | |
| *Ruminococcus (torques group)* | rs12434631 | A | G | 0.105 | 0.075 | 0.015 | 2.77E-06 | 0.025 | 0.031 | 0.42 | |
| *Ruminococcus (torques group)* | rs4073731 | T | C | 0.163 | 0.065 | 0.014 | 4.05E-06 | 0.047 | 0.026 | 0.07 | |
| *Ruminococcus (torques group)* | rs77034621 | T | G | 0.017 | -0.152 | 0.034 | 6.07E-06 | -0.114 | 0.074 | 0.13 | |
| *Ruminococcus (torques group)* | rs1475330 | T | C | 0.240 | 0.052 | 0.012 | 8.13E-06 | -0.006 | 0.023 | 0.79 | |
| *Ruminococcus (torques group)* | rs10967781 | C | A | 0.289 | 0.051 | 0.011 | 8.37E-06 | -0.028 | 0.021 | 0.18 | |
| *Sellimonas* | rs2371572 | A | C | 0.466 | 0.127 | 0.025 | 4.46E-07 | -0.004 | 0.019 | 0.82 | |
| *Sellimonas* | rs13417181 | T | C | 0.260 | 0.167 | 0.034 | 7.62E-07 | -0.028 | 0.023 | 0.21 | |
| *Sellimonas* | rs2016057 | A | C | 0.385 | -0.126 | 0.026 | 1.03E-06 | 0.023 | 0.020 | 0.24 | |
| *Sellimonas* | rs56203279 | T | C | 0.335 | -0.124 | 0.027 | 3.72E-06 | -0.003 | 0.020 | 0.89 | |
| *Sellimonas* | rs2187447 | A | C | 0.061 | 0.243 | 0.053 | 3.98E-06 | -0.021 | 0.040 | 0.61 | |
| *Sellimonas* | rs4600608 | A | G | 0.212 | -0.137 | 0.030 | 4.95E-06 | -0.004 | 0.023 | 0.88 | |
| *Sellimonas* | rs553697 | T | C | 0.188 | -0.154 | 0.034 | 6.13E-06 | -0.026 | 0.025 | 0.29 | |
| *Sellimonas* | rs113379006 | T | C | 0.176 | -0.163 | 0.036 | 7.21E-06 | 0.001 | 0.025 | 0.96 | |
| *Sellimonas* | rs41816 | A | G | 0.300 | 0.132 | 0.029 | 8.39E-06 | -0.008 | 0.021 | 0.72 | |
| *Senegalimassilia* | rs11787826 | C | A | 0.409 | 0.081 | 0.017 | 2.63E-06 | -0.036 | 0.019 | 0.06 | |
| *Senegalimassilia* | rs7225245 | G | A | 0.431 | 0.079 | 0.017 | 4.18E-06 | -0.004 | 0.019 | 0.85 | |
| *Senegalimassilia* | rs10036909 | C | T | 0.041 | 0.186 | 0.040 | 8.05E-06 | -0.024 | 0.048 | 0.62 | |
| *Senegalimassilia* | rs1990708 | A | C | 0.078 | -0.110 | 0.025 | 8.91E-06 | 0.022 | 0.036 | 0.54 | |
| *Senegalimassilia* | rs2017373 | C | T | 0.364 | 0.078 | 0.018 | 9.50E-06 | 0.003 | 0.020 | 0.88 | |
| *Slackia* | rs8901 | C | T | 0.292 | 0.093 | 0.019 | 6.07E-07 | 0.019 | 0.021 | 0.37 | |
| *Slackia* | rs4492265 | A | G | 0.304 | -0.091 | 0.019 | 2.41E-06 | 0.004 | 0.021 | 0.87 | |
| *Slackia* | rs12440440 | A | G | 0.342 | 0.090 | 0.019 | 2.63E-06 | 0.003 | 0.020 | 0.90 | |
| *Slackia* | rs16894137 | C | T | 0.131 | -0.123 | 0.026 | 2.71E-06 | -0.003 | 0.028 | 0.92 | |
| *Slackia* | rs10409783 | A | G | 0.291 | 0.095 | 0.021 | 7.70E-06 | -0.002 | 0.021 | 0.93 | |
| *Slackia* | rs35156985 | T | C | 0.042 | -0.156 | 0.035 | 8.06E-06 | -0.009 | 0.048 | 0.85 | |
| *Streptococcus* | rs11110281 | T | C | 0.048 | -0.138 | 0.023 | 2.58E-09 | 0.005 | 0.045 | 0.91 | |
| *Streptococcus* | rs11764382 | A | G | 0.140 | -0.070 | 0.014 | 1.29E-06 | -0.014 | 0.028 | 0.60 | |
| *Streptococcus* | rs1918540 | G | A | 0.187 | 0.060 | 0.013 | 2.44E-06 | -0.029 | 0.025 | 0.25 | |
| *Streptococcus* | rs7916711 | A | G | 0.140 | 0.103 | 0.022 | 2.72E-06 | -0.006 | 0.027 | 0.84 | |
| *Streptococcus* | rs17708276 | A | G | 0.109 | -0.079 | 0.017 | 3.04E-06 | -0.022 | 0.031 | 0.47 | |
| *Streptococcus* | rs10448310 | A | G | 0.361 | -0.052 | 0.011 | 3.31E-06 | -0.034 | 0.020 | 0.09 | |
| *Streptococcus* | rs11720390 | G | A | 0.062 | 0.107 | 0.023 | 3.59E-06 | 0.009 | 0.040 | 0.81 | |
| *Streptococcus* | rs6806351 | T | C | 0.220 | -0.063 | 0.014 | 4.94E-06 | -0.005 | 0.023 | 0.84 | |
| *Streptococcus* | rs57646748 | G | A | 0.038 | -0.091 | 0.020 | 5.48E-06 | -0.083 | 0.050 | 0.10 | |
| *Streptococcus* | rs71481756 | T | G | 0.066 | 0.093 | 0.021 | 6.51E-06 | 0.026 | 0.039 | 0.51 | |
| *Streptococcus* | rs10028567 | C | T | 0.121 | -0.092 | 0.019 | 7.30E-06 | -0.050 | 0.029 | 0.08 | |
| *Streptococcus* | rs2370083 | G | T | 0.064 | -0.082 | 0.019 | 9.75E-06 | 0.011 | 0.039 | 0.77 | |
| *Subdoligranulum* | rs10065321 | T | C | 0.413 | -0.051 | 0.011 | 2.10E-06 | -0.002 | 0.019 | 0.90 | |
| *Subdoligranulum* | rs4347804 | A | G | 0.032 | 0.166 | 0.036 | 2.18E-06 | -0.008 | 0.055 | 0.88 | |
| *Subdoligranulum* | rs2114677 | C | T | 0.115 | -0.104 | 0.023 | 2.72E-06 | -0.014 | 0.030 | 0.63 | |
| *Subdoligranulum* | rs6555306 | T | C | 0.141 | -0.074 | 0.016 | 2.81E-06 | -0.021 | 0.027 | 0.45 | |
| *Subdoligranulum* | rs3761728 | T | G | 0.253 | -0.054 | 0.012 | 3.87E-06 | -0.040 | 0.022 | 0.07 | |
| *Subdoligranulum* | rs35940633 | G | A | 0.324 | -0.051 | 0.011 | 4.22E-06 | -0.046 | 0.020 | 0.02 | |
| *Subdoligranulum* | rs2171249 | C | T | 0.074 | 0.107 | 0.023 | 4.51E-06 | 0.054 | 0.037 | 0.15 | |
| *Subdoligranulum* | rs1667315 | G | A | 0.391 | 0.049 | 0.011 | 6.72E-06 | 0.006 | 0.020 | 0.76 | |
| *Subdoligranulum* | rs76528319 | G | T | 0.083 | -0.143 | 0.031 | 7.41E-06 | 0.015 | 0.035 | 0.68 | |
| *Subdoligranulum* | rs75158211 | T | C | 0.148 | -0.072 | 0.016 | 7.52E-06 | 0.033 | 0.027 | 0.22 | |
| *Subdoligranulum* | rs10497836 | C | T | 0.212 | -0.052 | 0.012 | 8.38E-06 | 0.036 | 0.023 | 0.13 | |
| *Sutterella* | rs2321387 | G | A | 0.431 | -0.059 | 0.012 | 1.87E-06 | -0.001 | 0.019 | 0.97 | |
| *Sutterella* | rs7499539 | A | G | 0.263 | 0.062 | 0.013 | 2.36E-06 | 0.019 | 0.022 | 0.38 | |
| *Sutterella* | rs13173038 | A | G | 0.248 | -0.072 | 0.015 | 2.73E-06 | 0.042 | 0.022 | 0.06 | |
| *Sutterella* | rs143438747 | T | C | 0.076 | -0.146 | 0.031 | 3.28E-06 | 0.002 | 0.036 | 0.96 | |
| *Sutterella* | rs62501473 | G | A | 0.264 | 0.069 | 0.015 | 5.52E-06 | 0.026 | 0.022 | 0.24 | |
| *Sutterella* | rs11591622 | T | G | 0.162 | -0.069 | 0.015 | 6.50E-06 | 0.041 | 0.026 | 0.11 | |
| *Sutterella* | rs607327 | C | T | 0.375 | 0.058 | 0.013 | 6.63E-06 | -0.003 | 0.020 | 0.87 | |
| *Sutterella* | rs1145877 | A | G | 0.138 | -0.074 | 0.016 | 7.20E-06 | -0.031 | 0.028 | 0.26 | |
| *Sutterella* | rs2613606 | C | T | 0.418 | -0.056 | 0.012 | 7.20E-06 | -0.015 | 0.019 | 0.44 | |
| *Sutterella* | rs2050185 | G | A | 0.375 | 0.058 | 0.013 | 7.97E-06 | 0.012 | 0.020 | 0.55 | |
| *Sutterella* | rs9350083 | T | G | 0.355 | -0.059 | 0.013 | 8.23E-06 | -0.022 | 0.020 | 0.27 | |
| *Sutterella* | rs7638039 | T | C | 0.246 | 0.065 | 0.014 | 8.66E-06 | -0.002 | 0.022 | 0.92 | |
| *Terrisporobacter* | rs1883097 | C | T | 0.038 | 0.226 | 0.045 | 4.16E-07 | 0.001 | 0.050 | 0.98 | |
| *Terrisporobacter* | rs2872237 | C | A | 0.399 | -0.081 | 0.018 | 3.97E-06 | -0.009 | 0.019 | 0.66 | |
| *Terrisporobacter* | rs58405430 | G | T | 0.060 | 0.135 | 0.030 | 7.94E-06 | -0.004 | 0.041 | 0.92 | |
| *Terrisporobacter* | rs7184125 | T | C | 0.292 | 0.091 | 0.021 | 8.48E-06 | 0.006 | 0.021 | 0.77 | |
| *Terrisporobacter* | rs2569953 | A | C | 0.416 | -0.078 | 0.017 | 8.95E-06 | 0.012 | 0.019 | 0.54 | |
| *Turicibacter* | rs149744580 | A | G | 0.053 | 0.170 | 0.032 | 7.01E-08 | 0.029 | 0.043 | 0.50 | |
| *Turicibacter* | rs11054680 | T | C | 0.171 | -0.105 | 0.023 | 2.31E-06 | 0.019 | 0.025 | 0.46 | |
| *Turicibacter* | rs4869133 | G | A | 0.177 | 0.131 | 0.027 | 2.55E-06 | -0.021 | 0.025 | 0.40 | |
| *Turicibacter* | rs55756211 | T | C | 0.073 | -0.115 | 0.024 | 2.81E-06 | 0.008 | 0.037 | 0.83 | |
| *Turicibacter* | rs2834977 | T | C | 0.153 | -0.096 | 0.021 | 3.96E-06 | -0.021 | 0.027 | 0.43 | |
| *Turicibacter* | rs3734633 | G | A | 0.058 | -0.121 | 0.027 | 5.32E-06 | -0.037 | 0.041 | 0.37 | |
| *Turicibacter* | rs2952020 | G | A | 0.248 | -0.076 | 0.017 | 5.63E-06 | -0.003 | 0.022 | 0.88 | |
| *Turicibacter* | rs7199484 | G | A | 0.306 | -0.073 | 0.016 | 5.77E-06 | -0.030 | 0.021 | 0.14 | |
| *Turicibacter* | rs11666533 | C | T | 0.084 | -0.112 | 0.025 | 7.37E-06 | -0.041 | 0.035 | 0.24 | |
| *Tyzzerella 3* | rs67476743 | T | G | 0.273 | 0.132 | 0.022 | 3.74E-09 | 0.008 | 0.022 | 0.70 | |
| *Tyzzerella 3* | rs17706273 | T | C | 0.078 | -0.140 | 0.027 | 5.88E-07 | -0.017 | 0.036 | 0.64 | |
| *Tyzzerella 3* | rs55799124 | A | G | 0.275 | -0.114 | 0.024 | 1.34E-06 | 0.033 | 0.022 | 0.14 | |
| *Tyzzerella 3* | rs7561370 | T | C | 0.149 | 0.131 | 0.029 | 1.52E-06 | -0.057 | 0.027 | 0.03 | |
| *Tyzzerella 3* | rs75091807 | G | T | 0.062 | -0.185 | 0.038 | 1.71E-06 | 0.074 | 0.040 | 0.07 | |
| *Tyzzerella 3* | rs7019909 | T | C | 0.120 | 0.144 | 0.030 | 1.76E-06 | -0.066 | 0.029 | 0.02 | |
| *Tyzzerella 3* | rs4904512 | T | C | 0.129 | -0.117 | 0.025 | 3.09E-06 | 0.004 | 0.028 | 0.89 | |
| *Tyzzerella 3* | rs6920448 | C | T | 0.093 | -0.141 | 0.031 | 4.15E-06 | 0.038 | 0.033 | 0.26 | |
| *Tyzzerella 3* | rs7333521 | T | C | 0.032 | -0.207 | 0.045 | 4.88E-06 | -0.041 | 0.055 | 0.45 | |
| *Tyzzerella 3* | rs191093 | G | A | 0.107 | 0.159 | 0.035 | 6.76E-06 | 0.010 | 0.031 | 0.74 | |
| *Tyzzerella 3* | rs1232220 | G | T | 0.101 | -0.144 | 0.032 | 7.91E-06 | 0.034 | 0.033 | 0.30 | |
| *Tyzzerella 3* | rs11128780 | C | T | 0.339 | -0.214 | 0.048 | 8.42E-06 | 0.057 | 0.020 | 0.00 | |
| *Tyzzerella 3* | rs10898797 | C | T | 0.109 | 0.122 | 0.027 | 8.85E-06 | 0.057 | 0.031 | 0.06 | |
| *Veillonella* | rs1882878 | A | G | 0.298 | -0.077 | 0.016 | 2.98E-06 | 0.006 | 0.021 | 0.77 | |
| *Veillonella* | rs2013594 | T | C | 0.404 | -0.072 | 0.016 | 3.42E-06 | 0.000 | 0.020 | 0.99 | |
| *Veillonella* | rs62376424 | C | T | 0.305 | -0.076 | 0.016 | 3.65E-06 | 0.036 | 0.021 | 0.08 | |
| *Veillonella* | rs742016 | A | G | 0.334 | -0.069 | 0.015 | 4.66E-06 | -0.003 | 0.020 | 0.89 | |
| *Veillonella* | rs6656807 | A | G | 0.386 | 0.070 | 0.015 | 5.50E-06 | -0.001 | 0.020 | 0.95 | |
| *Veillonella* | rs55807413 | A | G | 0.101 | 0.107 | 0.024 | 5.51E-06 | -0.007 | 0.032 | 0.84 | |
| *Victivallis* | rs56349194 | A | G | 0.129 | -0.159 | 0.032 | 6.26E-07 | -0.005 | 0.029 | 0.87 | |
| *Victivallis* | rs4764863 | G | A | 0.551 | 0.122 | 0.025 | 8.22E-07 | 0.019 | 0.019 | 0.32 | |
| *Victivallis* | rs12512543 | A | C | 0.084 | -0.178 | 0.037 | 2.54E-06 | -0.037 | 0.034 | 0.29 | |
| *Victivallis* | rs4895919 | T | C | 0.480 | -0.117 | 0.025 | 2.75E-06 | 0.019 | 0.019 | 0.32 | |
| *Victivallis* | rs11899949 | G | A | 0.311 | 0.131 | 0.028 | 2.77E-06 | 0.012 | 0.021 | 0.57 | |
| *Victivallis* | rs911666 | T | C | 0.304 | -0.119 | 0.026 | 7.65E-06 | 0.021 | 0.021 | 0.30 | |
| *Victivallis* | rs173120 | T | C | 0.190 | 0.134 | 0.029 | 7.65E-06 | -0.012 | 0.025 | 0.63 | |
| *Victivallis* | rs342302 | A | G | 0.138 | -0.153 | 0.035 | 8.16E-06 | -0.002 | 0.028 | 0.95 | |
| *Victivallis* | rs1882775 | A | G | 0.183 | -0.138 | 0.031 | 8.73E-06 | -0.028 | 0.025 | 0.25 | |
| *Victivallis* | rs2546432 | T | C | 0.457 | -0.111 | 0.025 | 9.93E-06 | -0.036 | 0.019 | 0.06 | |
| MR, Mendelian randomization; PE, preeclampsia-eclampsia; SNP, single nucleotide polymorphism; MAF, minor allele frequency; SE, standard error. | | | | | | | | | | | |

| **Table S2 Full result of MR estimates for the association between gut microbiota and PE.** | | | | | | | | |
| --- | --- | --- | --- | --- | --- | --- | --- | --- |
| **Bacterial taxa (exposure)** | **MR method** | **No. of SNP** | **F-statistic** | **Power** | **OR** | **95% CI** | **P-value** | **q-value** |
| *Actinomyces* | IVW | 7 | 29.82 | 0.04 | 1.07 | 0.80–1.43 | 0.64 | 0.85 |
| *Actinomyces* | MR Egger | 7 |  |  | 1.25 | 0.59–2.66 | 0.58 | 1.00 |
| *Actinomyces* | Weighted median | 7 |  |  | 0.92 | 0.71–1.19 | 0.53 | 1.00 |
| *Actinomyces* | Weighted mode | 7 |  |  | 0.84 | 0.57–1.24 | 0.42 | 0.98 |
| *Actinomyces* | ML | 7 |  |  | 1.08 | 0.90–1.31 | 0.41 | 0.72 |
| *Actinomyces* | cML-MA-BIC | 7 |  |  | 1.07 | 0.88–1.31 | 0.47 | 0.70 |
| *Adlercreutzia* | IVW | 8 | 103.69 | 0.16 | 0.83 | 0.68–1.01 | 0.06 | 0.61 |
| *Adlercreutzia* | MR Egger | 8 |  |  | 0.95 | 0.37–2.45 | 0.92 | 1.00 |
| *Adlercreutzia* | Weighted median | 8 |  |  | 0.77 | 0.59–1.01 | 0.06 | 0.97 |
| *Adlercreutzia* | Weighted mode | 8 |  |  | 0.74 | 0.48–1.14 | 0.21 | 0.98 |
| *Adlercreutzia* | ML | 8 |  |  | 0.82 | 0.67–1.00 | 0.04 | 0.50 |
| *Adlercreutzia* | cML-MA-BIC | 8 |  |  | 0.82 | 0.68–1.00 | 0.05 | 0.39 |
| *Akkermansia* | IVW | 11 | 65.10 | 0.11 | 1.16 | 0.97–1.39 | 0.11 | 0.67 |
| *Akkermansia* | MR Egger | 11 |  |  | 1.99 | 1.08–3.65 | 0.05 | 1.00 |
| *Akkermansia* | Weighted median | 11 |  |  | 1.09 | 0.86–1.39 | 0.46 | 1.00 |
| *Akkermansia* | Weighted mode | 11 |  |  | 1.09 | 0.71–1.66 | 0.70 | 0.98 |
| *Akkermansia* | ML | 11 |  |  | 1.16 | 0.97–1.40 | 0.10 | 0.58 |
| *Akkermansia* | cML-MA-BIC | 11 |  |  | 1.16 | 0.97–1.40 | 0.10 | 0.55 |
| *Alistipes* | IVW | 12 | 21.17 | 0.04 | 0.92 | 0.68–1.23 | 0.57 | 0.85 |
| *Alistipes* | MR Egger | 12 |  |  | 0.93 | 0.21–4.08 | 0.93 | 1.00 |
| *Alistipes* | Weighted median | 12 |  |  | 0.72 | 0.50–1.03 | 0.07 | 0.97 |
| *Alistipes* | Weighted mode | 12 |  |  | 0.62 | 0.29–1.33 | 0.24 | 0.98 |
| *Alistipes* | ML | 12 |  |  | 0.92 | 0.72–1.17 | 0.49 | 0.73 |
| *Alistipes* | cML-MA-BIC | 12 |  |  | 0.91 | 0.71–1.16 | 0.45 | 0.70 |
| *Allisonella* | IVW | 8 | 0.17 | 0.03 | 1.00 | 0.90–1.12 | 1.00 | 0.89 |
| *Allisonella* | MR Egger | 8 |  |  | 1.04 | 0.49–2.19 | 0.93 | 1.00 |
| *Allisonella* | Weighted median | 8 |  |  | 1.02 | 0.88–1.18 | 0.81 | 1.00 |
| *Allisonella* | Weighted mode | 8 |  |  | 1.03 | 0.85–1.25 | 0.76 | 0.98 |
| *Allisonella* | ML | 8 |  |  | 1.00 | 0.89–1.12 | 1.00 | 0.81 |
| *Allisonella* | cML-MA-BIC | 8 |  |  | 1.00 | 0.89–1.12 | 1.00 | 0.81 |
| *Alloprevotella* | IVW | 5 | 9.80 | 0.03 | 0.99 | 0.77–1.26 | 0.91 | 0.87 |
| *Alloprevotella* | MR Egger | 5 |  |  | 0.50 | 0.05–5.27 | 0.60 | 1.00 |
| *Alloprevotella* | Weighted median | 5 |  |  | 1.08 | 0.86–1.37 | 0.50 | 1.00 |
| *Alloprevotella* | Weighted mode | 5 |  |  | 1.15 | 0.84–1.59 | 0.43 | 0.98 |
| *Alloprevotella* | ML | 5 |  |  | 0.98 | 0.85–1.14 | 0.84 | 0.76 |
| *Alloprevotella* | cML-MA-BIC | 5 |  |  | 0.99 | 0.85–1.15 | 0.87 | 0.77 |
| *Anaerofilum* | IVW | 11 | 13.69 | 0.03 | 1.03 | 0.88–1.19 | 0.73 | 0.85 |
| *Anaerofilum* | MR Egger | 11 |  |  | 0.79 | 0.34–1.83 | 0.60 | 1.00 |
| *Anaerofilum* | Weighted median | 11 |  |  | 1.06 | 0.89–1.27 | 0.50 | 1.00 |
| *Anaerofilum* | Weighted mode | 11 |  |  | 1.21 | 0.89–1.64 | 0.25 | 0.98 |
| *Anaerofilum* | ML | 11 |  |  | 1.03 | 0.90–1.17 | 0.67 | 0.76 |
| *Anaerofilum* | cML-MA-BIC | 11 |  |  | 1.03 | 0.91–1.18 | 0.63 | 0.76 |
| *Anaerostipes* | IVW | 13 | 7.18 | 0.03 | 0.97 | 0.76–1.25 | 0.83 | 0.85 |
| *Anaerostipes* | MR Egger | 13 |  |  | 0.58 | 0.23–1.43 | 0.26 | 1.00 |
| *Anaerostipes* | Weighted median | 13 |  |  | 0.88 | 0.65–1.20 | 0.42 | 1.00 |
| *Anaerostipes* | Weighted mode | 13 |  |  | 0.85 | 0.52–1.41 | 0.55 | 0.98 |
| *Anaerostipes* | ML | 13 |  |  | 0.97 | 0.78–1.21 | 0.81 | 0.76 |
| *Anaerostipes* | cML-MA-BIC | 13 |  |  | 0.97 | 0.78–1.21 | 0.81 | 0.77 |
| *Anaerotruncus* | IVW | 12 | 51.78 | 0.10 | 0.86 | 0.69–1.06 | 0.16 | 0.71 |
| *Anaerotruncus* | MR Egger | 12 |  |  | 1.06 | 0.50–2.22 | 0.89 | 1.00 |
| *Anaerotruncus* | Weighted median | 12 |  |  | 0.90 | 0.69–1.19 | 0.47 | 1.00 |
| *Anaerotruncus* | Weighted mode | 12 |  |  | 0.91 | 0.61–1.37 | 0.67 | 0.98 |
| *Anaerotruncus* | ML | 12 |  |  | 0.86 | 0.69–1.07 | 0.16 | 0.63 |
| *Anaerotruncus* | cML-MA-BIC | 12 |  |  | 0.85 | 0.69–1.07 | 0.16 | 0.58 |
| *Bacteroides* | IVW | 7 | 3.46 | 0.03 | 0.99 | 0.76–1.30 | 0.96 | 0.88 |
| *Bacteroides* | MR Egger | 7 |  |  | 0.85 | 0.20–3.60 | 0.83 | 1.00 |
| *Bacteroides* | Weighted median | 7 |  |  | 1.11 | 0.76–1.62 | 0.58 | 1.00 |
| *Bacteroides* | Weighted mode | 7 |  |  | 1.29 | 0.70–2.38 | 0.44 | 0.98 |
| *Bacteroides* | ML | 7 |  |  | 0.99 | 0.75–1.31 | 0.96 | 0.80 |
| *Bacteroides* | cML-MA-BIC | 7 |  |  | 0.99 | 0.75–1.31 | 0.95 | 0.79 |
| *Barnesiella* | IVW | 12 | 41.81 | 0.07 | 0.89 | 0.72–1.09 | 0.26 | 0.81 |
| *Barnesiella* | MR Egger | 12 |  |  | 1.79 | 0.80–3.98 | 0.19 | 1.00 |
| *Barnesiella* | Weighted median | 12 |  |  | 0.98 | 0.74–1.28 | 0.86 | 1.00 |
| *Barnesiella* | Weighted mode | 12 |  |  | 1.10 | 0.69–1.77 | 0.69 | 0.98 |
| *Barnesiella* | ML | 12 |  |  | 0.89 | 0.73–1.09 | 0.26 | 0.64 |
| *Barnesiella* | cML-MA-BIC | 12 |  |  | 0.89 | 0.72–1.09 | 0.24 | 0.61 |
| *Bifidobacterium* | IVW | 13 | 115.25 | 0.48 | 0.76 | 0.64–0.89 | 8.03E-04 | 0.08 |
| *Bifidobacterium* | MR Egger | 13 |  |  | 0.71 | 0.47–1.08 | 0.14 | 1.00 |
| *Bifidobacterium* | Weighted median | 13 |  |  | 0.78 | 0.61–0.98 | 0.04 | 0.97 |
| *Bifidobacterium* | Weighted mode | 13 |  |  | 0.75 | 0.54–1.03 | 0.10 | 0.98 |
| *Bifidobacterium* | ML | 13 |  |  | 0.76 | 0.65–0.90 | 1.29E-03 | 0.07 |
| *Bifidobacterium* | cML-MA-BIC | 13 |  |  | 0.75 | 0.64–0.89 | 9.24E-04 | 0.04 |
| *Bilophila* | IVW | 13 | 7.89 | 0.03 | 1.02 | 0.85–1.23 | 0.82 | 0.85 |
| *Bilophila* | MR Egger | 13 |  |  | 0.71 | 0.29–1.78 | 0.48 | 1.00 |
| *Bilophila* | Weighted median | 13 |  |  | 0.95 | 0.73–1.23 | 0.69 | 1.00 |
| *Bilophila* | Weighted mode | 13 |  |  | 0.89 | 0.58–1.36 | 0.60 | 0.98 |
| *Bilophila* | ML | 13 |  |  | 1.02 | 0.85–1.24 | 0.81 | 0.76 |
| *Bilophila* | cML-MA-BIC | 13 |  |  | 1.02 | 0.85–1.24 | 0.82 | 0.77 |
| *Blautia* | IVW | 12 | 3.78 | 0.03 | 0.99 | 0.79–1.23 | 0.92 | 0.87 |
| *Blautia* | MR Egger | 12 |  |  | 0.99 | 0.56–1.74 | 0.97 | 1.00 |
| *Blautia* | Weighted median | 12 |  |  | 1.00 | 0.75–1.33 | 0.99 | 1.00 |
| *Blautia* | Weighted mode | 12 |  |  | 0.96 | 0.62–1.49 | 0.86 | 0.98 |
| *Blautia* | ML | 12 |  |  | 0.99 | 0.79–1.23 | 0.92 | 0.80 |
| *Blautia* | cML-MA-BIC | 12 |  |  | 0.99 | 0.79–1.23 | 0.92 | 0.78 |
| *Butyricicoccus* | IVW | 8 | 15.17 | 0.03 | 1.03 | 0.82–1.30 | 0.78 | 0.85 |
| *Butyricicoccus* | MR Egger | 8 |  |  | 1.00 | 0.64–1.56 | 0.99 | 1.00 |
| *Butyricicoccus* | Weighted median | 8 |  |  | 1.14 | 0.84–1.53 | 0.40 | 1.00 |
| *Butyricicoccus* | Weighted mode | 8 |  |  | 1.15 | 0.79–1.66 | 0.50 | 0.98 |
| *Butyricicoccus* | ML | 8 |  |  | 1.03 | 0.82–1.30 | 0.79 | 0.76 |
| *Butyricicoccus* | cML-MA-BIC | 8 |  |  | 1.03 | 0.82–1.30 | 0.78 | 0.77 |
| *Butyricimonas* | IVW | 13 | 27.31 | 0.04 | 1.07 | 0.90–1.27 | 0.42 | 0.83 |
| *Butyricimonas* | MR Egger | 13 |  |  | 1.25 | 0.68–2.31 | 0.48 | 1.00 |
| *Butyricimonas* | Weighted median | 13 |  |  | 1.25 | 0.98–1.60 | 0.07 | 0.97 |
| *Butyricimonas* | Weighted mode | 13 |  |  | 1.31 | 0.87–1.98 | 0.22 | 0.98 |
| *Butyricimonas* | ML | 13 |  |  | 1.08 | 0.90–1.28 | 0.41 | 0.72 |
| *Butyricimonas* | cML-MA-BIC | 13 |  |  | 1.08 | 0.90–1.29 | 0.41 | 0.68 |
| *Butyrivibrio* | IVW | 14 | 30.84 | 0.04 | 0.94 | 0.83–1.06 | 0.33 | 0.83 |
| *Butyrivibrio* | MR Egger | 14 |  |  | 1.33 | 0.78–2.26 | 0.32 | 1.00 |
| *Butyrivibrio* | Weighted median | 14 |  |  | 0.94 | 0.82–1.08 | 0.38 | 1.00 |
| *Butyrivibrio* | Weighted mode | 14 |  |  | 0.87 | 0.70–1.07 | 0.22 | 0.98 |
| *Butyrivibrio* | ML | 14 |  |  | 0.94 | 0.86–1.03 | 0.18 | 0.64 |
| *Butyrivibrio* | cML-MA-BIC | 14 |  |  | 0.94 | 0.85–1.03 | 0.15 | 0.58 |
| *Candidatus Soleaferrea* | IVW | 9 | 54.31 | 0.05 | 0.92 | 0.80–1.07 | 0.27 | 0.81 |
| *Candidatus Soleaferrea* | MR Egger | 9 |  |  | 0.57 | 0.13–2.54 | 0.48 | 1.00 |
| *Candidatus Soleaferrea* | Weighted median | 9 |  |  | 0.91 | 0.75–1.09 | 0.30 | 1.00 |
| *Candidatus Soleaferrea* | Weighted mode | 9 |  |  | 0.90 | 0.68–1.19 | 0.48 | 0.98 |
| *Candidatus Soleaferrea* | ML | 9 |  |  | 0.92 | 0.79–1.07 | 0.27 | 0.64 |
| *Candidatus Soleaferrea* | cML-MA-BIC | 9 |  |  | 0.92 | 0.79–1.07 | 0.26 | 0.61 |
| *Catenibacterium* | IVW | 4 | 65.95 | 0.04 | 1.06 | 0.88–1.27 | 0.55 | 0.85 |
| *Catenibacterium* | MR Egger | 4 |  |  | 0.92 | 0.05–15.92 | 0.96 | 1.00 |
| *Catenibacterium* | Weighted median | 4 |  |  | 1.01 | 0.82–1.23 | 0.95 | 1.00 |
| *Catenibacterium* | Weighted mode | 4 |  |  | 0.93 | 0.67–1.31 | 0.71 | 0.98 |
| *Catenibacterium* | ML | 4 |  |  | 1.06 | 0.89–1.26 | 0.50 | 0.73 |
| *Catenibacterium* | cML-MA-BIC | 4 |  |  | 1.06 | 0.89–1.26 | 0.50 | 0.71 |
| *Christensenellaceae (R7 group)* | IVW | 8 | 18.18 | 0.03 | 1.05 | 0.80–1.37 | 0.74 | 0.85 |
| *Christensenellaceae (R7 group)* | MR Egger | 8 |  |  | 0.84 | 0.32–2.17 | 0.73 | 1.00 |
| *Christensenellaceae (R7 group)* | Weighted median | 8 |  |  | 1.07 | 0.75–1.52 | 0.72 | 1.00 |
| *Christensenellaceae (R7 group)* | Weighted mode | 8 |  |  | 1.11 | 0.63–1.95 | 0.72 | 0.98 |
| *Christensenellaceae (R7 group)* | ML | 8 |  |  | 1.05 | 0.80–1.38 | 0.74 | 0.76 |
| *Christensenellaceae (R7 group)* | cML-MA-BIC | 8 |  |  | 1.05 | 0.80–1.38 | 0.74 | 0.77 |
| *Clostridium (innocuum group)* | IVW | 7 | 10.32 | 0.03 | 1.01 | 0.89–1.15 | 0.87 | 0.86 |
| *Clostridium (innocuum group)* | MR Egger | 7 |  |  | 1.83 | 0.96–3.49 | 0.12 | 1.00 |
| *Clostridium (innocuum group)* | Weighted median | 7 |  |  | 1.01 | 0.85–1.19 | 0.93 | 1.00 |
| *Clostridium (innocuum group)* | Weighted mode | 7 |  |  | 0.98 | 0.78–1.23 | 0.88 | 0.98 |
| *Clostridium (innocuum group)* | ML | 7 |  |  | 1.01 | 0.89–1.16 | 0.87 | 0.78 |
| *Clostridium (innocuum group)* | cML-MA-BIC | 7 |  |  | 1.01 | 0.89–1.16 | 0.86 | 0.77 |
| *Clostridiumsensustricto 1* | IVW | 7 | 17.06 | 0.03 | 1.03 | 0.83–1.28 | 0.79 | 0.85 |
| *Clostridiumsensustricto 1* | MR Egger | 7 |  |  | 1.67 | 0.96–2.90 | 0.13 | 1.00 |
| *Clostridiumsensustricto 1* | Weighted median | 7 |  |  | 1.04 | 0.78–1.37 | 0.81 | 1.00 |
| *Clostridiumsensustricto 1* | Weighted mode | 7 |  |  | 1.04 | 0.75–1.44 | 0.83 | 0.98 |
| *Clostridiumsensustricto 1* | ML | 7 |  |  | 1.03 | 0.83–1.28 | 0.79 | 0.76 |
| *Clostridiumsensustricto 1* | cML-MA-BIC | 7 |  |  | 1.03 | 0.83–1.28 | 0.78 | 0.77 |
| *Collinsella* | IVW | 9 | 104.60 | 0.30 | 0.77 | 0.60–0.98 | 0.03 | 0.61 |
| *Collinsella* | MR Egger | 9 |  |  | 1.50 | 0.60–3.75 | 0.42 | 1.00 |
| *Collinsella* | Weighted median | 9 |  |  | 0.71 | 0.51–1.01 | 0.05 | 0.97 |
| *Collinsella* | Weighted mode | 9 |  |  | 0.65 | 0.38–1.12 | 0.16 | 0.98 |
| *Collinsella* | ML | 9 |  |  | 0.77 | 0.60–0.99 | 0.04 | 0.50 |
| *Collinsella* | cML-MA-BIC | 9 |  |  | 0.76 | 0.59–0.98 | 0.03 | 0.39 |
| *Coprobacter* | IVW | 10 | 0.28 | 0.03 | 1.00 | 0.80–1.25 | 0.99 | 0.89 |
| *Coprobacter* | MR Egger | 10 |  |  | 0.69 | 0.29–1.61 | 0.42 | 1.00 |
| *Coprobacter* | Weighted median | 10 |  |  | 1.00 | 0.81–1.24 | 0.99 | 1.00 |
| *Coprobacter* | Weighted mode | 10 |  |  | 1.04 | 0.76–1.42 | 0.80 | 0.98 |
| *Coprobacter* | ML | 10 |  |  | 1.00 | 0.86–1.17 | 0.99 | 0.81 |
| *Coprobacter* | cML-MA-BIC | 10 |  |  | 0.99 | 0.84–1.16 | 0.88 | 0.77 |
| *Coprococcus 1* | IVW | 11 | 3.37 | 0.03 | 0.99 | 0.81–1.21 | 0.93 | 0.87 |
| *Coprococcus 1* | MR Egger | 11 |  |  | 1.44 | 0.88–2.35 | 0.18 | 1.00 |
| *Coprococcus 1* | Weighted median | 11 |  |  | 1.10 | 0.84–1.45 | 0.47 | 1.00 |
| *Coprococcus 1* | Weighted mode | 11 |  |  | 1.17 | 0.79–1.73 | 0.46 | 0.98 |
| *Coprococcus 1* | ML | 11 |  |  | 0.99 | 0.81–1.22 | 0.94 | 0.80 |
| *Coprococcus 1* | cML-MA-BIC | 11 |  |  | 0.99 | 0.81–1.21 | 0.93 | 0.78 |
| *Coprococcus 2* | IVW | 8 | 84.61 | 0.12 | 0.84 | 0.68–1.05 | 0.13 | 0.67 |
| *Coprococcus 2* | MR Egger | 8 |  |  | 0.72 | 0.13–4.04 | 0.72 | 1.00 |
| *Coprococcus 2* | Weighted median | 8 |  |  | 0.87 | 0.66–1.14 | 0.31 | 1.00 |
| *Coprococcus 2* | Weighted mode | 8 |  |  | 1.00 | 0.63–1.59 | 1.00 | 1.00 |
| *Coprococcus 2* | ML | 8 |  |  | 0.84 | 0.67–1.05 | 0.13 | 0.58 |
| *Coprococcus 2* | cML-MA-BIC | 8 |  |  | 0.84 | 0.67–1.05 | 0.13 | 0.55 |
| *Coprococcus 3* | IVW | 9 | 12.10 | 0.03 | 0.96 | 0.72–1.30 | 0.81 | 0.85 |
| *Coprococcus 3* | MR Egger | 9 |  |  | 1.07 | 0.18–6.38 | 0.94 | 1.00 |
| *Coprococcus 3* | Weighted median | 9 |  |  | 0.90 | 0.63–1.27 | 0.53 | 1.00 |
| *Coprococcus 3* | Weighted mode | 9 |  |  | 0.82 | 0.48–1.38 | 0.47 | 0.98 |
| *Coprococcus 3* | ML | 9 |  |  | 0.96 | 0.74–1.24 | 0.76 | 0.76 |
| *Coprococcus 3* | cML-MA-BIC | 9 |  |  | 0.95 | 0.73–1.24 | 0.72 | 0.77 |
| *Defluviitaleaceae (UCG011)* | IVW | 9 | 36.46 | 0.04 | 0.93 | 0.78–1.12 | 0.46 | 0.85 |
| *Defluviitaleaceae (UCG011)* | MR Egger | 9 |  |  | 0.71 | 0.37–1.36 | 0.34 | 1.00 |
| *Defluviitaleaceae (UCG011)* | Weighted median | 9 |  |  | 0.96 | 0.75–1.21 | 0.70 | 1.00 |
| *Defluviitaleaceae (UCG011)* | Weighted mode | 9 |  |  | 0.87 | 0.61–1.24 | 0.47 | 0.98 |
| *Defluviitaleaceae (UCG011)* | ML | 9 |  |  | 0.93 | 0.78–1.12 | 0.45 | 0.73 |
| *Defluviitaleaceae (UCG011)* | cML-MA-BIC | 9 |  |  | 0.93 | 0.78–1.12 | 0.45 | 0.70 |
| *Desulfovibrio* | IVW | 10 | 65.46 | 0.09 | 1.14 | 0.96–1.36 | 0.14 | 0.68 |
| *Desulfovibrio* | MR Egger | 10 |  |  | 0.91 | 0.54–1.53 | 0.74 | 1.00 |
| *Desulfovibrio* | Weighted median | 10 |  |  | 1.04 | 0.82–1.32 | 0.75 | 1.00 |
| *Desulfovibrio* | Weighted mode | 10 |  |  | 0.99 | 0.71–1.37 | 0.93 | 0.99 |
| *Desulfovibrio* | ML | 10 |  |  | 1.15 | 0.96–1.38 | 0.13 | 0.58 |
| *Desulfovibrio* | cML-MA-BIC | 10 |  |  | 1.15 | 0.96–1.37 | 0.14 | 0.56 |
| *Dialister* | IVW | 11 | 1.19 | 0.03 | 1.00 | 0.80–1.26 | 0.98 | 0.88 |
| *Dialister* | MR Egger | 11 |  |  | 1.80 | 0.73–4.42 | 0.23 | 1.00 |
| *Dialister* | Weighted median | 11 |  |  | 1.10 | 0.84–1.44 | 0.49 | 1.00 |
| *Dialister* | Weighted mode | 11 |  |  | 1.18 | 0.77–1.82 | 0.46 | 0.98 |
| *Dialister* | ML | 11 |  |  | 1.00 | 0.83–1.22 | 0.97 | 0.80 |
| *Dialister* | cML-MA-BIC | 11 |  |  | 1.01 | 0.83–1.23 | 0.94 | 0.79 |
| *Dorea* | IVW | 10 | 24.59 | 0.04 | 1.08 | 0.83–1.39 | 0.58 | 0.85 |
| *Dorea* | MR Egger | 10 |  |  | 0.80 | 0.39–1.65 | 0.56 | 1.00 |
| *Dorea* | Weighted median | 10 |  |  | 1.08 | 0.75–1.56 | 0.66 | 1.00 |
| *Dorea* | Weighted mode | 10 |  |  | 1.31 | 0.71–2.43 | 0.41 | 0.98 |
| *Dorea* | ML | 10 |  |  | 1.08 | 0.84–1.39 | 0.54 | 0.75 |
| *Dorea* | cML-MA-BIC | 10 |  |  | 1.10 | 0.93–1.30 | 0.27 | 0.61 |
| *Eggerthella* | IVW | 10 | 15.36 | 0.03 | 0.98 | 0.85–1.12 | 0.73 | 0.85 |
| *Eggerthella* | MR Egger | 10 |  |  | 0.94 | 0.47–1.89 | 0.87 | 1.00 |
| *Eggerthella* | Weighted median | 10 |  |  | 0.95 | 0.80–1.14 | 0.61 | 1.00 |
| *Eggerthella* | Weighted mode | 10 |  |  | 0.85 | 0.61–1.20 | 0.38 | 0.98 |
| *Eggerthella* | ML | 10 |  |  | 0.97 | 0.85–1.12 | 0.70 | 0.76 |
| *Eggerthella* | cML-MA-BIC | 10 |  |  | 1.10 | 0.93–1.30 | 0.27 | 0.61 |
| *Eisenbergiella* | IVW | 11 | 11.95 | 0.03 | 0.98 | 0.86–1.11 | 0.77 | 0.85 |
| *Eisenbergiella* | MR Egger | 11 |  |  | 0.91 | 0.35–2.34 | 0.84 | 1.00 |
| *Eisenbergiella* | Weighted median | 11 |  |  | 1.00 | 0.85–1.18 | 0.99 | 1.00 |
| *Eisenbergiella* | Weighted mode | 11 |  |  | 1.07 | 0.82–1.39 | 0.64 | 0.98 |
| *Eisenbergiella* | ML | 11 |  |  | 0.98 | 0.86–1.12 | 0.77 | 0.76 |
| *Eisenbergiella* | cML-MA-BIC | 11 |  |  | 0.98 | 0.86–1.12 | 0.77 | 0.77 |
| *Enterorhabdus* | IVW | 6 | 194.91 | 0.39 | 0.76 | 0.62–0.93 | 8.76E-03 | 0.23 |
| *Enterorhabdus* | MR Egger | 6 |  |  | 0.62 | 0.36–1.07 | 0.16 | 1.00 |
| *Enterorhabdus* | Weighted median | 6 |  |  | 0.76 | 0.57–1.01 | 0.06 | 0.97 |
| *Enterorhabdus* | Weighted mode | 6 |  |  | 0.77 | 0.51–1.16 | 0.27 | 0.98 |
| *Enterorhabdus* | ML | 6 |  |  | 0.75 | 0.61–0.93 | 8.78E-03 | 0.17 |
| *Enterorhabdus* | cML-MA-BIC | 6 |  |  | 0.76 | 0.61–0.93 | 9.40E-03 | 0.15 |
| *Erysipelatoclostridium* | IVW | 15 | 33.76 | 0.05 | 0.92 | 0.80–1.06 | 0.25 | 0.81 |
| *Erysipelatoclostridium* | MR Egger | 15 |  |  | 1.02 | 0.58–1.77 | 0.95 | 1.00 |
| *Erysipelatoclostridium* | Weighted median | 15 |  |  | 0.88 | 0.73–1.06 | 0.18 | 1.00 |
| *Erysipelatoclostridium* | Weighted mode | 15 |  |  | 0.90 | 0.65–1.25 | 0.55 | 0.98 |
| *Erysipelatoclostridium* | ML | 15 |  |  | 0.92 | 0.80–1.07 | 0.27 | 0.64 |
| *Erysipelatoclostridium* | cML-MA-BIC | 15 |  |  | 0.92 | 0.79–1.06 | 0.25 | 0.61 |
| *Erysipelotrichaceae (UCG003)* | IVW | 16 | 29.98 | 0.05 | 1.09 | 0.93–1.29 | 0.28 | 0.81 |
| *Erysipelotrichaceae (UCG003)* | MR Egger | 16 |  |  | 1.09 | 0.70–1.70 | 0.70 | 1.00 |
| *Erysipelotrichaceae (UCG003)* | Weighted median | 16 |  |  | 1.13 | 0.90–1.40 | 0.29 | 1.00 |
| *Erysipelotrichaceae (UCG003)* | Weighted mode | 16 |  |  | 1.20 | 0.83–1.72 | 0.34 | 0.98 |
| *Erysipelotrichaceae (UCG003)* | ML | 16 |  |  | 1.10 | 0.93–1.30 | 0.27 | 0.64 |
| *Erysipelotrichaceae (UCG003)* | cML-MA-BIC | 16 |  |  | 1.10 | 0.93–1.30 | 0.27 | 0.61 |
| *Escherichia Shigella* | IVW | 10 | 54.96 | 0.08 | 0.88 | 0.71–1.08 | 0.22 | 0.81 |
| *Escherichia Shigella* | MR Egger | 10 |  |  | 0.58 | 0.30–1.11 | 0.14 | 1.00 |
| *Escherichia Shigella* | Weighted median | 10 |  |  | 0.90 | 0.68–1.18 | 0.45 | 1.00 |
| *Escherichia Shigella* | Weighted mode | 10 |  |  | 0.88 | 0.57–1.37 | 0.59 | 0.98 |
| *Escherichia Shigella* | ML | 10 |  |  | 0.88 | 0.71–1.08 | 0.22 | 0.64 |
| *Escherichia Shigella* | cML-MA-BIC | 10 |  |  | 0.87 | 0.71–1.08 | 0.21 | 0.61 |
| *Eubacterium (brachy group)* | IVW | 10 | 16.42 | 0.03 | 0.98 | 0.87–1.10 | 0.71 | 0.85 |
| *Eubacterium (brachy group)* | MR Egger | 10 |  |  | 1.12 | 0.71–1.79 | 0.64 | 1.00 |
| *Eubacterium (brachy group)* | Weighted median | 10 |  |  | 1.00 | 0.86–1.17 | 0.99 | 1.00 |
| *Eubacterium (brachy group)* | Weighted mode | 10 |  |  | 1.01 | 0.82–1.26 | 0.90 | 0.98 |
| *Eubacterium (brachy group)* | ML | 10 |  |  | 0.98 | 0.87–1.10 | 0.71 | 0.76 |
| *Eubacterium (brachy group)* | cML-MA-BIC | 10 |  |  | 0.98 | 0.87–1.10 | 0.71 | 0.77 |
| *Eubacterium (coprostanoligenes group)* | IVW | 14 | 21.70 | 0.04 | 1.07 | 0.88–1.32 | 0.49 | 0.85 |
| *Eubacterium (coprostanoligenes group)* | MR Egger | 14 |  |  | 1.23 | 0.55–2.75 | 0.62 | 1.00 |
| *Eubacterium (coprostanoligenes group)* | Weighted median | 14 |  |  | 1.10 | 0.84–1.43 | 0.50 | 1.00 |
| *Eubacterium (coprostanoligenes group)* | Weighted mode | 14 |  |  | 1.16 | 0.73–1.83 | 0.54 | 0.98 |
| *Eubacterium (coprostanoligenes group)* | ML | 14 |  |  | 1.08 | 0.88–1.32 | 0.48 | 0.73 |
| *Eubacterium (coprostanoligenes group)* | cML-MA-BIC | 14 |  |  | 1.08 | 0.87–1.32 | 0.49 | 0.71 |
| *Eubacterium (eligens group)* | IVW | 6 | 53.93 | 0.05 | 0.90 | 0.69–1.19 | 0.47 | 0.85 |
| *Eubacterium (eligens group)* | MR Egger | 6 |  |  | 0.91 | 0.32–2.54 | 0.86 | 1.00 |
| *Eubacterium (eligens group)* | Weighted median | 6 |  |  | 0.95 | 0.68–1.34 | 0.77 | 1.00 |
| *Eubacterium (eligens group)* | Weighted mode | 6 |  |  | 1.01 | 0.62–1.66 | 0.96 | 1.00 |
| *Eubacterium (eligens group)* | ML | 6 |  |  | 0.90 | 0.68–1.19 | 0.46 | 0.73 |
| *Eubacterium (eligens group)* | cML-MA-BIC | 6 |  |  | 0.90 | 0.68–1.19 | 0.47 | 0.70 |
| *Eubacterium (fissicatena group)* | IVW | 9 | 41.82 | 0.04 | 1.05 | 0.93–1.19 | 0.40 | 0.83 |
| *Eubacterium (fissicatena group)* | MR Egger | 9 |  |  | 1.29 | 0.68–2.45 | 0.46 | 1.00 |
| *Eubacterium (fissicatena group)* | Weighted median | 9 |  |  | 1.06 | 0.90–1.24 | 0.50 | 1.00 |
| *Eubacterium (fissicatena group)* | Weighted mode | 9 |  |  | 1.08 | 0.85–1.38 | 0.55 | 0.98 |
| *Eubacterium (fissicatena group)* | ML | 9 |  |  | 1.06 | 0.93–1.20 | 0.39 | 0.72 |
| *Eubacterium (fissicatena group)* | cML-MA-BIC | 9 |  |  | 1.06 | 0.93–1.20 | 0.39 | 0.68 |
| *Eubacterium (hallii group)* | IVW | 15 | 5.40 | 0.03 | 0.99 | 0.85–1.15 | 0.86 | 0.86 |
| *Eubacterium (hallii group)* | MR Egger | 15 |  |  | 0.82 | 0.60–1.14 | 0.26 | 1.00 |
| *Eubacterium (hallii group)* | Weighted median | 15 |  |  | 0.98 | 0.80–1.21 | 0.88 | 1.00 |
| *Eubacterium (hallii group)* | Weighted mode | 15 |  |  | 1.01 | 0.75–1.35 | 0.96 | 1.00 |
| *Eubacterium (hallii group)* | ML | 15 |  |  | 0.99 | 0.84–1.15 | 0.85 | 0.77 |
| *Eubacterium (hallii group)* | cML-MA-BIC | 15 |  |  | 0.99 | 0.84–1.15 | 0.86 | 0.77 |
| *Eubacterium (nodatum group)* | IVW | 11 | 3.03 | 0.03 | 0.99 | 0.85–1.16 | 0.94 | 0.87 |
| *Eubacterium (nodatum group)* | MR Egger | 11 |  |  | 1.71 | 0.92–3.17 | 0.12 | 1.00 |
| *Eubacterium (nodatum group)* | Weighted median | 11 |  |  | 1.01 | 0.86–1.18 | 0.92 | 1.00 |
| *Eubacterium (nodatum group)* | Weighted mode | 11 |  |  | 1.00 | 0.80–1.25 | 0.98 | 1.00 |
| *Eubacterium (nodatum group)* | ML | 11 |  |  | 0.99 | 0.89–1.10 | 0.91 | 0.80 |
| *Eubacterium (nodatum group)* | cML-MA-BIC | 11 |  |  | 1.01 | 0.90–1.13 | 0.88 | 0.77 |
| *Eubacterium (oxidoreducens group)* | IVW | 5 | 60.82 | 0.04 | 0.94 | 0.78–1.13 | 0.49 | 0.85 |
| *Eubacterium (oxidoreducens group)* | MR Egger | 5 |  |  | 0.94 | 0.47–1.88 | 0.88 | 1.00 |
| *Eubacterium (oxidoreducens group)* | Weighted median | 5 |  |  | 0.92 | 0.73–1.15 | 0.47 | 1.00 |
| *Eubacterium (oxidoreducens group)* | Weighted mode | 5 |  |  | 0.91 | 0.66–1.25 | 0.59 | 0.98 |
| *Eubacterium (oxidoreducens group)* | ML | 5 |  |  | 0.94 | 0.78–1.13 | 0.49 | 0.73 |
| *Eubacterium (oxidoreducens group)* | cML-MA-BIC | 5 |  |  | 0.75 | 0.63–0.90 | 2.48E-03 | 0.05 |
| *Eubacterium (rectale group)* | IVW | 8 | 46.47 | 0.06 | 0.89 | 0.69–1.16 | 0.40 | 0.83 |
| *Eubacterium (rectale group)* | MR Egger | 8 |  |  | 1.88 | 0.74–4.78 | 0.23 | 1.00 |
| *Eubacterium (rectale group)* | Weighted median | 8 |  |  | 1.04 | 0.73–1.47 | 0.83 | 1.00 |
| *Eubacterium (rectale group)* | Weighted mode | 8 |  |  | 1.13 | 0.66–1.93 | 0.67 | 0.98 |
| *Eubacterium (rectale group)* | ML | 8 |  |  | 0.89 | 0.68–1.16 | 0.38 | 0.72 |
| *Eubacterium (rectale group)* | cML-MA-BIC | 8 |  |  | 0.89 | 0.68–1.16 | 0.38 | 0.68 |
| *Eubacterium (ruminantium group)* | IVW | 18 | 20.10 | 0.04 | 0.95 | 0.83–1.08 | 0.42 | 0.83 |
| *Eubacterium (ruminantium group)* | MR Egger | 18 |  |  | 1.46 | 0.99–2.13 | 0.07 | 1.00 |
| *Eubacterium (ruminantium group)* | Weighted median | 18 |  |  | 1.00 | 0.84–1.18 | 0.98 | 1.00 |
| *Eubacterium (ruminantium group)* | Weighted mode | 18 |  |  | 1.06 | 0.83–1.37 | 0.63 | 0.98 |
| *Eubacterium (ruminantium group)* | ML | 18 |  |  | 0.95 | 0.84–1.06 | 0.36 | 0.72 |
| *Eubacterium (ruminantium group)* | cML-MA-BIC | 18 |  |  | 0.95 | 0.84–1.07 | 0.41 | 0.68 |
| *Eubacterium (ventriosum group)* | IVW | 15 | 90.27 | 0.45 | 0.76 | 0.63–0.91 | 2.43E-03 | 0.13 |
| *Eubacterium (ventriosum group)* | MR Egger | 15 |  |  | 0.47 | 0.21–1.03 | 0.08 | 1.00 |
| *Eubacterium (ventriosum group)* | Weighted median | 15 |  |  | 0.81 | 0.63–1.04 | 0.10 | 1.00 |
| *Eubacterium (ventriosum group)* | Weighted mode | 15 |  |  | 0.82 | 0.53–1.26 | 0.38 | 0.98 |
| *Eubacterium (ventriosum group)* | ML | 15 |  |  | 0.76 | 0.63–0.91 | 3.05E-03 | 0.07 |
| *Eubacterium (ventriosum group)* | cML-MA-BIC | 15 |  |  | 0.75 | 0.63–0.90 | 2.48E-03 | 0.05 |
| *Eubacterium (xylanophilum group)* | IVW | 9 | 24.67 | 0.03 | 0.95 | 0.78–1.16 | 0.62 | 0.85 |
| *Eubacterium (xylanophilum group)* | MR Egger | 9 |  |  | 0.87 | 0.48–1.57 | 0.66 | 1.00 |
| *Eubacterium (xylanophilum group)* | Weighted median | 9 |  |  | 0.89 | 0.69–1.16 | 0.40 | 1.00 |
| *Eubacterium (xylanophilum group)* | Weighted mode | 9 |  |  | 0.90 | 0.62–1.30 | 0.58 | 0.98 |
| *Eubacterium (xylanophilum group)* | ML | 9 |  |  | 0.95 | 0.78–1.16 | 0.62 | 0.76 |
| *Eubacterium (xylanophilum group)* | cML-MA-BIC | 9 |  |  | 0.95 | 0.78–1.16 | 0.62 | 0.76 |
| *Faecalibacterium* | IVW | 10 | 44.84 | 0.06 | 1.11 | 0.91–1.34 | 0.31 | 0.83 |
| *Faecalibacterium* | MR Egger | 10 |  |  | 0.86 | 0.60–1.22 | 0.41 | 1.00 |
| *Faecalibacterium* | Weighted median | 10 |  |  | 1.04 | 0.81–1.33 | 0.78 | 1.00 |
| *Faecalibacterium* | Weighted mode | 10 |  |  | 1.02 | 0.78–1.34 | 0.89 | 0.98 |
| *Faecalibacterium* | ML | 10 |  |  | 1.11 | 0.92–1.34 | 0.28 | 0.64 |
| *Faecalibacterium* | cML-MA-BIC | 10 |  |  | 1.11 | 0.91–1.34 | 0.30 | 0.62 |
| *Family XIII (AD3011 group)* | IVW | 13 | 61.86 | 0.14 | 0.84 | 0.70–1.01 | 0.07 | 0.61 |
| *Family XIII (AD3011 group)* | MR Egger | 13 |  |  | 1.30 | 0.53–3.14 | 0.58 | 1.00 |
| *Family XIII (AD3011 group)* | Weighted median | 13 |  |  | 0.86 | 0.66–1.10 | 0.23 | 1.00 |
| *Family XIII (AD3011 group)* | Weighted mode | 13 |  |  | 0.86 | 0.57–1.30 | 0.49 | 0.98 |
| *Family XIII (AD3011 group)* | ML | 13 |  |  | 0.84 | 0.69–1.02 | 0.07 | 0.51 |
| *Family XIII (AD3011 group)* | cML-MA-BIC | 13 |  |  | 0.84 | 0.69–1.01 | 0.07 | 0.41 |
| *Family XIII (UCG001)* | IVW | 8 | 27.23 | 0.04 | 0.94 | 0.74–1.19 | 0.62 | 0.85 |
| *Family XIII (UCG001)* | MR Egger | 8 |  |  | 1.02 | 0.46–2.25 | 0.96 | 1.00 |
| *Family XIII (UCG001)* | Weighted median | 8 |  |  | 1.04 | 0.77–1.41 | 0.79 | 1.00 |
| *Family XIII (UCG001)* | Weighted mode | 8 |  |  | 1.12 | 0.73–1.73 | 0.62 | 0.98 |
| *Family XIII (UCG001)* | ML | 8 |  |  | 0.94 | 0.75–1.18 | 0.59 | 0.76 |
| *Family XIII (UCG001)* | cML-MA-BIC | 8 |  |  | 0.86 | 0.74–1.00 | 0.05 | 0.39 |
| *Flavonifractor* | IVW | 4 | 63.48 | 0.04 | 1.09 | 0.82–1.45 | 0.57 | 0.85 |
| *Flavonifractor* | MR Egger | 4 |  |  | 0.78 | 0.17–3.52 | 0.77 | 1.00 |
| *Flavonifractor* | Weighted median | 4 |  |  | 1.08 | 0.77–1.50 | 0.66 | 1.00 |
| *Flavonifractor* | Weighted mode | 4 |  |  | 1.03 | 0.66–1.62 | 0.89 | 0.98 |
| *Flavonifractor* | ML | 4 |  |  | 1.09 | 0.82–1.45 | 0.57 | 0.76 |
| *Flavonifractor* | cML-MA-BIC | 4 |  |  | 1.09 | 0.81–1.45 | 0.57 | 0.76 |
| *Fusicatenibacter* | IVW | 18 | 15.73 | 0.04 | 0.94 | 0.79–1.13 | 0.52 | 0.85 |
| *Fusicatenibacter* | MR Egger | 18 |  |  | 0.66 | 0.34–1.27 | 0.23 | 1.00 |
| *Fusicatenibacter* | Weighted median | 18 |  |  | 0.89 | 0.70–1.13 | 0.33 | 1.00 |
| *Fusicatenibacter* | Weighted mode | 18 |  |  | 0.76 | 0.52–1.12 | 0.18 | 0.98 |
| *Fusicatenibacter* | ML | 18 |  |  | 0.95 | 0.79–1.13 | 0.54 | 0.75 |
| *Fusicatenibacter* | cML-MA-BIC | 18 |  |  | 0.94 | 0.78–1.13 | 0.50 | 0.71 |
| *Gordonibacter* | IVW | 10 | 24.22 | 0.03 | 1.03 | 0.92–1.15 | 0.59 | 0.85 |
| *Gordonibacter* | MR Egger | 10 |  |  | 1.41 | 0.91–2.20 | 0.16 | 1.00 |
| *Gordonibacter* | Weighted median | 10 |  |  | 1.04 | 0.90–1.21 | 0.58 | 1.00 |
| *Gordonibacter* | Weighted mode | 10 |  |  | 1.10 | 0.88–1.37 | 0.43 | 0.98 |
| *Gordonibacter* | ML | 10 |  |  | 1.03 | 0.92–1.16 | 0.57 | 0.76 |
| *Gordonibacter* | cML-MA-BIC | 10 |  |  | 1.03 | 0.92–1.16 | 0.57 | 0.76 |
| *Haemophilus* | IVW | 9 | 7.99 | 0.03 | 0.98 | 0.79–1.23 | 0.87 | 0.86 |
| *Haemophilus* | MR Egger | 9 |  |  | 1.15 | 0.69–1.92 | 0.62 | 1.00 |
| *Haemophilus* | Weighted median | 9 |  |  | 1.15 | 0.91–1.45 | 0.25 | 1.00 |
| *Haemophilus* | Weighted mode | 9 |  |  | 1.20 | 0.91–1.59 | 0.24 | 0.98 |
| *Haemophilus* | ML | 9 |  |  | 0.98 | 0.83–1.15 | 0.81 | 0.76 |
| *Haemophilus* | cML-MA-BIC | 9 |  |  | 0.98 | 0.83–1.16 | 0.86 | 0.77 |
| *Holdemanella* | IVW | 11 | 40.81 | 0.05 | 1.09 | 0.93–1.27 | 0.31 | 0.83 |
| *Holdemanella* | MR Egger | 11 |  |  | 1.11 | 0.69–1.79 | 0.68 | 1.00 |
| *Holdemanella* | Weighted median | 11 |  |  | 1.05 | 0.86–1.28 | 0.61 | 1.00 |
| *Holdemanella* | Weighted mode | 11 |  |  | 0.87 | 0.62–1.22 | 0.44 | 0.98 |
| *Holdemanella* | ML | 11 |  |  | 1.09 | 0.95–1.26 | 0.21 | 0.64 |
| *Holdemanella* | cML-MA-BIC | 11 |  |  | 1.09 | 0.95–1.26 | 0.23 | 0.61 |
| *Holdemania* | IVW | 14 | 62.45 | 0.12 | 0.87 | 0.75–1.00 | 0.05 | 0.61 |
| *Holdemania* | MR Egger | 14 |  |  | 0.61 | 0.40–0.92 | 0.04 | 1.00 |
| *Holdemania* | Weighted median | 14 |  |  | 0.89 | 0.73–1.09 | 0.28 | 1.00 |
| *Holdemania* | Weighted mode | 14 |  |  | 0.90 | 0.64–1.27 | 0.56 | 0.98 |
| *Holdemania* | ML | 14 |  |  | 0.87 | 0.75–1.01 | 0.06 | 0.51 |
| *Holdemania* | cML-MA-BIC | 14 |  |  | 0.86 | 0.74–1.00 | 0.05 | 0.39 |
| *Howardella* | IVW | 9 | 42.07 | 0.04 | 0.95 | 0.85–1.07 | 0.39 | 0.83 |
| *Howardella* | MR Egger | 9 |  |  | 1.14 | 0.72–1.79 | 0.59 | 1.00 |
| *Howardella* | Weighted median | 9 |  |  | 1.01 | 0.87–1.17 | 0.92 | 1.00 |
| *Howardella* | Weighted mode | 9 |  |  | 1.05 | 0.87–1.27 | 0.62 | 0.98 |
| *Howardella* | ML | 9 |  |  | 0.95 | 0.85–1.06 | 0.39 | 0.72 |
| *Howardella* | cML-MA-BIC | 9 |  |  | 0.95 | 0.85–1.06 | 0.37 | 0.68 |
| *Hungatella* | IVW | 5 | 18.39 | 0.03 | 1.02 | 0.85–1.21 | 0.84 | 0.85 |
| *Hungatella* | MR Egger | 5 |  |  | 0.57 | 0.20–1.58 | 0.36 | 1.00 |
| *Hungatella* | Weighted median | 5 |  |  | 1.00 | 0.81–1.23 | 1.00 | 1.00 |
| *Hungatella* | Weighted mode | 5 |  |  | 0.87 | 0.63–1.21 | 0.46 | 0.98 |
| *Hungatella* | ML | 5 |  |  | 1.02 | 0.86–1.21 | 0.82 | 0.76 |
| *Hungatella* | cML-MA-BIC | 5 |  |  | 1.02 | 0.86–1.21 | 0.82 | 0.77 |
| *Intestinibacter* | IVW | 15 | 52.76 | 0.13 | 0.85 | 0.71–1.02 | 0.08 | 0.61 |
| *Intestinibacter* | MR Egger | 15 |  |  | 0.88 | 0.48–1.62 | 0.69 | 1.00 |
| *Intestinibacter* | Weighted median | 15 |  |  | 0.80 | 0.65–1.00 | 0.05 | 0.97 |
| *Intestinibacter* | Weighted mode | 15 |  |  | 0.77 | 0.55–1.09 | 0.16 | 0.98 |
| *Intestinibacter* | ML | 15 |  |  | 0.85 | 0.72–1.00 | 0.05 | 0.50 |
| *Intestinibacter* | cML-MA-BIC | 15 |  |  | 0.85 | 0.72–1.00 | 0.05 | 0.39 |
| *Intestinimonas* | IVW | 16 | 41.66 | 0.08 | 0.89 | 0.77–1.04 | 0.13 | 0.67 |
| *Intestinimonas* | MR Egger | 16 |  |  | 0.98 | 0.65–1.49 | 0.92 | 1.00 |
| *Intestinimonas* | Weighted median | 16 |  |  | 0.91 | 0.74–1.12 | 0.38 | 1.00 |
| *Intestinimonas* | Weighted mode | 16 |  |  | 0.95 | 0.69–1.32 | 0.79 | 0.98 |
| *Intestinimonas* | ML | 16 |  |  | 0.89 | 0.76–1.04 | 0.14 | 0.58 |
| *Intestinimonas* | cML-MA-BIC | 16 |  |  | 0.89 | 0.76–1.04 | 0.13 | 0.55 |
| *Lachnoclostridium* | IVW | 13 | 23.54 | 0.04 | 0.92 | 0.73–1.16 | 0.49 | 0.85 |
| *Lachnoclostridium* | MR Egger | 13 |  |  | 1.39 | 0.64–3.03 | 0.42 | 1.00 |
| *Lachnoclostridium* | Weighted median | 13 |  |  | 0.88 | 0.66–1.18 | 0.40 | 1.00 |
| *Lachnoclostridium* | Weighted mode | 13 |  |  | 1.37 | 0.69–2.75 | 0.39 | 0.98 |
| *Lachnoclostridium* | ML | 13 |  |  | 0.92 | 0.74–1.14 | 0.44 | 0.73 |
| *Lachnoclostridium* | cML-MA-BIC | 13 |  |  | 0.92 | 0.74–1.13 | 0.42 | 0.68 |
| *Lachnospira* | IVW | 6 | 26.97 | 0.04 | 1.07 | 0.74–1.54 | 0.72 | 0.85 |
| *Lachnospira* | MR Egger | 6 |  |  | 3.06 | 0.35–26.42 | 0.37 | 1.00 |
| *Lachnospira* | Weighted median | 6 |  |  | 0.95 | 0.65–1.39 | 0.79 | 1.00 |
| *Lachnospira* | Weighted mode | 6 |  |  | 0.93 | 0.57–1.51 | 0.78 | 0.98 |
| *Lachnospira* | ML | 6 |  |  | 1.07 | 0.78–1.48 | 0.67 | 0.76 |
| *Lachnospira* | cML-MA-BIC | 6 |  |  | 1.07 | 0.77–1.48 | 0.71 | 0.77 |
| *Lachnospiraceae (FCS020 group)* | IVW | 12 | 40.98 | 0.06 | 0.90 | 0.75–1.08 | 0.27 | 0.81 |
| *Lachnospiraceae (FCS020 group)* | MR Egger | 12 |  |  | 1.16 | 0.72–1.88 | 0.55 | 1.00 |
| *Lachnospiraceae (FCS020 group)* | Weighted median | 12 |  |  | 0.93 | 0.73–1.19 | 0.58 | 1.00 |
| *Lachnospiraceae (FCS020 group)* | Weighted mode | 12 |  |  | 0.95 | 0.68–1.34 | 0.79 | 0.98 |
| *Lachnospiraceae (FCS020 group)* | ML | 12 |  |  | 0.90 | 0.75–1.08 | 0.27 | 0.64 |
| *Lachnospiraceae (FCS020 group)* | cML-MA-BIC | 12 |  |  | 0.90 | 0.75–1.08 | 0.26 | 0.61 |
| *Lachnospiraceae (NC2004 group)* | IVW | 9 | 3.73 | 0.03 | 1.01 | 0.85–1.19 | 0.94 | 0.87 |
| *Lachnospiraceae (NC2004 group)* | MR Egger | 9 |  |  | 0.98 | 0.47–2.05 | 0.96 | 1.00 |
| *Lachnospiraceae (NC2004 group)* | Weighted median | 9 |  |  | 1.00 | 0.80–1.25 | 1.00 | 1.00 |
| *Lachnospiraceae (NC2004 group)* | Weighted mode | 9 |  |  | 1.14 | 0.75–1.74 | 0.55 | 0.98 |
| *Lachnospiraceae (NC2004 group)* | ML | 9 |  |  | 1.01 | 0.86–1.18 | 0.93 | 0.80 |
| *Lachnospiraceae (NC2004 group)* | cML-MA-BIC | 9 |  |  | 1.01 | 0.86–1.18 | 0.91 | 0.78 |
| *Lachnospiraceae (ND3007 group)* | IVW | 3 | 205.43 | 0.26 | 1.35 | 0.88–2.05 | 0.17 | 0.71 |
| *Lachnospiraceae (ND3007 group)* | MR Egger | 3 |  |  | 65.98 | 0.05–80334.82 | 0.45 | 1.00 |
| *Lachnospiraceae (ND3007 group)* | Weighted median | 3 |  |  | 1.26 | 0.74–2.15 | 0.40 | 1.00 |
| *Lachnospiraceae (ND3007 group)* | Weighted mode | 3 |  |  | 1.19 | 0.64–2.25 | 0.64 | 0.98 |
| *Lachnospiraceae (ND3007 group)* | ML | 3 |  |  | 1.36 | 0.88–2.09 | 0.17 | 0.63 |
| *Lachnospiraceae (ND3007 group)* | cML-MA-BIC | 3 |  |  | 1.35 | 0.88–2.09 | 0.17 | 0.58 |
| *Lachnospiraceae (NK4A136 group)* | IVW | 15 | 86.22 | 0.38 | 0.77 | 0.65–0.92 | 3.77E-03 | 0.13 |
| *Lachnospiraceae (NK4A136 group)* | MR Egger | 15 |  |  | 0.67 | 0.47–0.94 | 0.04 | 1.00 |
| *Lachnospiraceae (NK4A136 group)* | Weighted median | 15 |  |  | 0.73 | 0.57–0.92 | 9.20E-03 | 0.55 |
| *Lachnospiraceae (NK4A136 group)* | Weighted mode | 15 |  |  | 0.71 | 0.52–0.95 | 0.04 | 0.98 |
| *Lachnospiraceae (NK4A136 group)* | ML | 15 |  |  | 0.77 | 0.66–0.91 | 2.05E-03 | 0.07 |
| *Lachnospiraceae (NK4A136 group)* | cML-MA-BIC | 15 |  |  | 0.77 | 0.65–0.90 | 1.37E-03 | 0.04 |
| *Lachnospiraceae (UCG001)* | IVW | 12 | 43.34 | 0.07 | 1.13 | 0.92–1.38 | 0.24 | 0.81 |
| *Lachnospiraceae (UCG001)* | MR Egger | 12 |  |  | 0.84 | 0.36–1.97 | 0.70 | 1.00 |
| *Lachnospiraceae (UCG001)* | Weighted median | 12 |  |  | 1.02 | 0.82–1.27 | 0.84 | 1.00 |
| *Lachnospiraceae (UCG001)* | Weighted mode | 12 |  |  | 0.97 | 0.72–1.29 | 0.82 | 0.98 |
| *Lachnospiraceae (UCG001)* | ML | 12 |  |  | 1.14 | 0.97–1.33 | 0.13 | 0.58 |
| *Lachnospiraceae (UCG001)* | cML-MA-BIC | 12 |  |  | 1.09 | 0.91–1.32 | 0.34 | 0.68 |
| *Lachnospiraceae (UCG004)* | IVW | 12 | 14.09 | 0.03 | 1.04 | 0.85–1.27 | 0.70 | 0.85 |
| *Lachnospiraceae (UCG004)* | MR Egger | 12 |  |  | 1.17 | 0.50–2.74 | 0.72 | 1.00 |
| *Lachnospiraceae (UCG004)* | Weighted median | 12 |  |  | 1.00 | 0.77–1.31 | 0.98 | 1.00 |
| *Lachnospiraceae (UCG004)* | Weighted mode | 12 |  |  | 0.92 | 0.59–1.44 | 0.72 | 0.98 |
| *Lachnospiraceae (UCG004)* | ML | 12 |  |  | 1.04 | 0.85–1.28 | 0.69 | 0.76 |
| *Lachnospiraceae (UCG004)* | cML-MA-BIC | 12 |  |  | 1.04 | 0.85–1.28 | 0.70 | 0.77 |
| *Lachnospiraceae (UCG008)* | IVW | 10 | 34.42 | 0.04 | 0.95 | 0.82–1.09 | 0.44 | 0.84 |
| *Lachnospiraceae (UCG008)* | MR Egger | 10 |  |  | 0.88 | 0.43–1.79 | 0.73 | 1.00 |
| *Lachnospiraceae (UCG008)* | Weighted median | 10 |  |  | 0.99 | 0.82–1.19 | 0.89 | 1.00 |
| *Lachnospiraceae (UCG008)* | Weighted mode | 10 |  |  | 1.00 | 0.76–1.31 | 0.98 | 1.00 |
| *Lachnospiraceae (UCG008)* | ML | 10 |  |  | 0.94 | 0.82–1.09 | 0.43 | 0.73 |
| *Lachnospiraceae (UCG008)* | cML-MA-BIC | 10 |  |  | 0.94 | 0.82–1.09 | 0.44 | 0.70 |
| *Lachnospiraceae (UCG010)* | IVW | 10 | 50.96 | 0.07 | 0.88 | 0.72–1.09 | 0.25 | 0.81 |
| *Lachnospiraceae (UCG010)* | MR Egger | 10 |  |  | 0.69 | 0.36–1.31 | 0.29 | 1.00 |
| *Lachnospiraceae (UCG010)* | Weighted median | 10 |  |  | 0.97 | 0.73–1.30 | 0.84 | 1.00 |
| *Lachnospiraceae (UCG010)* | Weighted mode | 10 |  |  | 1.00 | 0.66–1.51 | 0.99 | 1.00 |
| *Lachnospiraceae (UCG010)* | ML | 10 |  |  | 0.88 | 0.71–1.09 | 0.26 | 0.64 |
| *Lachnospiraceae (UCG010)* | cML-MA-BIC | 10 |  |  | 0.88 | 0.71–1.09 | 0.25 | 0.61 |
| *Lactobacillus* | IVW | 8 | 48.99 | 0.04 | 0.94 | 0.81–1.08 | 0.38 | 0.83 |
| *Lactobacillus* | MR Egger | 8 |  |  | 1.26 | 0.87–1.82 | 0.27 | 1.00 |
| *Lactobacillus* | Weighted median | 8 |  |  | 1.01 | 0.83–1.22 | 0.94 | 1.00 |
| *Lactobacillus* | Weighted mode | 8 |  |  | 1.03 | 0.81–1.30 | 0.82 | 0.98 |
| *Lactobacillus* | ML | 8 |  |  | 0.94 | 0.81–1.08 | 0.37 | 0.72 |
| *Lactobacillus* | cML-MA-BIC | 8 |  |  | 0.94 | 0.81–1.08 | 0.38 | 0.68 |
| *Lactococcus* | IVW | 9 | 14.55 | 0.03 | 1.03 | 0.87–1.22 | 0.77 | 0.85 |
| *Lactococcus* | MR Egger | 9 |  |  | 0.97 | 0.43–2.20 | 0.95 | 1.00 |
| *Lactococcus* | Weighted median | 9 |  |  | 0.97 | 0.82–1.14 | 0.67 | 1.00 |
| *Lactococcus* | Weighted mode | 9 |  |  | 0.98 | 0.77–1.24 | 0.84 | 0.98 |
| *Lactococcus* | ML | 9 |  |  | 1.03 | 0.90–1.17 | 0.67 | 0.76 |
| *Lactococcus* | cML-MA-BIC | 9 |  |  | 1.00 | 0.86–1.16 | 0.98 | 0.79 |
| *Marvinbryantia* | IVW | 10 | 9.59 | 0.03 | 0.98 | 0.79–1.21 | 0.83 | 0.85 |
| *Marvinbryantia* | MR Egger | 10 |  |  | 1.02 | 0.42–2.46 | 0.96 | 1.00 |
| *Marvinbryantia* | Weighted median | 10 |  |  | 0.98 | 0.73–1.31 | 0.89 | 1.00 |
| *Marvinbryantia* | Weighted mode | 10 |  |  | 0.93 | 0.54–1.62 | 0.81 | 0.98 |
| *Marvinbryantia* | ML | 10 |  |  | 0.98 | 0.79–1.20 | 0.82 | 0.76 |
| *Marvinbryantia* | cML-MA-BIC | 10 |  |  | 0.98 | 0.79–1.20 | 0.83 | 0.77 |
| *Methanobrevibacter* | IVW | 6 | 137.60 | 0.12 | 0.86 | 0.73–1.01 | 0.06 | 0.61 |
| *Methanobrevibacter* | MR Egger | 6 |  |  | 1.00 | 0.51–1.96 | 1.00 | 1.00 |
| *Methanobrevibacter* | Weighted median | 6 |  |  | 0.86 | 0.71–1.05 | 0.13 | 1.00 |
| *Methanobrevibacter* | Weighted mode | 6 |  |  | 0.88 | 0.69–1.12 | 0.35 | 0.98 |
| *Methanobrevibacter* | ML | 6 |  |  | 0.85 | 0.73–0.99 | 0.04 | 0.50 |
| *Methanobrevibacter* | cML-MA-BIC | 6 |  |  | 0.85 | 0.73–0.99 | 0.04 | 0.39 |
| *Odoribacter* | IVW | 7 | 97.00 | 0.15 | 1.21 | 0.95–1.56 | 0.13 | 0.67 |
| *Odoribacter* | MR Egger | 7 |  |  | 1.28 | 0.58–2.83 | 0.57 | 1.00 |
| *Odoribacter* | Weighted median | 7 |  |  | 1.28 | 0.91–1.80 | 0.15 | 1.00 |
| *Odoribacter* | Weighted mode | 7 |  |  | 1.45 | 0.85–2.49 | 0.22 | 0.98 |
| *Odoribacter* | ML | 7 |  |  | 1.22 | 0.95–1.58 | 0.12 | 0.58 |
| *Odoribacter* | cML-MA-BIC | 7 |  |  | 1.22 | 0.95–1.58 | 0.12 | 0.55 |
| *Olsenella* | IVW | 10 | 66.79 | 0.07 | 1.11 | 0.97–1.27 | 0.13 | 0.67 |
| *Olsenella* | MR Egger | 10 |  |  | 0.96 | 0.62–1.49 | 0.85 | 1.00 |
| *Olsenella* | Weighted median | 10 |  |  | 1.04 | 0.89–1.22 | 0.59 | 1.00 |
| *Olsenella* | Weighted mode | 10 |  |  | 0.96 | 0.77–1.20 | 0.76 | 0.98 |
| *Olsenella* | ML | 10 |  |  | 1.12 | 1.00–1.25 | 0.05 | 0.51 |
| *Olsenella* | cML-MA-BIC | 10 |  |  | 1.11 | 0.99–1.25 | 0.07 | 0.41 |
| *Oscillibacter* | IVW | 13 | 6.98 | 0.03 | 1.02 | 0.87–1.18 | 0.84 | 0.85 |
| *Oscillibacter* | MR Egger | 13 |  |  | 0.93 | 0.51–1.70 | 0.82 | 1.00 |
| *Oscillibacter* | Weighted median | 13 |  |  | 1.06 | 0.87–1.31 | 0.56 | 1.00 |
| *Oscillibacter* | Weighted mode | 13 |  |  | 1.02 | 0.70–1.48 | 0.91 | 0.98 |
| *Oscillibacter* | ML | 13 |  |  | 1.02 | 0.88–1.18 | 0.82 | 0.76 |
| *Oscillibacter* | cML-MA-BIC | 13 |  |  | 1.02 | 0.88–1.18 | 0.82 | 0.77 |
| *Oscillospira* | IVW | 8 | 21.96 | 0.03 | 1.04 | 0.85–1.27 | 0.69 | 0.85 |
| *Oscillospira* | MR Egger | 8 |  |  | 1.11 | 0.48–2.56 | 0.81 | 1.00 |
| *Oscillospira* | Weighted median | 8 |  |  | 1.10 | 0.85–1.43 | 0.46 | 1.00 |
| *Oscillospira* | Weighted mode | 8 |  |  | 1.16 | 0.79–1.71 | 0.47 | 0.98 |
| *Oscillospira* | ML | 8 |  |  | 1.04 | 0.85–1.27 | 0.69 | 0.76 |
| *Oscillospira* | cML-MA-BIC | 8 |  |  | 1.04 | 0.85–1.27 | 0.68 | 0.77 |
| *Oxalobacter* | IVW | 11 | 24.69 | 0.04 | 1.05 | 0.90–1.24 | 0.54 | 0.85 |
| *Oxalobacter* | MR Egger | 11 |  |  | 0.57 | 0.29–1.14 | 0.15 | 1.00 |
| *Oxalobacter* | Weighted median | 11 |  |  | 1.01 | 0.86–1.18 | 0.88 | 1.00 |
| *Oxalobacter* | Weighted mode | 11 |  |  | 0.98 | 0.75–1.29 | 0.90 | 0.98 |
| *Oxalobacter* | ML | 11 |  |  | 1.06 | 0.94–1.19 | 0.36 | 0.72 |
| *Oxalobacter* | cML-MA-BIC | 11 |  |  | 1.01 | 0.88–1.16 | 0.88 | 0.77 |
| *Parabacteroides* | IVW | 5 | 56.24 | 0.05 | 0.90 | 0.66–1.23 | 0.53 | 0.85 |
| *Parabacteroides* | MR Egger | 5 |  |  | 1.06 | 0.15–7.30 | 0.96 | 1.00 |
| *Parabacteroides* | Weighted median | 5 |  |  | 0.84 | 0.57–1.26 | 0.40 | 1.00 |
| *Parabacteroides* | Weighted mode | 5 |  |  | 0.79 | 0.48–1.31 | 0.42 | 0.98 |
| *Parabacteroides* | ML | 5 |  |  | 0.90 | 0.66–1.23 | 0.52 | 0.75 |
| *Parabacteroides* | cML-MA-BIC | 5 |  |  | 0.90 | 0.66–1.23 | 0.52 | 0.72 |
| *Paraprevotella* | IVW | 13 | 11.61 | 0.03 | 1.03 | 0.88–1.19 | 0.73 | 0.85 |
| *Paraprevotella* | MR Egger | 13 |  |  | 0.80 | 0.45–1.41 | 0.45 | 1.00 |
| *Paraprevotella* | Weighted median | 13 |  |  | 1.00 | 0.83–1.20 | 0.97 | 1.00 |
| *Paraprevotella* | Weighted mode | 13 |  |  | 0.97 | 0.72–1.30 | 0.84 | 0.98 |
| *Paraprevotella* | ML | 13 |  |  | 1.03 | 0.91–1.17 | 0.66 | 0.76 |
| *Paraprevotella* | cML-MA-BIC | 13 |  |  | 1.03 | 0.91–1.17 | 0.64 | 0.77 |
| *Parasutterella* | IVW | 14 | 12.66 | 0.03 | 1.03 | 0.88–1.21 | 0.69 | 0.85 |
| *Parasutterella* | MR Egger | 14 |  |  | 0.89 | 0.56–1.39 | 0.60 | 1.00 |
| *Parasutterella* | Weighted median | 14 |  |  | 1.04 | 0.83–1.29 | 0.75 | 1.00 |
| *Parasutterella* | Weighted mode | 14 |  |  | 1.05 | 0.77–1.41 | 0.78 | 0.98 |
| *Parasutterella* | ML | 14 |  |  | 1.03 | 0.89–1.20 | 0.66 | 0.76 |
| *Parasutterella* | cML-MA-BIC | 14 |  |  | 1.03 | 0.89–1.20 | 0.67 | 0.77 |
| *Peptococcus* | IVW | 12 | 2.95 | 0.03 | 1.00 | 0.89–1.13 | 0.94 | 0.87 |
| *Peptococcus* | MR Egger | 12 |  |  | 1.00 | 0.64–1.57 | 0.99 | 1.00 |
| *Peptococcus* | Weighted median | 12 |  |  | 1.06 | 0.92–1.23 | 0.42 | 1.00 |
| *Peptococcus* | Weighted mode | 12 |  |  | 1.07 | 0.86–1.35 | 0.55 | 0.98 |
| *Peptococcus* | ML | 12 |  |  | 1.00 | 0.89–1.13 | 0.94 | 0.80 |
| *Peptococcus* | cML-MA-BIC | 12 |  |  | 1.01 | 0.89–1.13 | 0.93 | 0.78 |
| *Phascolarctobacterium* | IVW | 8 | 15.43 | 0.03 | 1.03 | 0.84–1.27 | 0.78 | 0.85 |
| *Phascolarctobacterium* | MR Egger | 8 |  |  | 2.98 | 1.12–7.88 | 0.07 | 1.00 |
| *Phascolarctobacterium* | Weighted median | 8 |  |  | 1.05 | 0.80–1.39 | 0.72 | 1.00 |
| *Phascolarctobacterium* | Weighted mode | 8 |  |  | 1.07 | 0.74–1.55 | 0.73 | 0.98 |
| *Phascolarctobacterium* | ML | 8 |  |  | 1.03 | 0.83–1.28 | 0.78 | 0.76 |
| *Phascolarctobacterium* | cML-MA-BIC | 8 |  |  | 1.03 | 0.83–1.28 | 0.77 | 0.77 |
| *Prevotella 7* | IVW | 11 | 43.28 | 0.04 | 0.94 | 0.85–1.05 | 0.28 | 0.81 |
| *Prevotella 7* | MR Egger | 11 |  |  | 0.99 | 0.54–1.81 | 0.97 | 1.00 |
| *Prevotella 7* | Weighted median | 11 |  |  | 0.91 | 0.80–1.04 | 0.19 | 1.00 |
| *Prevotella 7* | Weighted mode | 11 |  |  | 0.91 | 0.74–1.12 | 0.40 | 0.98 |
| *Prevotella 7* | ML | 11 |  |  | 0.94 | 0.85–1.05 | 0.28 | 0.64 |
| *Prevotella 7* | cML-MA-BIC | 11 |  |  | 0.94 | 0.85–1.05 | 0.28 | 0.62 |
| *Prevotella 9* | IVW | 15 | 53.81 | 0.09 | 1.13 | 0.99–1.30 | 0.07 | 0.61 |
| *Prevotella 9* | MR Egger | 15 |  |  | 0.94 | 0.63–1.40 | 0.77 | 1.00 |
| *Prevotella 9* | Weighted median | 15 |  |  | 1.13 | 0.93–1.36 | 0.22 | 1.00 |
| *Prevotella 9* | Weighted mode | 15 |  |  | 0.96 | 0.70–1.31 | 0.79 | 0.98 |
| *Prevotella 9* | ML | 15 |  |  | 1.14 | 0.99–1.31 | 0.07 | 0.51 |
| *Prevotella 9* | cML-MA-BIC | 15 |  |  | 1.14 | 0.99–1.31 | 0.07 | 0.41 |
| *Rikenellaceae (RC9 gut group)* | IVW | 11 | 45.76 | 0.04 | 0.94 | 0.85–1.04 | 0.26 | 0.81 |
| *Rikenellaceae (RC9 gut group)* | MR Egger | 11 |  |  | 1.07 | 0.57–2.01 | 0.83 | 1.00 |
| *Rikenellaceae (RC9 gut group)* | Weighted median | 11 |  |  | 0.94 | 0.82–1.07 | 0.35 | 1.00 |
| *Rikenellaceae (RC9 gut group)* | Weighted mode | 11 |  |  | 0.93 | 0.76–1.14 | 0.48 | 0.98 |
| *Rikenellaceae (RC9 gut group)* | ML | 11 |  |  | 0.94 | 0.85–1.04 | 0.26 | 0.64 |
| *Rikenellaceae (RC9 gut group)* | cML-MA-BIC | 11 |  |  | 0.94 | 0.85–1.04 | 0.25 | 0.61 |
| *Romboutsia* | IVW | 13 | 4.83 | 0.03 | 0.99 | 0.81–1.20 | 0.89 | 0.87 |
| *Romboutsia* | MR Egger | 13 |  |  | 1.18 | 0.65–2.14 | 0.59 | 1.00 |
| *Romboutsia* | Weighted median | 13 |  |  | 1.10 | 0.84–1.44 | 0.49 | 1.00 |
| *Romboutsia* | Weighted mode | 13 |  |  | 1.30 | 0.74–2.26 | 0.38 | 0.98 |
| *Romboutsia* | ML | 13 |  |  | 0.98 | 0.82–1.19 | 0.87 | 0.78 |
| *Romboutsia* | cML-MA-BIC | 13 |  |  | 0.99 | 0.82–1.19 | 0.88 | 0.77 |
| *Roseburia* | IVW | 14 | 15.10 | 0.03 | 1.05 | 0.86–1.28 | 0.63 | 0.85 |
| *Roseburia* | MR Egger | 14 |  |  | 1.23 | 0.66–2.29 | 0.52 | 1.00 |
| *Roseburia* | Weighted median | 14 |  |  | 0.97 | 0.74–1.26 | 0.81 | 1.00 |
| *Roseburia* | Weighted mode | 14 |  |  | 0.89 | 0.55–1.45 | 0.64 | 0.98 |
| *Roseburia* | ML | 14 |  |  | 1.05 | 0.86–1.29 | 0.62 | 0.76 |
| *Roseburia* | cML-MA-BIC | 14 |  |  | 1.05 | 0.86–1.29 | 0.63 | 0.76 |
| *Ruminiclostridium 5* | IVW | 11 | 12.03 | 0.03 | 1.05 | 0.77–1.42 | 0.77 | 0.85 |
| *Ruminiclostridium 5* | MR Egger | 11 |  |  | 0.74 | 0.20–2.79 | 0.67 | 1.00 |
| *Ruminiclostridium 5* | Weighted median | 11 |  |  | 1.17 | 0.85–1.60 | 0.35 | 1.00 |
| *Ruminiclostridium 5* | Weighted mode | 11 |  |  | 1.29 | 0.76–2.18 | 0.36 | 0.98 |
| *Ruminiclostridium 5* | ML | 11 |  |  | 1.05 | 0.83–1.32 | 0.67 | 0.76 |
| *Ruminiclostridium 5* | cML-MA-BIC | 11 |  |  | 1.05 | 0.84–1.33 | 0.65 | 0.77 |
| *Ruminiclostridium 6* | IVW | 14 | 11.68 | 0.03 | 0.97 | 0.81–1.16 | 0.71 | 0.85 |
| *Ruminiclostridium 6* | MR Egger | 14 |  |  | 0.95 | 0.62–1.46 | 0.81 | 1.00 |
| *Ruminiclostridium 6* | Weighted median | 14 |  |  | 0.93 | 0.73–1.19 | 0.59 | 1.00 |
| *Ruminiclostridium 6* | Weighted mode | 14 |  |  | 0.90 | 0.62–1.31 | 0.59 | 0.98 |
| *Ruminiclostridium 6* | ML | 14 |  |  | 0.97 | 0.81–1.16 | 0.71 | 0.76 |
| *Ruminiclostridium 6* | cML-MA-BIC | 14 |  |  | 0.97 | 0.81–1.16 | 0.70 | 0.77 |
| *Ruminiclostridium 9* | IVW | 7 | 19.86 | 0.03 | 0.94 | 0.64–1.38 | 0.75 | 0.85 |
| *Ruminiclostridium 9* | MR Egger | 7 |  |  | 0.52 | 0.08–3.23 | 0.51 | 1.00 |
| *Ruminiclostridium 9* | Weighted median | 7 |  |  | 1.04 | 0.67–1.61 | 0.87 | 1.00 |
| *Ruminiclostridium 9* | Weighted mode | 7 |  |  | 1.28 | 0.59–2.74 | 0.56 | 0.98 |
| *Ruminiclostridium 9* | ML | 7 |  |  | 0.94 | 0.70–1.25 | 0.66 | 0.76 |
| *Ruminiclostridium 9* | cML-MA-BIC | 7 |  |  | 0.93 | 0.70–1.25 | 0.65 | 0.77 |
| *Ruminococcaceae (NK4A214 group)* | IVW | 13 | 17.98 | 0.04 | 0.94 | 0.74–1.19 | 0.60 | 0.85 |
| *Ruminococcaceae (NK4A214 group)* | MR Egger | 13 |  |  | 0.97 | 0.44–2.15 | 0.94 | 1.00 |
| *Ruminococcaceae (NK4A214 group)* | Weighted median | 13 |  |  | 0.89 | 0.67–1.20 | 0.46 | 1.00 |
| *Ruminococcaceae (NK4A214 group)* | Weighted mode | 13 |  |  | 0.78 | 0.42–1.43 | 0.43 | 0.98 |
| *Ruminococcaceae (NK4A214 group)* | ML | 13 |  |  | 0.93 | 0.77–1.14 | 0.50 | 0.73 |
| *Ruminococcaceae (NK4A214 group)* | cML-MA-BIC | 13 |  |  | 0.94 | 0.77–1.16 | 0.58 | 0.76 |
| *Ruminococcaceae (UCG002)* | IVW | 19 | 39.13 | 0.10 | 0.87 | 0.74–1.02 | 0.09 | 0.67 |
| *Ruminococcaceae (UCG002)* | MR Egger | 19 |  |  | 1.02 | 0.67–1.55 | 0.93 | 1.00 |
| *Ruminococcaceae (UCG002)* | Weighted median | 19 |  |  | 0.81 | 0.65–1.03 | 0.08 | 0.97 |
| *Ruminococcaceae (UCG002)* | Weighted mode | 19 |  |  | 0.68 | 0.41–1.15 | 0.17 | 0.98 |
| *Ruminococcaceae (UCG002)* | ML | 19 |  |  | 0.87 | 0.74–1.02 | 0.08 | 0.53 |
| *Ruminococcaceae (UCG002)* | cML-MA-BIC | 19 |  |  | 0.86 | 0.73–1.01 | 0.06 | 0.41 |
| *Ruminococcaceae (UCG003)* | IVW | 12 | 59.19 | 0.11 | 0.86 | 0.72–1.03 | 0.11 | 0.67 |
| *Ruminococcaceae (UCG003)* | MR Egger | 12 |  |  | 0.70 | 0.38–1.27 | 0.27 | 1.00 |
| *Ruminococcaceae (UCG003)* | Weighted median | 12 |  |  | 0.92 | 0.72–1.17 | 0.48 | 1.00 |
| *Ruminococcaceae (UCG003)* | Weighted mode | 12 |  |  | 0.92 | 0.65–1.31 | 0.65 | 0.98 |
| *Ruminococcaceae (UCG003)* | ML | 12 |  |  | 0.86 | 0.72–1.04 | 0.12 | 0.58 |
| *Ruminococcaceae (UCG003)* | cML-MA-BIC | 12 |  |  | 0.86 | 0.71–1.04 | 0.11 | 0.55 |
| *Ruminococcaceae (UCG004)* | IVW | 11 | 45.13 | 0.06 | 0.91 | 0.77–1.07 | 0.26 | 0.81 |
| *Ruminococcaceae (UCG004)* | MR Egger | 11 |  |  | 1.19 | 0.48–2.99 | 0.71 | 1.00 |
| *Ruminococcaceae (UCG004)* | Weighted median | 11 |  |  | 0.86 | 0.69–1.07 | 0.19 | 1.00 |
| *Ruminococcaceae (UCG004)* | Weighted mode | 11 |  |  | 0.80 | 0.56–1.14 | 0.25 | 0.98 |
| *Ruminococcaceae (UCG004)* | ML | 11 |  |  | 0.91 | 0.77–1.07 | 0.26 | 0.64 |
| *Ruminococcaceae (UCG004)* | cML-MA-BIC | 11 |  |  | 0.91 | 0.76–1.07 | 0.25 | 0.61 |
| *Ruminococcaceae (UCG005)* | IVW | 14 | 12.75 | 0.03 | 1.04 | 0.85–1.28 | 0.69 | 0.85 |
| *Ruminococcaceae (UCG005)* | MR Egger | 14 |  |  | 0.97 | 0.55–1.70 | 0.91 | 1.00 |
| *Ruminococcaceae (UCG005)* | Weighted median | 14 |  |  | 0.98 | 0.75–1.27 | 0.86 | 1.00 |
| *Ruminococcaceae (UCG005)* | Weighted mode | 14 |  |  | 0.88 | 0.61–1.25 | 0.48 | 0.98 |
| *Ruminococcaceae (UCG005)* | ML | 14 |  |  | 1.05 | 0.88–1.24 | 0.62 | 0.76 |
| *Ruminococcaceae (UCG005)* | cML-MA-BIC | 14 |  |  | 1.05 | 0.88–1.25 | 0.62 | 0.76 |
| *Ruminococcaceae (UCG009)* | IVW | 12 | 36.57 | 0.05 | 1.07 | 0.93–1.24 | 0.32 | 0.83 |
| *Ruminococcaceae (UCG009)* | MR Egger | 12 |  |  | 1.83 | 1.06–3.17 | 0.06 | 1.00 |
| *Ruminococcaceae (UCG009)* | Weighted median | 12 |  |  | 1.18 | 0.97–1.44 | 0.10 | 1.00 |
| *Ruminococcaceae (UCG009)* | Weighted mode | 12 |  |  | 1.24 | 0.90–1.70 | 0.21 | 0.98 |
| *Ruminococcaceae (UCG009)* | ML | 12 |  |  | 1.08 | 0.94–1.24 | 0.30 | 0.66 |
| *Ruminococcaceae (UCG009)* | cML-MA-BIC | 12 |  |  | 1.08 | 0.94–1.24 | 0.30 | 0.62 |
| *Ruminococcaceae (UCG010)* | IVW | 6 | 82.53 | 0.07 | 0.87 | 0.69–1.11 | 0.27 | 0.81 |
| *Ruminococcaceae (UCG010)* | MR Egger | 6 |  |  | 1.21 | 0.63–2.35 | 0.60 | 1.00 |
| *Ruminococcaceae (UCG010)* | Weighted median | 6 |  |  | 0.87 | 0.64–1.17 | 0.35 | 1.00 |
| *Ruminococcaceae (UCG010)* | Weighted mode | 6 |  |  | 0.95 | 0.66–1.38 | 0.81 | 0.98 |
| *Ruminococcaceae (UCG010)* | ML | 6 |  |  | 0.87 | 0.68–1.11 | 0.26 | 0.64 |
| *Ruminococcaceae (UCG010)* | cML-MA-BIC | 6 |  |  | 0.87 | 0.68–1.11 | 0.27 | 0.61 |
| *Ruminococcaceae (UCG011)* | IVW | 8 | 12.77 | 0.03 | 1.02 | 0.89–1.16 | 0.82 | 0.85 |
| *Ruminococcaceae (UCG011)* | MR Egger | 8 |  |  | 0.57 | 0.32–1.01 | 0.10 | 1.00 |
| *Ruminococcaceae (UCG011)* | Weighted median | 8 |  |  | 0.97 | 0.82–1.14 | 0.69 | 1.00 |
| *Ruminococcaceae (UCG011)* | Weighted mode | 8 |  |  | 0.93 | 0.72–1.18 | 0.55 | 0.98 |
| *Ruminococcaceae (UCG011)* | ML | 8 |  |  | 1.02 | 0.90–1.14 | 0.79 | 0.76 |
| *Ruminococcaceae (UCG011)* | cML-MA-BIC | 8 |  |  | 1.01 | 0.90–1.14 | 0.82 | 0.77 |
| *Ruminococcaceae (UCG013)* | IVW | 11 | 28.93 | 0.04 | 0.93 | 0.75–1.14 | 0.47 | 0.85 |
| *Ruminococcaceae (UCG013)* | MR Egger | 11 |  |  | 1.57 | 0.86–2.85 | 0.17 | 1.00 |
| *Ruminococcaceae (UCG013)* | Weighted median | 11 |  |  | 0.95 | 0.72–1.25 | 0.71 | 1.00 |
| *Ruminococcaceae (UCG013)* | Weighted mode | 11 |  |  | 1.14 | 0.70–1.84 | 0.62 | 0.98 |
| *Ruminococcaceae (UCG013)* | ML | 11 |  |  | 0.93 | 0.75–1.15 | 0.48 | 0.73 |
| *Ruminococcaceae (UCG013)* | cML-MA-BIC | 11 |  |  | 0.92 | 0.75–1.14 | 0.46 | 0.70 |
| *Ruminococcaceae (UCG014)* | IVW | 11 | 37.34 | 0.05 | 1.09 | 0.91–1.31 | 0.36 | 0.83 |
| *Ruminococcaceae (UCG014)* | MR Egger | 11 |  |  | 1.26 | 0.82–1.93 | 0.32 | 1.00 |
| *Ruminococcaceae (UCG014)* | Weighted median | 11 |  |  | 1.16 | 0.90–1.50 | 0.26 | 1.00 |
| *Ruminococcaceae (UCG014)* | Weighted mode | 11 |  |  | 1.19 | 0.89–1.61 | 0.27 | 0.98 |
| *Ruminococcaceae (UCG014)* | ML | 11 |  |  | 1.09 | 0.91–1.31 | 0.35 | 0.72 |
| *Ruminococcaceae (UCG014)* | cML-MA-BIC | 11 |  |  | 1.09 | 0.91–1.31 | 0.35 | 0.68 |
| *Ruminococcus (gauvreauii group)* | IVW | 11 | 25.97 | 0.04 | 0.94 | 0.78–1.14 | 0.52 | 0.85 |
| *Ruminococcus (gauvreauii group)* | MR Egger | 11 |  |  | 0.94 | 0.43–2.07 | 0.88 | 1.00 |
| *Ruminococcus (gauvreauii group)* | Weighted median | 11 |  |  | 0.92 | 0.72–1.18 | 0.52 | 1.00 |
| *Ruminococcus (gauvreauii group)* | Weighted mode | 11 |  |  | 0.90 | 0.63–1.31 | 0.60 | 0.98 |
| *Ruminococcus (gauvreauii group)* | ML | 11 |  |  | 0.94 | 0.77–1.14 | 0.52 | 0.75 |
| *Ruminococcus (gauvreauii group)* | cML-MA-BIC | 11 |  |  | 0.94 | 0.77–1.14 | 0.51 | 0.71 |
| *Ruminococcus (gnavus group)* | IVW | 11 | 11.21 | 0.03 | 1.02 | 0.89–1.17 | 0.78 | 0.85 |
| *Ruminococcus (gnavus group)* | MR Egger | 11 |  |  | 1.12 | 0.59–2.15 | 0.73 | 1.00 |
| *Ruminococcus (gnavus group)* | Weighted median | 11 |  |  | 1.05 | 0.88–1.25 | 0.57 | 1.00 |
| *Ruminococcus (gnavus group)* | Weighted mode | 11 |  |  | 1.09 | 0.84–1.42 | 0.54 | 0.98 |
| *Ruminococcus (gnavus group)* | ML | 11 |  |  | 1.02 | 0.89–1.17 | 0.77 | 0.76 |
| *Ruminococcus (gnavus group)* | cML-MA-BIC | 11 |  |  | 1.02 | 0.89–1.17 | 0.78 | 0.77 |
| *Ruminococcus (torques group)* | IVW | 7 | 53.70 | 0.08 | 1.18 | 0.81–1.72 | 0.40 | 0.83 |
| *Ruminococcus (torques group)* | MR Egger | 7 |  |  | 3.25 | 1.31–8.06 | 0.05 | 1.00 |
| *Ruminococcus (torques group)* | Weighted median | 7 |  |  | 1.16 | 0.77–1.74 | 0.48 | 1.00 |
| *Ruminococcus (torques group)* | Weighted mode | 7 |  |  | 1.70 | 0.78–3.72 | 0.23 | 0.98 |
| *Ruminococcus (torques group)* | ML | 7 |  |  | 1.19 | 0.87–1.62 | 0.27 | 0.64 |
| *Ruminococcus (torques group)* | cML-MA-BIC | 7 |  |  | 1.19 | 0.87–1.63 | 0.27 | 0.61 |
| *Ruminococcus 1* | IVW | 10 | 79.81 | 0.16 | 0.83 | 0.67–1.02 | 0.07 | 0.61 |
| *Ruminococcus 1* | MR Egger | 10 |  |  | 0.89 | 0.51–1.55 | 0.70 | 1.00 |
| *Ruminococcus 1* | Weighted median | 10 |  |  | 0.83 | 0.64–1.08 | 0.17 | 1.00 |
| *Ruminococcus 1* | Weighted mode | 10 |  |  | 0.83 | 0.56–1.23 | 0.38 | 0.98 |
| *Ruminococcus 1* | ML | 10 |  |  | 0.82 | 0.67–1.02 | 0.07 | 0.51 |
| *Ruminococcus 1* | cML-MA-BIC | 10 |  |  | 0.82 | 0.67–1.02 | 0.07 | 0.41 |
| *Ruminococcus 2* | IVW | 15 | 14.90 | 0.03 | 1.04 | 0.89–1.22 | 0.61 | 0.85 |
| *Ruminococcus 2* | MR Egger | 15 |  |  | 1.00 | 0.69–1.47 | 0.98 | 1.00 |
| *Ruminococcus 2* | Weighted median | 15 |  |  | 0.99 | 0.79–1.24 | 0.96 | 1.00 |
| *Ruminococcus 2* | Weighted mode | 15 |  |  | 0.98 | 0.76–1.27 | 0.88 | 0.98 |
| *Ruminococcus 2* | ML | 15 |  |  | 1.04 | 0.89–1.22 | 0.60 | 0.76 |
| *Ruminococcus 2* | cML-MA-BIC | 15 |  |  | 1.04 | 0.89–1.22 | 0.61 | 0.76 |
| *Sellimonas* | IVW | 9 | 39.79 | 0.04 | 0.96 | 0.87–1.06 | 0.42 | 0.83 |
| *Sellimonas* | MR Egger | 9 |  |  | 0.91 | 0.50–1.66 | 0.77 | 1.00 |
| *Sellimonas* | Weighted median | 9 |  |  | 0.96 | 0.84–1.10 | 0.58 | 1.00 |
| *Sellimonas* | Weighted mode | 9 |  |  | 0.98 | 0.80–1.20 | 0.85 | 0.98 |
| *Sellimonas* | ML | 9 |  |  | 0.96 | 0.86–1.06 | 0.42 | 0.72 |
| *Sellimonas* | cML-MA-BIC | 9 |  |  | 0.96 | 0.86–1.06 | 0.41 | 0.68 |
| *Senegalimassilia* | IVW | 5 | 122.64 | 0.11 | 0.85 | 0.68–1.07 | 0.17 | 0.71 |
| *Senegalimassilia* | MR Egger | 5 |  |  | 0.85 | 0.35–2.03 | 0.74 | 1.00 |
| *Senegalimassilia* | Weighted median | 5 |  |  | 0.90 | 0.68–1.19 | 0.44 | 1.00 |
| *Senegalimassilia* | Weighted mode | 5 |  |  | 0.94 | 0.62–1.41 | 0.78 | 0.98 |
| *Senegalimassilia* | ML | 5 |  |  | 0.85 | 0.67–1.07 | 0.17 | 0.63 |
| *Senegalimassilia* | cML-MA-BIC | 5 |  |  | 0.85 | 0.67–1.07 | 0.17 | 0.58 |
| *Slackia* | IVW | 6 | 32.30 | 0.03 | 1.04 | 0.86–1.26 | 0.66 | 0.85 |
| *Slackia* | MR Egger | 6 |  |  | 1.05 | 0.31–3.60 | 0.94 | 1.00 |
| *Slackia* | Weighted median | 6 |  |  | 1.03 | 0.81–1.29 | 0.83 | 1.00 |
| *Slackia* | Weighted mode | 6 |  |  | 1.01 | 0.75–1.37 | 0.95 | 1.00 |
| *Slackia* | ML | 6 |  |  | 1.04 | 0.86–1.26 | 0.66 | 0.76 |
| *Slackia* | cML-MA-BIC | 6 |  |  | 1.04 | 0.86–1.26 | 0.66 | 0.77 |
| *Streptococcus* | IVW | 12 | 56.98 | 0.12 | 1.18 | 0.96–1.46 | 0.12 | 0.67 |
| *Streptococcus* | MR Egger | 12 |  |  | 0.98 | 0.45–2.15 | 0.97 | 1.00 |
| *Streptococcus* | Weighted median | 12 |  |  | 1.09 | 0.82–1.44 | 0.56 | 1.00 |
| *Streptococcus* | Weighted mode | 12 |  |  | 1.04 | 0.69–1.56 | 0.87 | 0.98 |
| *Streptococcus* | ML | 12 |  |  | 1.19 | 0.96–1.48 | 0.11 | 0.58 |
| *Streptococcus* | cML-MA-BIC | 12 |  |  | 1.19 | 0.96–1.47 | 0.12 | 0.55 |
| *Subdoligranulum* | IVW | 11 | 35.97 | 0.06 | 1.12 | 0.88–1.43 | 0.37 | 0.83 |
| *Subdoligranulum* | MR Egger | 11 |  |  | 0.91 | 0.48–1.73 | 0.77 | 1.00 |
| *Subdoligranulum* | Weighted median | 11 |  |  | 1.08 | 0.82–1.43 | 0.56 | 1.00 |
| *Subdoligranulum* | Weighted mode | 11 |  |  | 1.03 | 0.71–1.51 | 0.87 | 0.98 |
| *Subdoligranulum* | ML | 11 |  |  | 1.12 | 0.91–1.39 | 0.28 | 0.64 |
| *Subdoligranulum* | cML-MA-BIC | 11 |  |  | 1.12 | 0.91–1.39 | 0.28 | 0.62 |
| *Sutterella* | IVW | 12 | 17.83 | 0.03 | 1.05 | 0.87–1.27 | 0.63 | 0.85 |
| *Sutterella* | MR Egger | 12 |  |  | 0.80 | 0.34–1.86 | 0.61 | 1.00 |
| *Sutterella* | Weighted median | 12 |  |  | 1.01 | 0.79–1.30 | 0.93 | 1.00 |
| *Sutterella* | Weighted mode | 12 |  |  | 1.04 | 0.70–1.54 | 0.86 | 0.98 |
| *Sutterella* | ML | 12 |  |  | 1.05 | 0.87–1.27 | 0.61 | 0.76 |
| *Sutterella* | cML-MA-BIC | 12 |  |  | 1.05 | 0.87–1.27 | 0.60 | 0.76 |
| *Terrisporobacter* | IVW | 5 | 3.65 | 0.03 | 1.00 | 0.81–1.25 | 0.97 | 0.88 |
| *Terrisporobacter* | MR Egger | 5 |  |  | 1.02 | 0.53–1.95 | 0.96 | 1.00 |
| *Terrisporobacter* | Weighted median | 5 |  |  | 1.02 | 0.79–1.32 | 0.88 | 1.00 |
| *Terrisporobacter* | Weighted mode | 5 |  |  | 1.04 | 0.75–1.45 | 0.81 | 0.98 |
| *Terrisporobacter* | ML | 5 |  |  | 1.00 | 0.81–1.25 | 0.97 | 0.80 |
| *Terrisporobacter* | cML-MA-BIC | 5 |  |  | 1.00 | 0.81–1.25 | 0.97 | 0.79 |
| *Turicibacter* | IVW | 9 | 42.12 | 0.05 | 1.08 | 0.91–1.29 | 0.39 | 0.83 |
| *Turicibacter* | MR Egger | 9 |  |  | 0.81 | 0.40–1.65 | 0.58 | 1.00 |
| *Turicibacter* | Weighted median | 9 |  |  | 1.07 | 0.85–1.35 | 0.57 | 1.00 |
| *Turicibacter* | Weighted mode | 9 |  |  | 0.89 | 0.62–1.28 | 0.54 | 0.98 |
| *Turicibacter* | ML | 9 |  |  | 1.08 | 0.90–1.30 | 0.39 | 0.72 |
| *Turicibacter* | cML-MA-BIC | 9 |  |  | 1.08 | 0.90–1.30 | 0.39 | 0.68 |
| *Tyzzerella 3* | IVW | 13 | 85.50 | 0.17 | 0.85 | 0.74–0.97 | 0.01 | 0.27 |
| *Tyzzerella 3* | MR Egger | 13 |  |  | 0.66 | 0.36–1.21 | 0.21 | 1.00 |
| *Tyzzerella 3* | Weighted median | 13 |  |  | 0.77 | 0.66–0.89 | 6.00E-04 | 0.07 |
| *Tyzzerella 3* | Weighted mode | 13 |  |  | 0.75 | 0.62–0.92 | 0.02 | 0.98 |
| *Tyzzerella 3* | ML | 13 |  |  | 0.85 | 0.76–0.94 | 1.68E-03 | 0.07 |
| *Tyzzerella 3* | cML-MA-BIC | 13 |  |  | 0.84 | 0.76–0.93 | 1.08E-03 | 0.04 |
| *Veillonella* | IVW | 6 | 66.18 | 0.06 | 0.90 | 0.72–1.13 | 0.37 | 0.83 |
| *Veillonella* | MR Egger | 6 |  |  | 0.76 | 0.13–4.54 | 0.78 | 1.00 |
| *Veillonella* | Weighted median | 6 |  |  | 0.96 | 0.73–1.26 | 0.78 | 1.00 |
| *Veillonella* | Weighted mode | 6 |  |  | 0.98 | 0.66–1.46 | 0.92 | 0.99 |
| *Veillonella* | ML | 6 |  |  | 0.90 | 0.72–1.13 | 0.37 | 0.72 |
| *Veillonella* | cML-MA-BIC | 6 |  |  | 0.90 | 0.72–1.13 | 0.37 | 0.68 |
| *Victivallis* | IVW | 10 | 46.08 | 0.04 | 1.06 | 0.95–1.18 | 0.30 | 0.83 |
| *Victivallis* | MR Egger | 10 |  |  | 1.21 | 0.52–2.79 | 0.67 | 1.00 |
| *Victivallis* | Weighted median | 10 |  |  | 1.06 | 0.92–1.23 | 0.42 | 1.00 |
| *Victivallis* | Weighted mode | 10 |  |  | 1.12 | 0.87–1.44 | 0.42 | 0.98 |
| *Victivallis* | ML | 10 |  |  | 1.06 | 0.95–1.19 | 0.28 | 0.64 |
| *Victivallis* | cML-MA-BIC | 10 |  |  | 1.06 | 0.95–1.18 | 0.29 | 0.62 |
| MR, Mendelian randomization; PE, preeclampsia-eclampsia; SNP, single nucleotide polymorphism; OR, odds ratio; CI, confidence interval; IVW, inverse variance weighted; ML, maximum likelihood. | | | | | | | | |

| **Table S3 The heterogeneity of gut microbiota instrumental variables.** | | | |
| --- | --- | --- | --- |
| **Bacterial taxa (exposure)** | **Cochran's Q** | **df** | **P-value** |
| *Actinomyces* | 16.55 | 6 | 0.01 |
| *Adlercreutzia* | 7.84 | 7 | 0.35 |
| *Akkermansia* | 7.05 | 10 | 0.72 |
| *Alistipes* | 17.66 | 11 | 0.09 |
| *Allisonella* | 3.85 | 7 | 0.80 |
| *Alloprevotella* | 12.54 | 4 | 0.01 |
| *Anaerofilum* | 14.36 | 10 | 0.16 |
| *Anaerostipes* | 17.65 | 12 | 0.13 |
| *Anaerotruncus* | 3.71 | 11 | 0.98 |
| *Bacteroides* | 5.87 | 6 | 0.44 |
| *Barnesiella* | 12.01 | 11 | 0.36 |
| *Bifidobacterium* | 11.23 | 12 | 0.51 |
| *Bilophila* | 10.75 | 12 | 0.55 |
| *Blautia* | 5.07 | 11 | 0.93 |
| *Butyricicoccus* | 5.02 | 7 | 0.66 |
| *Butyricimonas* | 10.40 | 12 | 0.58 |
| *Butyrivibrio* | 25.26 | 13 | 0.02 |
| *Candidatus Soleaferrea* | 4.43 | 8 | 0.82 |
| *Catenibacterium* | 3.76 | 3 | 0.29 |
| *Christensenellaceae (R7 group)* | 5.80 | 7 | 0.56 |
| *Clostridium (innocuum group)* | 4.68 | 6 | 0.59 |
| *Clostridium sensustricto 1* | 4.52 | 6 | 0.61 |
| *Collinsella* | 7.98 | 8 | 0.44 |
| *Coprobacter* | 21.28 | 9 | 0.01 |
| *Coprococcus 1* | 6.63 | 10 | 0.76 |
| *Coprococcus 2* | 3.75 | 7 | 0.81 |
| *Coprococcus3* | 11.45 | 8 | 0.18 |
| *Defluviitaleaceae (UCG011)* | 3.51 | 8 | 0.90 |
| *Desulfovibrio* | 6.41 | 9 | 0.70 |
| *Dialister* | 14.65 | 10 | 0.15 |
| *Dorea* | 10.25 | 9 | 0.33 |
| *Eggerthella* | 10.37 | 9 | 0.32 |
| *Eisenbergiella* | 3.33 | 10 | 0.97 |
| *Enterorhabdus* | 3.86 | 5 | 0.57 |
| *Erysipelatoclostridium* | 8.99 | 14 | 0.83 |
| *Erysipelotrichaceae (UCG003)* | 11.72 | 15 | 0.70 |
| *Escherichia Shigella* | 5.84 | 9 | 0.76 |
| *Eubacterium (brachy group)* | 4.74 | 9 | 0.86 |
| *Eubacterium (coprostanoligenes group)* | 11.01 | 13 | 0.61 |
| *Eubacterium (eligens group)* | 1.81 | 5 | 0.87 |
| *Eubacterium (fissicatena group)* | 3.42 | 8 | 0.90 |
| *Eubacterium (hallii group)* | 8.28 | 14 | 0.87 |
| *Eubacterium (nodatum group)* | 22.91 | 10 | 0.01 |
| *Eubacterium (oxidoreducens group)* | 1.65 | 4 | 0.80 |
| *Eubacterium (rectale group)* | 7.44 | 7 | 0.38 |
| *Eubacterium (ruminantium group)* | 22.43 | 17 | 0.17 |
| *Eubacterium (ventriosum group)* | 7.43 | 14 | 0.92 |
| *Eubacterium (xylanophilum group)* | 6.62 | 8 | 0.58 |
| *Faecalibacterium* | 10.30 | 9 | 0.33 |
| *Family XIIIAD (3011 group)* | 11.36 | 12 | 0.50 |
| *Family XIII (UCG001)* | 8.21 | 7 | 0.31 |
| *Flavonifractor* | 0.62 | 3 | 0.89 |
| *Fusicatenibacter* | 15.00 | 17 | 0.60 |
| *Gordonibacter* | 7.28 | 9 | 0.61 |
| *Haemophilus* | 16.14 | 8 | 0.04 |
| *Holdemanella* | 14.01 | 10 | 0.17 |
| *Holdemania* | 11.72 | 13 | 0.55 |
| *Howardella* | 8.54 | 8 | 0.38 |
| *Hungatella* | 4.49 | 4 | 0.34 |
| *Intestinibacter* | 17.51 | 14 | 0.23 |
| *Intestinimonas* | 10.00 | 15 | 0.82 |
| *Lachnoclostridium* | 15.40 | 12 | 0.22 |
| *Lachnospira* | 6.69 | 5 | 0.24 |
| *Lachnospiraceae (FCS020 group)* | 7.87 | 11 | 0.73 |
| *Lachnospiraceae (NC2004 group)* | 10.39 | 8 | 0.24 |
| *Lachnospiraceae (ND3007 group)* | 1.22 | 2 | 0.54 |
| *Lachnospiraceae (NK4A136 group)* | 16.87 | 14 | 0.26 |
| *Lachnospiraceae (UCG001)* | 18.22 | 11 | 0.08 |
| *Lachnospiraceae (UCG004)* | 6.14 | 11 | 0.86 |
| *Lachnospiraceae (UCG008)* | 6.90 | 9 | 0.65 |
| *Lachnospiraceae (UCG010)* | 6.43 | 9 | 0.70 |
| *Lactobacillus* | 5.98 | 7 | 0.54 |
| *Lactococcus* | 15.37 | 8 | 0.05 |
| *Marvinbryantia* | 9.84 | 9 | 0.36 |
| *Methanobrevibacter* | 5.95 | 5 | 0.31 |
| *Odoribacter* | 5.15 | 6 | 0.52 |
| *Olsenella* | 14.21 | 9 | 0.12 |
| *Oscillibacter* | 13.52 | 12 | 0.33 |
| *Oscillospira* | 4.55 | 7 | 0.71 |
| *Oxalobacter* | 21.36 | 10 | 0.02 |
| *Parabacteroides* | 2.33 | 4 | 0.67 |
| *Paraprevotella* | 17.84 | 12 | 0.12 |
| *Parasutterella* | 15.32 | 13 | 0.29 |
| *Peptococcus* | 6.73 | 11 | 0.82 |
| *Phascolarctobacterium* | 6.50 | 7 | 0.48 |
| *Prevotella 7* | 2.46 | 10 | 0.99 |
| *Prevotella 9* | 11.91 | 14 | 0.61 |
| *Rikenellaceae (RC9gut group)* | 3.22 | 10 | 0.98 |
| *Romboutsia* | 14.40 | 12 | 0.28 |
| *Roseburia* | 9.60 | 13 | 0.73 |
| *Ruminiclostridium 5* | 18.94 | 10 | 0.04 |
| *Ruminiclostridium 6* | 11.16 | 13 | 0.60 |
| *Ruminiclostridium 9* | 11.14 | 6 | 0.08 |
| *Ruminococcaceae (NK4A214 group)* | 18.41 | 12 | 0.10 |
| *Ruminococcaceae (UCG002)* | 20.62 | 18 | 0.30 |
| *Ruminococcaceae (UCG003)* | 6.38 | 11 | 0.85 |
| *Ruminococcaceae (UCG004)* | 9.22 | 10 | 0.51 |
| *Ruminococcaceae (UCG005)* | 18.46 | 13 | 0.14 |
| *Ruminococcaceae (UCG009)* | 11.62 | 11 | 0.39 |
| *Ruminococcaceae (UCG010)* | 2.77 | 5 | 0.74 |
| *Ruminococcaceae (UCG011)* | 9.07 | 7 | 0.25 |
| *Ruminococcaceae (UCG013)* | 9.13 | 10 | 0.52 |
| *Ruminococcaceae (UCG014)* | 5.54 | 10 | 0.85 |
| *Ruminococcus 1* | 8.29 | 9 | 0.51 |
| *Ruminococcus 2* | 11.99 | 14 | 0.61 |
| *Ruminococcus (gauvreauii group)* | 5.01 | 10 | 0.89 |
| *Ruminococcus (gnavus group)* | 3.39 | 10 | 0.97 |
| *Ruminococcus (torques group)* | 9.67 | 6 | 0.14 |
| *Sellimonas* | 3.87 | 8 | 0.87 |
| *Senegalimassilia* | 2.32 | 4 | 0.68 |
| *Slackia* | 0.73 | 5 | 0.98 |
| *Streptococcus* | 9.03 | 11 | 0.62 |
| *Subdoligranulum* | 14.22 | 10 | 0.16 |
| *Sutterella* | 11.50 | 11 | 0.40 |
| *Terrisporobacter* | 0.68 | 4 | 0.95 |
| *Turicibacter* | 6.01 | 8 | 0.65 |
| *Tyzzerella 3* | 19.96 | 12 | 0.07 |
| *Veillonella* | 2.33 | 5 | 0.80 |
| *Victivallis* | 8.39 | 9 | 0.50 |
| Df, degree of freedom. | | | |

| **Table S4 Directional horizontal pleiotropy assessed by intercept term in MR Egger regression of the association between gut microbiota and PE.** | | | |
| --- | --- | --- | --- |
| **Bacterial taxa (exposure)** | **Egger_intercept** | **SE** | **P-value** |
| *Actinomyces* | -0.02 | 0.04 | 0.67 |
| *Adlercreutzia* | -0.01 | 0.04 | 0.78 |
| *Akkermansia* | -0.04 | 0.02 | 0.10 |
| *Alistipes* | 0.00 | 0.05 | 0.98 |
| *Allisonella* | -0.01 | 0.05 | 0.92 |
| *Alloprevotella* | 0.09 | 0.17 | 0.61 |
| *Anaerofilum* | 0.03 | 0.05 | 0.55 |
| *Anaerostipes* | 0.03 | 0.03 | 0.27 |
| *Anaerotruncus* | -0.01 | 0.02 | 0.58 |
| *Bacteroides* | 0.01 | 0.05 | 0.84 |
| *Barnesiella* | -0.05 | 0.03 | 0.11 |
| *Bifidobacterium* | 0.01 | 0.02 | 0.75 |
| *Bilophila* | 0.03 | 0.03 | 0.45 |
| *Blautia* | 0.00 | 0.02 | 1.00 |
| *Butyricicoccus* | 0.00 | 0.02 | 0.87 |
| *Butyricimonas* | -0.01 | 0.03 | 0.61 |
| *Butyrivibrio* | -0.05 | 0.04 | 0.22 |
| *Candidatus Soleaferrea* | 0.05 | 0.07 | 0.54 |
| *Catenibacterium* | 0.02 | 0.19 | 0.93 |
| *Christensenellaceae (R7 group)* | 0.02 | 0.04 | 0.65 |
| *Clostridium (innocuum group)* | -0.08 | 0.05 | 0.12 |
| *Clostridium sensustricto 1* | -0.05 | 0.03 | 0.12 |
| *Collinsella* | -0.05 | 0.03 | 0.18 |
| *Coprobacter* | 0.04 | 0.05 | 0.40 |
| *Coprococcus 1* | -0.03 | 0.02 | 0.14 |
| *Coprococcus 2* | 0.01 | 0.06 | 0.86 |
| *Coprococcus3* | -0.01 | 0.05 | 0.91 |
| *Defluviitaleaceae (UCG011)* | 0.03 | 0.03 | 0.42 |
| *Desulfovibrio* | 0.02 | 0.03 | 0.40 |
| *Dialister* | -0.04 | 0.03 | 0.22 |
| *Dorea* | 0.02 | 0.02 | 0.42 |
| *Eggerthella* | 0.00 | 0.04 | 0.92 |
| *Eisenbergiella* | 0.01 | 0.05 | 0.87 |
| *Enterorhabdus* | 0.03 | 0.04 | 0.47 |
| *Erysipelatoclostridium* | -0.01 | 0.02 | 0.72 |
| *Erysipelotrichaceae (UCG003)* | 0.00 | 0.02 | 1.00 |
| *Escherichia Shigella* | 0.03 | 0.02 | 0.22 |
| *Eubacterium (brachy group)* | -0.02 | 0.03 | 0.57 |
| *Eubacterium (coprostanoligenes group)* | -0.01 | 0.03 | 0.73 |
| *Eubacterium (eligens group)* | 0.00 | 0.04 | 0.99 |
| *Eubacterium (fissicatena group)* | -0.03 | 0.04 | 0.55 |
| *Eubacterium (hallii group)* | 0.02 | 0.01 | 0.24 |
| *Eubacterium (nodatum group)* | -0.08 | 0.05 | 0.11 |
| *Eubacterium (oxidoreducens group)* | 0.00 | 0.04 | 0.99 |
| *Eubacterium (rectale group)* | -0.05 | 0.03 | 0.15 |
| *Eubacterium (ruminantium group)* | -0.04 | 0.02 | 0.03 |
| *Eubacterium (ventriosum group)* | 0.04 | 0.03 | 0.24 |
| *Eubacterium (xylanophilum group)* | 0.01 | 0.02 | 0.77 |
| *Faecalibacterium* | 0.03 | 0.02 | 0.14 |
| *Family XIIIAD (3011 group)* | -0.04 | 0.04 | 0.35 |
| *Family XIII (UCG001)* | -0.01 | 0.03 | 0.84 |
| *Flavonifractor* | 0.03 | 0.06 | 0.70 |
| *Fusicatenibacter* | 0.03 | 0.02 | 0.28 |
| *Gordonibacter* | -0.05 | 0.03 | 0.19 |
| *Haemophilus* | -0.02 | 0.03 | 0.53 |
| *Holdemanella* | 0.00 | 0.03 | 0.92 |
| *Holdemania* | 0.04 | 0.02 | 0.10 |
| *Howardella* | -0.03 | 0.04 | 0.45 |
| *Hungatella* | 0.08 | 0.07 | 0.34 |
| *Intestinibacter* | 0.00 | 0.02 | 0.90 |
| *Intestinimonas* | -0.01 | 0.02 | 0.64 |
| *Lachnoclostridium* | -0.03 | 0.03 | 0.30 |
| *Lachnospira* | -0.06 | 0.07 | 0.39 |
| *Lachnospiraceae (FCS020 group)* | -0.02 | 0.02 | 0.29 |
| *Lachnospiraceae (NC2004 group)* | 0.00 | 0.04 | 0.95 |
| *Lachnospiraceae (ND3007 group)* | -0.23 | 0.21 | 0.48 |
| *Lachnospiraceae (NK4A136 group)* | 0.01 | 0.01 | 0.35 |
| *Lachnospiraceae (UCG001)* | 0.03 | 0.04 | 0.50 |
| *Lachnospiraceae (UCG004)* | -0.01 | 0.03 | 0.78 |
| *Lachnospiraceae (UCG008)* | 0.01 | 0.04 | 0.84 |
| *Lachnospiraceae (UCG010)* | 0.02 | 0.03 | 0.44 |
| *Lactobacillus* | -0.04 | 0.02 | 0.14 |
| *Lactococcus* | 0.01 | 0.05 | 0.90 |
| *Marvinbryantia* | 0.00 | 0.04 | 0.92 |
| *Methanobrevibacter* | -0.02 | 0.05 | 0.66 |
| *Odoribacter* | 0.00 | 0.03 | 0.90 |
| *Olsenella* | 0.02 | 0.03 | 0.52 |
| *Oscillibacter* | 0.01 | 0.03 | 0.78 |
| *Oscillospira* | -0.01 | 0.04 | 0.88 |
| *Oxalobacter* | 0.09 | 0.05 | 0.11 |
| *Parabacteroides* | -0.01 | 0.09 | 0.88 |
| *Paraprevotella* | 0.03 | 0.03 | 0.39 |
| *Parasutterella* | 0.01 | 0.02 | 0.48 |
| *Peptococcus* | 0.00 | 0.03 | 0.99 |
| *Phascolarctobacterium* | -0.09 | 0.04 | 0.07 |
| *Prevotella 7* | -0.01 | 0.04 | 0.89 |
| *Prevotella 9* | 0.02 | 0.02 | 0.35 |
| *Rikenellaceae (RC9gut group)* | -0.02 | 0.04 | 0.69 |
| *Romboutsia* | -0.02 | 0.02 | 0.53 |
| *Roseburia* | -0.01 | 0.02 | 0.60 |
| *Ruminiclostridium 5* | 0.02 | 0.04 | 0.61 |
| *Ruminiclostridium 6* | 0.00 | 0.02 | 0.92 |
| *Ruminiclostridium 9* | 0.04 | 0.06 | 0.54 |
| *Ruminococcaceae (NK4A214 group)* | 0.00 | 0.03 | 0.93 |
| *Ruminococcaceae (UCG002)* | -0.01 | 0.02 | 0.43 |
| *Ruminococcaceae (UCG003)* | 0.02 | 0.02 | 0.49 |
| *Ruminococcaceae (UCG004)* | -0.02 | 0.04 | 0.57 |
| *Ruminococcaceae (UCG005)* | 0.01 | 0.02 | 0.78 |
| *Ruminococcaceae (UCG009)* | -0.05 | 0.03 | 0.08 |
| *Ruminococcaceae (UCG010)* | -0.03 | 0.02 | 0.35 |
| *Ruminococcaceae (UCG011)* | 0.08 | 0.04 | 0.09 |
| *Ruminococcaceae (UCG013)* | -0.04 | 0.02 | 0.10 |
| *Ruminococcaceae (UCG014)* | -0.01 | 0.02 | 0.48 |
| *Ruminococcus 1* | -0.01 | 0.02 | 0.79 |
| *Ruminococcus 2* | 0.00 | 0.02 | 0.84 |
| *Ruminococcus (gauvreauii group)* | 0.00 | 0.03 | 1.00 |
| *Ruminococcus (gnavus group)* | -0.01 | 0.04 | 0.77 |
| *Ruminococcus (torques group)* | -0.07 | 0.03 | 0.07 |
| *Sellimonas* | 0.01 | 0.04 | 0.87 |
| *Senegalimassilia* | 0.00 | 0.04 | 0.99 |
| *Slackia* | 0.00 | 0.06 | 0.99 |
| *Streptococcus* | 0.01 | 0.03 | 0.65 |
| *Subdoligranulum* | 0.02 | 0.02 | 0.51 |
| *Sutterella* | 0.02 | 0.03 | 0.53 |
| *Terrisporobacter* | 0.00 | 0.03 | 0.97 |
| *Turicibacter* | 0.03 | 0.04 | 0.44 |
| *Tyzzerella 3* | 0.04 | 0.05 | 0.43 |
| *Veillonella* | 0.01 | 0.07 | 0.86 |
| *Victivallis* | -0.02 | 0.06 | 0.76 |
| MR, Mendelian randomization; PE, preeclampsia-eclampsia; SE, standard error. | | | |

| **Table S5 MR-PRESSO analysis for the association between gut microbiota and PE.** | | | | | | | | |
| --- | --- | --- | --- | --- | --- | --- | --- | --- |
| **Bacterial taxa (exposure)** | **MR Analysis** | **Causal Estimate** | **SD** | **T** | **P-value** | **RSS_obs_** | **Global test P-value** | **Remove SNP** |
| *Actinomyces* | MR-PRESSO | 0.02 | 0.15 | 0.11 | 0.92 | 29.84 | 0.01 | 5 SNP |
| *Actinomyces* | Outlier-corrected MR-PRESSO | -0.10 | 0.14 | -0.71 | 0.50 | - |  |  |
| *Adlercreutzia* | MR-PRESSO | -0.14 | 0.08 | -1.64 | 0.13 | 14.93 | 0.32 |  |
| *Akkermansia* | MR-PRESSO | 0.12 | 0.08 | 1.46 | 0.17 | 10.80 | 0.64 |  |
| *Alistipes* | MR-PRESSO | -0.09 | 0.15 | -0.57 | 0.58 | 21.36 | 0.09 |  |
| *Allisonella* | MR-PRESSO | 0.01 | 0.04 | 0.14 | 0.89 | 4.90 | 0.87 |  |
| *Alloprevotella* | MR-PRESSO | -0.02 | 0.11 | -0.15 | 0.89 | 18.10 | 0.05 |  |
| *Anaerofilum* | MR-PRESSO | 0.03 | 0.07 | 0.45 | 0.66 | 17.55 | 0.22 |  |
| *Anaerostipes* | MR-PRESSO | -0.10 | 0.12 | -0.78 | 0.45 | 25.28 | 0.08 |  |
| *Anaerotruncus* | MR-PRESSO | -0.12 | 0.07 | -1.66 | 0.12 | 8.00 | 0.95 |  |
| *Bacteroides* | MR-PRESSO | -0.04 | 0.11 | -0.39 | 0.71 | 9.22 | 0.58 |  |
| *Barnesiella* | MR-PRESSO | -0.08 | 0.10 | -0.78 | 0.45 | 19.85 | 0.27 |  |
| *Bifidobacterium* | MR-PRESSO | -0.23 | 0.08 | -3.02 | 0.01 | 19.39 | 0.44 |  |
| *Bilophila* | MR-PRESSO | 0.00 | 0.08 | 0.04 | 0.97 | 13.86 | 0.67 |  |
| *Blautia* | MR-PRESSO | -0.01 | 0.08 | -0.15 | 0.88 | 6.04 | 0.93 |  |
| *Butyricicoccus* | MR-PRESSO | 0.00 | 0.10 | 0.03 | 0.97 | 7.22 | 0.71 |  |
| *Butyricimonas* | MR-PRESSO | 0.15 | 0.07 | 2.05 | 0.06 | 17.03 | 0.55 |  |
| *Butyrivibrio* | MR-PRESSO | -0.06 | 0.06 | -1.04 | 0.32 | 28.72 | 0.04 |  |
| *Candidatus Soleaferrea* | MR-PRESSO | -0.04 | 0.05 | -0.79 | 0.44 | 10.72 | 0.80 |  |
| *Catenibacterium* | MR-PRESSO | 0.06 | 0.09 | 0.59 | 0.59 | 6.66 | 0.36 |  |
| *Christensenellaceae (R7 group)* | MR-PRESSO | 0.00 | 0.12 | 0.01 | 0.99 | 8.26 | 0.58 |  |
| *Clostridium (innocuum group)* | MR-PRESSO | 0.00 | 0.05 | 0.04 | 0.97 | 6.06 | 0.78 |  |
| *Clostridium sensustricto 1* | MR-PRESSO | 0.06 | 0.08 | 0.67 | 0.52 | 6.48 | 0.75 |  |
| *Collinsella* | MR-PRESSO | -0.19 | 0.12 | -1.58 | 0.14 | 16.87 | 0.25 |  |
| *Coprobacter* | MR-PRESSO | 0.01 | 0.09 | 0.09 | 0.93 | 26.36 | 0.03 | 3 SNP |
| *Coprobacter* | Outlier-corrected MR-PRESSO | -0.04 | 0.08 | -0.56 | 0.58 | - |  |  |
| *Coprococcus 1* | MR-PRESSO | 0.00 | 0.07 | -0.02 | 0.99 | 8.40 | 0.84 |  |
| *Coprococcus 2* | MR-PRESSO | -0.07 | 0.09 | -0.77 | 0.46 | 9.38 | 0.61 |  |
| *Coprococcus 3* | MR-PRESSO | -0.05 | 0.13 | -0.34 | 0.74 | 14.38 | 0.24 |  |
| *Defluviitaleaceae (UCG011)* | MR-PRESSO | -0.04 | 0.06 | -0.60 | 0.56 | 5.85 | 0.85 |  |
| *Desulfovibrio* | MR-PRESSO | 0.11 | 0.07 | 1.46 | 0.17 | 8.95 | 0.70 |  |
| *Dialister* | MR-PRESSO | -0.02 | 0.11 | -0.16 | 0.87 | 18.30 | 0.18 |  |
| *Dialister* | MR-PRESSO | 0.05 | 0.11 | 0.50 | 0.63 | 12.73 | 0.51 |  |
| *Eggerthella* | MR-PRESSO | -0.03 | 0.07 | -0.35 | 0.74 | 12.98 | 0.31 |  |
| *Eisenbergiella* | MR-PRESSO | -0.03 | 0.03 | -0.78 | 0.45 | 4.13 | 0.99 |  |
| *Enterorhabdus* | MR-PRESSO | -0.22 | 0.07 | -3.03 | 0.02 | 8.20 | 0.64 |  |
| *Erysipelatoclostridium* | MR-PRESSO | -0.07 | 0.06 | -1.28 | 0.22 | 10.68 | 0.85 |  |
| *Erysipelotrichaceae (UCG003)* | MR-PRESSO | 0.10 | 0.07 | 1.38 | 0.19 | 13.86 | 0.71 |  |
| *Escherichia Shigella* | MR-PRESSO | -0.12 | 0.07 | -1.77 | 0.10 | 10.90 | 0.81 |  |
| *Eubacterium (brachy group)* | MR-PRESSO | -0.02 | 0.04 | -0.40 | 0.70 | 5.87 | 0.90 |  |
| *Eubacterium (coprostanoligenes group)* | MR-PRESSO | 0.07 | 0.10 | 0.74 | 0.47 | 12.56 | 0.62 |  |
| *Eubacterium (eligens group)* | MR-PRESSO | 0.04 | 0.12 | 0.38 | 0.71 | 16.08 | 0.17 |  |
| *Eubacterium (fissicatena group)* | MR-PRESSO | 0.05 | 0.04 | 1.30 | 0.23 | 4.38 | 0.91 |  |
| *Eubacterium (hallii group)* | MR-PRESSO | -0.04 | 0.06 | -0.71 | 0.49 | 11.31 | 0.83 |  |
| *Eubacterium (nodatum group)* | MR-PRESSO | -0.01 | 0.08 | -0.08 | 0.94 | 27.34 | 0.02 | 4 SNP |
| *Eubacterium (nodatum group)* | Outlier-corrected MR-PRESSO | 0.04 | 0.06 | 0.69 | 0.51 | - |  |  |
| *Eubacterium (oxidoreducens group)* | MR-PRESSO | -0.09 | 0.05 | -1.59 | 0.17 | 3.02 | 0.85 |  |
| *Eubacterium (rectale group)* | MR-PRESSO | 0.00 | 0.10 | 0.02 | 0.98 | 13.15 | 0.47 |  |
| *Eubacterium (ruminantium group)* | MR-PRESSO | -0.03 | 0.06 | -0.46 | 0.65 | 27.47 | 0.13 |  |
| *Eubacterium (ventriosum group)* | MR-PRESSO | -0.25 | 0.07 | -3.70 | 0.00 | 10.06 | 0.89 |  |
| *Eubacterium (xylanophilum group)* | MR-PRESSO | 0.00 | 0.09 | 0.04 | 0.97 | 12.99 | 0.46 |  |
| *Faecalibacterium* | MR-PRESSO | 0.09 | 0.08 | 1.09 | 0.30 | 12.18 | 0.61 |  |
| *Family XIII (AD3011 group)* | MR-PRESSO | -0.12 | 0.09 | -1.30 | 0.21 | 16.20 | 0.42 |  |
| *Family XIII (UCG001)* | MR-PRESSO | -0.06 | 0.10 | -0.58 | 0.58 | 10.79 | 0.50 |  |
| *Flavonifractor* | MR-PRESSO | 0.07 | 0.08 | 0.83 | 0.44 | 4.44 | 0.78 |  |
| *Fusicatenibacter* | MR-PRESSO | -0.07 | 0.08 | -0.81 | 0.43 | 16.82 | 0.66 |  |
| *Gordonibacter* | MR-PRESSO | 0.04 | 0.04 | 0.91 | 0.38 | 11.24 | 0.66 |  |
| *Haemophilus* | MR-PRESSO | 0.03 | 0.09 | 0.36 | 0.73 | 25.27 | 0.05 |  |
| *Holdemanella* | MR-PRESSO | 0.05 | 0.07 | 0.71 | 0.49 | 19.92 | 0.24 |  |
| *Holdemania* | MR-PRESSO | -0.13 | 0.07 | -1.87 | 0.08 | 19.97 | 0.36 |  |
| *Howardella* | MR-PRESSO | -0.06 | 0.05 | -1.09 | 0.31 | 11.50 | 0.42 |  |
| *Hungatella* | MR-PRESSO | 0.02 | 0.09 | 0.21 | 0.85 | 7.30 | 0.39 |  |
| *Intestinibacter* | MR-PRESSO | -0.16 | 0.09 | -1.78 | 0.10 | 19.78 | 0.26 |  |
| *Intestinimonas* | MR-PRESSO | -0.10 | 0.05 | -1.74 | 0.10 | 11.88 | 0.92 |  |
| *Lachnoclostridium* | MR-PRESSO | -0.09 | 0.11 | -0.85 | 0.41 | 19.85 | 0.27 |  |
| *Lachnospira* | MR-PRESSO | 0.11 | 0.16 | 0.71 | 0.50 | 8.99 | 0.36 |  |
| *Lachnospiraceae (FCS020 group)* | MR-PRESSO | -0.05 | 0.08 | -0.68 | 0.50 | 14.98 | 0.63 |  |
| *Lachnospiraceae (NC2004 group)* | MR-PRESSO | 0.02 | 0.08 | 0.20 | 0.85 | 13.67 | 0.29 |  |
| *Lachnospiraceae (ND3007 group)* | MR-PRESSO | Not enough instrumental variables | | | | | | |
| *Lachnospiraceae (NK4A136 group)* | MR-PRESSO | -0.23 | 0.09 | -2.64 | 0.02 | 21.66 | 0.24 |  |
| *Lachnospiraceae (UCG001)* | MR-PRESSO | 0.09 | 0.09 | 1.01 | 0.33 | 25.56 | 0.08 |  |
| *Lachnospiraceae (UCG004)* | MR-PRESSO | 0.01 | 0.08 | 0.16 | 0.87 | 8.39 | 0.84 |  |
| *Lachnospiraceae (UCG008)* | MR-PRESSO | -0.03 | 0.06 | -0.46 | 0.65 | 10.49 | 0.55 |  |
| *Lachnospiraceae (UCG010)* | MR-PRESSO | -0.01 | 0.11 | -0.11 | 0.92 | 15.85 | 0.28 |  |
| *Lactobacillus* | MR-PRESSO | -0.05 | 0.07 | -0.82 | 0.43 | 10.50 | 0.48 |  |
| *Lactococcus* | MR-PRESSO | 0.02 | 0.07 | 0.30 | 0.77 | 18.73 | 0.13 |  |
| *Marvinbryantia* | MR-PRESSO | -0.02 | 0.10 | -0.17 | 0.87 | 12.85 | 0.43 |  |
| *Methanobrevibacter* | MR-PRESSO | -0.12 | 0.07 | -1.88 | 0.10 | 8.63 | 0.53 |  |
| *Odoribacter* | MR-PRESSO | 0.13 | 0.13 | 1.02 | 0.34 | 12.64 | 0.32 |  |
| *Olsenella* | MR-PRESSO | 0.10 | 0.07 | 1.50 | 0.17 | 19.10 | 0.11 |  |
| *Oscillibacter* | MR-PRESSO | -0.01 | 0.07 | -0.10 | 0.92 | 20.36 | 0.28 |  |
| *Oscillospira* | MR-PRESSO | 0.01 | 0.08 | 0.07 | 0.95 | 7.54 | 0.64 |  |
| *Oxalobacter* | MR-PRESSO | 0.06 | 0.08 | 0.81 | 0.44 | 26.86 | 0.02 | 1 SNP |
| *Oxalobacter* | Outlier-corrected MR-PRESSO | 0.00 | 0.06 | -0.07 | 0.94 | - |  |  |
| *Parabacteroides* | MR-PRESSO | -0.12 | 0.10 | -1.21 | 0.27 | 5.36 | 0.78 |  |
| *Paraprevotella* | MR-PRESSO | 0.03 | 0.08 | 0.34 | 0.74 | 20.76 | 0.12 |  |
| *Parasutterella* | MR-PRESSO | 0.04 | 0.08 | 0.47 | 0.65 | 19.20 | 0.36 |  |
| *Peptococcus* | MR-PRESSO | 0.05 | 0.05 | 1.11 | 0.29 | 15.17 | 0.58 |  |
| *Phascolarctobacterium* | MR-PRESSO | 0.05 | 0.09 | 0.58 | 0.57 | 10.51 | 0.50 |  |
| *Prevotella 7* | MR-PRESSO | -0.03 | 0.04 | -0.70 | 0.50 | 7.18 | 0.89 |  |
| *Prevotella 9* | MR-PRESSO | 0.12 | 0.06 | 2.09 | 0.05 | 15.94 | 0.66 |  |
| *Rikenellaceae (RC9 gut group)* | MR-PRESSO | -0.03 | 0.03 | -1.05 | 0.32 | 5.90 | 0.97 |  |
| *Romboutsia* | MR-PRESSO | -0.01 | 0.10 | -0.14 | 0.89 | 17.22 | 0.25 |  |
| *Roseburia* | MR-PRESSO | 0.03 | 0.07 | 0.37 | 0.72 | 11.59 | 0.86 |  |
| *Ruminiclostridium 5* | MR-PRESSO | 0.03 | 0.11 | 0.32 | 0.75 | 25.11 | 0.09 |  |
| *Ruminiclostridium 6* | MR-PRESSO | -0.03 | 0.08 | -0.40 | 0.70 | 12.59 | 0.73 |  |
| *Ruminiclostridium 9* | MR-PRESSO | -0.16 | 0.12 | -1.33 | 0.21 | 18.41 | 0.21 |  |
| *Ruminococcaceae (NK4A214 group)* | MR-PRESSO | -0.07 | 0.10 | -0.69 | 0.50 | 25.69 | 0.11 |  |
| *Ruminococcaceae (UCG002)* | MR-PRESSO | -0.11 | 0.08 | -1.37 | 0.19 | 29.09 | 0.24 |  |
| *Ruminococcaceae (UCG003)* | MR-PRESSO | -0.18 | 0.07 | -2.59 | 0.02 | 9.43 | 0.83 |  |
| *Ruminococcaceae (UCG004)* | MR-PRESSO | -0.08 | 0.08 | -1.12 | 0.29 | 11.18 | 0.60 |  |
| *Ruminococcaceae (UCG005)* | MR-PRESSO | 0.01 | 0.09 | 0.16 | 0.88 | 22.57 | 0.22 |  |
| *Ruminococcaceae (UCG009)* | MR-PRESSO | 0.06 | 0.07 | 0.83 | 0.42 | 19.39 | 0.24 |  |
| *Ruminococcaceae (UCG010)* | MR-PRESSO | -0.09 | 0.08 | -1.12 | 0.30 | 4.94 | 0.83 |  |
| *Ruminococcaceae (UCG011)* | MR-PRESSO | 0.02 | 0.07 | 0.23 | 0.82 | 12.10 | 0.27 |  |
| *Ruminococcaceae (UCG013)* | MR-PRESSO | -0.05 | 0.08 | -0.64 | 0.53 | 11.70 | 0.70 |  |
| *Ruminococcaceae (UCG014)* | MR-PRESSO | 0.01 | 0.07 | 0.21 | 0.84 | 15.84 | 0.60 |  |
| *Ruminococcus (gauvreauii group)* | MR-PRESSO | 0.03 | 0.11 | 0.31 | 0.76 | 18.42 | 0.17 |  |
| *Ruminococcus (gnavus group)* | MR-PRESSO | 0.02 | 0.04 | 0.48 | 0.64 | 4.03 | 0.97 |  |
| *Ruminococcus (torques group)* | MR-PRESSO | 0.04 | 0.13 | 0.30 | 0.77 | 14.56 | 0.29 |  |
| *Ruminococcus 1* | MR-PRESSO | -0.09 | 0.08 | -1.14 | 0.27 | 12.81 | 0.60 |  |
| *Ruminococcus 2* | MR-PRESSO | 0.04 | 0.07 | 0.54 | 0.59 | 13.32 | 0.64 |  |
| *Sellimonas* | MR-PRESSO | -0.04 | 0.03 | -1.35 | 0.21 | 4.87 | 0.92 |  |
| *Senegalimassilia* | MR-PRESSO | -0.11 | 0.07 | -1.48 | 0.18 | 5.95 | 0.72 |  |
| *Slackia* | MR-PRESSO | 0.02 | 0.03 | 0.80 | 0.45 | 1.28 | 1.00 |  |
| *Streptococcus* | MR-PRESSO | 0.07 | 0.11 | 0.66 | 0.52 | 18.30 | 0.29 |  |
| *Subdoligranulum* | MR-PRESSO | 0.09 | 0.12 | 0.81 | 0.43 | 21.94 | 0.14 |  |
| *Sutterella* | MR-PRESSO | 0.05 | 0.10 | 0.48 | 0.64 | 13.61 | 0.42 |  |
| *Terrisporobacter* | MR-PRESSO | 0.07 | 0.07 | 0.93 | 0.40 | 3.80 | 0.76 |  |
| *Turicibacter* | MR-PRESSO | 0.02 | 0.06 | 0.36 | 0.72 | 9.62 | 0.77 |  |
| *Tyzzerella 3* | MR-PRESSO | -0.11 | 0.06 | -1.94 | 0.07 | 30.22 | 0.06 |  |
| *Veillonella* | MR-PRESSO | -0.02 | 0.08 | -0.30 | 0.77 | 5.62 | 0.76 |  |
| *Victivallis* | MR-PRESSO | 0.04 | 0.05 | 0.88 | 0.40 | 11.52 | 0.57 |  |
| MR, Mendelian randomization; PE, preeclampsia and eclampsia; SD, standard deviation; RSSobs, observed residual sum of squares; SNP, single nucleotide polymorphism. | | | | | | | | |

| **Table S6 Instrumental variables used in the MR analysis of the association between PE and gut microbiota.** | | | | | | | | | | |
| --- | --- | --- | --- | --- | --- | --- | --- | --- | --- | --- |
| **Bacterial taxa (outcome)** | **SNP** | **Effect allele** | **Other allele** | **MAF** | **Exposure (PE)** | | | **Outcome (Bacterial taxa)** | | |
|  |  |  |  |  | **Beta** | **SE** | **P-value** | **Beta** | **SE** | **P-value** |
| *Adlercreutzia* | rs12775642 | A | G | 0.32 | 0.11 | 0.02 | 3.21E-08 | 0.00 | 0.02 | 0.89 |
| *Adlercreutzia* | rs167479 | G | T | 0.57 | 0.10 | 0.02 | 1.63E-07 | -0.02 | 0.02 | 0.12 |
| *Adlercreutzia* | rs1417578 | G | A | 0.22 | -0.12 | 0.02 | 2.52E-07 | 0.00 | 0.02 | 0.86 |
| *Adlercreutzia* | rs45603435 | G | A | 0.15 | -0.14 | 0.03 | 4.41E-07 | -0.03 | 0.02 | 0.24 |
| *Adlercreutzia* | rs17289318 | T | C | 0.07 | -0.19 | 0.04 | 8.40E-07 | -0.01 | 0.03 | 0.89 |
| *Adlercreutzia* | rs7350699 | T | G | 0.17 | 0.12 | 0.02 | 1.21E-06 | 0.00 | 0.02 | 0.94 |
| *Adlercreutzia* | rs8111930 | G | A | 0.92 | -0.16 | 0.03 | 1.45E-06 | 0.02 | 0.02 | 0.47 |
| *Adlercreutzia* | rs116510165 | A | G | 0.05 | -0.23 | 0.05 | 1.79E-06 | 0.05 | 0.04 | 0.21 |
| *Adlercreutzia* | rs11650519 | C | T | 0.34 | 0.10 | 0.02 | 1.95E-06 | 0.03 | 0.02 | 0.16 |
| *Adlercreutzia* | rs7027115 | G | A | 0.37 | 0.09 | 0.02 | 2.56E-06 | 0.01 | 0.02 | 0.49 |
| *Adlercreutzia* | rs619624 | G | T | 0.41 | -0.09 | 0.02 | 4.08E-06 | -0.03 | 0.02 | 0.09 |
| *Adlercreutzia* | rs10192327 | C | A | 0.30 | -0.10 | 0.02 | 4.16E-06 | 0.00 | 0.02 | 0.88 |
| *Adlercreutzia* | rs1874490 | C | T | 0.15 | 0.12 | 0.03 | 6.09E-06 | -0.02 | 0.02 | 0.42 |
| *Adlercreutzia* | rs11786021 | C | T | 0.21 | 0.10 | 0.02 | 7.89E-06 | 0.00 | 0.02 | 0.93 |
| *Adlercreutzia* | rs72868846 | G | A | 0.17 | -0.12 | 0.03 | 7.91E-06 | 0.00 | 0.02 | 0.97 |
| *Adlercreutzia* | rs9972653 | T | G | 0.40 | 0.09 | 0.02 | 8.64E-06 | -0.03 | 0.02 | 0.03 |
| *Bifidobacterium* | rs12775642 | A | G | 0.32 | 0.11 | 0.02 | 3.21E-08 | -0.02 | 0.01 | 0.18 |
| *Bifidobacterium* | rs167479 | G | T | 0.57 | 0.10 | 0.02 | 1.63E-07 | 0.00 | 0.01 | 0.72 |
| *Bifidobacterium* | rs1417578 | G | A | 0.22 | -0.12 | 0.02 | 2.52E-07 | 0.01 | 0.01 | 0.45 |
| *Bifidobacterium* | rs45603435 | G | A | 0.15 | -0.14 | 0.03 | 4.41E-07 | -0.01 | 0.02 | 0.49 |
| *Bifidobacterium* | rs17289318 | T | C | 0.07 | -0.19 | 0.04 | 8.40E-07 | 0.00 | 0.02 | 0.98 |
| *Bifidobacterium* | rs7350699 | T | G | 0.17 | 0.12 | 0.02 | 1.21E-06 | 0.01 | 0.01 | 0.66 |
| *Bifidobacterium* | rs8111930 | G | A | 0.92 | -0.16 | 0.03 | 1.45E-06 | 0.00 | 0.02 | 0.96 |
| *Bifidobacterium* | rs116510165 | A | G | 0.05 | -0.23 | 0.05 | 1.79E-06 | 0.01 | 0.03 | 0.70 |
| *Bifidobacterium* | rs11650519 | C | T | 0.34 | 0.10 | 0.02 | 1.95E-06 | -0.01 | 0.01 | 0.58 |
| *Bifidobacterium* | rs7027115 | G | A | 0.37 | 0.09 | 0.02 | 2.56E-06 | 0.00 | 0.01 | 0.84 |
| *Bifidobacterium* | rs619624 | G | T | 0.41 | -0.09 | 0.02 | 4.08E-06 | -0.01 | 0.01 | 0.50 |
| *Bifidobacterium* | rs10192327 | C | A | 0.30 | -0.10 | 0.02 | 4.16E-06 | 0.00 | 0.01 | 0.82 |
| *Bifidobacterium* | rs1874490 | C | T | 0.15 | 0.12 | 0.03 | 6.09E-06 | 0.03 | 0.02 | 0.07 |
| *Bifidobacterium* | rs11786021 | C | T | 0.21 | 0.10 | 0.02 | 7.89E-06 | 0.02 | 0.01 | 0.12 |
| *Bifidobacterium* | rs72868846 | G | A | 0.17 | -0.12 | 0.03 | 7.91E-06 | -0.01 | 0.02 | 0.58 |
| *Bifidobacterium* | rs9972653 | T | G | 0.40 | 0.09 | 0.02 | 8.64E-06 | -0.01 | 0.01 | 0.36 |
| *Collinsella* | rs12775642 | A | G | 0.32 | 0.11 | 0.02 | 3.21E-08 | -0.03 | 0.01 | 0.04 |
| *Collinsella* | rs167479 | G | T | 0.57 | 0.10 | 0.02 | 1.63E-07 | 0.00 | 0.01 | 0.94 |
| *Collinsella* | rs1417578 | G | A | 0.22 | -0.12 | 0.02 | 2.52E-07 | 0.01 | 0.01 | 0.35 |
| *Collinsella* | rs45603435 | G | A | 0.15 | -0.14 | 0.03 | 4.41E-07 | -0.01 | 0.02 | 0.46 |
| *Collinsella* | rs17289318 | T | C | 0.07 | -0.19 | 0.04 | 8.40E-07 | 0.01 | 0.02 | 0.77 |
| *Collinsella* | rs7350699 | T | G | 0.17 | 0.12 | 0.02 | 1.21E-06 | -0.02 | 0.01 | 0.12 |
| *Collinsella* | rs8111930 | G | A | 0.92 | -0.16 | 0.03 | 1.45E-06 | -0.01 | 0.02 | 0.69 |
| *Collinsella* | rs116510165 | A | G | 0.05 | -0.23 | 0.05 | 1.79E-06 | 0.04 | 0.03 | 0.19 |
| *Collinsella* | rs11650519 | C | T | 0.34 | 0.10 | 0.02 | 1.95E-06 | 0.01 | 0.01 | 0.63 |
| *Collinsella* | rs7027115 | G | A | 0.37 | 0.09 | 0.02 | 2.56E-06 | 0.00 | 0.01 | 0.86 |
| *Collinsella* | rs619624 | G | T | 0.41 | -0.09 | 0.02 | 4.08E-06 | 0.00 | 0.01 | 0.81 |
| *Collinsella* | rs10192327 | C | A | 0.30 | -0.10 | 0.02 | 4.16E-06 | 0.00 | 0.01 | 1.00 |
| *Collinsella* | rs1874490 | C | T | 0.15 | 0.12 | 0.03 | 6.09E-06 | -0.01 | 0.02 | 0.40 |
| *Collinsella* | rs11786021 | C | T | 0.21 | 0.10 | 0.02 | 7.89E-06 | -0.02 | 0.02 | 0.28 |
| *Collinsella* | rs72868846 | G | A | 0.17 | -0.12 | 0.03 | 7.91E-06 | 0.00 | 0.02 | 0.80 |
| *Collinsella* | rs9972653 | T | G | 0.40 | 0.09 | 0.02 | 8.64E-06 | -0.01 | 0.01 | 0.25 |
| *Enterorhabdus* | rs12775642 | A | G | 0.32 | 0.11 | 0.02 | 3.21E-08 | -0.01 | 0.02 | 0.51 |
| *Enterorhabdus* | rs167479 | G | T | 0.57 | 0.10 | 0.02 | 1.63E-07 | -0.02 | 0.02 | 0.33 |
| *Enterorhabdus* | rs1417578 | G | A | 0.22 | -0.12 | 0.02 | 2.52E-07 | 0.00 | 0.02 | 0.86 |
| *Enterorhabdus* | rs45603435 | G | A | 0.15 | -0.14 | 0.03 | 4.41E-07 | -0.02 | 0.02 | 0.32 |
| *Enterorhabdus* | rs17289318 | T | C | 0.07 | -0.19 | 0.04 | 8.40E-07 | -0.01 | 0.03 | 0.89 |
| *Enterorhabdus* | rs7350699 | T | G | 0.17 | 0.12 | 0.02 | 1.21E-06 | 0.04 | 0.02 | 0.03 |
| *Enterorhabdus* | rs8111930 | G | A | 0.92 | -0.16 | 0.03 | 1.45E-06 | 0.03 | 0.03 | 0.20 |
| *Enterorhabdus* | rs116510165 | A | G | 0.05 | -0.23 | 0.05 | 1.79E-06 | -0.02 | 0.04 | 0.67 |
| *Enterorhabdus* | rs11650519 | C | T | 0.34 | 0.10 | 0.02 | 1.95E-06 | 0.03 | 0.02 | 0.11 |
| *Enterorhabdus* | rs7027115 | G | A | 0.37 | 0.09 | 0.02 | 2.56E-06 | 0.01 | 0.02 | 0.54 |
| *Enterorhabdus* | rs619624 | G | T | 0.41 | -0.09 | 0.02 | 4.08E-06 | -0.01 | 0.02 | 0.57 |
| *Enterorhabdus* | rs10192327 | C | A | 0.30 | -0.10 | 0.02 | 4.16E-06 | 0.01 | 0.02 | 0.49 |
| *Enterorhabdus* | rs1874490 | C | T | 0.15 | 0.12 | 0.03 | 6.09E-06 | 0.01 | 0.02 | 0.58 |
| *Enterorhabdus* | rs11786021 | C | T | 0.21 | 0.10 | 0.02 | 7.89E-06 | -0.01 | 0.02 | 0.69 |
| *Enterorhabdus* | rs72868846 | G | A | 0.17 | -0.12 | 0.03 | 7.91E-06 | 0.00 | 0.02 | 0.88 |
| *Enterorhabdus* | rs9972653 | T | G | 0.40 | 0.09 | 0.02 | 8.64E-06 | -0.02 | 0.02 | 0.16 |
| *Eubacterium (ventriosum group)* | rs12775642 | A | G | 0.32 | 0.11 | 0.02 | 3.21E-08 | 0.01 | 0.01 | 0.36 |
| *Eubacterium (ventriosum group)* | rs167479 | G | T | 0.57 | 0.10 | 0.02 | 1.63E-07 | 0.01 | 0.01 | 0.32 |
| *Eubacterium (ventriosum group)* | rs1417578 | G | A | 0.22 | -0.12 | 0.02 | 2.52E-07 | 0.00 | 0.01 | 0.84 |
| *Eubacterium (ventriosum group)* | rs45603435 | G | A | 0.15 | -0.14 | 0.03 | 4.41E-07 | 0.01 | 0.02 | 0.63 |
| *Eubacterium (ventriosum group)* | rs17289318 | T | C | 0.07 | -0.19 | 0.04 | 8.40E-07 | -0.02 | 0.02 | 0.47 |
| *Eubacterium (ventriosum group)* | rs7350699 | T | G | 0.17 | 0.12 | 0.02 | 1.21E-06 | 0.00 | 0.01 | 0.79 |
| *Eubacterium (ventriosum group)* | rs8111930 | G | A | 0.92 | -0.16 | 0.03 | 1.45E-06 | -0.01 | 0.02 | 0.46 |
| *Eubacterium (ventriosum group)* | rs116510165 | A | G | 0.05 | -0.23 | 0.05 | 1.79E-06 | 0.08 | 0.03 | 0.00 |
| *Eubacterium (ventriosum group)* | rs11650519 | C | T | 0.34 | 0.10 | 0.02 | 1.95E-06 | 0.01 | 0.01 | 0.57 |
| *Eubacterium (ventriosum group)* | rs7027115 | G | A | 0.37 | 0.09 | 0.02 | 2.56E-06 | 0.00 | 0.01 | 0.86 |
| *Eubacterium (ventriosum group)* | rs619624 | G | T | 0.41 | -0.09 | 0.02 | 4.08E-06 | -0.01 | 0.01 | 0.32 |
| *Eubacterium (ventriosum group)* | rs10192327 | C | A | 0.30 | -0.10 | 0.02 | 4.16E-06 | 0.00 | 0.01 | 1.00 |
| *Eubacterium (ventriosum group)* | rs1874490 | C | T | 0.15 | 0.12 | 0.03 | 6.09E-06 | 0.01 | 0.02 | 0.72 |
| *Eubacterium (ventriosum group)* | rs11786021 | C | T | 0.21 | 0.10 | 0.02 | 7.89E-06 | -0.02 | 0.01 | 0.23 |
| *Eubacterium (ventriosum group)* | rs72868846 | G | A | 0.17 | -0.12 | 0.03 | 7.91E-06 | 0.00 | 0.02 | 0.82 |
| *Eubacterium (ventriosum group)* | rs9972653 | T | G | 0.40 | 0.09 | 0.02 | 8.64E-06 | 0.01 | 0.01 | 0.21 |
| *Lachnospiraceae (NK4A136 group)* | rs12775642 | A | G | 0.32 | 0.11 | 0.02 | 3.21E-08 | 0.00 | 0.01 | 0.71 |
| *Lachnospiraceae (NK4A136 group)* | rs167479 | G | T | 0.57 | 0.10 | 0.02 | 1.63E-07 | 0.00 | 0.01 | 0.70 |
| *Lachnospiraceae (NK4A136 group)* | rs1417578 | G | A | 0.22 | -0.12 | 0.02 | 2.52E-07 | 0.00 | 0.01 | 0.93 |
| *Lachnospiraceae (NK4A136 group)* | rs45603435 | G | A | 0.15 | -0.14 | 0.03 | 4.41E-07 | 0.01 | 0.02 | 0.58 |
| *Lachnospiraceae (NK4A136 group)* | rs17289318 | T | C | 0.07 | -0.19 | 0.04 | 8.40E-07 | -0.01 | 0.02 | 0.52 |
| *Lachnospiraceae (NK4A136 group)* | rs7350699 | T | G | 0.17 | 0.12 | 0.02 | 1.21E-06 | 0.02 | 0.01 | 0.16 |
| *Lachnospiraceae (NK4A136 group)* | rs8111930 | G | A | 0.92 | -0.16 | 0.03 | 1.45E-06 | -0.01 | 0.02 | 0.50 |
| *Lachnospiraceae (NK4A136 group)* | rs116510165 | A | G | 0.05 | -0.23 | 0.05 | 1.79E-06 | 0.01 | 0.03 | 0.58 |
| *Lachnospiraceae (NK4A136 group)* | rs11650519 | C | T | 0.34 | 0.10 | 0.02 | 1.95E-06 | 0.00 | 0.01 | 0.86 |
| *Lachnospiraceae (NK4A136 group)* | rs7027115 | G | A | 0.37 | 0.09 | 0.02 | 2.56E-06 | -0.01 | 0.01 | 0.26 |
| *Lachnospiraceae (NK4A136 group)* | rs619624 | G | T | 0.41 | -0.09 | 0.02 | 4.08E-06 | 0.01 | 0.01 | 0.53 |
| *Lachnospiraceae (NK4A136 group)* | rs10192327 | C | A | 0.30 | -0.10 | 0.02 | 4.16E-06 | 0.01 | 0.01 | 0.66 |
| *Lachnospiraceae (NK4A136 group)* | rs1874490 | C | T | 0.15 | 0.12 | 0.03 | 6.09E-06 | 0.02 | 0.02 | 0.26 |
| *Lachnospiraceae (NK4A136 group)* | rs11786021 | C | T | 0.21 | 0.10 | 0.02 | 7.89E-06 | 0.01 | 0.01 | 0.34 |
| *Lachnospiraceae (NK4A136 group)* | rs72868846 | G | A | 0.17 | -0.12 | 0.03 | 7.91E-06 | 0.01 | 0.02 | 0.50 |
| *Lachnospiraceae (NK4A136 group)* | rs9972653 | T | G | 0.40 | 0.09 | 0.02 | 8.64E-06 | 0.00 | 0.01 | 0.70 |
| *Methanobrevibacter* | rs12775642 | A | G | 0.32 | 0.11 | 0.02 | 3.21E-08 | 0.02 | 0.03 | 0.49 |
| *Methanobrevibacter* | rs167479 | G | T | 0.57 | 0.10 | 0.02 | 1.63E-07 | 0.00 | 0.02 | 0.89 |
| *Methanobrevibacter* | rs1417578 | G | A | 0.22 | -0.12 | 0.02 | 2.52E-07 | -0.01 | 0.03 | 0.68 |
| *Methanobrevibacter* | rs45603435 | G | A | 0.15 | -0.14 | 0.03 | 4.41E-07 | 0.03 | 0.03 | 0.37 |
| *Methanobrevibacter* | rs17289318 | T | C | 0.07 | -0.19 | 0.04 | 8.40E-07 | 0.04 | 0.05 | 0.32 |
| *Methanobrevibacter* | rs7350699 | T | G | 0.17 | 0.12 | 0.02 | 1.21E-06 | 0.00 | 0.03 | 0.98 |
| *Methanobrevibacter* | rs8111930 | G | A | 0.92 | -0.16 | 0.03 | 1.45E-06 | 0.03 | 0.04 | 0.38 |
| *Methanobrevibacter* | rs11650519 | C | T | 0.34 | 0.10 | 0.02 | 1.95E-06 | 0.00 | 0.03 | 0.88 |
| *Methanobrevibacter* | rs7027115 | G | A | 0.37 | 0.09 | 0.02 | 2.56E-06 | -0.03 | 0.02 | 0.23 |
| *Methanobrevibacter* | rs619624 | G | T | 0.41 | -0.09 | 0.02 | 4.08E-06 | -0.02 | 0.02 | 0.47 |
| *Methanobrevibacter* | rs10192327 | C | A | 0.30 | -0.10 | 0.02 | 4.16E-06 | -0.01 | 0.03 | 0.82 |
| *Methanobrevibacter* | rs1874490 | C | T | 0.15 | 0.12 | 0.03 | 6.09E-06 | -0.03 | 0.03 | 0.37 |
| *Methanobrevibacter* | rs11786021 | C | T | 0.21 | 0.10 | 0.02 | 7.89E-06 | 0.02 | 0.03 | 0.43 |
| *Methanobrevibacter* | rs72868846 | G | A | 0.17 | -0.12 | 0.03 | 7.91E-06 | -0.02 | 0.03 | 0.64 |
| *Methanobrevibacter* | rs9972653 | T | G | 0.40 | 0.09 | 0.02 | 8.64E-06 | -0.05 | 0.02 | 0.04 |
| *Tyzzerella 3* | rs12775642 | A | G | 0.32 | 0.11 | 0.02 | 3.21E-08 | -0.02 | 0.02 | 0.27 |
| *Tyzzerella 3* | rs167479 | G | T | 0.57 | 0.10 | 0.02 | 1.63E-07 | 0.00 | 0.02 | 0.85 |
| *Tyzzerella 3* | rs1417578 | G | A | 0.22 | -0.12 | 0.02 | 2.52E-07 | 0.01 | 0.02 | 0.79 |
| *Tyzzerella 3* | rs45603435 | G | A | 0.15 | -0.14 | 0.03 | 4.41E-07 | -0.05 | 0.03 | 0.07 |
| *Tyzzerella 3* | rs17289318 | T | C | 0.07 | -0.19 | 0.04 | 8.40E-07 | 0.00 | 0.04 | 0.93 |
| *Tyzzerella 3* | rs7350699 | T | G | 0.17 | 0.12 | 0.02 | 1.21E-06 | 0.01 | 0.02 | 0.78 |
| *Tyzzerella 3* | rs8111930 | G | A | 0.92 | -0.16 | 0.03 | 1.45E-06 | 0.02 | 0.03 | 0.63 |
| *Tyzzerella 3* | rs116510165 | A | G | 0.05 | -0.23 | 0.05 | 1.79E-06 | -0.01 | 0.05 | 0.80 |
| *Tyzzerella 3* | rs11650519 | C | T | 0.34 | 0.10 | 0.02 | 1.95E-06 | 0.03 | 0.02 | 0.10 |
| *Tyzzerella 3* | rs7027115 | G | A | 0.37 | 0.09 | 0.02 | 2.56E-06 | 0.01 | 0.02 | 0.50 |
| *Tyzzerella 3* | rs619624 | G | T | 0.41 | -0.09 | 0.02 | 4.08E-06 | 0.02 | 0.02 | 0.45 |
| *Tyzzerella 3* | rs10192327 | C | A | 0.30 | -0.10 | 0.02 | 4.16E-06 | 0.00 | 0.02 | 0.81 |
| *Tyzzerella 3* | rs1874490 | C | T | 0.15 | 0.12 | 0.03 | 6.09E-06 | 0.04 | 0.03 | 0.13 |
| *Tyzzerella 3* | rs11786021 | C | T | 0.21 | 0.10 | 0.02 | 7.89E-06 | -0.03 | 0.02 | 0.29 |
| *Tyzzerella 3* | rs72868846 | G | A | 0.17 | -0.12 | 0.03 | 7.91E-06 | 0.02 | 0.03 | 0.59 |
| *Tyzzerella 3* | rs9972653 | T | G | 0.40 | 0.09 | 0.02 | 8.64E-06 | -0.01 | 0.02 | 0.74 |
| MR, Mendelian randomization; PE, preeclampsia-eclampsia; SNP, single nucleotide polymorphism; MAF, minor allele frequency; SE, standard error. | | | | | | | | | | |

| **Table S7 Full result of MR estimates for the association between PE and gut microbiota.** | | | | | | | |
| --- | --- | --- | --- | --- | --- | --- | --- |
| **Bacterial taxa (outcome)** | **MR method** | **No. of SNP** | **F-statistic** | **OR** | **95% CI** | **P-value** | **q-value** |
| *Adlercreutzia* | IVW | 16 | 25.08 | 0.99 | 0.90–1.07 | 0.74 | 0.95 |
| *Adlercreutzia* | MR Egger | 16 |  | 0.90 | 0.61–1.32 | 0.61 | 0.92 |
| *Adlercreutzia* | Weighted median | 16 |  | 1.00 | 0.89–1.13 | 0.94 | 0.98 |
| *Adlercreutzia* | Weighted mode | 16 |  | 1.00 | 0.82–1.22 | 0.98 | 0.98 |
| *Adlercreutzia* | ML | 16 |  | 0.99 | 0.91–1.07 | 0.73 | 0.96 |
| *Adlercreutzia* | cML-MA-BIC | 16 |  | 0.99 | 0.91–1.07 | 0.74 | 0.90 |
| *Bifidobacterium* | IVW | 16 | 5.10 | 1.00 | 0.94–1.07 | 0.95 | 1.00 |
| *Bifidobacterium* | MR Egger | 16 |  | 1.03 | 0.78–1.35 | 0.86 | 1.00 |
| *Bifidobacterium* | Weighted median | 16 |  | 0.97 | 0.89–1.06 | 0.54 | 0.98 |
| *Bifidobacterium* | Weighted mode | 16 |  | 0.97 | 0.84–1.11 | 0.62 | 0.98 |
| *Bifidobacterium* | ML | 16 |  | 1.00 | 0.94–1.07 | 0.95 | 1.00 |
| *Bifidobacterium* | cML-MA-BIC | 16 |  | 1.00 | 0.94–1.07 | 0.95 | 0.95 |
| *Collinsella* | IVW | 16 | 160.42 | 0.94 | 0.88–1.00 | 0.04 | 0.33 |
| *Collinsella* | MR Egger | 16 |  | 0.93 | 0.71–1.22 | 0.61 | 0.92 |
| *Collinsella* | Weighted median | 16 |  | 0.96 | 0.88–1.04 | 0.32 | 0.98 |
| *Collinsella* | Weighted mode | 16 |  | 1.00 | 0.84–1.19 | 0.97 | 0.98 |
| *Collinsella* | ML | 16 |  | 0.94 | 0.88–1.00 | 0.04 | 0.34 |
| *Collinsella* | cML-MA-BIC | 16 |  | 0.93 | 0.88–1.00 | 0.04 | 0.31 |
| *Enterorhabdus* | IVW | 16 | 26.35 | 1.02 | 0.93–1.11 | 0.73 | 0.95 |
| *Enterorhabdus* | MR Egger | 16 |  | 1.08 | 0.73–1.61 | 0.70 | 0.92 |
| *Enterorhabdus* | Weighted median | 16 |  | 1.00 | 0.88–1.13 | 0.98 | 0.98 |
| *Enterorhabdus* | Weighted mode | 16 |  | 0.97 | 0.79–1.19 | 0.76 | 0.98 |
| *Enterorhabdus* | ML | 16 |  | 1.02 | 0.93–1.11 | 0.72 | 0.96 |
| *Enterorhabdus* | cML-MA-BIC | 16 |  | 1.02 | 0.93–1.11 | 0.73 | 0.90 |
| *Eubacterium (ventriosum group)* | IVW | 16 | 35.02 | 1.01 | 0.95–1.08 | 0.65 | 0.95 |
| *Eubacterium (ventriosum group)* | MR Egger | 16 |  | 0.78 | 0.60–1.02 | 0.09 | 0.86 |
| *Eubacterium (ventriosum group)* | Weighted median | 16 |  | 1.04 | 0.96–1.13 | 0.38 | 0.98 |
| *Eubacterium (ventriosum group)* | Weighted mode | 16 |  | 1.07 | 0.94–1.22 | 0.31 | 0.98 |
| *Eubacterium (ventriosum group)* | ML | 16 |  | 1.02 | 0.95–1.08 | 0.63 | 0.96 |
| *Eubacterium (ventriosum group)* | cML-MA-BIC | 16 |  | 1.02 | 0.96–1.09 | 0.51 | 0.90 |
| *Lachnospiraceae (NK4A136 group)* | IVW | 16 | 20.47 | 1.01 | 0.95–1.07 | 0.79 | 0.95 |
| *Lachnospiraceae (NK4A136 group)* | MR Egger | 16 |  | 1.11 | 0.87–1.43 | 0.41 | 0.92 |
| *Lachnospiraceae (NK4A136 group)* | Weighted median | 16 |  | 0.99 | 0.92–1.07 | 0.79 | 0.98 |
| *Lachnospiraceae (NK4A136 group)* | Weighted mode | 16 |  | 0.96 | 0.84–1.09 | 0.50 | 0.98 |
| *Lachnospiraceae (NK4A136 group)* | ML | 16 |  | 1.01 | 0.95–1.07 | 0.79 | 0.96 |
| *Lachnospiraceae (NK4A136 group)* | cML-MA-BIC | 16 |  | 1.01 | 0.95–1.07 | 0.79 | 0.90 |
| *Methanobrevibacter* | IVW | 15 | 69.07 | 0.95 | 0.83–1.08 | 0.40 | 0.95 |
| *Methanobrevibacter* | MR Egger | 15 |  | 0.84 | 0.44–1.61 | 0.61 | 0.92 |
| *Methanobrevibacter* | Weighted median | 15 |  | 1.02 | 0.86–1.22 | 0.79 | 0.98 |
| *Methanobrevibacter* | Weighted mode | 15 |  | 1.09 | 0.80–1.49 | 0.58 | 0.98 |
| *Methanobrevibacter* | ML | 15 |  | 0.95 | 0.83–1.08 | 0.40 | 0.96 |
| *Methanobrevibacter* | cML-MA-BIC | 15 |  | 0.94 | 0.83–1.08 | 0.40 | 0.90 |
| *Tyzzerella 3* | IVW | 16 | 27.71 | 1.02 | 0.92–1.13 | 0.72 | 0.95 |
| *Tyzzerella 3* | MR Egger | 16 |  | 1.10 | 0.71–1.70 | 0.69 | 0.92 |
| *Tyzzerella 3* | Weighted median | 16 |  | 0.99 | 0.85–1.14 | 0.85 | 0.98 |
| *Tyzzerella 3* | Weighted mode | 16 |  | 0.97 | 0.77–1.23 | 0.81 | 0.98 |
| *Tyzzerella 3* | ML | 16 |  | 1.02 | 0.92–1.13 | 0.72 | 0.96 |
| *Tyzzerella 3* | cML-MA-BIC | 16 |  | 1.02 | 0.92–1.13 | 0.72 | 0.90 |
| MR, Mendelian randomization; PE, preeclampsia-eclampsia; SNP, single nucleotide polymorphism; OR, odds ratio; CI, confidence interval; IVW, inverse variance weighted; ML, maximum likelihood. | | | | | | | |

| **Table S8 The heterogeneity of gut microbiota instrumental variables.** | | | |
| --- | --- | --- | --- |
| **Bacterial taxa (outcome)** | **Q** | **df** | **P-value** |
| *Adlercreutzia* | 16.35 | 15 | 0.36 |
| *Bifidobacterium* | 11.28 | 15 | 0.73 |
| *Collinsella* | 9.73 | 15 | 0.84 |
| *Enterorhabdus* | 15.69 | 15 | 0.40 |
| *Eubacterium (ventriosum group)* | 16.19 | 15 | 0.37 |
| *Lachnospiraceae (NK4A136 group)* | 8.32 | 15 | 0.91 |
| *Methanobrevibacter* | 10.61 | 14 | 0.72 |
| *Tyzzerella 3* | 12.06 | 15 | 0.67 |
| Df, degree of freedom. | | | |

| **Table S9 Directional horizontal pleiotropy assessed by intercept term in MR Egger regression of the association between PE and gut microbiota.** | | | |
| --- | --- | --- | --- |
| **Bacterial taxa (outcome)** | **Egger_intercept** | **SE** | **P-value** |
| *Adlercreutzia* | 0.01 | 0.02 | 0.65 |
| *Bifidobacterium* | 0.01 | 0.02 | 0.87 |
| *Collinsella* | 0.01 | 0.02 | 0.96 |
| *Enterorhabdus* | -0.01 | 0.02 | 0.75 |
| *Eubacterium (ventriosum group)* | 0.03 | 0.02 | 0.07 |
| *Lachnospiraceae (NK4A136 group)* | -0.01 | 0.01 | 0.44 |
| *Methanobrevibacter* | 0.01 | 0.04 | 0.73 |
| *Tyzzerella 3* | -0.01 | 0.03 | 0.75 |
| MR, Mendelian randomization; PE, preeclampsia-eclampsia; SE, standard error. | | | |

| **Table S10 MR-PRESSO analysis for the association between PE and gut microbiota.** | | | | | | | |
| --- | --- | --- | --- | --- | --- | --- | --- |
| **Bacterial taxa (outcome)** | **MR Analysis** | **Causal Estimate** | **SD** | **T** | **P-value** | **RSS_obs_** | **Global test P-value** |
| *Adlercreutzia* | MR-PRESSO | -0.03 | 0.04 | -0.77 | 0.45 | 25.47 | 0.26 |
| *Bifidobacterium* | MR-PRESSO | -0.03 | 0.03 | -1.04 | 0.31 | 19.12 | 0.58 |
| *Collinsella* | MR-PRESSO | -0.06 | 0.02 | -2.66 | 0.02 | 13.15 | 0.89 |
| *Enterorhabdus* | MR-PRESSO | 0.01 | 0.05 | 0.26 | 0.80 | 28.97 | 0.15 |
| *Eubacterium (ventriosum group)* | MR-PRESSO | -0.01 | 0.03 | -0.29 | 0.77 | 22.50 | 0.36 |
| *Lachnospiraceae (NK4A136 group)* | MR-PRESSO | 0.01 | 0.02 | 0.15 | 0.88 | 11.94 | 0.93 |
| *Methanobrevibacter* | MR-PRESSO | -0.03 | 0.05 | -0.54 | 0.59 | 15.35 | 0.71 |
| *Tyzzerella 3* | MR-PRESSO | 0.04 | 0.04 | 1.02 | 0.32 | 17.99 | 0.66 |
| MR, Mendelian randomization; PE, preeclampsia-eclampsia; SD, standard deviation; RSS_obs_, observed residual sum of squares. | | | | | | | |
